# Baseline and Disease-Induced Transcriptional Profiles in Children with Sickle Cell Disease

**DOI:** 10.1038/s41598-020-65822-3

**Published:** 2020-06-02

**Authors:** Susan Creary, Chandra L. Shrestha, Kavitha Kotha, Abena Minta, James Fitch, Lisa Jaramillo, Shuzhong Zhang, Swaroop Pinto, Rohan Thompson, Octavio Ramilo, Peter White, M. Asuncion Mejias, Benjamin T. Kopp

**Affiliations:** 1 Center for Innovation in Pediatric Practice, The Abigail Wexner Research Institute, Columbus, OH USA; 2 Division of Hematology and Oncology, Nationwide Children’s Hospital, Columbus, OH USA; 3 Center for Microbial Pathogenesis, The Abigail Wexner Research Institute, Columbus, OH USA; 4 Division of Pulmonary Medicine, Nationwide Children’s Hospital, Columbus, OH USA; 5 The Institute for Genomic Medicine, The Abigail Wexner Research Institute, Columbus, OH USA; 6 Center for Vaccines and Immunity, The Abigail Wexner Research Institute, Columbus, OH USA; 7 Division of Infectious Diseases, Nationwide Children’s Hospital, Columbus, OH USA

**Keywords:** Immunology, Systems biology, Medical research, Pathogenesis

## Abstract

Acute chest syndrome (ACS) is a significant cause of morbidity and mortality in sickle cell disease (SCD), but preventive, diagnostic, and therapeutic options are limited. Further, ACS and acute vasoccclusive pain crises (VOC) have overlapping features, which causes diagnostic dilemmas. We explored changes in gene expression profiles among patients with SCD hospitalized for VOC and ACS episodes to better understand ACS disease pathogenesis. Whole blood RNA-Seq was performed for 20 samples from children with SCD at baseline and during a hospitalization for either an ACS (n = 10) or a VOC episode (n = 10). Respiratory viruses were identified from nasopharyngeal swabs. Functional gene analyses were performed using modular repertoires, IPA, Gene Ontology, and NetworkAnalyst 3.0. The VOC group had a numerically higher percentage of female, older, and hemoglobin SS participants compared to the ACS group. Viruses were detected in 50% of ACS cases and 20% of VOC cases. We identified 3004 transcripts that were differentially expressed during ACS episodes, and 1802 transcripts during VOC episodes. Top canonical pathways during ACS episodes were related to interferon signaling, neuro-inflammation, pattern recognition receptors, and macrophages. Top canonical pathways in patients with VOC included IL-10 signaling, iNOS signaling, IL-6 signaling, and B cell signaling. Several genes related to antimicrobial function were down-regulated during ACS compared to VOC. Gene enrichment nodal interactions demonstrated significantly altered pathways during ACS and VOC. A complex network of changes in innate and adaptive immune gene expression were identified during both ACS and VOC episodes. These results provide unique insights into changes during acute events in children with SCD.

## Introduction

Sickle cell disease (SCD) is a chronic genetic hemoglobin disorder characterized by structural changes in circulating red blood cells. SCD is caused by a single point mutation in the beta globin gene that results in increased red cell rigidity and adhesion and subsequent decreased oxygen delivery. Of the many complications that can result from SCD, vaso-occulsive pain crisis (VOC) is the most common and acute chest syndrome (ACS) is the leading cause of mortality and a top cause of morbidity^1,2^. Approximately 50% of people with SCD will have an episode of ACS during their lifetime^3^. ACS is a vaso-occlusive crisis of the pulmonary vasculature that can result in hypoxia, difficulty breathing, and rapid progression to respiratory insufficiency and failure^4^. ACS is a clinical diagnosis defined as a new infiltrate on chest imaging with at least one of the symptoms of fever, cough, hypoxia, leukocytosis, tachypnea, sputum production, decreasing hemoglobin level, chest pain and/or dyspnea^2,5^. ACS can present as a primary or secondary complication of SCD and 10–20% of hospitalized patients will develop ACS^5,6^, with the primary triggers being concurrent VOC and respiratory infections^4,7,8^.

Despite the frequent and severe nature of ACS and a significant correlation between recurrent ACS episodes and reduced lung function^9^, preventive and therapeutic interventions are limited. Furthermore, ACS diagnostic criteria are non-specific and can be present in patients with VOC without ACS^10,11^. Unfortunately, there are no commercially available biomarkers that reliably predict which patients will develop ACS, and little is known about markers of ACS pathogenesis. These findings suggest the need for more specific clinical and/or biomarkers to identify SCD patients who are at highest risk of developing ACS.

Transcriptomics is a rapidly developing field that has been utilized in a variety of clinical scenarios to predict clinical or therapeutic responses, identify high-risk patients, and monitor changing disease states^12–15^. We conducted a small study to explore changes in whole-blood RNA-Seq profiles that occurred during hospitalization for VOC or ACS episodes to better understand ACS disease pathogenesis in children with SCD. We hypothesized that patients hospitalized for ACS or VOC would have differentially expressed genes during these episodes compared to their baseline, but the gene expression patterns during ACS would be distinct compared to VOC.

## Results

### Patient demographics

Of the 86 children with SCD who enrolled at baseline health and had blood collection, 26 had a hospitalization for either a VOC or an ACS episode and a second blood collection performed. Five of these children were excluded for poor quality blood RNA samples, and 1 child excluded who had a baseline sample obtained after presenting first with ACS. None of the participants admitted for VOC were diagnosed with ACS during their admission. The demographics of the 20 children analyzed are presented in Table 1. The VOC group had a numerically but not significant higher percentage of female, older, and hemoglobin SS participants compared to the ACS group. The VOC group was also more likely to be prescribed hydroxyurea compared to the ACS group. Fifty percent of ACS cases had a virus detected, compared to 20% of VOC cases. Length of stay was significantly longer for VOC cases. Hematologic characteristics for each cohort at baseline and during ACS or VOC events are found in Table 2. Mean absolute neutrophil and absolute monocyte counts significantly increased during ACS episodes, while mean absolute reticulocyte count and hemoglobin significantly decreased during ACS events. Mean absolute monocyte counts significantly increased during VOC episodes, while hemoglobin significantly decreased.

**Table 1:** Cohort Demographics.

	ACS, n = 10	VOC, n = 10	P value
Female	50.00%	90.00%	0.05
Age (mean years)	8.1 ± 6.2	11.6 ± 2.7	0.27
SCD Genotype			
Hemoglobin SS	40.00%	60.00%	0.4
Hemoglobin SC	50.00%	20.00%	0.18
Hemoglobin SB +	10.00%	20.00%	0.56
Prescribed hydroxyurea	30.00%	70.00%	0.08
Viral +	50.00%	20.00%	0.18
Rhino/enterovirus	20.00%	10.00%	
Respiratory syncytial virus (RSV)	10.00%	0.00%	
Human metapneumovirus	10.00%	0.00%	
AH3 (Influenza A)	10.00%	0.00%	
Length of stay (days)	3.2 ± 1.5	5.0 ± 1.9	0.03

**Table 2:** Cohort Hematologic Studies.

Hematologic Parameters (mean ± SD)	ACS n = 10			VOC n = 10		
	Baseline	During ACS	p value	Baseline	During VOC	p value
White Blood Cell Count (×10^3^/µL)	9.3 ± 3.1	12.8 ± 5.3	0.06	10.6 ± 3.8	14.4 ± 8.1	0.08
Absolute Neutrophil Count (×10^3^/mm^3^)	4.5 ± 1.6	8.3 ± 4.7	0.03	5.4 ± 2.3	9.7 ± 6.7	0.06
Absolute Lymphocyte Count (×10^3^/mm^3^)	3.7 ± 1.5	3.0 ± 2.1	0.4	3.9 ± 1.6	3.0 ± 2.3	0.2
Absolute Monocyte Count (×10^3^/mm^3^)	0.7 ± 0.4	1.4 ± 1.0	0.03	1.0 ± 0.5	1.3 ± 1.0	0.05
Hemoglobin (g/dL)	10.3 ± 1.7	9.6 ± 2.1	0.04	9.5 ± 1.5	8.6 ± 2.0	0.03
Absolute Reticulocyte Count (×10^9^/L)	235.4 ± 137.1	163.4 ± 114.9	0.001	297.7 ± 130.7	243.9 ± 128.7	0.2
Platelet Count (×10^3^/µL)	313.1 ± 130.4	288.0 ± 141.4	0.6	360.9 ± 143.7	270.8 ± 129.3	0.08

### SCD gene expression signatures are altered with acute illness

**Figure 1:**
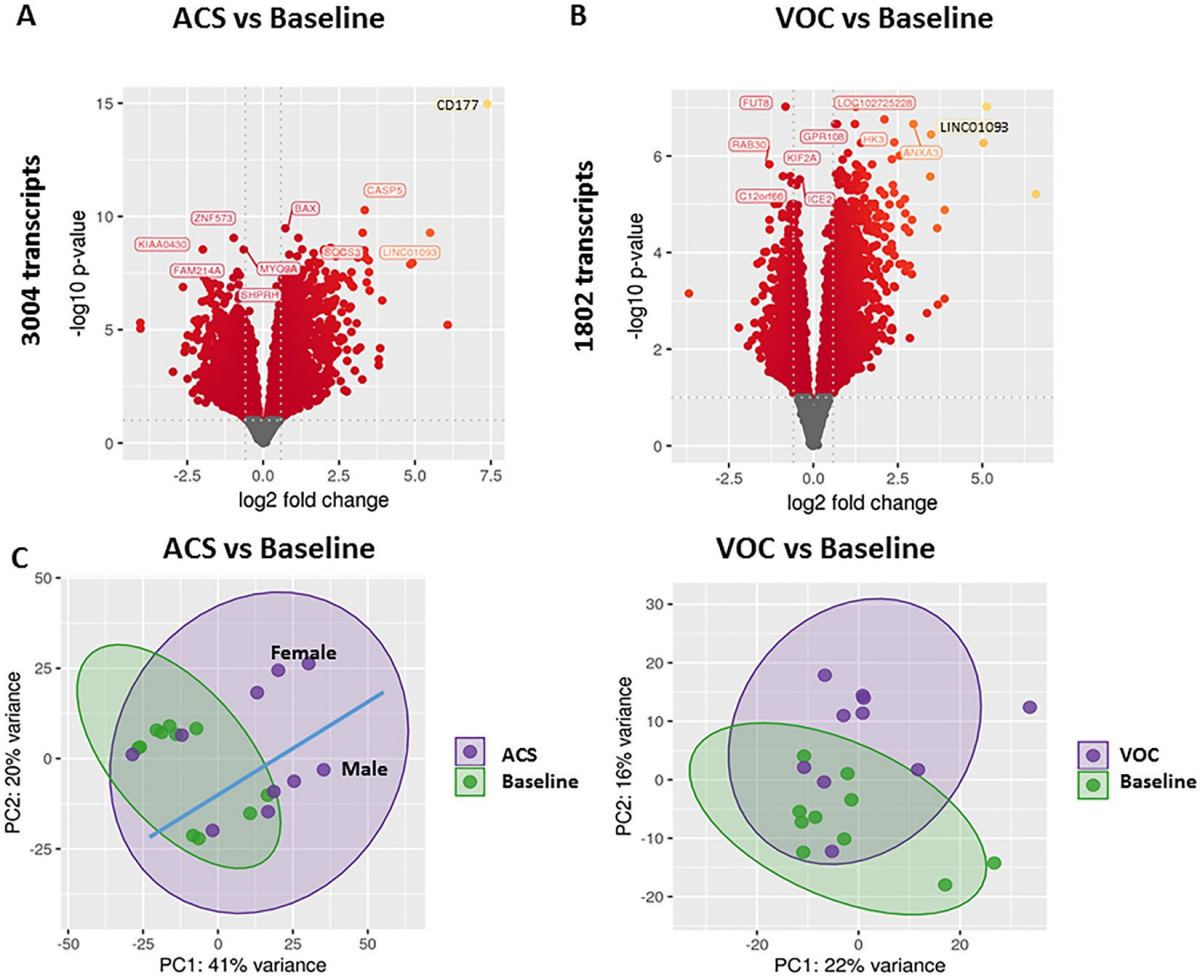
Alterations in global gene expression profiles during ACS and VOC in children with SCD. Volcano plot of changes in whole-blood RNA-Seq gene expression profiles for SCD patients (**A**) during ACS compared to baseline health (3130 transcripts) and (**B**) during VOC compared to baseline health (1802 transcripts). Colored genes represent significant changes in expression, with yellow colors representing greater changes in comparison to red. Log fold changes are presented on the x axis and –log10 p values shown on the y axis. Principal component analysis (PCA) of whole blood RNA-Seq profiles for SCD patients (**C**) during ACS compared to baseline health and (**D**) during VOC compared to baseline health. Grouping are indicated by colors and shading with baseline samples in green in both comparisons. For the ACS to baseline comparison (**C**), further separation by gender is shown by the blue line. The % variance accounted for by the top 2 components are shown on the X and Y axes for both PCA plots.

Patient’s whole blood samples were subjected to RNA-Seq analysis. Changes in patient gene expression profiles from baseline to when they were hospitalized for ACS or VOC were recorded. In the ACS group over 3000 transcripts were differentially expressed during ACS episodes compared to when these patients were at their baseline (Fig. 1A, all significant genes listed in Supplementary Table S1). In the VOC group, over 1800 transcripts were differentially expressed during VOC episodes compared to when these patients were at their baseline (Fig. 1B, all significant genes listed in Supplementary Table S1).

To compare global RNA-Seq profiles according to the incident of ACS or VOC, we used principal component analysis (PCA) to define overall profiles. Patients with ACS demonstrated distinct profile changes compared to their baseline profiles. (Fig. 1C) However, there was a further separation between profiles, with patients clustering into two overall distinct groups (Fig. 1C, demarcated by blue linear line). When analyzing which features contributed to cluster separation (genotype, age, gender, hydroxyurea use), gender was the main driver, with all females included in the top cluster, while the bottom cluster was comprised of all males. Patients with VOC also demonstrated distinct global profiles from baseline (Fig. 1D). Because there were few males in the VOC group, we did not analyze for cluster separation by gender within the VOC group. We also used PCA to compare the overall signatures of ACS and VOC events. There was some heterogeneity amongst the overall profiles with four ACS cases clustering with the VOC cases and six ACS cases clustering separately (Supplemental Fig. S1A). There were no discernible clinical characteristics shared amongst the four ACS cases that clustered with VOC cases.

**Figure 2:**
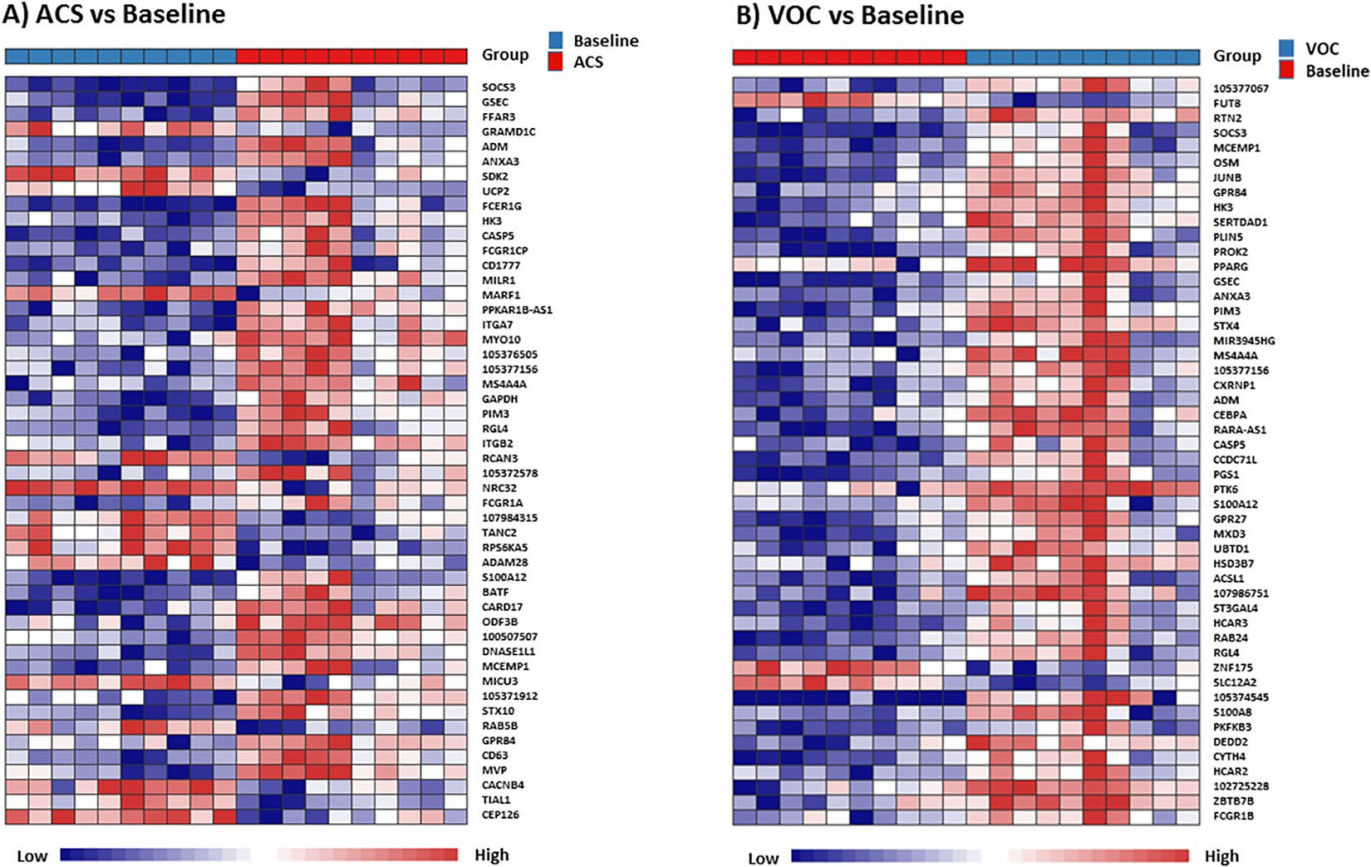
ACS and VOC are associated with distinct regulation of genes in children with SCD. Heat maps of top differentially expressed genes for (**A**) SCD patients during ACS compared to baseline health and (**B**) during VOC compared to baseline health. The top 50 differentially expressed genes are annotated. Each vertical row represents an individual patient with groupings of patients indicated by color at the top of the figures (Group). At the bottom of each figure a legend represents the color continuum scheme used in the heat maps (low to high gene expression). Group clustering determined by Ward’s methods.

Next, we generated heatmaps of the top 50 differentially expressed genes during ACS and VOC episodes. Figure 2A demonstrates a distinct group separation between patients at their baseline and during their ACS episodes. Top differentially expressed genes included CD177 (encodes a cell surface glycoprotein involved in neutrophil activation), caspase 5 (CASP5, involved in apoptosis and anti-viral host defense), suppressor of cytokine signaling 3 (SOCS3, negatively regulates cytokines that signal through the JAK/STAT pathway), free fatty acid receptor 3 (FFAR3, regulation of energy homeostasis and intestinal immunity), and GRAM domain containing 1 C (GRAMD1C, cholesterol transporter). Figure 2B also demonstrates a distinct group separation of RNA-Seq profiles between patients at their baseline and during their VOC episode. Some of the top differentially expressed genes during VOC episodes were similar to the top differentially expressed genes during ACS episodes in Fig. 2A including CD177, SOCS3, CASP5, and annexin A3 (ANXA3, regulates cellular growth and phospholipase A2 signal transduction pathways). However several genes were differentially expressed only in patients with VOC and not in patients with ACS, including oncostatin M (pleiotropic effects including regulation of inflammation and cytokine production) and hexokinase 3 (mediates the initial step of glycolysis). Next, we generated a heatmap of the top differentially regulated genes between ACS and VOC events (Supplemental Fig. S1B). Top differentially expressed genes included ribonuclease A family member 3 (RNASE3) and lactotransferrin, both of which were down-regulated in the ACS group. RNASE3 is a protein with antimicrobial functions and lactotransferrin is an iron-binding protein that also has antimicrobial properties. Last, we generated a heatmap of the top differentially expressed genes at baseline in the ACS group by gender, due to the separation of transcriptomic profiles in children with ACS by gender (Fig. 1C). However, only 34 genes were differentially expressed between these males and females at baseline (Supplementary Fig. S2). Of these, 21 genes had consistent down-regulation in females compared to males. There were no significant differences in gene expression profiles between SCD patients with or without hydroxyurea usage at baseline (not shown).

**Figure 3:**
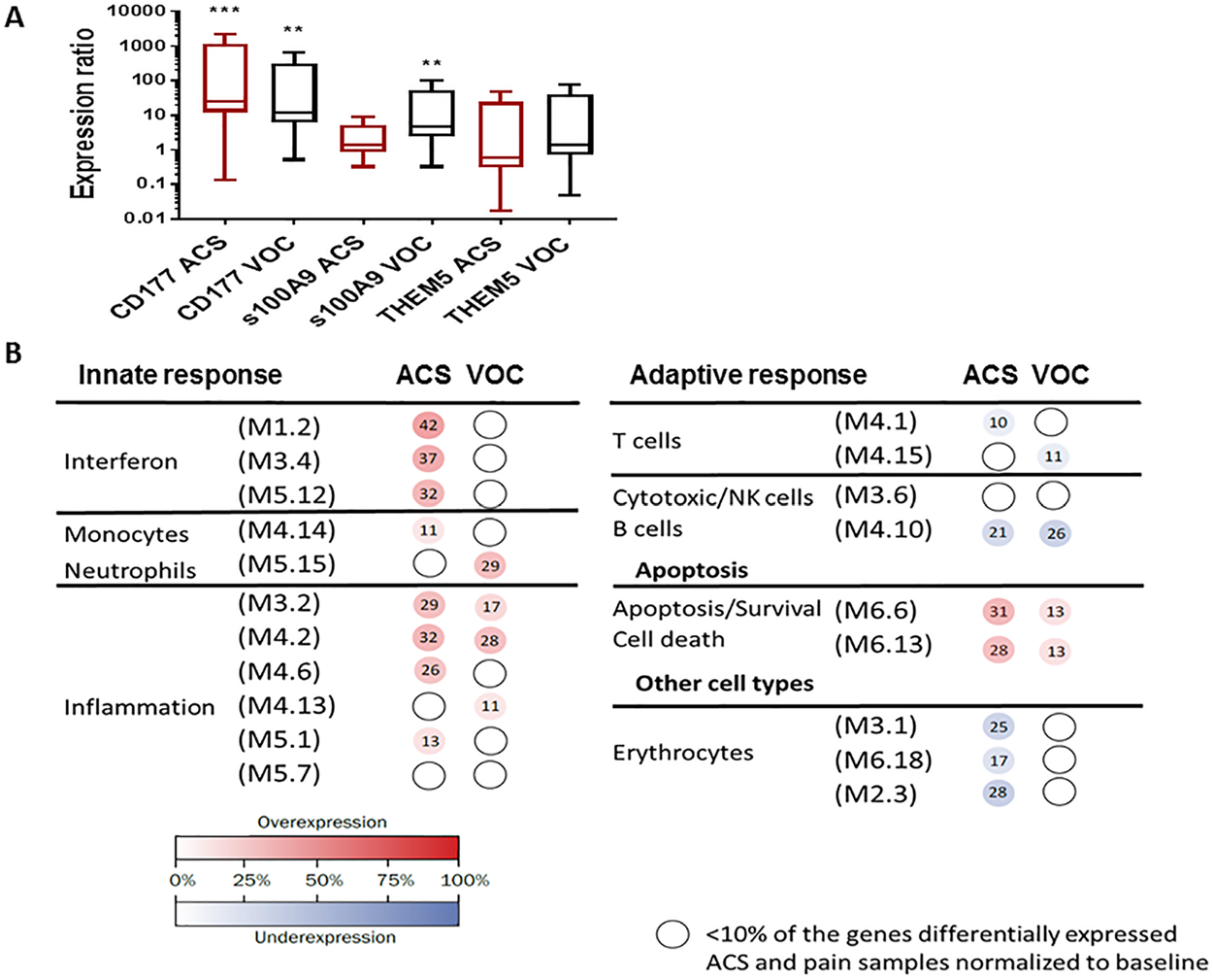
Altered immune-related gene responses during ACS and VOC. (**A**) qRT-PCR of CD177, s100A9, and THEM5 gene expression in 10 SCD patients during ACS or VOC. Results are presented as gene expression ratios of SCD patients during ACS or VOC relative to baseline samples and normalized to the housekeeping genes GAPDH and HPRT1. Statistical significance determined by REST mathematical modeling for each comparison (“**”=p value ≤0.01, “***”=p value <0.001). (**B**) Grouping of significantly altered genes by modular expression in SCD patients during ACS or VOC. Each M# designation represents a cluster of coordinately expressed genes (modules) that share the same biological function^16^. Each group was compared to matched, baseline samples. Both ACS and VOC groups demonstrated significant overexpression of inflammation-related genes and apoptosis genes, and significant under-expression of T and B cell-related genes during these episodes compared to baseline. Patients during ACS demonstrated overexpression of interferon and monocyte-related genes, and under-expression of erythrocyte-related genes. Patients during VOC had over-expression of neutrophil-related genes. The intensity of the modules (dots) indicates the proportion of over-expressed (in red) or under-expressed (in blue) transcripts within each module. Numeric values indicate the exact percentage of transcripts expressed in each specific module. A blank dot indicates that <10% of the genes in the module were differentially expressed.

We then technically validated by qRT-PCR two top differentially expressed genes with available primers (CD177, S100A9) and one control gene (THEM5) in 5 patients from the VOC group and 5 patients from the ACS group. THEM5 was chosen as it is differentially regulated between SCD and non-SCD samples (not shown), but not expected to be differentially regulated during ACS or VOC episodes based on RNA-Seq data. We used REST software to analyze the relative changes in expression of these genes during ACS and VOC episodes. CD177 was significantly overexpressed in patients with SCD during ACS and VOC (Fig. 3A). S100A9 was only upregulated during VOC and not ACS, similar to RNA-Seq data (Fig. 3A). There was no difference in THEM5 expression during ACS or VOC (Fig. 3A). Together, these qRT-PCR data help validate the technical accuracy of our RNA-Seq findings.

### Inflammatory signature alterations during ACS and VOC

To determine the biologic significance of the observed changes in RNA-seq profiles in children during ACS and VOC episodes, we performed modular analysis as described^16^. Modular analysis is a data driven approach based on clusters of coordinately expressed genes (modules) that share the same biological function. Patients with ACS demonstrated overexpression of several modules related to innate immune responses during these episodes, including interferon-related genes, monocytes, and inflammation-related genes (Fig. 3B). In contrast, modules related to adaptive immunity including T cell and B cell-related genes were under-expressed. These data are consistent with significantly increased absolute monocyte counts and relatively decreased absolute lymphocyte counts for the ACS group (Table 2). Further, they demonstrated over-expression of apoptosis-related genes and importantly, suppression of erythrocyte-related genes during ACS. This is consistent with a significant decrease in absolute reticulocyte counts in the ACS group (Table 2). Children in the VOC group demonstrated some shared changes in modular response during their VOC episodes compared to the ACS group, but the VOC group also had unique modules that were differentially expressed. For example, children with VOC demonstrated over-expression of inflammation and apoptosis-related genes, under-expression of B and T cell modules and over-expression of a neutrophil-related module (Fig. 3B**)**. These changes are consistent with a relative increase in absolute neutrophil count and relative decrease in absolute lymphocyte count in the VOC group (Table 2). However, the ACS group had a significant increase in absolute neutrophil count without overexpression of neutrophil-related modules. There were no changes in expression of erythrocyte-related genes in the VOC group, consistent with no change in absolute reticulocyte counts (Table 2). Combined, these results suggest features of shared and unique gene expression changes occur in children during ACS and VOC episodes.

### Pathway analysis reveals unique signatures of disease states in children with SCD

**Figure 4:**
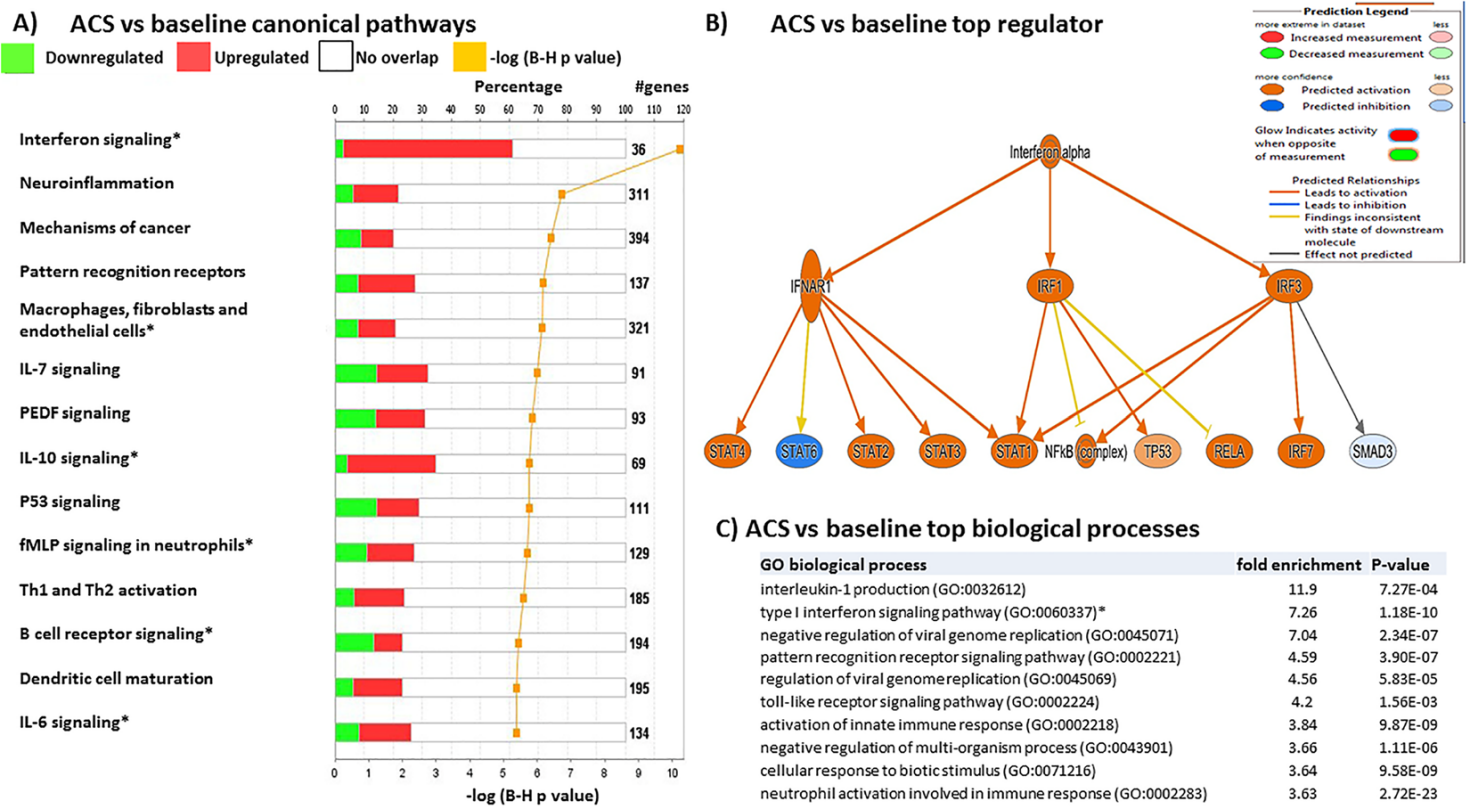
Unique pathways are regulated during ACS. (**A**) IPA analysis of canonical pathways altered during ACS compared to baseline. The top 14 differentially regulated canonical pathways are presented in descending order of statistical significance of −log p values. The % of genes within each pathway significantly altered is shown, with the exact # of genes altered presented at the end of each horizontal row. Upregulated genes are shown in red and downregulated in green. “*” indicates shared pathway with VOC events in Fig. 5. (**B**) Representative network analysis of the top predicted regulator of alterations in interferon signaling genes during ACS. A colored prediction legend is listed to describe predicted interactions. (**C**) Top biological processes determined by gene ontology (GO) analysis for genes regulated during ACS. Biological processes are arranged in descending order of fold enrichment of each pathway. “*” indicates shared pathway with VOC events in Fig. 5.

To confirm the findings derived from the modular analyses, we used Ingenuity Pathway Analysis software (IPA) to identify relevant pathways and upstream regulators of gene function predicted from our observed changes in gene expression. Top canonical pathways in patients during ACS were related to inflammation, including interferon signaling, neuroinflammation, pattern recognition receptors, and macrophage signaling **(**Fig. 4A). The top predicted upstream regulator during ACS was interferon alpha, with several downstream signal transduction and transcription factors affected (Fig. 4B). We verified these changes using a separate bioinformatics approach that utilized the Gene Ontology (GO) knowledgebase. Top predicted biological processes included again type 1 interferon signaling, activation of innate immune responses, and pattern recognition receptor signaling, along with interleukin-1 production (Fig. 4C).

**Figure 5:**
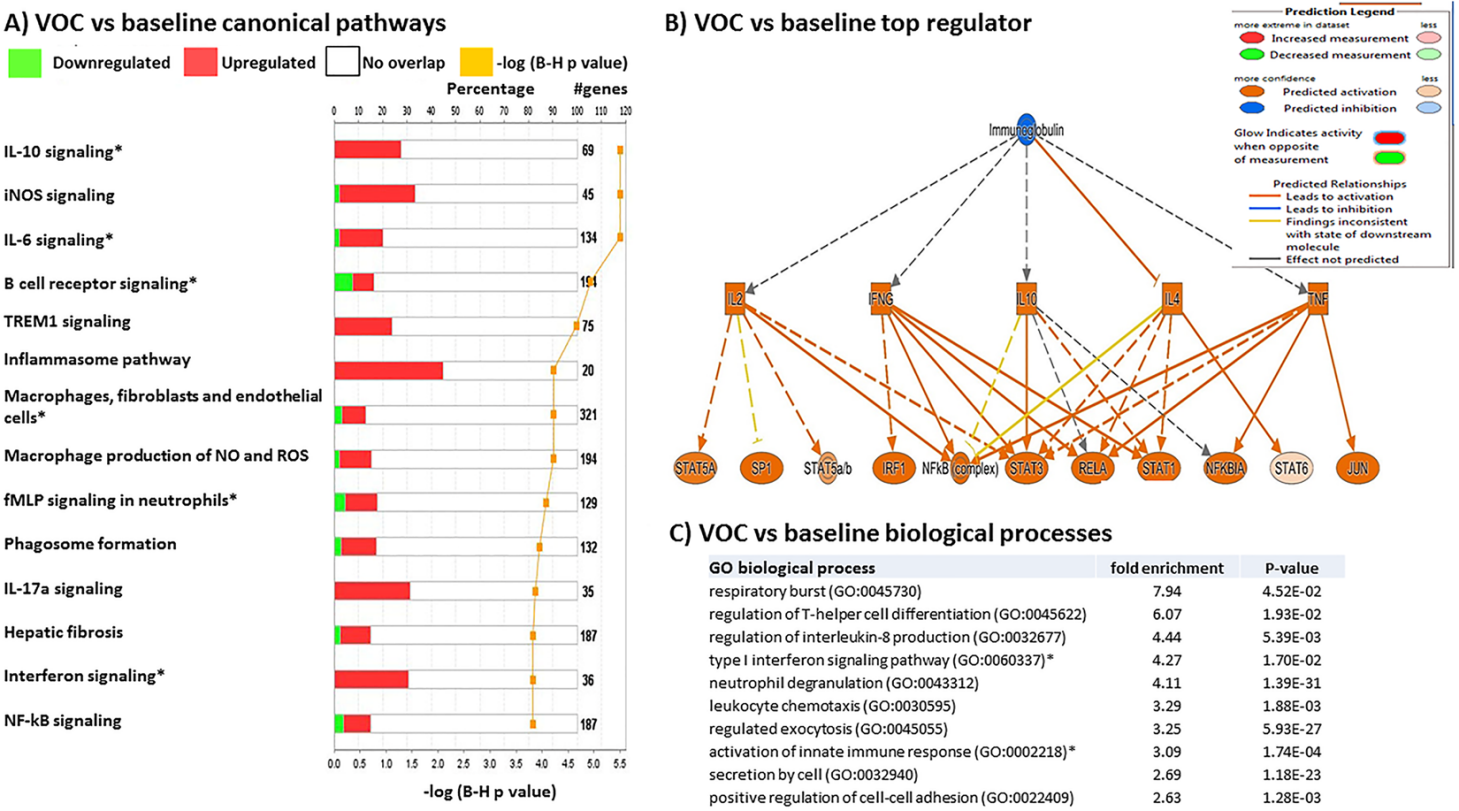
Unique pathways are regulated during VOC. (**A**) IPA analysis of canonical pathways altered during VOC compared to baseline. The top 14 differentially regulated canonical pathways are presented in descending order of statistical significance of –log p values. The % of genes within each pathway significantly altered is shown, with the exact # of genes altered presented at the end of each horizontal row. Upregulated genes are shown in red and downregulated in green. “*” indicates shared pathway with ACS events in Fig. 4. (**B**) Representative network analysis of the top predicted regulator of alterations in inflammatory signaling genes during VOC. A colored prediction legend is listed to describe predicted interactions. (**C**) Top biological processes determined by GO analysis for genes regulated during VOC. Biological processes are arranged in descending order of fold enrichment of each pathway. “*” indicates shared pathway with ACS events in Fig. 4.

Top canonical pathways in patients during VOC included IL-10 signaling, iNOS signaling, IL-6 signaling, and B cell receptor signaling (Fig. 5A). The top predicted upstream regulator was immunoglobulin, which regulates many downstream pro- and anti-inflammatory effects (Fig. 5B). Verification with GO analysis revealed many top pathways related to innate immune responses including the respiratory burst, T helper cell differentiation, and neutrophil degranulation (Fig. 5C). While pathway analysis revealed unique pathways in patients during VOC including iNOS and TREM1 signaling, several pathways were similar during ACS, including macrophage and interferon signaling (shared pathways denoted by stars in Figs. 4 and 5). Combined with our prior modular analyses, these data suggest upregulation of many pathways related to innate immunity occur during acute ACS and VOC events in children with SCD.

**Figure 6:**
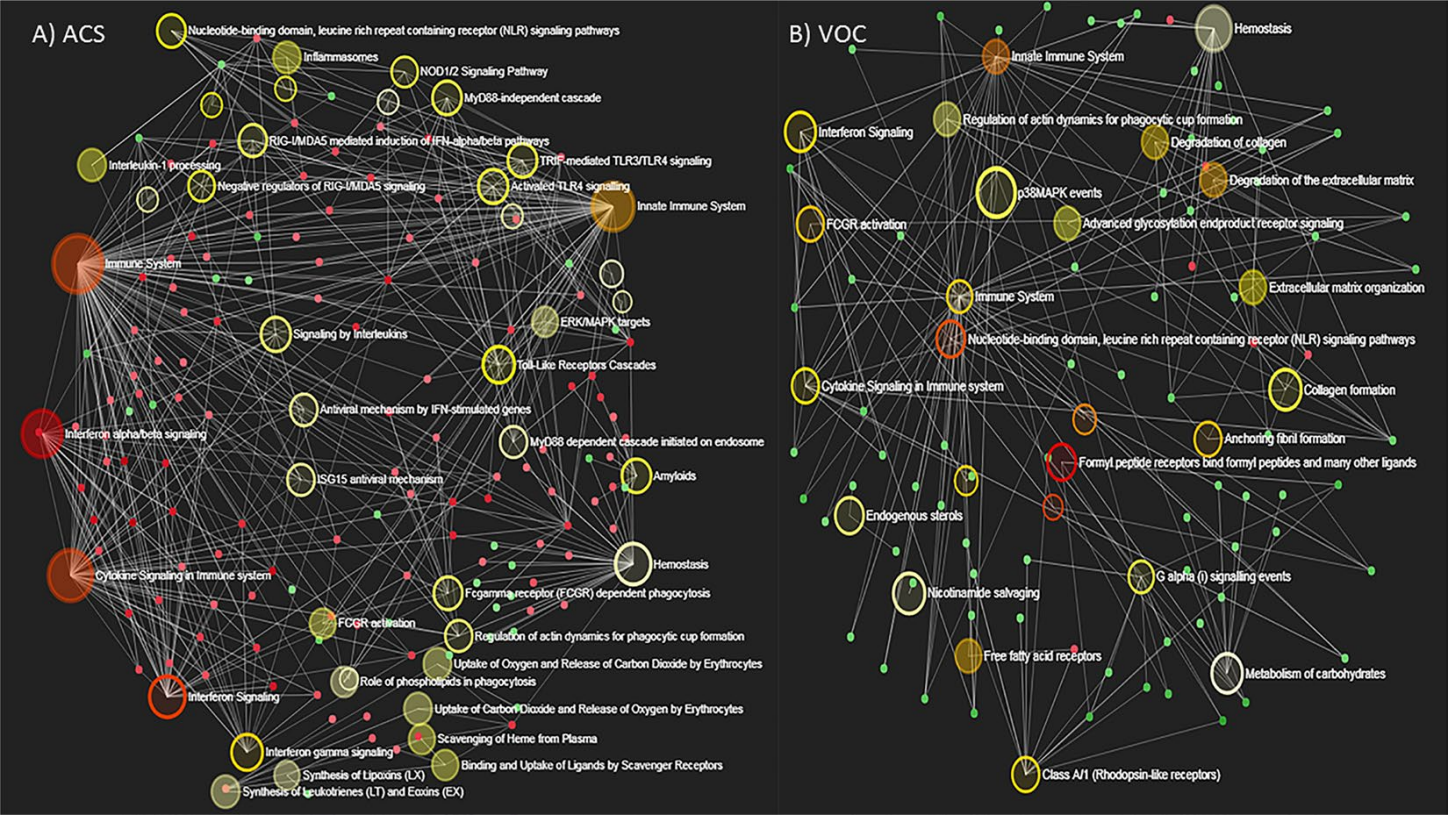
Nodal networks of gene interactions during ACS and VOC. Gene set enrichment analysis (GSEA) of SCD gene expression profiles during (**A**) ACS and (**B**) VOC. Nodes are colored according to their enrichment score (Red > orange> yellow> grey). The size of the node corresponds to the number of genes from that gene set that are on the analyzed gene list (larger nodes with more genes). The small filled nodes correspond to individual genes, and they are colored according to their fold change (red = overexpressed, green = under-expressed). Pathway names are listed next to the large nodes. White lines indicate connections between genes and pathways.

Last, we used a gene enrichment module to understand how the differentially regulated pathways were interacting during ACS and VOC. Patients with ACS had a complex nodal network related to inflammatory and infection response pathways including activation of NOD-like, Toll-like, and RIG-I like receptor signaling as specific pathogen recognition receptor pathways, and interaction with genes related to phagocytosis and phagosome formation (Fig. 6A). Other important nodal interactions included interferon signaling, inflammatory cytokine signaling, hemostasis, and amyloids (Fig. 6A). Patients during VOC displayed somewhat less complex nodal network interactions, with interactions between extracellular matrix and collagen organization/degradation along with decreased expression of genes controlling fatty acid receptor activation and p38MAPK (Fig. 6B).

## Discussion

While therapeutic advances such as gene therapy^17–19^ provide great hope for people with SCD, advances in the prevention and treatment of acute complications of SCD remain stalled. Further, many underdeveloped countries where SCD is most prevalent do not have access to advanced therapeutics or monitoring techniques. As such, ACS remains a leading contributor to morbidity and mortality in people with SCD across the globe, and better understanding ACS pathophysiology is needed to develop targeted ACS preventative and treatment modalities that may be more widely available to patients. To this end, we explored gene expression profile changes that occurred in a small sample of children with SCD during acute ACS and VOC events. We found both unique and shared changes in gene expression between the ACS and VOC groups. Our findings have implications for understanding ACS and VOC pathogenesis and the development of novel predictive, preventative, and therapeutic strategies.

Overall, we observed that patients differentially expressed 1800 and 3100 transcripts during VOC and ACS episodes respectively, suggesting that whole blood RNA-Seq was able to distinguish between changes in clinical status. Interestingly, among those participants that had an ACS episode, differential expression was also observed when classifying their gene profiles by gender. This may reflect that there could be hormonal signaling differences during ACS, but this would need verification in future, larger cohort studies designed to examine the effects of gender upon gene transcription. Previous transcriptomics studies in SCD utilized blood microarrays to generate profiles for SCD patients at baseline compared to controls^20^ and to risk-stratify the severity of disease^21^. However, RNA-Seq has only been employed in more limited studies of SCD^22^, and no prior studies have utilized changes in RNA-Seq during ACS or VOC episodes in children with SCD. Larger studies comparing clinical characteristics of illness severity with transcriptional profiles would also be useful in the future based on some overlap between baseline and VOC and ACS transcriptional profiles observed in our study. Our unique dataset is available to the SCD community to serve as a comparator for other transcriptomic studies in SCD, to generate novel hypotheses, and to spur the examination of new pathways for the treatment of acute complications. Whether changes in transcriptional profiles persist after ACS or VOC events or resolve with time is a future area of research.

We found that CD177 was over-expressed during both ACS and VOC, although over-expression was at a greater extent during ACS. CD177 is elevated in patients with polycythemia rubra vera and thalassemias^23,24^. One prior study demonstrated that increased expression of CD177 correlates to both erythropoietin-independent and erythropoietin-dependent increases in erythropoiesis in response to tissue hypoxia^25^. Thus, we postulate that increased CD177 expression may be a marker of increased tissue hypoxia found during both ACS and VOC episodes. CD177 could function as a useful biomarker to delineate changes from baseline clinical status in SCD, but would need further study to see if a threshold of expression can predict VOC or differentiate onset of ACS during a VOC episode.

In addition to over-expression of specific markers such as CD177, over-expression of inflammatory genes related to innate immune responses and concomitant suppression of genes related to adaptive immune responses (B and T cell signaling) occurred during both ACS and VOC episodes. These responses suggest a common altered immune response during acute illness in children with SCD. This result is not surprising based on known SCD risk factors, such as altered splenic and lymphocyte function^26^, and increased hemolysis of red blood cells (which promotes inflammatory byproducts). However, specific areas of innate and adaptive immune dysfunction are highlighted by our results. For example, platelet-inflammasome activation was recently found to promote vaso-occlusion in humanized SCD mice and human blood samples^27^. Our gene-enrichment analysis supports a role for inflammasomes during ACS, as we demonstrated that up-regulated inflammasome activation is linked to multiple aspects of inflammatory signaling during ACS. Furthermore, variants in toll-like receptor 2 (TLR2) were found to correlate with the occurrence of bacterial infection in a large SCD study^28^. However, our gene enrichment analysis suggests a role for multiple aspects of pathogen recognition receptors during ACS (e.g. TLR4, NOD1, etc.) and not just TLR2.

Another interesting aspect uncovered was how frequent viruses were isolated during ACS episodes and the upregulation of many aspects of interferon and RIG-I signaling during ACS. In a prior small cohort of people with SCD and non-SCD controls, blood neutrophils showed a high basal level expression of type-1 interferon signaling proteins when compared to controls^29^. In contrast, specific blood interferon alpha levels was equivalent to non-SCD controls in a larger cohort^30^. Our data indicate that these type-1 interferon responses are upregulated during ACS and to a lesser extent during VOC. It is unclear if over-expression of type-1 interferon signaling is an aberrant response to an already activated immune system in SCD, or an appropriate response to the pathogens encountered during ACS episodes. Upregulation during VOC would suggest that they are not just in response to viruses or other pathogens. Further research is needed to clarify the role of type 1 interferon signaling in SCD.

We found overexpression of S100A9 during VOC and ACS episodes. S100A9 expression has not been reported in prior SCD studies. Previous non-SCD studies demonstrated a correlation between serum levels of S100A9 proteins and inflammation. It is believed that S100A9 utilizes a calcium-binding mechanism to achieve effects, and increased serum S100A9 levels correlate with clinical severity in other diseases^31,32^. Since S100A9 transcripts were highly upregulated during VOC, this suggests increased pro-inflammatory calcium binding and decreased cytoplasmic calcium availability during VOC. Furthermore, when considering the vital role that calcium plays in cellular signaling, particularly in red blood cells in which cellular calcium volumes influence differentiation from precursors cells^33^, it is likely that decreasing the availability of free cationic calcium impacts the production and function of red blood cells in SCD. Thus, anti-S100A9 antibodies may have an interesting role as a therapeutic agent to inhibit inflammatory amplification by reducing cytokine release during VOC episodes.

Our study had several important limitations. First, our sample size was smaller than anticipated because participants who enrolled in this single-center study were hospitalized less frequently with ACS and with VOC than anticipated. This precluded us from validating our results using a second internal cohort. Also, our small sample size likely contributed to imbalances in the baseline characteristics between the ACS and VOC groups. However, we used baseline health samples as controls to compare to changes that occurred during hospitalization for VOC or ACS. Second, while whole blood transcriptomes are germane to study SCD pathogenesis that involves multiple cell types in circulation, blood profiles may limit generalization to airway-specific responses during ACS. Last, although those in the VOC group had clinical improvement without receiving ACS-specific treatment, it is also possible that some of these children had undiagnosed ACS as routine chest radiographs were not performed unless clinical signs and symptoms of ACS developed. However, this seems unlikely considering that seven of the 10 children with VOC had a negative chest radiograph during their admission.

In summary, results revealed a complex transcriptional network of changes in innate and adaptive immune gene expression in SCD children during both ACS and VOC. Together, these results provide unique insights into changes during acute events in children with SCD.

## Methods

### Study design and study participants

This was an institutional review board approved prospective, longitudinal, non-randomized, cohort study of patients with SCD at Nationwide Children’s Hospital. Informed consent and/or assent was obtained for all participants. For minors, a parent or guardian provided informed consent on their behalf. All methods were performed in accordance with the Declaration of Helsinki for medical research involving human subjects. Patients with any SCD genotype who were less than <19 years of age were eligible to participate if they did not have a congenital or acquired immunodeficiency besides functional or surgical asplenia, if they had not previously received a stem cell transplant or chronic blood transfusion therapy in the past 6 months, they had not had systemic corticosteroid or antibiotic use in the prior week (except for standard penicillin, amoxicillin or erythromycin encapsulated organism prophylaxis for functional or surgically asplenic patients), and had absence of current upper respiratory symptoms (including, increased cough, increased rhinorrhea, fever). Hydroxyurea use was allowed.

### Recruitment and study procedures

Eligible patients were sequentially approached to participate during their routine hematology clinic visits or during an acute ACS hospitalization from 2016–2018. Participants were then followed until the end of the study and had a second blood sample obtained within 24 hours of their next admission for either VOC or ACS. Baseline and hospitalizations study visits included a nasopharyngeal swab for viral testing, blood for whole blood RNA-sequencing, and a clinical history survey. At collection, one mL of whole blood was placed in a RNA stabilization tube and immediately frozen at −20 °C for later extraction. Participants’ demographic, hematologic, and clinical data at the time of each of their samples were abstracted from their electronic medical records and stored in a secured electronic REDCap database.

### ACS cases

ACS was defined by the sudden onset of signs and symptoms of lower respiratory tract disease (e.g., some combination of cough, shortness of breath, retractions, rales, hypoxia, etc.) and a new pulmonary infiltrate on chest radiograph. The definition was based on the National Heart, Lung, and Blood Institute’s SCD guidelines and was determined by the inpatient medical team independent of the research team. ACS cases were verified by a hematologist who primarily cares for patients with SCD, but who was not a member of the study team.

### VOC cases

VOC episodes were defined by acute pain in one or more body part without an alternative diagnostic explanation and that required hospitalization per the treating hematologist. If patients were diagnosed with ACS during their VOC admission, our protocol was to recollect these participants’ samples and reassign them to the ACS group.

### RNA-Seq library preparation and sequencing

RNA-Seq library preparation was performed per prior methods^12^. In brief, whole blood RNA quality was assessed using the Agilent 2100 Bioanalyzer and RNA Nano Chip kit (Agilent Technologies, CA). RNA-seq libraries were generated using TruSeq Stranded Total RNA with Ribo-Zero Globin Complete kit (Illumina, CA). Ribosomal RNA (rRNA) was removed from 1000 ng of total RNA with biotinylated, target-specific oligos combined with Ribo-Zero rRNA removal beads from the Human/Mouse/Rat Globin kit. Following rRNA removal, mRNA was fragmented using divalent cations under elevated temperature and converted into ds cDNA. Dual same indexed P5 and P7 adapters were ligated to ends of cDNA (Integrated DNA Technologies, Inc., Iowa). Following purification using the AMPure XP System (Beckman Coulter), the adaptor-ligated cDNA was amplified by limit-cycle PCR. Quality of libraries were determined via Agilent 4200 Tapestation using a High Sensitivity D1000 ScreenTape Assay kit, and quantified by KAPA qPCR (KAPA BioSystems). Approximately 60–80 million paired-end 150 bp reads were generated per sample using Illumina HiSeq. 4000 platform. Raw data was converted to FASTQ using v2.19.0.316 of Illumina’s bcl2fastq application. Sequencing adapters matching at least 6 bases were then removed from the reads, as well as low-quality bases (<10) using v1.10 of cutadapt (https://cutadapt.readthedocs.io/en/stable/index.html). An alignment report was also generated, using custom scripts, and manually reviewed to ensure that at least ~ 80% of reads aligned to the expected reference and that at least ~ 50% of the reads aligned to features annotated as protein coding.

### RNA-Seq data analysis

Each sample was aligned to the GRCh38.p9 assembly of the Homo Sapiens reference from the National Center for Biotechnology Information (NCBI) (http://www.ncbi.nlm.nih.gov/assembly/GCF_000001405.35/) using version 2.6.0c of the RNA-Seq aligner STAR (http://bioinformatics.oxfordjournals.org/content/early/2012/10/25/ bioinformatics.bts635). Features were identified from the general feature format (GFF) file that came with the assembly from NCBI. Feature coverage counts were calculated using HTSeq (https://htseq.readthedocs.io/en/master/count.html). Differentially expressed features were calculated using DESeq. 2 (http://genomebiology.com/2014/15/12/550). Genes were removed from comparisons if they were not expressed above a background threshold (0.5 reads per million) for the majority of samples within each group. Principal Components Analysis (PCA) was performed with standard methodology.

Gene transcription profiles were generated based on changes that occurred in the ACS group compared to baseline and for changes in the VOC group compared to baseline. Similarities and differences in gene transcription between the ACS and VOC groups were further characterized to determine how differentially regulated pathways may occur and/or interact during ACS and VOC episodes.

Functional gene analyses were performed using modular repertoires, IPA, GO, and NetworkAnalyst 3.0 gene enrichment module as described^16,34–37^. Modular analysis is a data driven approach based on clusters of coordinately expressed genes (modules) that share the same biological function^16^. We have previously validated this approach for whole-blood RNA-Seq^12^. The data is deposited in the NCBI Gene Expression Omnibus (GEO accession number: GSE139912).

### Gene validation by qRT-PCR

Three of the top overexpressed genes (CD177, S100A9, and THEM5) were selected for quantitative real-time PCR (qRT-PCR) validation. qRT-PCR was then performed using pre-designed TaqMan primer/probe combinations for CD177 (Hs00360669_m1, Thermo Fisher), S100A9 (Hs00610058_m1, Thermo Fisher), and THEM5 (Hs00699477_m1, Thermo Fisher). Whole blood RNA was extracted using the preserved RNA purification kit (Norgen Biotek, 43400) in accordance with the manufacturer’s instructions. The first strand of cDNA was synthesized from 1 µg total RNA using a high capacity cDNA reverse transcription kit (Thermo Fisher, 4368814) with 10X RT buffer, 25X dNTP mix (100 mM), 10X RT random primers, MultiScribe reverse transcriptase and RNase Inhibitor (Thermo Fisher, N8080119). Quantitative PCR (qPCR) mixes were assembled using Taqman Fast Master Mix (Thermo Fisher, 4444556) according to the manufacturer’s instructions. qPCR amplifications were performed in the 7500 Real Time PCR System (AB Applied Biosystems). The thermal cycler conditions were as follows: step 150 °C for 2:00 min, 95 °C for 10:00 min, 95 °C for 15 s, Step 2, 60 °C for 1:00 min (40 cycles) followed by a melting curve analysis. All reactions were performed with 10 biological replicates with reference dye normalization. The median cycle threshold (CT) value was used for analysis, and all CT values were normalized to expression of three housekeeping genes: 18 S ribosomal RNA (rRNA) (Thermo Fisher, Hs03928990_g1), GAPDH (Thermo Fisher, Hs03929097_g1) and HPRT1 (Thermo Fisher, Hs02800695_m1). GAPDH and HPRT1 were chosen for final normalized expression analysis based upon stability. Differences in mRNA expression between SCD groups and compared to controls were analyzed by pair-wise fixed reallocation randomization tests with REST 2009 software^38^.

### Virus detection

Nasopharyngeal swabs were collected and put into M4 viral transport media. The samples were immediately stored at −20 °C until tested. The BioFire FilmArray Respiratory Panel version 1.7 (BioFire/bioMeriux, Salt Lake City, UT) was used for viral testing on all samples per manufacturer’s instructions. This panel tests the most common 17 viruses isolated in North America.

### Statistical analysis

Demographic data was reported using means and the standard deviations. Participants’ hematologic data at baseline and during their ACS or VOC episode were compared using paired t-tests. For transcriptomic data, a list of significant differentially expressed features between the two groups was generated using an absolute value of log_2_ fold change ≥1.5 and an adjusted p-value of ≤ 0.05 [5% false discovery rate (FDR)]. Patient clustering for heatmaps was based upon Ward’s methods. For IPA, a 5% FDR was used for comparisons. For qRT-PCR analysis, statistical significance was determined using the integrated randomization and bootstrapping methods in the REST 2009 software package. Data analysis was performed in Stata/MP 13.1, R software, and GraphPad Prism 7.0, with a two-tail p < 0.05 considered statistically significant.

## Supplementary information

Supplementary Information.
