# Author Correction: Baseline and Disease-Induced Transcriptional Profiles in Children with Sickle Cell Disease

**DOI:** 10.1038/s41598-020-70341-2

**Published:** 2020-08-06

**Authors:** Susan Creary, Chandra L. Shrestha, Kavitha Kotha, Abena Minta, James Fitch, Lisa Jaramillo, Shuzhong Zhang, Swaroop Pinto, Rohan Thompson, Octavio Ramilo, Peter White, Asuncion Mejias, Benjamin T. Kopp

**Affiliations:** 1 Center for Innovation in Pediatric Practice, The Abigail Wexner Research Institute, Columbus, OH USA; 2 Division of Hematology and Oncology, Nationwide Children’s Hospital, Columbus, OH USA; 3 Center for Microbial Pathogenesis, The Abigail Wexner Research Institute, Columbus, OH USA; 4 Division of Pulmonary Medicine, Nationwide Children’s Hospital, Columbus, OH USA; 5 The Institute for Genomic Medicine, The Abigail Wexner Research Institute, Columbus, OH USA; 6 Center for Vaccines and Immunity, The Abigail Wexner Research Institute, Columbus, OH USA; 7 Division of Infectious Diseases, Nationwide Children’s Hospital, Columbus, OH USA

Correction to: *Scientific Reports*10.1038/s41598-020-65822-3, published online 02 June 2020

The original version of this Article contained a typographical error in the spelling of the author Asuncion Mejias which was incorrectly given as M. Asuncion Mejias.

As a result of this error, the Author Contribution statement was also incorrect in the original HTML and PDF versions of the Article.

S.C., K.K., M.A.M. and B.T.K. designed the study. S.C. and B.T.K. wrote the manuscript. J.F. and P.W. performed sequencing. C.L.S., J.F., L.J., P.W. and B.T.K. performed statistical analysis. S.C., C.L.S., K.K., A.M., J.F., S.Z., S.P., R.T., O.R., P.W., M.A.M. and B.T.K. analyzed and interpreted data. S.C., K.K. and B.T.K. collected clinical samples. All authors contributed to editing and approved the final manuscript.

now reads:

S.C., K.K., As.M. and B.T.K. designed the study. S.C. and B.T.K. wrote the manuscript. J.F. and P.W. performed sequencing. C.L.S., J.F., L.J., P.W. and B.T.K. performed statistical analysis. S.C., C.L.S., K.K., Ab.M., J.F., S.Z., S.P., R.T., O.R., P.W., As.M. and B.T.K. analyzed and interpreted data. S.C., K.K. and B.T.K. collected clinical samples. All authors contributed to editing and approved the final manuscript.

Additionally, the original version of the Supplementary Information file omitted Abena Minta and Lisa Jaramillo from the author list.

These errors have now been corrected in the PDF and HTML versions of the Article, and in the accompanying Supplementary Information file.

