# Supplementary material for: Baseline and Disease-Induced Transcriptional Profiles in Children with Sickle Cell Disease
Source: Sci Rep. 2020 Jun 2;10:9013. doi: 10.1038/s41598-020-65822-3 (PMC7265336; doi:10.1038/s41598-020-65822-3)
Supplement: Supplementary file 1 — Supplementary Information. [file 41598_2020_65822_MOESM1_ESM.docx]

**Supplemental Information**

**TRANSCRIPTIONAL RESPONSES IN CHILDREN WITH SICKLE CELL AT BASELINE AND DURING ACUTE VASO-OCCLUSIVE CRISIS OR ACUTE CHEST SYNDROME EPISODES**

Susan Creary^1,2^, Chandra L. Shrestha^3^, Kavitha Kotha^4^, James Fitch^5^, Shuzhong Zhang^3^, Swaroop Pinto^4^, Rohan Thompson^4^, Octavio Ramilo^6^, Peter White^5^, M. Asuncion Mejias^6^, and Benjamin T. Kopp^3,4^

Center for Innovation in Pediatric Practice^1^

Center for Microbial Pathogenesis^3^

The Institute for Genomic Medicine^5^

Center for Vaccines and Immunity^6^

The Abigail Wexner Research Institute

Columbus, OH USA

Division of Hematology and Oncology^2^

Division of Pulmonary Medicine^4^

Nationwide Children’s Hospital

Columbus, OH, USA

**
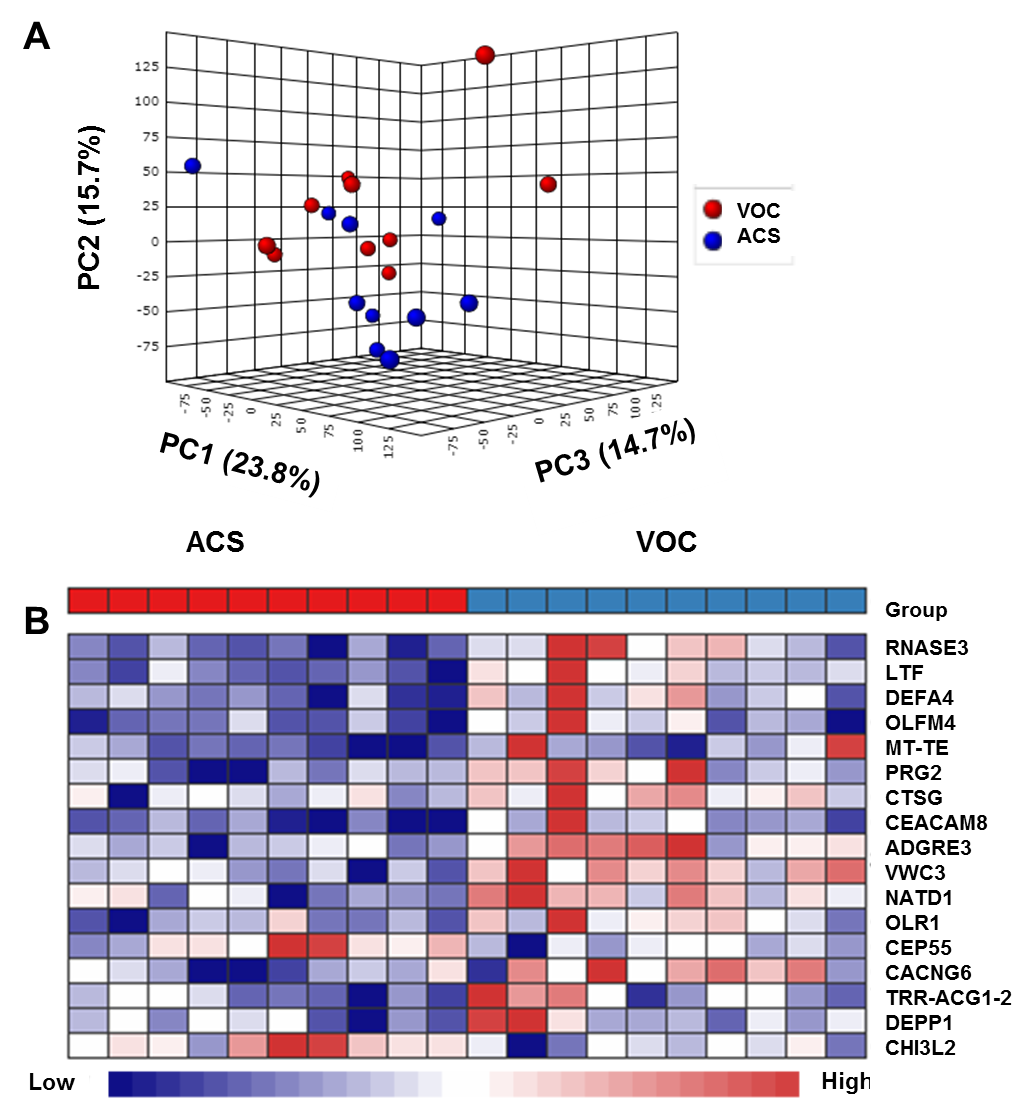
**

**Supplemental Figure S1:** A) Principal component analysis (PCA) of whole blood RNA-Seq profiles for SCD patients with ACS events compared to VOC events. Grouping are indicated by colors red (VOC) and blue (ACS. The % variance accounted for by the top 3 components are shown on the X, Y, and Z axes. B) Heat map of top differentially expressed genes between SCD patients with VOC compared to ACS. Each vertical row represents an individual patient with groupings of patients indicated by color at the top of the figures. At the bottom of the figure a legend represents the color continuum scheme used in the heat map (low to high gene expression). Group clustering determined by Ward’s methods.

**
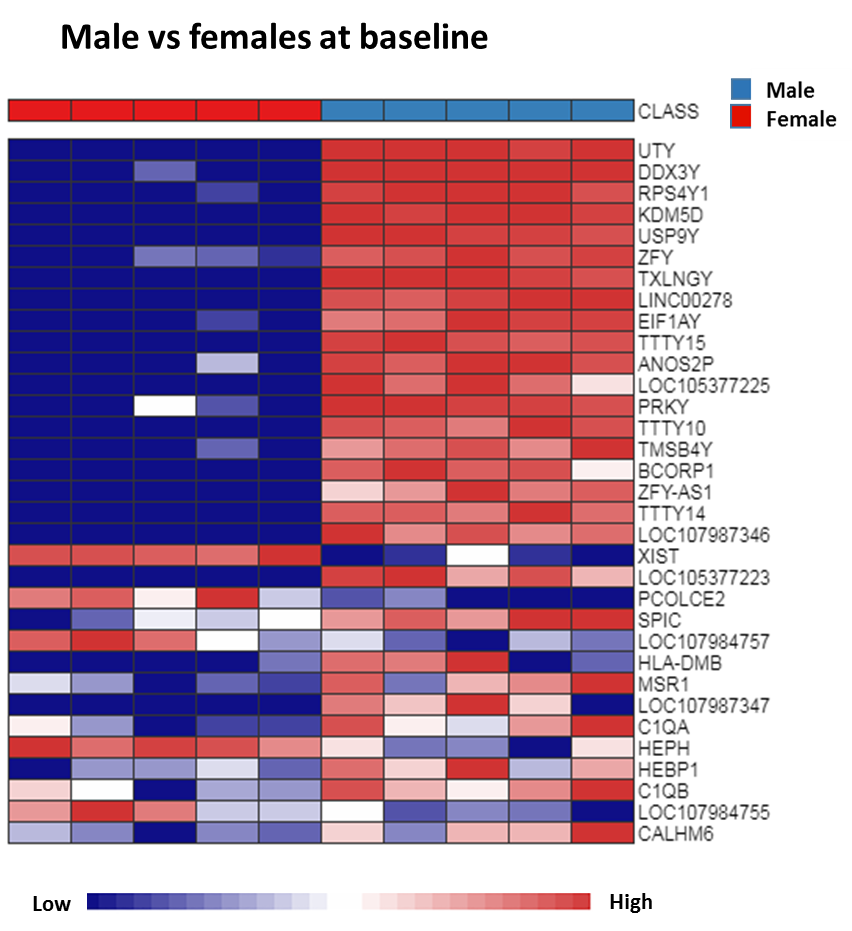
**

**Supplemental Figure S2: Differences in gene expression by gender.** Heat map of top differentially expressed genes at baseline for SCD patients among the ACS group by gender. The top 35 differentially expressed genes are annotated. Each vertical row represents an individual patient with groupings of patients indicated by color at the top of the figures (Class). At the bottom of the figure a legend represents the color continuum scheme used in the heat map (low to high gene expression). Group clustering determined by Ward’s methods.

**For the following tables, mean counts represent mean normalized read counts per gene (done by DeSeq2).**

**Supplementary Table S1: Summary of differentially expressed genes in SCD patients with ACS compared to baseline**

| **ID** | **Description** | **NCBIGeneID** | **meanFC** | **pAdj** | **Mean_ACS** | **Mean_Baseline** |
| --- | --- | --- | --- | --- | --- | --- |
| CD177 | CD177 molecule | [57126](https://www.ncbi.nlm.nih.gov/gene/?term=57126) | 166.5 | 0.00000 | 9964.5 | 52.6 |
| SLC51A | solute carrier family 51 alpha subunit | [200931](https://www.ncbi.nlm.nih.gov/gene/?term=200931) | 67.5 | 0.00001 | 14.6 | 0.1 |
| LINC01093 | long intergenic non-protein coding RNA 1093 | [100506229](https://www.ncbi.nlm.nih.gov/gene/?term=100506229) | 45.2 | 0.00000 | 192.6 | 3.7 |
| LOC105374545 |  | [105374545](https://www.ncbi.nlm.nih.gov/gene/?term=105374545) | 30.2 | 0.00000 | 27.3 | 1.0 |
| LOC105369402 |  | [105369402](https://www.ncbi.nlm.nih.gov/gene/?term=105369402) | 28.8 | 0.00000 | 193.4 | 5.8 |
| LOC107985357 |  | [107985357](https://www.ncbi.nlm.nih.gov/gene/?term=107985357) | -16.5 | 0.00000 | 0.8 | 19.5 |
| LOC105373165 |  | [105373165](https://www.ncbi.nlm.nih.gov/gene/?term=105373165) | -16.5 | 0.00001 | 0.4 | 9.0 |
| LOC105372578 |  | [105372578](https://www.ncbi.nlm.nih.gov/gene/?term=105372578) | 15.2 | 0.00000 | 852.8 | 47.2 |
| OTOF | otoferlin | [9381](https://www.ncbi.nlm.nih.gov/gene/?term=9381) | 14.5 | 0.00007 | 423.5 | 21.7 |
| LOC107986198 |  | [107986198](https://www.ncbi.nlm.nih.gov/gene/?term=107986198) | 14.0 | 0.00020 | 7.2 | 0.3 |
| LOC107985374 |  | [107985374](https://www.ncbi.nlm.nih.gov/gene/?term=107985374) | 14.0 | 0.00037 | 13.6 | 0.6 |
| LOC105375769 |  | [105375769](https://www.ncbi.nlm.nih.gov/gene/?term=105375769) | 11.4 | 0.00000 | 83.7 | 6.3 |
| FAM20A | family with sequence similarity 20 member A | [54757](https://www.ncbi.nlm.nih.gov/gene/?term=54757) | 11.2 | 0.00000 | 150.7 | 12.0 |
| LOC105376505 |  | [105376505](https://www.ncbi.nlm.nih.gov/gene/?term=105376505) | 11.1 | 0.00000 | 449.7 | 36.4 |
| LOC105376504 |  | [105376504](https://www.ncbi.nlm.nih.gov/gene/?term=105376504) | 10.6 | 0.00000 | 164.9 | 13.7 |
| FCGR1CP | Fc fragment of IgG receptor Ic, pseudogene | [100132417](https://www.ncbi.nlm.nih.gov/gene/?term=100132417) | 10.2 | 0.00000 | 1338.0 | 117.8 |
| GPR84 | G protein-coupled receptor 84 | [53831](https://www.ncbi.nlm.nih.gov/gene/?term=53831) | 10.2 | 0.00000 | 137.9 | 12.2 |
| CASP5 | caspase 5 | [838](https://www.ncbi.nlm.nih.gov/gene/?term=838) | 10.2 | 0.00000 | 1767.9 | 161.0 |
| MIR3945HG | MIR3945 host gene | [731424](https://www.ncbi.nlm.nih.gov/gene/?term=731424) | 10.1 | 0.00000 | 1282.8 | 115.2 |
| SOCS3 | suppressor of cytokine signaling 3 | [9021](https://www.ncbi.nlm.nih.gov/gene/?term=9021) | 9.7 | 0.00000 | 5163.4 | 488.9 |
| TNFAIP8L3 | TNF alpha induced protein 8 like 3 | [388121](https://www.ncbi.nlm.nih.gov/gene/?term=388121) | 9.6 | 0.00158 | 11.8 | 0.8 |
| GSDMC | gasdermin C | [56169](https://www.ncbi.nlm.nih.gov/gene/?term=56169) | 9.2 | 0.00006 | 23.9 | 2.0 |
| FFAR3 | free fatty acid receptor 3 | [2865](https://www.ncbi.nlm.nih.gov/gene/?term=2865) | 8.7 | 0.00000 | 141.5 | 14.6 |
| ALMS1P1 | ALMS1, centrosome and basal body associated protein pseudogene 1 | [200420](https://www.ncbi.nlm.nih.gov/gene/?term=200420) | 8.5 | 0.00008 | 114.8 | 10.6 |
| LOC107985550 |  | [107985550](https://www.ncbi.nlm.nih.gov/gene/?term=107985550) | 8.5 | 0.00001 | 19.9 | 1.9 |
| LOC105369467 |  | [105369467](https://www.ncbi.nlm.nih.gov/gene/?term=105369467) | 8.1 | 0.00064 | 47.9 | 4.2 |
| LOC107984113 |  | [107984113](https://www.ncbi.nlm.nih.gov/gene/?term=107984113) | -7.8 | 0.00072 | 1.0 | 11.9 |
| CARD17 | caspase recruitment domain family member 17 | [440068](https://www.ncbi.nlm.nih.gov/gene/?term=440068) | 7.8 | 0.00000 | 440.9 | 50.2 |
| ANXA3 | annexin A3 | [306](https://www.ncbi.nlm.nih.gov/gene/?term=306) | 7.5 | 0.00000 | 6001.9 | 723.3 |
| LOC101928674 | uncharacterized LOC101928674 | [101928674](https://www.ncbi.nlm.nih.gov/gene/?term=101928674) | 7.5 | 0.00001 | 18.5 | 2.2 |
| MCEMP1 | mast cell-expressed membrane protein 1 | [199675](https://www.ncbi.nlm.nih.gov/gene/?term=199675) | 7.5 | 0.00000 | 379.8 | 45.7 |
| SLC1A3 | solute carrier family 1 member 3 | [6507](https://www.ncbi.nlm.nih.gov/gene/?term=6507) | 7.4 | 0.00000 | 133.0 | 15.7 |
| CD274 | CD274 molecule | [29126](https://www.ncbi.nlm.nih.gov/gene/?term=29126) | 7.1 | 0.00000 | 3745.2 | 458.9 |
| FCGR1A | Fc fragment of IgG receptor Ia | [2209](https://www.ncbi.nlm.nih.gov/gene/?term=2209) | 7.0 | 0.00000 | 5469.0 | 701.2 |
| IL27 | interleukin 27 | [246778](https://www.ncbi.nlm.nih.gov/gene/?term=246778) | 6.9 | 0.00000 | 27.5 | 3.5 |
| GBP1P1 | guanylate binding protein 1 pseudogene 1 | [400759](https://www.ncbi.nlm.nih.gov/gene/?term=400759) | 6.8 | 0.00024 | 255.2 | 28.9 |
| UNQ6494 | uncharacterized LOC100129066 | [100129066](https://www.ncbi.nlm.nih.gov/gene/?term=100129066) | 6.8 | 0.00008 | 9.1 | 1.2 |
| LOC107985562 |  | [107985562](https://www.ncbi.nlm.nih.gov/gene/?term=107985562) | 6.8 | 0.00536 | 9.9 | 0.8 |
| PCOLCE2 | procollagen C-endopeptidase enhancer 2 | [26577](https://www.ncbi.nlm.nih.gov/gene/?term=26577) | 6.4 | 0.00462 | 23.1 | 2.1 |
| LOC107986751 |  | [107986751](https://www.ncbi.nlm.nih.gov/gene/?term=107986751) | 6.3 | 0.00000 | 25.3 | 3.8 |
| CCNA1 | cyclin A1 | [8900](https://www.ncbi.nlm.nih.gov/gene/?term=8900) | 6.2 | 0.00003 | 94.4 | 12.5 |
| VWCE | von Willebrand factor C and EGF domains | [220001](https://www.ncbi.nlm.nih.gov/gene/?term=220001) | -6.2 | 0.00000 | 5.5 | 37.9 |
| ST3GAL4-AS1 | ST3GAL4 antisense RNA 1 (head to head) | [399972](https://www.ncbi.nlm.nih.gov/gene/?term=399972) | 6.2 | 0.00000 | 423.7 | 63.4 |
| LOC100422497 | UTP18, small subunit (SSU) processome component, homolog (yeast) pseudogene | [100422497](https://www.ncbi.nlm.nih.gov/gene/?term=100422497) | 6.2 | 0.00002 | 12.6 | 1.7 |
| LOC105377156 |  | [105377156](https://www.ncbi.nlm.nih.gov/gene/?term=105377156) | 6.1 | 0.00000 | 104.8 | 15.7 |
| MYO3A | myosin IIIA | [53904](https://www.ncbi.nlm.nih.gov/gene/?term=53904) | -6.0 | 0.00010 | 1.8 | 12.3 |
| EXOC3L1 | exocyst complex component 3 like 1 | [283849](https://www.ncbi.nlm.nih.gov/gene/?term=283849) | 6.0 | 0.00000 | 161.6 | 23.5 |
| FCGR1B | Fc fragment of IgG receptor Ib | [2210](https://www.ncbi.nlm.nih.gov/gene/?term=2210) | 5.9 | 0.00000 | 1621.5 | 249.1 |
| LOC102723914 |  | [102723914](https://www.ncbi.nlm.nih.gov/gene/?term=102723914) | -5.9 | 0.00005 | 3.5 | 24.4 |
| ANKRD22 | ankyrin repeat domain 22 | [118932](https://www.ncbi.nlm.nih.gov/gene/?term=118932) | 5.9 | 0.00002 | 856.9 | 123.6 |
| CCL2 | C-C motif chemokine ligand 2 | [6347](https://www.ncbi.nlm.nih.gov/gene/?term=6347) | 5.9 | 0.00185 | 62.6 | 7.3 |
| USP18 | ubiquitin specific peptidase 18 | [11274](https://www.ncbi.nlm.nih.gov/gene/?term=11274) | 5.7 | 0.00003 | 1116.1 | 163.7 |
| HESX1 | HESX homeobox 1 | [8820](https://www.ncbi.nlm.nih.gov/gene/?term=8820) | 5.6 | 0.00003 | 67.6 | 9.9 |
| HBG1 | hemoglobin subunit gamma 1 | [3047](https://www.ncbi.nlm.nih.gov/gene/?term=3047) | -5.6 | 0.00147 | 26.8 | 213.0 |
| SLC38A11 | solute carrier family 38 member 11 | [151258](https://www.ncbi.nlm.nih.gov/gene/?term=151258) | -5.6 | 0.00002 | 4.8 | 33.2 |
| XCR1 | X-C motif chemokine receptor 1 | [2829](https://www.ncbi.nlm.nih.gov/gene/?term=2829) | 5.5 | 0.00008 | 62.7 | 9.6 |
| PDCD1LG2 | programmed cell death 1 ligand 2 | [80380](https://www.ncbi.nlm.nih.gov/gene/?term=80380) | 5.4 | 0.00001 | 149.4 | 24.3 |
| LOC105371912 |  | [105371912](https://www.ncbi.nlm.nih.gov/gene/?term=105371912) | 5.4 | 0.00000 | 59.9 | 10.1 |
| CXCL10 | C-X-C motif chemokine ligand 10 | [3627](https://www.ncbi.nlm.nih.gov/gene/?term=3627) | 5.3 | 0.00011 | 430.9 | 67.1 |
| ADM | adrenomedullin | [133](https://www.ncbi.nlm.nih.gov/gene/?term=133) | 5.3 | 0.00000 | 3892.8 | 687.9 |
| S100A12 | S100 calcium binding protein A12 | [6283](https://www.ncbi.nlm.nih.gov/gene/?term=6283) | 5.2 | 0.00000 | 10325.6 | 1793.3 |
| LOC105369192 | uncharacterized LOC105369192 | [105369192](https://www.ncbi.nlm.nih.gov/gene/?term=105369192) | 5.2 | 0.00086 | 26.4 | 4.0 |
| IRF7 | interferon regulatory factor 7 | [3665](https://www.ncbi.nlm.nih.gov/gene/?term=3665) | 5.1 | 0.00000 | 352.6 | 63.1 |
| COL23A1 | collagen type XXIII alpha 1 | [91522](https://www.ncbi.nlm.nih.gov/gene/?term=91522) | 5.1 | 0.00001 | 16.6 | 2.9 |
| MS4A4A | membrane spanning 4-domains A4A | [51338](https://www.ncbi.nlm.nih.gov/gene/?term=51338) | 5.1 | 0.00000 | 464.1 | 83.2 |
| LOC105377067 |  | [105377067](https://www.ncbi.nlm.nih.gov/gene/?term=105377067) | 5.1 | 0.00000 | 670.5 | 118.7 |
| LOC101929544 | uncharacterized LOC101929544 | [101929544](https://www.ncbi.nlm.nih.gov/gene/?term=101929544) | 5.0 | 0.00001 | 37.9 | 6.9 |
| DEPDC1P2 | DEP domain containing 1 pseudogene 2 | [100126446](https://www.ncbi.nlm.nih.gov/gene/?term=100126446) | -5.0 | 0.00002 | 8.3 | 49.3 |
| LOC107984519 |  | [107984519](https://www.ncbi.nlm.nih.gov/gene/?term=107984519) | -5.0 | 0.00008 | 3.1 | 18.6 |
| LOC102724861 |  | [102724861](https://www.ncbi.nlm.nih.gov/gene/?term=102724861) | -5.0 | 0.00596 | 4.5 | 37.5 |
| C1QB | complement component 1, q subcomponent, B chain | [713](https://www.ncbi.nlm.nih.gov/gene/?term=713) | 5.0 | 0.00001 | 260.7 | 45.9 |
| TRHDE-AS1 | TRHDE antisense RNA 1 | [283392](https://www.ncbi.nlm.nih.gov/gene/?term=283392) | -5.0 | 0.00621 | 3.9 | 32.3 |
| ISG15 | ISG15 ubiquitin-like modifier | [9636](https://www.ncbi.nlm.nih.gov/gene/?term=9636) | 5.0 | 0.00014 | 17127.3 | 2860.4 |
| IL10 | interleukin 10 | [3586](https://www.ncbi.nlm.nih.gov/gene/?term=3586) | 4.9 | 0.00000 | 39.3 | 7.1 |
| PPARG | peroxisome proliferator activated receptor gamma | [5468](https://www.ncbi.nlm.nih.gov/gene/?term=5468) | 4.9 | 0.00001 | 42.0 | 7.7 |
| ITGA7 | integrin subunit alpha 7 | [3679](https://www.ncbi.nlm.nih.gov/gene/?term=3679) | 4.8 | 0.00000 | 63.8 | 11.7 |
| LOC105377587 |  | [105377587](https://www.ncbi.nlm.nih.gov/gene/?term=105377587) | 4.8 | 0.00012 | 11.3 | 2.0 |
| IFIT1 | interferon induced protein with tetratricopeptide repeats 1 | [3434](https://www.ncbi.nlm.nih.gov/gene/?term=3434) | 4.8 | 0.00017 | 35256.4 | 6050.3 |
| FGF13 | fibroblast growth factor 13 | [2258](https://www.ncbi.nlm.nih.gov/gene/?term=2258) | 4.8 | 0.00000 | 69.1 | 12.9 |
| LOC105372652 |  | [105372652](https://www.ncbi.nlm.nih.gov/gene/?term=105372652) | 4.8 | 0.00008 | 17.7 | 3.2 |
| RUFY4 | RUN and FYVE domain containing 4 | [285180](https://www.ncbi.nlm.nih.gov/gene/?term=285180) | 4.8 | 0.00002 | 84.6 | 15.2 |
| MICALCL | MICAL C-terminal like | [84953](https://www.ncbi.nlm.nih.gov/gene/?term=84953) | -4.8 | 0.00000 | 290.7 | 1579.4 |
| EDNRB | endothelin receptor type B | [1910](https://www.ncbi.nlm.nih.gov/gene/?term=1910) | 4.7 | 0.00059 | 25.2 | 4.1 |
| LOC105374304 |  | [105374304](https://www.ncbi.nlm.nih.gov/gene/?term=105374304) | 4.7 | 0.00202 | 31.9 | 5.0 |
| MYO10 | myosin X | [4651](https://www.ncbi.nlm.nih.gov/gene/?term=4651) | 4.7 | 0.00000 | 109.5 | 21.1 |
| HK3 | hexokinase 3 | [3101](https://www.ncbi.nlm.nih.gov/gene/?term=3101) | 4.7 | 0.00000 | 10339.0 | 2071.8 |
| TRIM51BP | tripartite motif-containing 51B, pseudogene | [399937](https://www.ncbi.nlm.nih.gov/gene/?term=399937) | -4.7 | 0.00096 | 1.1 | 6.8 |
| OAS1 | 2'-5'-oligoadenylate synthetase 1 | [4938](https://www.ncbi.nlm.nih.gov/gene/?term=4938) | 4.7 | 0.00001 | 15524.8 | 2915.0 |
| LOC105373033 |  | [105373033](https://www.ncbi.nlm.nih.gov/gene/?term=105373033) | 4.6 | 0.00089 | 29.6 | 4.9 |
| IFI6 | interferon alpha inducible protein 6 | [2537](https://www.ncbi.nlm.nih.gov/gene/?term=2537) | 4.6 | 0.00001 | 11135.5 | 2110.2 |
| HMGB3P9 | high mobility group box 3 pseudogene 9 | [100873879](https://www.ncbi.nlm.nih.gov/gene/?term=100873879) | -4.6 | 0.00423 | 1.1 | 8.4 |
| SLC26A8 | solute carrier family 26 member 8 | [116369](https://www.ncbi.nlm.nih.gov/gene/?term=116369) | 4.6 | 0.00000 | 429.3 | 86.0 |
| OASL | 2'-5'-oligoadenylate synthetase like | [8638](https://www.ncbi.nlm.nih.gov/gene/?term=8638) | 4.6 | 0.00000 | 5781.7 | 1120.6 |
| VNN1 | vanin 1 | [8876](https://www.ncbi.nlm.nih.gov/gene/?term=8876) | 4.6 | 0.00000 | 4512.7 | 905.3 |
| GATSL2 | GATS protein-like 2 | [729438](https://www.ncbi.nlm.nih.gov/gene/?term=729438) | -4.6 | 0.00000 | 146.1 | 750.1 |
| LOC285696 | uncharacterized LOC285696 | [285696](https://www.ncbi.nlm.nih.gov/gene/?term=285696) | 4.6 | 0.00001 | 66.8 | 13.2 |
| HIGD1AP16 | HIG1 hypoxia inducible domain family member 1A pseudogene 16 | [100874476](https://www.ncbi.nlm.nih.gov/gene/?term=100874476) | -4.5 | 0.00001 | 11.9 | 62.3 |
| LOC105371082 |  | [105371082](https://www.ncbi.nlm.nih.gov/gene/?term=105371082) | 4.5 | 0.00029 | 59.1 | 10.7 |
| C1QA | complement component 1, q subcomponent, A chain | [712](https://www.ncbi.nlm.nih.gov/gene/?term=712) | 4.5 | 0.00000 | 380.5 | 75.7 |
| LOC105372801 |  | [105372801](https://www.ncbi.nlm.nih.gov/gene/?term=105372801) | 4.5 | 0.00001 | 75.0 | 15.2 |
| IFIT3 | interferon induced protein with tetratricopeptide repeats 3 | [3437](https://www.ncbi.nlm.nih.gov/gene/?term=3437) | 4.5 | 0.00005 | 44237.9 | 8571.2 |
| NYAP2 | neuronal tyrosine-phosphorylated phosphoinositide-3-kinase adaptor 2 | [57624](https://www.ncbi.nlm.nih.gov/gene/?term=57624) | -4.4 | 0.01335 | 0.6 | 5.4 |
| ST13P7 | suppression of tumorigenicity 13 (colon carcinoma) (Hsp70 interacting protein) pseudogene 7 | [155019](https://www.ncbi.nlm.nih.gov/gene/?term=155019) | -4.4 | 0.00003 | 2.1 | 10.5 |
| AIM2 | absent in melanoma 2 | [9447](https://www.ncbi.nlm.nih.gov/gene/?term=9447) | 4.4 | 0.00000 | 1663.5 | 349.2 |
| LOC107984315 |  | [107984315](https://www.ncbi.nlm.nih.gov/gene/?term=107984315) | -4.4 | 0.00001 | 105.7 | 527.0 |
| LOC105373715 |  | [105373715](https://www.ncbi.nlm.nih.gov/gene/?term=105373715) | -4.4 | 0.00000 | 4.9 | 24.5 |
| TNFAIP6 | TNF alpha induced protein 6 | [7130](https://www.ncbi.nlm.nih.gov/gene/?term=7130) | 4.4 | 0.00000 | 2113.3 | 436.5 |
| LOC105378041 |  | [105378041](https://www.ncbi.nlm.nih.gov/gene/?term=105378041) | 4.3 | 0.00105 | 15.0 | 2.6 |
| PKD2L1 | polycystin 2 like 1, transient receptor potential cation channel | [9033](https://www.ncbi.nlm.nih.gov/gene/?term=9033) | 4.3 | 0.00179 | 19.7 | 3.3 |
| CDKN1C | cyclin-dependent kinase inhibitor 1C | [1028](https://www.ncbi.nlm.nih.gov/gene/?term=1028) | 4.3 | 0.00180 | 10.7 | 1.8 |
| LOC344887 | NmrA-like family domain containing 1 pseudogene | [344887](https://www.ncbi.nlm.nih.gov/gene/?term=344887) | 4.3 | 0.00058 | 13.7 | 2.7 |
| PLSCR1 | phospholipid scramblase 1 | [5359](https://www.ncbi.nlm.nih.gov/gene/?term=5359) | 4.3 | 0.00000 | 7379.7 | 1574.0 |
| OPLAH | 5-oxoprolinase (ATP-hydrolysing) | [26873](https://www.ncbi.nlm.nih.gov/gene/?term=26873) | 4.3 | 0.00000 | 337.0 | 71.5 |
| LOC105372633 |  | [105372633](https://www.ncbi.nlm.nih.gov/gene/?term=105372633) | 4.3 | 0.00268 | 10.5 | 1.8 |
| TRIM6 | tripartite motif containing 6 | [117854](https://www.ncbi.nlm.nih.gov/gene/?term=117854) | 4.3 | 0.00002 | 82.8 | 16.8 |
| ICAM5 | intercellular adhesion molecule 5 | [7087](https://www.ncbi.nlm.nih.gov/gene/?term=7087) | 4.3 | 0.00000 | 56.2 | 12.2 |
| PROK2 | prokineticin 2 | [60675](https://www.ncbi.nlm.nih.gov/gene/?term=60675) | 4.3 | 0.00000 | 6708.4 | 1429.6 |
| HERC5 | HECT and RLD domain containing E3 ubiquitin protein ligase 5 | [51191](https://www.ncbi.nlm.nih.gov/gene/?term=51191) | 4.3 | 0.00012 | 12418.6 | 2468.0 |
| METTL7B | methyltransferase like 7B | [196410](https://www.ncbi.nlm.nih.gov/gene/?term=196410) | 4.2 | 0.00620 | 15.0 | 2.2 |
| NRN1 | neuritin 1 | [51299](https://www.ncbi.nlm.nih.gov/gene/?term=51299) | 4.2 | 0.00024 | 99.0 | 19.1 |
| DHX58 | DEXH-box helicase 58 | [79132](https://www.ncbi.nlm.nih.gov/gene/?term=79132) | 4.2 | 0.00000 | 1755.3 | 384.4 |
| LOC101927780 | uncharacterized LOC101927780 | [101927780](https://www.ncbi.nlm.nih.gov/gene/?term=101927780) | 4.2 | 0.00004 | 87.8 | 18.2 |
| MYBPH | myosin binding protein H | [4608](https://www.ncbi.nlm.nih.gov/gene/?term=4608) | -4.2 | 0.00448 | 0.6 | 4.2 |
| HES4 | hes family bHLH transcription factor 4 | [57801](https://www.ncbi.nlm.nih.gov/gene/?term=57801) | 4.2 | 0.00011 | 353.8 | 71.4 |
| OSM | oncostatin M | [5008](https://www.ncbi.nlm.nih.gov/gene/?term=5008) | 4.2 | 0.00000 | 266.3 | 57.9 |
| IL34 | interleukin 34 | [146433](https://www.ncbi.nlm.nih.gov/gene/?term=146433) | -4.2 | 0.00262 | 1.2 | 6.6 |
| IFIT2 | interferon induced protein with tetratricopeptide repeats 2 | [3433](https://www.ncbi.nlm.nih.gov/gene/?term=3433) | 4.2 | 0.00001 | 66709.1 | 14054.9 |
| FBXO6 | F-box protein 6 | [26270](https://www.ncbi.nlm.nih.gov/gene/?term=26270) | 4.2 | 0.00000 | 1180.6 | 265.2 |
| HP | haptoglobin | [3240](https://www.ncbi.nlm.nih.gov/gene/?term=3240) | 4.1 | 0.00091 | 295.7 | 56.1 |
| RPS29P12 | ribosomal protein S29 pseudogene 12 | [100131173](https://www.ncbi.nlm.nih.gov/gene/?term=100131173) | -4.1 | 0.00069 | 4.1 | 22.4 |
| OCLM | oculomedin | [10896](https://www.ncbi.nlm.nih.gov/gene/?term=10896) | -4.1 | 0.00483 | 1.4 | 8.9 |
| LOC100996274 |  | [100996274](https://www.ncbi.nlm.nih.gov/gene/?term=100996274) | -4.1 | 0.00014 | 8.5 | 42.8 |
| LOC105375500 |  | [105375500](https://www.ncbi.nlm.nih.gov/gene/?term=105375500) | -4.1 | 0.00108 | 3.4 | 18.9 |
| DUSP13 | dual specificity phosphatase 13 | [51207](https://www.ncbi.nlm.nih.gov/gene/?term=51207) | 4.1 | 0.00122 | 20.4 | 4.1 |
| MIR744 | microRNA 744 | [100126313](https://www.ncbi.nlm.nih.gov/gene/?term=100126313) | -4.1 | 0.00000 | 4.4 | 20.1 |
| NATD1 | N-acetyltransferase domain containing 1 | [256302](https://www.ncbi.nlm.nih.gov/gene/?term=256302) | -4.0 | 0.00000 | 969.1 | 4270.2 |
| FAM90A1 | family with sequence similarity 90 member A1 | [55138](https://www.ncbi.nlm.nih.gov/gene/?term=55138) | 4.0 | 0.00670 | 6.8 | 1.1 |
| ODF3B | outer dense fiber of sperm tails 3B | [440836](https://www.ncbi.nlm.nih.gov/gene/?term=440836) | 4.0 | 0.00000 | 1019.4 | 238.5 |
| PARP10 | poly(ADP-ribose) polymerase family member 10 | [84875](https://www.ncbi.nlm.nih.gov/gene/?term=84875) | 4.0 | 0.00000 | 4867.8 | 1142.7 |
| PHF24 | PHD finger protein 24 | [23349](https://www.ncbi.nlm.nih.gov/gene/?term=23349) | -4.0 | 0.00446 | 2.7 | 14.8 |
| C5orf67 | chromosome 5 open reading frame 67 | [101928448](https://www.ncbi.nlm.nih.gov/gene/?term=101928448) | 4.0 | 0.00001 | 62.0 | 14.1 |
| KIAA0430 | KIAA0430 | [9665](https://www.ncbi.nlm.nih.gov/gene/?term=9665) | -4.0 | 0.00000 | 38.2 | 159.2 |
| IFI35 | interferon induced protein 35 | [3430](https://www.ncbi.nlm.nih.gov/gene/?term=3430) | 4.0 | 0.00000 | 2763.4 | 645.5 |
| RTP4 | receptor (chemosensory) transporter protein 4 | [64108](https://www.ncbi.nlm.nih.gov/gene/?term=64108) | 4.0 | 0.00001 | 1369.7 | 309.8 |
| PSMF1 | proteasome inhibitor subunit 1 | [9491](https://www.ncbi.nlm.nih.gov/gene/?term=9491) | -3.9 | 0.00000 | 3263.9 | 14030.4 |
| LOC107987045 |  | [107987045](https://www.ncbi.nlm.nih.gov/gene/?term=107987045) | 3.9 | 0.00003 | 28.6 | 6.6 |
| LOC107984586 |  | [107984586](https://www.ncbi.nlm.nih.gov/gene/?term=107984586) | -3.9 | 0.00096 | 5.3 | 26.2 |
| RGL4 | ral guanine nucleotide dissociation stimulator like 4 | [266747](https://www.ncbi.nlm.nih.gov/gene/?term=266747) | 3.9 | 0.00000 | 1214.7 | 288.6 |
| LOC107987182 |  | [107987182](https://www.ncbi.nlm.nih.gov/gene/?term=107987182) | -3.9 | 0.00187 | 4.1 | 21.7 |
| SEPT4 | septin 4 | [5414](https://www.ncbi.nlm.nih.gov/gene/?term=5414) | 3.9 | 0.00006 | 126.6 | 28.2 |
| UCP2 | uncoupling protein 2 | [7351](https://www.ncbi.nlm.nih.gov/gene/?term=7351) | -3.9 | 0.00000 | 850.1 | 3525.8 |
| GLYATL2 | glycine-N-acyltransferase like 2 | [219970](https://www.ncbi.nlm.nih.gov/gene/?term=219970) | -3.8 | 0.00357 | 3.0 | 16.1 |
| LOC107986347 |  | [107986347](https://www.ncbi.nlm.nih.gov/gene/?term=107986347) | -3.8 | 0.00038 | 4.9 | 23.3 |
| LOC107985242 |  | [107985242](https://www.ncbi.nlm.nih.gov/gene/?term=107985242) | -3.8 | 0.00012 | 4.1 | 19.3 |
| ATF3 | activating transcription factor 3 | [467](https://www.ncbi.nlm.nih.gov/gene/?term=467) | 3.8 | 0.00022 | 102.3 | 22.9 |
| HBD | hemoglobin subunit delta | [3045](https://www.ncbi.nlm.nih.gov/gene/?term=3045) | -3.8 | 0.00009 | 21.5 | 93.3 |
| LOC101929398 |  | [101929398](https://www.ncbi.nlm.nih.gov/gene/?term=101929398) | 3.8 | 0.00001 | 16.9 | 4.0 |
| TIFA | TRAF interacting protein with forkhead associated domain | [92610](https://www.ncbi.nlm.nih.gov/gene/?term=92610) | 3.8 | 0.00000 | 2166.2 | 534.9 |
| LOC105370265 |  | [105370265](https://www.ncbi.nlm.nih.gov/gene/?term=105370265) | -3.8 | 0.00003 | 19.4 | 84.1 |
| SLC25A27 | solute carrier family 25 member 27 | [9481](https://www.ncbi.nlm.nih.gov/gene/?term=9481) | -3.8 | 0.00079 | 1.6 | 7.6 |
| PTPRU | protein tyrosine phosphatase, receptor type U | [10076](https://www.ncbi.nlm.nih.gov/gene/?term=10076) | 3.8 | 0.00258 | 8.1 | 1.6 |
| ALOX15 | arachidonate 15-lipoxygenase | [246](https://www.ncbi.nlm.nih.gov/gene/?term=246) | -3.8 | 0.00236 | 32.1 | 160.8 |
| MIR1255A | microRNA 1255a | [100302193](https://www.ncbi.nlm.nih.gov/gene/?term=100302193) | -3.8 | 0.00017 | 6.3 | 28.1 |
| HSP90B2P | heat shock protein 90kDa beta family member 2, pseudogene | [7190](https://www.ncbi.nlm.nih.gov/gene/?term=7190) | -3.8 | 0.00014 | 5.7 | 26.9 |
| MIR3945 | microRNA 3945 | [100500818](https://www.ncbi.nlm.nih.gov/gene/?term=100500818) | 3.7 | 0.00021 | 25.6 | 5.9 |
| LOC729291 |  | [729291](https://www.ncbi.nlm.nih.gov/gene/?term=729291) | -3.7 | 0.00000 | 29.8 | 121.1 |
| LOC100507586 | cytochrome c oxidase subunit 7B pseudogene | [100507586](https://www.ncbi.nlm.nih.gov/gene/?term=100507586) | -3.7 | 0.00057 | 2.4 | 11.9 |
| LOC100506314 | uncharacterized LOC100506314 | [100506314](https://www.ncbi.nlm.nih.gov/gene/?term=100506314) | -3.7 | 0.01654 | 0.4 | 2.9 |
| LOC102724608 |  | [102724608](https://www.ncbi.nlm.nih.gov/gene/?term=102724608) | 3.7 | 0.00000 | 412.9 | 101.9 |
| GALNT14 | polypeptide N-acetylgalactosaminyltransferase 14 | [79623](https://www.ncbi.nlm.nih.gov/gene/?term=79623) | 3.7 | 0.00003 | 364.7 | 86.9 |
| SNORD98 | small nucleolar RNA, C/D box 98 | [692211](https://www.ncbi.nlm.nih.gov/gene/?term=692211) | -3.7 | 0.00047 | 3.3 | 16.4 |
| LOC100996792 |  | [100996792](https://www.ncbi.nlm.nih.gov/gene/?term=100996792) | -3.7 | 0.00162 | 3.1 | 12.8 |
| RPS6KA6 | ribosomal protein S6 kinase A6 | [27330](https://www.ncbi.nlm.nih.gov/gene/?term=27330) | -3.7 | 0.00232 | 1.5 | 7.5 |
| FBXO39 | F-box protein 39 | [162517](https://www.ncbi.nlm.nih.gov/gene/?term=162517) | 3.7 | 0.00163 | 61.0 | 12.9 |
| PFKFB3 | 6-phosphofructo-2-kinase/fructose-2,6-biphosphatase 3 | [5209](https://www.ncbi.nlm.nih.gov/gene/?term=5209) | 3.7 | 0.00001 | 2452.1 | 597.5 |
| GRIN3A | glutamate ionotropic receptor NMDA type subunit 3A | [116443](https://www.ncbi.nlm.nih.gov/gene/?term=116443) | 3.7 | 0.00011 | 75.2 | 17.6 |
| LOC645468 | insulin like growth factor 2 mRNA binding protein 3 pseudogene | [645468](https://www.ncbi.nlm.nih.gov/gene/?term=645468) | -3.7 | 0.00096 | 7.8 | 37.1 |
| GPM6A | glycoprotein M6A | [2823](https://www.ncbi.nlm.nih.gov/gene/?term=2823) | -3.7 | 0.00326 | 3.0 | 15.5 |
| TLK2P2 | tousled like kinase 2 pseudogene 2 | [553121](https://www.ncbi.nlm.nih.gov/gene/?term=553121) | -3.7 | 0.00006 | 3.0 | 14.0 |
| VCAM1 | vascular cell adhesion molecule 1 | [7412](https://www.ncbi.nlm.nih.gov/gene/?term=7412) | 3.7 | 0.01031 | 9.6 | 1.6 |
| LOC646358 | DnaJ heat shock protein family (Hsp40) member B14 pseudogene | [646358](https://www.ncbi.nlm.nih.gov/gene/?term=646358) | -3.7 | 0.00003 | 49.9 | 208.3 |
| LOC107986278 |  | [107986278](https://www.ncbi.nlm.nih.gov/gene/?term=107986278) | -3.7 | 0.00234 | 3.5 | 18.1 |
| IFITM1 | interferon induced transmembrane protein 1 | [8519](https://www.ncbi.nlm.nih.gov/gene/?term=8519) | 3.7 | 0.00000 | 26084.9 | 6717.2 |
| CLEC4D | C-type lectin domain family 4 member D | [338339](https://www.ncbi.nlm.nih.gov/gene/?term=338339) | 3.6 | 0.00000 | 1521.4 | 382.3 |
| LOC100130531 | ribosomal protein L39 pseudogene | [100130531](https://www.ncbi.nlm.nih.gov/gene/?term=100130531) | -3.6 | 0.00629 | 2.2 | 12.0 |
| S100A8 | S100 calcium binding protein A8 | [6279](https://www.ncbi.nlm.nih.gov/gene/?term=6279) | 3.6 | 0.00000 | 112235.2 | 28395.6 |
| ZDHHC19 | zinc finger DHHC-type containing 19 | [131540](https://www.ncbi.nlm.nih.gov/gene/?term=131540) | 3.6 | 0.00367 | 22.2 | 4.5 |
| CACNB4 | calcium voltage-gated channel auxiliary subunit beta 4 | [785](https://www.ncbi.nlm.nih.gov/gene/?term=785) | -3.6 | 0.00000 | 56.2 | 218.6 |
| LOC105378046 |  | [105378046](https://www.ncbi.nlm.nih.gov/gene/?term=105378046) | -3.6 | 0.00059 | 6.4 | 29.6 |
| RAB5B | RAB5B, member RAS oncogene family | [5869](https://www.ncbi.nlm.nih.gov/gene/?term=5869) | -3.6 | 0.00000 | 723.2 | 2809.9 |
| TP53INP2 | tumor protein p53 inducible nuclear protein 2 | [58476](https://www.ncbi.nlm.nih.gov/gene/?term=58476) | -3.6 | 0.00000 | 19.8 | 77.4 |
| GBP1 | guanylate binding protein 1 | [2633](https://www.ncbi.nlm.nih.gov/gene/?term=2633) | 3.6 | 0.00004 | 15221.2 | 3720.8 |
| FAM64A | family with sequence similarity 64 member A | [54478](https://www.ncbi.nlm.nih.gov/gene/?term=54478) | 3.6 | 0.00239 | 11.9 | 2.5 |
| GBP6 | guanylate binding protein family member 6 | [163351](https://www.ncbi.nlm.nih.gov/gene/?term=163351) | 3.6 | 0.00008 | 186.3 | 45.4 |
| ZNF208 | zinc finger protein 208 | [7757](https://www.ncbi.nlm.nih.gov/gene/?term=7757) | -3.6 | 0.00004 | 21.1 | 86.0 |
| IL1B | interleukin 1, beta | [3553](https://www.ncbi.nlm.nih.gov/gene/?term=3553) | 3.6 | 0.00000 | 3091.8 | 803.0 |
| CFAP58-AS1 | CFAP58 antisense RNA 1 (head to head) | [100505869](https://www.ncbi.nlm.nih.gov/gene/?term=100505869) | 3.6 | 0.00000 | 239.4 | 63.0 |
| LOC107983947 |  | [107983947](https://www.ncbi.nlm.nih.gov/gene/?term=107983947) | 3.6 | 0.00114 | 10.3 | 2.2 |
| CNGA4 | cyclic nucleotide gated channel alpha 4 | [1262](https://www.ncbi.nlm.nih.gov/gene/?term=1262) | 3.5 | 0.00374 | 10.4 | 2.3 |
| LOC107986361 |  | [107986361](https://www.ncbi.nlm.nih.gov/gene/?term=107986361) | -3.5 | 0.00002 | 42.2 | 168.5 |
| AGAP7P | ArfGAP with GTPase domain, ankyrin repeat and PH domain 7, pseudogene | [653268](https://www.ncbi.nlm.nih.gov/gene/?term=653268) | -3.5 | 0.00009 | 8.8 | 36.8 |
| MARCO | macrophage receptor with collagenous structure | [8685](https://www.ncbi.nlm.nih.gov/gene/?term=8685) | 3.5 | 0.00003 | 537.0 | 134.9 |
| LOC105378978 |  | [105378978](https://www.ncbi.nlm.nih.gov/gene/?term=105378978) | 3.5 | 0.00004 | 47.0 | 12.2 |
| BCL2A1 | BCL2 related protein A1 | [597](https://www.ncbi.nlm.nih.gov/gene/?term=597) | 3.5 | 0.00001 | 4839.0 | 1246.6 |
| TBC1D8 | TBC1 domain family member 8 | [11138](https://www.ncbi.nlm.nih.gov/gene/?term=11138) | 3.5 | 0.00000 | 1474.2 | 388.8 |
| LOC105373180 |  | [105373180](https://www.ncbi.nlm.nih.gov/gene/?term=105373180) | 3.5 | 0.01308 | 10.1 | 1.7 |
| HBBP1 | hemoglobin subunit beta pseudogene 1 | [3044](https://www.ncbi.nlm.nih.gov/gene/?term=3044) | -3.5 | 0.01099 | 4.5 | 27.1 |
| FCAR | Fc fragment of IgA receptor | [2204](https://www.ncbi.nlm.nih.gov/gene/?term=2204) | 3.5 | 0.00317 | 51.4 | 11.0 |
| LOC107987145 |  | [107987145](https://www.ncbi.nlm.nih.gov/gene/?term=107987145) | 3.5 | 0.00045 | 36.2 | 8.7 |
| CCNI | cyclin I | [10983](https://www.ncbi.nlm.nih.gov/gene/?term=10983) | -3.5 | 0.00000 | 9369.3 | 35196.0 |
| LOC105376591 |  | [105376591](https://www.ncbi.nlm.nih.gov/gene/?term=105376591) | -3.5 | 0.00014 | 3.2 | 11.8 |
| NFIA | nuclear factor I A | [4774](https://www.ncbi.nlm.nih.gov/gene/?term=4774) | -3.5 | 0.00000 | 653.3 | 2421.7 |
| ETV7 | ETS variant 7 | [51513](https://www.ncbi.nlm.nih.gov/gene/?term=51513) | 3.5 | 0.00517 | 577.1 | 118.5 |
| SCO2 | SCO2 cytochrome c oxidase assembly protein | [9997](https://www.ncbi.nlm.nih.gov/gene/?term=9997) | 3.5 | 0.00000 | 1436.1 | 389.2 |
| LOC107985972 |  | [107985972](https://www.ncbi.nlm.nih.gov/gene/?term=107985972) | -3.5 | 0.00002 | 9.8 | 39.6 |
| LOC105369349 |  | [105369349](https://www.ncbi.nlm.nih.gov/gene/?term=105369349) | 3.5 | 0.00368 | 8.0 | 1.6 |
| LOC105374162 |  | [105374162](https://www.ncbi.nlm.nih.gov/gene/?term=105374162) | -3.5 | 0.00007 | 1086.3 | 4285.6 |
| LOC105376568 |  | [105376568](https://www.ncbi.nlm.nih.gov/gene/?term=105376568) | 3.4 | 0.00228 | 326.7 | 73.8 |
| TLR5 | toll like receptor 5 | [7100](https://www.ncbi.nlm.nih.gov/gene/?term=7100) | 3.4 | 0.00000 | 1948.2 | 525.1 |
| ST3GAL4 | ST3 beta-galactoside alpha-2,3-sialyltransferase 4 | [6484](https://www.ncbi.nlm.nih.gov/gene/?term=6484) | 3.4 | 0.00000 | 815.9 | 224.3 |
| MIR1284 | microRNA 1284 | [100302112](https://www.ncbi.nlm.nih.gov/gene/?term=100302112) | -3.4 | 0.00562 | 1.9 | 9.7 |
| CDH2 | cadherin 2 | [1000](https://www.ncbi.nlm.nih.gov/gene/?term=1000) | -3.4 | 0.00001 | 6.8 | 27.0 |
| WNK1 | WNK lysine deficient protein kinase 1 | [65125](https://www.ncbi.nlm.nih.gov/gene/?term=65125) | -3.4 | 0.00001 | 23200.8 | 89139.1 |
| RSAD2 | radical S-adenosyl methionine domain containing 2 | [91543](https://www.ncbi.nlm.nih.gov/gene/?term=91543) | 3.4 | 0.00183 | 37447.2 | 8777.6 |
| LOC107986482 |  | [107986482](https://www.ncbi.nlm.nih.gov/gene/?term=107986482) | 3.4 | 0.00003 | 57.8 | 15.3 |
| PPM1N | protein phosphatase, Mg2+/Mn2+ dependent 1N (putative) | [147699](https://www.ncbi.nlm.nih.gov/gene/?term=147699) | 3.4 | 0.00000 | 85.5 | 23.7 |
| BMX | BMX non-receptor tyrosine kinase | [660](https://www.ncbi.nlm.nih.gov/gene/?term=660) | 3.4 | 0.00014 | 429.0 | 110.6 |
| TNFSF10 | tumor necrosis factor superfamily member 10 | [8743](https://www.ncbi.nlm.nih.gov/gene/?term=8743) | 3.4 | 0.00000 | 24086.2 | 6658.8 |
| LOC105370821 |  | [105370821](https://www.ncbi.nlm.nih.gov/gene/?term=105370821) | -3.4 | 0.00149 | 2.3 | 9.6 |
| ARL4AP2 | ADP ribosylation factor like GTPase 4A pseudogene 2 | [152709](https://www.ncbi.nlm.nih.gov/gene/?term=152709) | -3.4 | 0.00087 | 2.6 | 9.0 |
| S100A9 | S100 calcium binding protein A9 | [6280](https://www.ncbi.nlm.nih.gov/gene/?term=6280) | 3.4 | 0.00000 | 301309.5 | 81808.7 |
| CACNA1E | calcium voltage-gated channel subunit alpha1 E | [777](https://www.ncbi.nlm.nih.gov/gene/?term=777) | 3.4 | 0.00007 | 356.2 | 94.0 |
| LOC105377319 |  | [105377319](https://www.ncbi.nlm.nih.gov/gene/?term=105377319) | -3.4 | 0.00008 | 35.1 | 135.8 |
| LOC105378524 |  | [105378524](https://www.ncbi.nlm.nih.gov/gene/?term=105378524) | -3.3 | 0.00001 | 32.1 | 120.3 |
| LOC107984694 |  | [107984694](https://www.ncbi.nlm.nih.gov/gene/?term=107984694) | -3.3 | 0.00386 | 2.0 | 9.9 |
| GRAMD1C | GRAM domain containing 1C | [54762](https://www.ncbi.nlm.nih.gov/gene/?term=54762) | -3.3 | 0.00000 | 92.9 | 331.3 |
| RIN1 | Ras and Rab interactor 1 | [9610](https://www.ncbi.nlm.nih.gov/gene/?term=9610) | 3.3 | 0.00000 | 133.5 | 37.1 |
| ROM1 | retinal outer segment membrane protein 1 | [6094](https://www.ncbi.nlm.nih.gov/gene/?term=6094) | 3.3 | 0.00000 | 38.6 | 10.7 |
| LOC107986613 |  | [107986613](https://www.ncbi.nlm.nih.gov/gene/?term=107986613) | -3.3 | 0.00301 | 1.9 | 9.4 |
| IGLV10-54 | immunoglobulin lambda variable 10-54 | [28772](https://www.ncbi.nlm.nih.gov/gene/?term=28772) | 3.3 | 0.01428 | 101.3 | 17.8 |
| TRIM51EP |  | [399940](https://www.ncbi.nlm.nih.gov/gene/?term=399940) | -3.3 | 0.00001 | 4.7 | 17.3 |
| MAP2K3 | mitogen-activated protein kinase kinase 3 | [5606](https://www.ncbi.nlm.nih.gov/gene/?term=5606) | -3.3 | 0.00002 | 4192.7 | 15517.9 |
| TNFSF13B | tumor necrosis factor superfamily member 13b | [10673](https://www.ncbi.nlm.nih.gov/gene/?term=10673) | 3.3 | 0.00000 | 8459.7 | 2388.7 |
| LOC107984754 |  | [107984754](https://www.ncbi.nlm.nih.gov/gene/?term=107984754) | -3.3 | 0.00258 | 5.3 | 23.6 |
| FCER1G | Fc fragment of IgE receptor Ig | [2207](https://www.ncbi.nlm.nih.gov/gene/?term=2207) | 3.3 | 0.00000 | 7208.7 | 2063.5 |
| PIK3R2 | phosphoinositide-3-kinase regulatory subunit 2 | [5296](https://www.ncbi.nlm.nih.gov/gene/?term=5296) | -3.3 | 0.00001 | 248.4 | 903.2 |
| DCAF13P3 | DDB1 and CUL4 associated factor 13 pseudogene 3 | [100132724](https://www.ncbi.nlm.nih.gov/gene/?term=100132724) | -3.3 | 0.00001 | 13.9 | 51.9 |
| CLEC5A | C-type lectin domain family 5 member A | [23601](https://www.ncbi.nlm.nih.gov/gene/?term=23601) | 3.3 | 0.00001 | 516.9 | 142.8 |
| MAD2L1P1 | MAD2 mitotic arrest deficient-like 1 (yeast) pseudogene 1 | [326596](https://www.ncbi.nlm.nih.gov/gene/?term=326596) | -3.3 | 0.00485 | 2.0 | 9.9 |
| SFR1P1 | SFR1 pseudogene 1 | [100130090](https://www.ncbi.nlm.nih.gov/gene/?term=100130090) | -3.3 | 0.00224 | 4.1 | 17.5 |
| MYBPC3 | myosin binding protein C, cardiac | [4607](https://www.ncbi.nlm.nih.gov/gene/?term=4607) | 3.3 | 0.00000 | 244.7 | 69.6 |
| MIR3682 | microRNA 3682 | [100500850](https://www.ncbi.nlm.nih.gov/gene/?term=100500850) | -3.3 | 0.00225 | 2.2 | 9.9 |
| C8orf46 | chromosome 8 open reading frame 46 | [254778](https://www.ncbi.nlm.nih.gov/gene/?term=254778) | -3.3 | 0.00090 | 1.8 | 8.3 |
| ADAMTS5 | ADAM metallopeptidase with thrombospondin type 1 motif 5 | [11096](https://www.ncbi.nlm.nih.gov/gene/?term=11096) | -3.3 | 0.00160 | 10.3 | 40.8 |
| SEMA6B | semaphorin 6B | [10501](https://www.ncbi.nlm.nih.gov/gene/?term=10501) | 3.3 | 0.00057 | 30.2 | 8.0 |
| GADD45G | growth arrest and DNA damage inducible gamma | [10912](https://www.ncbi.nlm.nih.gov/gene/?term=10912) | 3.2 | 0.00000 | 70.0 | 20.3 |
| LMNB1 | lamin B1 | [4001](https://www.ncbi.nlm.nih.gov/gene/?term=4001) | 3.2 | 0.00000 | 5942.0 | 1709.4 |
| IL10RB-AS1 | IL10RB antisense RNA 1 (head to head) | [100288432](https://www.ncbi.nlm.nih.gov/gene/?term=100288432) | 3.2 | 0.00002 | 49.3 | 13.9 |
| RAB20 | RAB20, member RAS oncogene family | [55647](https://www.ncbi.nlm.nih.gov/gene/?term=55647) | 3.2 | 0.00002 | 652.4 | 182.6 |
| EPHX1 | epoxide hydrolase 1 | [2052](https://www.ncbi.nlm.nih.gov/gene/?term=2052) | -3.2 | 0.00013 | 5.9 | 23.1 |
| WASF2 | WAS protein family member 2 | [10163](https://www.ncbi.nlm.nih.gov/gene/?term=10163) | -3.2 | 0.00000 | 4880.6 | 17147.5 |
| TCEB1P19 | transcription elongation factor B subunit 1 pseudogene 19 | [100462880](https://www.ncbi.nlm.nih.gov/gene/?term=100462880) | -3.2 | 0.00000 | 56.5 | 196.6 |
| TPRG1-AS1 | TPRG1 antisense RNA 1 | [100874043](https://www.ncbi.nlm.nih.gov/gene/?term=100874043) | 3.2 | 0.00001 | 29.3 | 8.1 |
| BATF | basic leucine zipper ATF-like transcription factor | [10538](https://www.ncbi.nlm.nih.gov/gene/?term=10538) | 3.2 | 0.00000 | 529.6 | 156.8 |
| LAP3 | leucine aminopeptidase 3 | [51056](https://www.ncbi.nlm.nih.gov/gene/?term=51056) | 3.2 | 0.00003 | 6798.5 | 1891.7 |
| CDC42EP1 | CDC42 effector protein 1 | [11135](https://www.ncbi.nlm.nih.gov/gene/?term=11135) | -3.2 | 0.00234 | 14.4 | 59.5 |
| RNU4ATAC16P | RNA, U4atac small nuclear 16, pseudogene | [106481160](https://www.ncbi.nlm.nih.gov/gene/?term=106481160) | -3.2 | 0.00273 | 9.7 | 40.7 |
| DDX50P1 | DEAD-box helicase 50 pseudogene 1 | [192146](https://www.ncbi.nlm.nih.gov/gene/?term=192146) | -3.2 | 0.00045 | 14.3 | 55.2 |
| GAS6 | growth arrest specific 6 | [2621](https://www.ncbi.nlm.nih.gov/gene/?term=2621) | 3.2 | 0.00000 | 208.5 | 61.1 |
| LOC105376001 |  | [105376001](https://www.ncbi.nlm.nih.gov/gene/?term=105376001) | -3.2 | 0.00007 | 26.3 | 95.8 |
| WDR26 | WD repeat domain 26 | [80232](https://www.ncbi.nlm.nih.gov/gene/?term=80232) | -3.2 | 0.00002 | 23313.5 | 82334.3 |
| PIM3 | Pim-3 proto-oncogene, serine/threonine kinase | [415116](https://www.ncbi.nlm.nih.gov/gene/?term=415116) | 3.2 | 0.00000 | 2093.3 | 629.0 |
| LOC100506860 | uncharacterized LOC100506860 | [100506860](https://www.ncbi.nlm.nih.gov/gene/?term=100506860) | 3.2 | 0.00410 | 9.5 | 2.2 |
| LOC105374377 |  | [105374377](https://www.ncbi.nlm.nih.gov/gene/?term=105374377) | -3.2 | 0.00067 | 3.1 | 12.6 |
| SPATS2L | spermatogenesis associated serine rich 2 like | [26010](https://www.ncbi.nlm.nih.gov/gene/?term=26010) | 3.1 | 0.00102 | 977.1 | 256.6 |
| TCN2 | transcobalamin 2 | [6948](https://www.ncbi.nlm.nih.gov/gene/?term=6948) | 3.1 | 0.00001 | 218.5 | 63.0 |
| ACSL1 | acyl-CoA synthetase long-chain family member 1 | [2180](https://www.ncbi.nlm.nih.gov/gene/?term=2180) | 3.1 | 0.00001 | 49431.1 | 14324.7 |
| LAMP3 | lysosomal associated membrane protein 3 | [27074](https://www.ncbi.nlm.nih.gov/gene/?term=27074) | 3.1 | 0.00169 | 285.2 | 73.2 |
| TOR1B | torsin family 1 member B | [27348](https://www.ncbi.nlm.nih.gov/gene/?term=27348) | 3.1 | 0.00000 | 2475.9 | 746.2 |
| PRSS33 | protease, serine 33 | [260429](https://www.ncbi.nlm.nih.gov/gene/?term=260429) | -3.1 | 0.00278 | 18.4 | 74.3 |
| INSC | inscuteable homolog (Drosophila) | [387755](https://www.ncbi.nlm.nih.gov/gene/?term=387755) | 3.1 | 0.00123 | 18.0 | 4.9 |
| LOC107985599 |  | [107985599](https://www.ncbi.nlm.nih.gov/gene/?term=107985599) | -3.1 | 0.00039 | 30.9 | 114.3 |
| GPR27 | G protein-coupled receptor 27 | [2850](https://www.ncbi.nlm.nih.gov/gene/?term=2850) | 3.1 | 0.00000 | 331.5 | 98.8 |
| CCDC71L | coiled-coil domain containing 71-like | [168455](https://www.ncbi.nlm.nih.gov/gene/?term=168455) | 3.1 | 0.00000 | 2002.2 | 606.2 |
| SHISA7 | shisa family member 7 | [729956](https://www.ncbi.nlm.nih.gov/gene/?term=729956) | -3.1 | 0.00236 | 40.7 | 160.5 |
| RNASE1 | ribonuclease A family member 1, pancreatic | [6035](https://www.ncbi.nlm.nih.gov/gene/?term=6035) | 3.1 | 0.00528 | 75.7 | 18.1 |
| MIR1537 | microRNA 1537 | [100302139](https://www.ncbi.nlm.nih.gov/gene/?term=100302139) | -3.1 | 0.00219 | 11.1 | 44.8 |
| MIR3198-1 | microRNA 3198-1 | [100423025](https://www.ncbi.nlm.nih.gov/gene/?term=100423025) | -3.1 | 0.00123 | 6.3 | 25.2 |
| TNS1 | tensin 1 | [7145](https://www.ncbi.nlm.nih.gov/gene/?term=7145) | -3.1 | 0.00017 | 9549.7 | 33851.5 |
| TRIM22 | tripartite motif containing 22 | [10346](https://www.ncbi.nlm.nih.gov/gene/?term=10346) | 3.1 | 0.00000 | 23279.6 | 6994.4 |
| CNTN4-AS1 | CNTN4 antisense RNA 1 | [100873975](https://www.ncbi.nlm.nih.gov/gene/?term=100873975) | -3.1 | 0.01012 | 2.4 | 11.2 |
| LOC100216355 | methylmalonic aciduria and homocystinuria, cblD type pseudogene | [100216355](https://www.ncbi.nlm.nih.gov/gene/?term=100216355) | -3.1 | 0.00411 | 4.3 | 17.5 |
| LOC107984292 |  | [107984292](https://www.ncbi.nlm.nih.gov/gene/?term=107984292) | -3.1 | 0.00080 | 45.2 | 166.9 |
| PLVAP | plasmalemma vesicle associated protein | [83483](https://www.ncbi.nlm.nih.gov/gene/?term=83483) | -3.1 | 0.00017 | 328.8 | 1150.6 |
| LOC105371730 | uncharacterized LOC105371730 | [105371730](https://www.ncbi.nlm.nih.gov/gene/?term=105371730) | 3.1 | 0.00038 | 15.0 | 4.2 |
| CYB5R2 | cytochrome b5 reductase 2 | [51700](https://www.ncbi.nlm.nih.gov/gene/?term=51700) | 3.1 | 0.00151 | 24.5 | 6.5 |
| CARD16 | caspase recruitment domain family member 16 | [114769](https://www.ncbi.nlm.nih.gov/gene/?term=114769) | 3.1 | 0.00000 | 5052.7 | 1564.4 |
| G0S2 | G0/G1 switch 2 | [50486](https://www.ncbi.nlm.nih.gov/gene/?term=50486) | 3.1 | 0.00031 | 130.8 | 37.2 |
| TYMP | thymidine phosphorylase | [1890](https://www.ncbi.nlm.nih.gov/gene/?term=1890) | 3.1 | 0.00000 | 9455.2 | 2937.9 |
| LOC107985783 |  | [107985783](https://www.ncbi.nlm.nih.gov/gene/?term=107985783) | -3.1 | 0.00007 | 17.5 | 60.1 |
| HTRA1 | HtrA serine peptidase 1 | [5654](https://www.ncbi.nlm.nih.gov/gene/?term=5654) | 3.0 | 0.00079 | 16.6 | 4.5 |
| SLC7A5 | solute carrier family 7 member 5 | [8140](https://www.ncbi.nlm.nih.gov/gene/?term=8140) | -3.0 | 0.00022 | 563.0 | 1970.0 |
| NPTXR | neuronal pentraxin receptor | [23467](https://www.ncbi.nlm.nih.gov/gene/?term=23467) | -3.0 | 0.00111 | 3.6 | 14.1 |
| TIAL1 | TIA1 cytotoxic granule-associated RNA binding protein-like 1 | [7073](https://www.ncbi.nlm.nih.gov/gene/?term=7073) | -3.0 | 0.00000 | 768.0 | 2475.6 |
| MIR4451 | microRNA 4451 | [100616349](https://www.ncbi.nlm.nih.gov/gene/?term=100616349) | -3.0 | 0.00143 | 6.2 | 23.7 |
| LOC107986643 |  | [107986643](https://www.ncbi.nlm.nih.gov/gene/?term=107986643) | -3.0 | 0.00356 | 4.6 | 19.2 |
| YES1P1 | YES1 pseudogene 1 | [7526](https://www.ncbi.nlm.nih.gov/gene/?term=7526) | -3.0 | 0.00027 | 49.2 | 173.6 |
| LOC107985262 |  | [107985262](https://www.ncbi.nlm.nih.gov/gene/?term=107985262) | -3.0 | 0.00232 | 11.7 | 45.7 |
| LOC107985098 |  | [107985098](https://www.ncbi.nlm.nih.gov/gene/?term=107985098) | 3.0 | 0.00075 | 8.0 | 2.1 |
| DAP | death-associated protein | [1611](https://www.ncbi.nlm.nih.gov/gene/?term=1611) | -3.0 | 0.00001 | 705.7 | 2323.4 |
| CSRNP1 | cysteine and serine rich nuclear protein 1 | [64651](https://www.ncbi.nlm.nih.gov/gene/?term=64651) | 3.0 | 0.00000 | 1515.7 | 474.3 |
| SALL2 | spalt-like transcription factor 2 | [6297](https://www.ncbi.nlm.nih.gov/gene/?term=6297) | -3.0 | 0.00037 | 3.4 | 12.8 |
| LOC105369354 |  | [105369354](https://www.ncbi.nlm.nih.gov/gene/?term=105369354) | -3.0 | 0.00023 | 26.9 | 93.6 |
| LOC102723690 |  | [102723690](https://www.ncbi.nlm.nih.gov/gene/?term=102723690) | -3.0 | 0.00059 | 3.6 | 13.7 |
| MX1 | MX dynamin like GTPase 1 | [4599](https://www.ncbi.nlm.nih.gov/gene/?term=4599) | 3.0 | 0.00122 | 17715.4 | 4935.5 |
| MAP1B | microtubule associated protein 1B | [4131](https://www.ncbi.nlm.nih.gov/gene/?term=4131) | -3.0 | 0.00171 | 13.6 | 48.7 |
| LOC105374010 |  | [105374010](https://www.ncbi.nlm.nih.gov/gene/?term=105374010) | -3.0 | 0.00008 | 37.5 | 127.7 |
| GALM | galactose mutarotase (aldose 1-epimerase) | [130589](https://www.ncbi.nlm.nih.gov/gene/?term=130589) | 3.0 | 0.00000 | 959.5 | 299.4 |
| TDRD9 | tudor domain containing 9 | [122402](https://www.ncbi.nlm.nih.gov/gene/?term=122402) | 3.0 | 0.00001 | 310.2 | 95.8 |
| XAF1 | XIAP associated factor 1 | [54739](https://www.ncbi.nlm.nih.gov/gene/?term=54739) | 3.0 | 0.00027 | 12285.8 | 3594.9 |
| PHF11 | PHD finger protein 11 | [51131](https://www.ncbi.nlm.nih.gov/gene/?term=51131) | 3.0 | 0.00005 | 33.9 | 10.2 |
| LOC105375142 |  | [105375142](https://www.ncbi.nlm.nih.gov/gene/?term=105375142) | -3.0 | 0.00167 | 2.7 | 11.0 |
| ANKUB1 | ankyrin repeat and ubiquitin domain containing 1 | [389161](https://www.ncbi.nlm.nih.gov/gene/?term=389161) | -3.0 | 0.00121 | 8.7 | 29.5 |
| OLFML2B | olfactomedin like 2B | [25903](https://www.ncbi.nlm.nih.gov/gene/?term=25903) | 3.0 | 0.00082 | 23.7 | 6.9 |
| LOC105374306 |  | [105374306](https://www.ncbi.nlm.nih.gov/gene/?term=105374306) | -3.0 | 0.00358 | 3.2 | 12.6 |
| PGS1 | phosphatidylglycerophosphate synthase 1 | [9489](https://www.ncbi.nlm.nih.gov/gene/?term=9489) | 3.0 | 0.00000 | 3403.4 | 1080.3 |
| LOC613037 | nuclear pore complex interacting protein member | [613037](https://www.ncbi.nlm.nih.gov/gene/?term=613037) | -3.0 | 0.00000 | 79.9 | 250.3 |
| FSIP2 | fibrous sheath interacting protein 2 | [401024](https://www.ncbi.nlm.nih.gov/gene/?term=401024) | -3.0 | 0.00722 | 4.1 | 17.6 |
| PPP3R1 | protein phosphatase 3 regulatory subunit B, alpha | [5534](https://www.ncbi.nlm.nih.gov/gene/?term=5534) | -2.9 | 0.00000 | 8174.1 | 25851.3 |
| LOC105370355 |  | [105370355](https://www.ncbi.nlm.nih.gov/gene/?term=105370355) | 2.9 | 0.00258 | 49.1 | 13.4 |
| EBF1 | early B-cell factor 1 | [1879](https://www.ncbi.nlm.nih.gov/gene/?term=1879) | -2.9 | 0.00004 | 304.9 | 994.4 |
| WASF4P | WAS protein family member 4, pseudogene | [644739](https://www.ncbi.nlm.nih.gov/gene/?term=644739) | -2.9 | 0.00044 | 4.8 | 16.0 |
| RPL21P48 | ribosomal protein L21 pseudogene 48 | [100271165](https://www.ncbi.nlm.nih.gov/gene/?term=100271165) | -2.9 | 0.00839 | 2.7 | 11.7 |
| HCAR3 | hydroxycarboxylic acid receptor 3 | [8843](https://www.ncbi.nlm.nih.gov/gene/?term=8843) | 2.9 | 0.00001 | 2347.4 | 736.8 |
| LOC101927989 | uncharacterized LOC101927989 | [101927989](https://www.ncbi.nlm.nih.gov/gene/?term=101927989) | 2.9 | 0.00027 | 10.9 | 3.2 |
| LOC105379130 |  | [105379130](https://www.ncbi.nlm.nih.gov/gene/?term=105379130) | -2.9 | 0.00646 | 3.1 | 11.1 |
| LOC105373429 |  | [105373429](https://www.ncbi.nlm.nih.gov/gene/?term=105373429) | -2.9 | 0.00260 | 11.0 | 39.8 |
| LOC107984999 |  | [107984999](https://www.ncbi.nlm.nih.gov/gene/?term=107984999) | -2.9 | 0.00013 | 10.6 | 36.3 |
| MICU3 | mitochondrial calcium uptake family member 3 | [286097](https://www.ncbi.nlm.nih.gov/gene/?term=286097) | -2.9 | 0.00000 | 73.5 | 231.9 |
| LOC102723446 |  | [102723446](https://www.ncbi.nlm.nih.gov/gene/?term=102723446) | -2.9 | 0.00345 | 5.1 | 19.2 |
| LOC107985867 |  | [107985867](https://www.ncbi.nlm.nih.gov/gene/?term=107985867) | -2.9 | 0.00273 | 6.4 | 24.5 |
| HCAR2 | hydroxycarboxylic acid receptor 2 | [338442](https://www.ncbi.nlm.nih.gov/gene/?term=338442) | 2.9 | 0.00001 | 1885.5 | 598.0 |
| BRD4 | bromodomain containing 4 | [23476](https://www.ncbi.nlm.nih.gov/gene/?term=23476) | -2.9 | 0.00001 | 1081.2 | 3400.3 |
| GBP3 | guanylate binding protein 3 | [2635](https://www.ncbi.nlm.nih.gov/gene/?term=2635) | 2.9 | 0.00000 | 2827.1 | 906.3 |
| GYG1 | glycogenin 1 | [2992](https://www.ncbi.nlm.nih.gov/gene/?term=2992) | 2.9 | 0.00004 | 1616.4 | 508.5 |
| NPIPB3 | nuclear pore complex interacting protein family member B3 | [23117](https://www.ncbi.nlm.nih.gov/gene/?term=23117) | -2.9 | 0.00005 | 6.3 | 19.3 |
| RARA-AS1 | RARA antisense RNA 1 | [101929693](https://www.ncbi.nlm.nih.gov/gene/?term=101929693) | 2.9 | 0.00000 | 329.7 | 109.5 |
| GADD45B | growth arrest and DNA damage inducible beta | [4616](https://www.ncbi.nlm.nih.gov/gene/?term=4616) | 2.9 | 0.00000 | 1291.4 | 425.3 |
| TSPAN7 | tetraspanin 7 | [7102](https://www.ncbi.nlm.nih.gov/gene/?term=7102) | -2.9 | 0.00051 | 78.1 | 259.5 |
| SLC2A3 | solute carrier family 2 member 3 | [6515](https://www.ncbi.nlm.nih.gov/gene/?term=6515) | 2.9 | 0.00001 | 20900.8 | 6669.3 |
| FAM225A | family with sequence similarity 225 member A (non-protein coding) | [286333](https://www.ncbi.nlm.nih.gov/gene/?term=286333) | 2.9 | 0.01144 | 8.5 | 2.0 |
| HEBP1 | heme binding protein 1 | [50865](https://www.ncbi.nlm.nih.gov/gene/?term=50865) | -2.9 | 0.00150 | 124.5 | 433.0 |
| RAB36 | RAB36, member RAS oncogene family | [9609](https://www.ncbi.nlm.nih.gov/gene/?term=9609) | -2.9 | 0.00008 | 7.2 | 23.4 |
| THAP12P7 | THAP domain containing 12 pseudogene 7 | [100422712](https://www.ncbi.nlm.nih.gov/gene/?term=100422712) | -2.9 | 0.00001 | 9.4 | 30.3 |
| LOC105369469 |  | [105369469](https://www.ncbi.nlm.nih.gov/gene/?term=105369469) | 2.9 | 0.00084 | 17.0 | 5.0 |
| CLRN1-AS1 | CLRN1 antisense RNA 1 | [116933](https://www.ncbi.nlm.nih.gov/gene/?term=116933) | 2.9 | 0.02174 | 9.9 | 1.8 |
| FTH1P11 | ferritin, heavy polypeptide 1 pseudogene 11 | [2503](https://www.ncbi.nlm.nih.gov/gene/?term=2503) | -2.9 | 0.00257 | 2.1 | 8.2 |
| LOC107984713 |  | [107984713](https://www.ncbi.nlm.nih.gov/gene/?term=107984713) | -2.9 | 0.00144 | 11.1 | 39.4 |
| GPRC5C | G protein-coupled receptor class C group 5 member C | [55890](https://www.ncbi.nlm.nih.gov/gene/?term=55890) | 2.9 | 0.00183 | 14.5 | 4.0 |
| LOC105373098 |  | [105373098](https://www.ncbi.nlm.nih.gov/gene/?term=105373098) | 2.8 | 0.00004 | 66.4 | 21.1 |
| PRNCR1 | prostate cancer associated non-coding RNA 1 | [101867536](https://www.ncbi.nlm.nih.gov/gene/?term=101867536) | -2.8 | 0.00579 | 14.4 | 55.3 |
| OR2T33 | olfactory receptor family 2 subfamily T member 33 | [391195](https://www.ncbi.nlm.nih.gov/gene/?term=391195) | -2.8 | 0.01584 | 3.4 | 15.5 |
| KCNQ5-IT1 | KCNQ5 intronic transcript 1 | [100507381](https://www.ncbi.nlm.nih.gov/gene/?term=100507381) | -2.8 | 0.00029 | 16.0 | 52.8 |
| LOC100505798 |  | [100505798](https://www.ncbi.nlm.nih.gov/gene/?term=100505798) | 2.8 | 0.00000 | 360.0 | 122.0 |
| ANOS1 | anosmin 1 | [3730](https://www.ncbi.nlm.nih.gov/gene/?term=3730) | 2.8 | 0.00595 | 21.5 | 5.6 |
| MAPK14 | mitogen-activated protein kinase 14 | [1432](https://www.ncbi.nlm.nih.gov/gene/?term=1432) | 2.8 | 0.00000 | 13146.3 | 4404.9 |
| JAZF1 | JAZF zinc finger 1 | [221895](https://www.ncbi.nlm.nih.gov/gene/?term=221895) | -2.8 | 0.00008 | 3919.0 | 12333.6 |
| SIPA1L2 | signal induced proliferation associated 1 like 2 | [57568](https://www.ncbi.nlm.nih.gov/gene/?term=57568) | 2.8 | 0.00004 | 2187.5 | 704.5 |
| LINC01503 | long intergenic non-protein coding RNA 1503 | [100506119](https://www.ncbi.nlm.nih.gov/gene/?term=100506119) | 2.8 | 0.00000 | 66.5 | 22.2 |
| FLVCR2 | feline leukemia virus subgroup C cellular receptor family member 2 | [55640](https://www.ncbi.nlm.nih.gov/gene/?term=55640) | 2.8 | 0.00000 | 705.8 | 234.9 |
| LOC107984474 |  | [107984474](https://www.ncbi.nlm.nih.gov/gene/?term=107984474) | 2.8 | 0.00040 | 56.2 | 17.5 |
| MIR99AHG | mir-99a-let-7c cluster host gene | [388815](https://www.ncbi.nlm.nih.gov/gene/?term=388815) | -2.8 | 0.00964 | 1.9 | 8.3 |
| KIF19 | kinesin family member 19 | [124602](https://www.ncbi.nlm.nih.gov/gene/?term=124602) | 2.8 | 0.00538 | 25.3 | 6.9 |
| LOC107985727 |  | [107985727](https://www.ncbi.nlm.nih.gov/gene/?term=107985727) | -2.8 | 0.00229 | 10.6 | 37.7 |
| BFSP2-AS1 | BFSP2 antisense RNA 1 | [85003](https://www.ncbi.nlm.nih.gov/gene/?term=85003) | -2.8 | 0.00080 | 6.0 | 19.2 |
| LOC100420587 | SHC binding and spindle associated 1 pseudogene | [100420587](https://www.ncbi.nlm.nih.gov/gene/?term=100420587) | -2.8 | 0.00069 | 10.8 | 34.0 |
| STEAP3 | STEAP3 metalloreductase | [55240](https://www.ncbi.nlm.nih.gov/gene/?term=55240) | -2.8 | 0.00224 | 93.9 | 322.0 |
| PCAT29 | prostate cancer associated transcript 29 (non-protein coding) | [104472713](https://www.ncbi.nlm.nih.gov/gene/?term=104472713) | -2.8 | 0.00319 | 2.2 | 7.7 |
| MORF4L2-AS1 | MORF4L2 antisense RNA 1 | [340544](https://www.ncbi.nlm.nih.gov/gene/?term=340544) | -2.8 | 0.00009 | 6.0 | 19.6 |
| CYP7B1 | cytochrome P450 family 7 subfamily B member 1 | [9420](https://www.ncbi.nlm.nih.gov/gene/?term=9420) | -2.8 | 0.00005 | 23.6 | 72.7 |
| BASP1 | brain abundant membrane attached signal protein 1 | [10409](https://www.ncbi.nlm.nih.gov/gene/?term=10409) | 2.8 | 0.00002 | 7452.3 | 2447.6 |
| ANKRD20A5P | ankyrin repeat domain 20 family member A5, pseudogene | [440482](https://www.ncbi.nlm.nih.gov/gene/?term=440482) | -2.8 | 0.00038 | 15.7 | 51.3 |
| FAM122B | family with sequence similarity 122B | [159090](https://www.ncbi.nlm.nih.gov/gene/?term=159090) | -2.8 | 0.00001 | 475.5 | 1435.6 |
| IFIH1 | interferon induced with helicase C domain 1 | [64135](https://www.ncbi.nlm.nih.gov/gene/?term=64135) | 2.8 | 0.00004 | 5992.1 | 1946.7 |
| EFEMP2 | EGF containing fibulin-like extracellular matrix protein 2 | [30008](https://www.ncbi.nlm.nih.gov/gene/?term=30008) | 2.8 | 0.00000 | 72.2 | 24.0 |
| LOC107984987 |  | [107984987](https://www.ncbi.nlm.nih.gov/gene/?term=107984987) | -2.8 | 0.00544 | 5.0 | 19.1 |
| LOC105369734 |  | [105369734](https://www.ncbi.nlm.nih.gov/gene/?term=105369734) | 2.8 | 0.02251 | 8.8 | 1.7 |
| KCNJ15 | potassium voltage-gated channel subfamily J member 15 | [3772](https://www.ncbi.nlm.nih.gov/gene/?term=3772) | 2.8 | 0.00002 | 12335.3 | 4064.1 |
| ENAH | enabled homolog (Drosophila) | [55740](https://www.ncbi.nlm.nih.gov/gene/?term=55740) | -2.8 | 0.00035 | 7.6 | 23.2 |
| EPSTI1 | epithelial stromal interaction 1 (breast) | [94240](https://www.ncbi.nlm.nih.gov/gene/?term=94240) | 2.8 | 0.00029 | 11314.2 | 3598.2 |
| LOC107984698 |  | [107984698](https://www.ncbi.nlm.nih.gov/gene/?term=107984698) | 2.8 | 0.00914 | 6.6 | 1.9 |
| LBX2 | ladybird homeobox 2 | [85474](https://www.ncbi.nlm.nih.gov/gene/?term=85474) | 2.8 | 0.00073 | 13.1 | 4.0 |
| ALOX5 | arachidonate 5-lipoxygenase | [240](https://www.ncbi.nlm.nih.gov/gene/?term=240) | 2.8 | 0.00071 | 21.7 | 6.6 |
| ZBP1 | Z-DNA binding protein 1 | [81030](https://www.ncbi.nlm.nih.gov/gene/?term=81030) | 2.8 | 0.00005 | 2272.7 | 746.4 |
| DDAH1 | dimethylarginine dimethylaminohydrolase 1 | [23576](https://www.ncbi.nlm.nih.gov/gene/?term=23576) | -2.8 | 0.01147 | 2.4 | 9.7 |
| IL18RAP | interleukin 18 receptor accessory protein | [8807](https://www.ncbi.nlm.nih.gov/gene/?term=8807) | 2.8 | 0.00014 | 4792.9 | 1551.9 |
| LSMEM2 | leucine rich single-pass membrane protein 2 | [132228](https://www.ncbi.nlm.nih.gov/gene/?term=132228) | 2.8 | 0.01070 | 8.9 | 2.4 |
| PXDNL | peroxidasin like | [137902](https://www.ncbi.nlm.nih.gov/gene/?term=137902) | -2.8 | 0.00000 | 17.6 | 52.7 |
| SEMA3B | semaphorin 3B | [7869](https://www.ncbi.nlm.nih.gov/gene/?term=7869) | 2.8 | 0.00060 | 22.4 | 7.1 |
| LOC107987112 |  | [107987112](https://www.ncbi.nlm.nih.gov/gene/?term=107987112) | -2.8 | 0.00031 | 13.5 | 42.2 |
| LOC100421309 | DEAD-box helicase 25 pseudogene | [100421309](https://www.ncbi.nlm.nih.gov/gene/?term=100421309) | -2.8 | 0.00102 | 41.7 | 136.1 |
| PIK3AP1 | phosphoinositide-3-kinase adaptor protein 1 | [118788](https://www.ncbi.nlm.nih.gov/gene/?term=118788) | 2.8 | 0.00000 | 20454.6 | 7062.7 |
| A2M-AS1 | A2M antisense RNA 1 (head to head) | [144571](https://www.ncbi.nlm.nih.gov/gene/?term=144571) | -2.7 | 0.00085 | 68.1 | 220.6 |
| NPIPB5 | nuclear pore complex interacting protein family member B5 | [100132247](https://www.ncbi.nlm.nih.gov/gene/?term=100132247) | -2.7 | 0.00083 | 10.1 | 33.0 |
| BICDL2 | BICD family like cargo adaptor 2 | [146439](https://www.ncbi.nlm.nih.gov/gene/?term=146439) | 2.7 | 0.00645 | 45.3 | 12.7 |
| LOC100422294 | capping actin protein of muscle Z-line alpha subunit 1 pseudogene | [100422294](https://www.ncbi.nlm.nih.gov/gene/?term=100422294) | -2.7 | 0.00112 | 17.9 | 59.3 |
| SERPING1 | serpin family G member 1 | [710](https://www.ncbi.nlm.nih.gov/gene/?term=710) | 2.7 | 0.00933 | 691.9 | 183.6 |
| KIF26A | kinesin family member 26A | [26153](https://www.ncbi.nlm.nih.gov/gene/?term=26153) | 2.7 | 0.01053 | 140.7 | 36.7 |
| IFI44L | interferon induced protein 44 like | [10964](https://www.ncbi.nlm.nih.gov/gene/?term=10964) | 2.7 | 0.01386 | 15427.9 | 3844.6 |
| DDX60 | DEXD/H-box helicase 60 | [55601](https://www.ncbi.nlm.nih.gov/gene/?term=55601) | 2.7 | 0.00021 | 11763.7 | 3855.5 |
| TRAJ8 | T cell receptor alpha joining 8 | [28747](https://www.ncbi.nlm.nih.gov/gene/?term=28747) | -2.7 | 0.00063 | 10.3 | 33.0 |
| CST7 | cystatin F | [8530](https://www.ncbi.nlm.nih.gov/gene/?term=8530) | 2.7 | 0.00065 | 1075.7 | 344.8 |
| SUGCT | succinyl-CoA:glutarate-CoA transferase | [79783](https://www.ncbi.nlm.nih.gov/gene/?term=79783) | -2.7 | 0.00001 | 8.0 | 24.9 |
| KHDRBS2 | KH domain containing, RNA binding, signal transduction associated 2 | [202559](https://www.ncbi.nlm.nih.gov/gene/?term=202559) | -2.7 | 0.00001 | 75.4 | 218.5 |
| LOC105374902 |  | [105374902](https://www.ncbi.nlm.nih.gov/gene/?term=105374902) | 2.7 | 0.00077 | 22.4 | 7.3 |
| LOC107987081 |  | [107987081](https://www.ncbi.nlm.nih.gov/gene/?term=107987081) | -2.7 | 0.00007 | 398.8 | 1190.5 |
| LOC105376786 |  | [105376786](https://www.ncbi.nlm.nih.gov/gene/?term=105376786) | 2.7 | 0.00154 | 45.2 | 14.0 |
| ZNF684 | zinc finger protein 684 | [127396](https://www.ncbi.nlm.nih.gov/gene/?term=127396) | 2.7 | 0.00000 | 295.2 | 102.4 |
| LOC107984139 |  | [107984139](https://www.ncbi.nlm.nih.gov/gene/?term=107984139) | -2.7 | 0.00000 | 14.7 | 43.4 |
| LOC105377267 | uncharacterized LOC105377267 | [105377267](https://www.ncbi.nlm.nih.gov/gene/?term=105377267) | -2.7 | 0.00232 | 55.5 | 181.3 |
| HIGD1AP14 | HIG1 hypoxia inducible domain family member 1A pseudogene 14 | [100874455](https://www.ncbi.nlm.nih.gov/gene/?term=100874455) | -2.7 | 0.00171 | 12.9 | 42.5 |
| MDK | midkine (neurite growth-promoting factor 2) | [4192](https://www.ncbi.nlm.nih.gov/gene/?term=4192) | 2.7 | 0.00035 | 18.4 | 6.0 |
| TAF9P3 | TATA-box binding protein associated factor 9 pseudogene 3 | [163088](https://www.ncbi.nlm.nih.gov/gene/?term=163088) | -2.7 | 0.00338 | 8.1 | 27.4 |
| LOC101927949 |  | [101927949](https://www.ncbi.nlm.nih.gov/gene/?term=101927949) | 2.7 | 0.00017 | 31.0 | 10.2 |
| IFI44 | interferon induced protein 44 | [10561](https://www.ncbi.nlm.nih.gov/gene/?term=10561) | 2.7 | 0.00612 | 10195.0 | 2944.3 |
| FLJ46906 | uncharacterized LOC441172 | [441172](https://www.ncbi.nlm.nih.gov/gene/?term=441172) | 2.7 | 0.00019 | 12.4 | 4.0 |
| NFAT5 | nuclear factor of activated T-cells 5, tonicity-responsive | [10725](https://www.ncbi.nlm.nih.gov/gene/?term=10725) | -2.7 | 0.00000 | 2439.8 | 6964.1 |
| DLG2 | discs large homolog 2 | [1740](https://www.ncbi.nlm.nih.gov/gene/?term=1740) | -2.7 | 0.00001 | 6.3 | 17.7 |
| LOC107985047 |  | [107985047](https://www.ncbi.nlm.nih.gov/gene/?term=107985047) | 2.7 | 0.01067 | 11.9 | 3.4 |
| UPP1 | uridine phosphorylase 1 | [7378](https://www.ncbi.nlm.nih.gov/gene/?term=7378) | 2.7 | 0.00000 | 1249.6 | 441.0 |
| PTK6 | protein tyrosine kinase 6 | [5753](https://www.ncbi.nlm.nih.gov/gene/?term=5753) | 2.7 | 0.00000 | 24.8 | 8.9 |
| RPS3AP38 | ribosomal protein S3a pseudogene 38 | [100271594](https://www.ncbi.nlm.nih.gov/gene/?term=100271594) | -2.7 | 0.00011 | 22.3 | 67.0 |
| RANBP10 | RAN binding protein 10 | [57610](https://www.ncbi.nlm.nih.gov/gene/?term=57610) | -2.7 | 0.00010 | 2775.1 | 8271.4 |
| P2RY14 | purinergic receptor P2Y14 | [9934](https://www.ncbi.nlm.nih.gov/gene/?term=9934) | 2.7 | 0.00056 | 2031.7 | 664.0 |
| LOC107986699 |  | [107986699](https://www.ncbi.nlm.nih.gov/gene/?term=107986699) | -2.7 | 0.00012 | 29.2 | 87.6 |
| MIRLET7A1 | microRNA let-7a-1 | [406881](https://www.ncbi.nlm.nih.gov/gene/?term=406881) | -2.7 | 0.00032 | 3.9 | 11.7 |
| LOC107984943 |  | [107984943](https://www.ncbi.nlm.nih.gov/gene/?term=107984943) | 2.7 | 0.00044 | 72.0 | 23.8 |
| LOC100287497 | septin 7 pseudogene | [100287497](https://www.ncbi.nlm.nih.gov/gene/?term=100287497) | -2.7 | 0.00017 | 49.9 | 149.8 |
| LINC00671 | long intergenic non-protein coding RNA 671 | [388387](https://www.ncbi.nlm.nih.gov/gene/?term=388387) | 2.7 | 0.00251 | 42.4 | 13.2 |
| LOC101930164 |  | [101930164](https://www.ncbi.nlm.nih.gov/gene/?term=101930164) | 2.7 | 0.00055 | 305.9 | 100.9 |
| LOC105373311 |  | [105373311](https://www.ncbi.nlm.nih.gov/gene/?term=105373311) | 2.7 | 0.00004 | 42.9 | 14.7 |
| HSPD1P11 | heat shock protein family D (Hsp60) member 1 pseudogene 11 | [100359396](https://www.ncbi.nlm.nih.gov/gene/?term=100359396) | -2.7 | 0.00400 | 3.1 | 10.8 |
| DMTN | dematin actin binding protein | [2039](https://www.ncbi.nlm.nih.gov/gene/?term=2039) | -2.7 | 0.00025 | 6316.8 | 18911.3 |
| LOC101928166 | uncharacterized LOC101928166 | [101928166](https://www.ncbi.nlm.nih.gov/gene/?term=101928166) | -2.7 | 0.00004 | 17.0 | 49.4 |
| ADCY3 | adenylate cyclase 3 | [109](https://www.ncbi.nlm.nih.gov/gene/?term=109) | 2.7 | 0.00002 | 536.3 | 186.8 |
| AGRN | agrin | [375790](https://www.ncbi.nlm.nih.gov/gene/?term=375790) | 2.6 | 0.00087 | 205.9 | 66.9 |
| LINC01268 | long intergenic non-protein coding RNA 1268 | [285758](https://www.ncbi.nlm.nih.gov/gene/?term=285758) | 2.6 | 0.00799 | 13.3 | 3.9 |
| NOV | nephroblastoma overexpressed | [4856](https://www.ncbi.nlm.nih.gov/gene/?term=4856) | -2.6 | 0.00013 | 92.1 | 267.9 |
| F12 | coagulation factor XII | [2161](https://www.ncbi.nlm.nih.gov/gene/?term=2161) | 2.6 | 0.00012 | 41.4 | 13.9 |
| LOC107985115 |  | [107985115](https://www.ncbi.nlm.nih.gov/gene/?term=107985115) | 2.6 | 0.00103 | 21.4 | 6.8 |
| GPR52 | G protein-coupled receptor 52 | [9293](https://www.ncbi.nlm.nih.gov/gene/?term=9293) | -2.6 | 0.00274 | 17.8 | 58.5 |
| MFSD7 | major facilitator superfamily domain containing 7 | [84179](https://www.ncbi.nlm.nih.gov/gene/?term=84179) | 2.6 | 0.00000 | 290.1 | 103.4 |
| CKAP4 | cytoskeleton-associated protein 4 | [10970](https://www.ncbi.nlm.nih.gov/gene/?term=10970) | 2.6 | 0.00000 | 2076.2 | 737.3 |
| SDK2 | sidekick cell adhesion molecule 2 | [54549](https://www.ncbi.nlm.nih.gov/gene/?term=54549) | -2.6 | 0.00009 | 27.5 | 80.6 |
| MBNL3 | muscleblind like splicing regulator 3 | [55796](https://www.ncbi.nlm.nih.gov/gene/?term=55796) | -2.6 | 0.00025 | 76297.6 | 225849.0 |
| PCSK1N | proprotein convertase subtilisin/kexin type 1 inhibitor | [27344](https://www.ncbi.nlm.nih.gov/gene/?term=27344) | -2.6 | 0.00477 | 56.3 | 187.5 |
| NPIPB4 | nuclear pore complex interacting protein family member B4 | [440345](https://www.ncbi.nlm.nih.gov/gene/?term=440345) | -2.6 | 0.00015 | 88.6 | 258.5 |
| LOC729040 |  | [729040](https://www.ncbi.nlm.nih.gov/gene/?term=729040) | -2.6 | 0.00140 | 31.4 | 98.4 |
| PARP9 | poly(ADP-ribose) polymerase family member 9 | [83666](https://www.ncbi.nlm.nih.gov/gene/?term=83666) | 2.6 | 0.00001 | 12342.7 | 4352.4 |
| CASP1 | caspase 1 | [834](https://www.ncbi.nlm.nih.gov/gene/?term=834) | 2.6 | 0.00000 | 8721.8 | 3169.5 |
| PGM2L1 | phosphoglucomutase 2-like 1 | [283209](https://www.ncbi.nlm.nih.gov/gene/?term=283209) | -2.6 | 0.00024 | 2596.0 | 7694.6 |
| TREX1 | three prime repair exonuclease 1 | [11277](https://www.ncbi.nlm.nih.gov/gene/?term=11277) | 2.6 | 0.00000 | 438.0 | 158.4 |
| LOC105370108 |  | [105370108](https://www.ncbi.nlm.nih.gov/gene/?term=105370108) | -2.6 | 0.00055 | 11.8 | 35.9 |
| APOBEC3A | apolipoprotein B mRNA editing enzyme catalytic subunit 3A | [200315](https://www.ncbi.nlm.nih.gov/gene/?term=200315) | 2.6 | 0.00178 | 1499.1 | 484.5 |
| LOC107986646 |  | [107986646](https://www.ncbi.nlm.nih.gov/gene/?term=107986646) | -2.6 | 0.00028 | 8.0 | 23.2 |
| PARP12 | poly(ADP-ribose) polymerase family member 12 | [64761](https://www.ncbi.nlm.nih.gov/gene/?term=64761) | 2.6 | 0.00002 | 3478.2 | 1231.1 |
| LOC105377342 |  | [105377342](https://www.ncbi.nlm.nih.gov/gene/?term=105377342) | -2.6 | 0.00101 | 115.8 | 349.4 |
| LOC105373826 |  | [105373826](https://www.ncbi.nlm.nih.gov/gene/?term=105373826) | -2.6 | 0.00181 | 69.1 | 214.7 |
| PTGDR | prostaglandin D2 receptor (DP) | [5729](https://www.ncbi.nlm.nih.gov/gene/?term=5729) | -2.6 | 0.00062 | 121.2 | 363.0 |
| IL18R1 | interleukin 18 receptor 1 | [8809](https://www.ncbi.nlm.nih.gov/gene/?term=8809) | 2.6 | 0.00005 | 1780.8 | 626.6 |
| LOC105376991 |  | [105376991](https://www.ncbi.nlm.nih.gov/gene/?term=105376991) | -2.6 | 0.00169 | 32.1 | 100.0 |
| EIF1B | eukaryotic translation initiation factor 1B | [10289](https://www.ncbi.nlm.nih.gov/gene/?term=10289) | -2.6 | 0.00001 | 2063.5 | 5797.1 |
| PLIN4 | perilipin 4 | [729359](https://www.ncbi.nlm.nih.gov/gene/?term=729359) | 2.6 | 0.00083 | 176.6 | 59.0 |
| LOC102724517 |  | [102724517](https://www.ncbi.nlm.nih.gov/gene/?term=102724517) | -2.6 | 0.00001 | 68.0 | 191.4 |
| BRE-AS1 | BRE antisense RNA 1 | [100302650](https://www.ncbi.nlm.nih.gov/gene/?term=100302650) | 2.6 | 0.00015 | 20.3 | 7.4 |
| LINC00659 | long intergenic non-protein coding RNA 659 | [100652730](https://www.ncbi.nlm.nih.gov/gene/?term=100652730) | 2.6 | 0.00378 | 9.7 | 3.1 |
| MMP24-AS1 | MMP24 antisense RNA 1 | [101410538](https://www.ncbi.nlm.nih.gov/gene/?term=101410538) | -2.6 | 0.00002 | 75.6 | 210.6 |
| LOC107986428 |  | [107986428](https://www.ncbi.nlm.nih.gov/gene/?term=107986428) | -2.6 | 0.00541 | 3.4 | 11.9 |
| LOC107987297 |  | [107987297](https://www.ncbi.nlm.nih.gov/gene/?term=107987297) | -2.6 | 0.00130 | 3.8 | 12.5 |
| STRAP | serine/threonine kinase receptor associated protein | [11171](https://www.ncbi.nlm.nih.gov/gene/?term=11171) | -2.6 | 0.00000 | 605.3 | 1648.4 |
| VPS9D1 | VPS9 domain containing 1 | [9605](https://www.ncbi.nlm.nih.gov/gene/?term=9605) | 2.6 | 0.00001 | 513.1 | 185.5 |
| PLBD1 | phospholipase B domain containing 1 | [79887](https://www.ncbi.nlm.nih.gov/gene/?term=79887) | 2.6 | 0.00001 | 9585.4 | 3464.5 |
| SCN2A | sodium voltage-gated channel alpha subunit 2 | [6326](https://www.ncbi.nlm.nih.gov/gene/?term=6326) | -2.6 | 0.01622 | 1.6 | 6.6 |
| MYL4 | myosin light chain 4 | [4635](https://www.ncbi.nlm.nih.gov/gene/?term=4635) | -2.6 | 0.00115 | 861.4 | 2577.9 |
| ANKRD44-IT1 | ANKRD44 intronic transcript 1 | [101927547](https://www.ncbi.nlm.nih.gov/gene/?term=101927547) | -2.6 | 0.00069 | 139.2 | 412.3 |
| RPL5P4 | ribosomal protein L5 pseudogene 4 | [643343](https://www.ncbi.nlm.nih.gov/gene/?term=643343) | -2.6 | 0.00019 | 6.0 | 18.5 |
| LINC00597 | long intergenic non-protein coding RNA 597 | [81698](https://www.ncbi.nlm.nih.gov/gene/?term=81698) | -2.6 | 0.00585 | 6.3 | 21.4 |
| CASP4 | caspase 4 | [837](https://www.ncbi.nlm.nih.gov/gene/?term=837) | 2.6 | 0.00000 | 11894.7 | 4411.8 |
| KIAA1161 | KIAA1161 | [57462](https://www.ncbi.nlm.nih.gov/gene/?term=57462) | -2.6 | 0.00033 | 4.9 | 14.5 |
| CES1 | carboxylesterase 1 | [1066](https://www.ncbi.nlm.nih.gov/gene/?term=1066) | 2.6 | 0.00232 | 56.6 | 18.3 |
| LOC105371637 |  | [105371637](https://www.ncbi.nlm.nih.gov/gene/?term=105371637) | -2.6 | 0.00018 | 14.1 | 41.3 |
| LOC105379426 |  | [105379426](https://www.ncbi.nlm.nih.gov/gene/?term=105379426) | -2.6 | 0.01618 | 8.7 | 34.3 |
| SPATA22 | spermatogenesis associated 22 | [84690](https://www.ncbi.nlm.nih.gov/gene/?term=84690) | -2.6 | 0.01830 | 3.0 | 12.4 |
| LOC105373774 |  | [105373774](https://www.ncbi.nlm.nih.gov/gene/?term=105373774) | -2.6 | 0.00093 | 16.3 | 49.6 |
| C3AR1 | complement component 3a receptor 1 | [719](https://www.ncbi.nlm.nih.gov/gene/?term=719) | 2.6 | 0.00006 | 731.9 | 262.2 |
| BST2 | bone marrow stromal cell antigen 2 | [684](https://www.ncbi.nlm.nih.gov/gene/?term=684) | 2.5 | 0.00000 | 2141.3 | 789.7 |
| MIR1200 | microRNA 1200 | [100302113](https://www.ncbi.nlm.nih.gov/gene/?term=100302113) | -2.5 | 0.00515 | 3.9 | 13.4 |
| SESN3 | sestrin 3 | [143686](https://www.ncbi.nlm.nih.gov/gene/?term=143686) | -2.5 | 0.00038 | 36256.5 | 108027.9 |
| POLL | polymerase (DNA) lambda | [27343](https://www.ncbi.nlm.nih.gov/gene/?term=27343) | -2.5 | 0.00018 | 887.1 | 2512.8 |
| MARCH6 | membrane associated ring-CH-type finger 6 | [10299](https://www.ncbi.nlm.nih.gov/gene/?term=10299) | -2.5 | 0.00000 | 5273.1 | 14418.9 |
| LOC105372964 |  | [105372964](https://www.ncbi.nlm.nih.gov/gene/?term=105372964) | -2.5 | 0.00118 | 37.3 | 112.0 |
| GGT1 | gamma-glutamyltransferase 1 | [2678](https://www.ncbi.nlm.nih.gov/gene/?term=2678) | 2.5 | 0.00000 | 366.6 | 137.0 |
| OAS2 | 2'-5'-oligoadenylate synthetase 2 | [4939](https://www.ncbi.nlm.nih.gov/gene/?term=4939) | 2.5 | 0.00162 | 10908.2 | 3677.8 |
| LOC107986187 |  | [107986187](https://www.ncbi.nlm.nih.gov/gene/?term=107986187) | -2.5 | 0.00027 | 11.4 | 33.2 |
| SCGN | secretagogin, EF-hand calcium binding protein | [10590](https://www.ncbi.nlm.nih.gov/gene/?term=10590) | -2.5 | 0.01069 | 4.4 | 13.8 |
| THBS4 | thrombospondin 4 | [7060](https://www.ncbi.nlm.nih.gov/gene/?term=7060) | -2.5 | 0.00059 | 5.3 | 16.2 |
| SYNPO | synaptopodin | [11346](https://www.ncbi.nlm.nih.gov/gene/?term=11346) | -2.5 | 0.00306 | 9.5 | 30.1 |
| BATF2 | basic leucine zipper ATF-like transcription factor 2 | [116071](https://www.ncbi.nlm.nih.gov/gene/?term=116071) | 2.5 | 0.01181 | 63.6 | 18.1 |
| LOC105376544 |  | [105376544](https://www.ncbi.nlm.nih.gov/gene/?term=105376544) | 2.5 | 0.00008 | 82.6 | 30.2 |
| HOMER3 | homer scaffolding protein 3 | [9454](https://www.ncbi.nlm.nih.gov/gene/?term=9454) | 2.5 | 0.00000 | 330.9 | 122.8 |
| LRG1 | leucine-rich alpha-2-glycoprotein 1 | [116844](https://www.ncbi.nlm.nih.gov/gene/?term=116844) | 2.5 | 0.00027 | 2443.5 | 863.1 |
| LOC105377449 |  | [105377449](https://www.ncbi.nlm.nih.gov/gene/?term=105377449) | 2.5 | 0.00002 | 94.7 | 35.0 |
| LOC107985366 |  | [107985366](https://www.ncbi.nlm.nih.gov/gene/?term=107985366) | 2.5 | 0.00003 | 50.4 | 18.6 |
| NAV3 | neuron navigator 3 | [89795](https://www.ncbi.nlm.nih.gov/gene/?term=89795) | -2.5 | 0.00327 | 10.8 | 33.0 |
| HAUS4 | HAUS augmin like complex subunit 4 | [54930](https://www.ncbi.nlm.nih.gov/gene/?term=54930) | 2.5 | 0.00001 | 2161.5 | 803.2 |
| TNNT1 | troponin T1, slow skeletal type | [7138](https://www.ncbi.nlm.nih.gov/gene/?term=7138) | 2.5 | 0.00395 | 146.2 | 47.2 |
| TDRD7 | tudor domain containing 7 | [23424](https://www.ncbi.nlm.nih.gov/gene/?term=23424) | 2.5 | 0.00000 | 2799.0 | 1044.0 |
| MIR630 | microRNA 630 | [693215](https://www.ncbi.nlm.nih.gov/gene/?term=693215) | -2.5 | 0.00003 | 17.3 | 48.0 |
| CA4 | carbonic anhydrase 4 | [762](https://www.ncbi.nlm.nih.gov/gene/?term=762) | 2.5 | 0.00077 | 416.4 | 145.1 |
| LOC107985597 |  | [107985597](https://www.ncbi.nlm.nih.gov/gene/?term=107985597) | -2.5 | 0.00015 | 10.8 | 31.7 |
| LOC105371242 |  | [105371242](https://www.ncbi.nlm.nih.gov/gene/?term=105371242) | -2.5 | 0.00888 | 5.2 | 17.7 |
| NRADDP | neurotrophin receptor associated death domain, pseudogene | [100129354](https://www.ncbi.nlm.nih.gov/gene/?term=100129354) | 2.5 | 0.00147 | 22.8 | 8.2 |
| FAT4 | FAT atypical cadherin 4 | [79633](https://www.ncbi.nlm.nih.gov/gene/?term=79633) | -2.5 | 0.00342 | 19.9 | 61.3 |
| APOL2 | apolipoprotein L2 | [23780](https://www.ncbi.nlm.nih.gov/gene/?term=23780) | 2.5 | 0.00000 | 3491.1 | 1326.4 |
| GRINA | glutamate ionotropic receptor NMDA type subunit associated protein 1 | [2907](https://www.ncbi.nlm.nih.gov/gene/?term=2907) | -2.5 | 0.00025 | 1974.9 | 5544.5 |
| ZNF781 | zinc finger protein 781 | [163115](https://www.ncbi.nlm.nih.gov/gene/?term=163115) | -2.5 | 0.00001 | 27.5 | 74.5 |
| LOC107986344 |  | [107986344](https://www.ncbi.nlm.nih.gov/gene/?term=107986344) | -2.5 | 0.02501 | 1.6 | 8.9 |
| CTSL | cathepsin L | [1514](https://www.ncbi.nlm.nih.gov/gene/?term=1514) | 2.5 | 0.00000 | 889.6 | 335.4 |
| NAIP | NLR family, apoptosis inhibitory protein | [4671](https://www.ncbi.nlm.nih.gov/gene/?term=4671) | 2.5 | 0.01452 | 102.7 | 28.5 |
| LOC100507387 | uncharacterized LOC100507387 | [100507387](https://www.ncbi.nlm.nih.gov/gene/?term=100507387) | -2.5 | 0.00146 | 8.2 | 23.4 |
| IL1RN | interleukin 1 receptor antagonist | [3557](https://www.ncbi.nlm.nih.gov/gene/?term=3557) | 2.5 | 0.00006 | 1319.3 | 483.6 |
| AOC3 | amine oxidase, copper containing 3 | [8639](https://www.ncbi.nlm.nih.gov/gene/?term=8639) | -2.5 | 0.00264 | 16.4 | 50.7 |
| UNC93B1 | unc-93 homolog B1 (C. elegans) | [81622](https://www.ncbi.nlm.nih.gov/gene/?term=81622) | 2.5 | 0.00000 | 3688.0 | 1418.7 |
| MAP1LC3B | microtubule associated protein 1 light chain 3 beta | [81631](https://www.ncbi.nlm.nih.gov/gene/?term=81631) | -2.5 | 0.00000 | 4265.4 | 11319.7 |
| TMEM150B | transmembrane protein 150B | [284417](https://www.ncbi.nlm.nih.gov/gene/?term=284417) | 2.5 | 0.00006 | 110.4 | 40.4 |
| LY96 | lymphocyte antigen 96 | [23643](https://www.ncbi.nlm.nih.gov/gene/?term=23643) | 2.5 | 0.00001 | 2114.6 | 791.9 |
| NTNG2 | netrin G2 | [84628](https://www.ncbi.nlm.nih.gov/gene/?term=84628) | 2.5 | 0.00014 | 802.8 | 292.9 |
| IFITM2 | interferon induced transmembrane protein 2 | [10581](https://www.ncbi.nlm.nih.gov/gene/?term=10581) | 2.5 | 0.00002 | 14760.4 | 5512.4 |
| LOC105374968 |  | [105374968](https://www.ncbi.nlm.nih.gov/gene/?term=105374968) | -2.5 | 0.00064 | 15.6 | 45.2 |
| TBKBP1 | TBK1 binding protein 1 | [9755](https://www.ncbi.nlm.nih.gov/gene/?term=9755) | 2.5 | 0.00004 | 206.9 | 76.9 |
| LOC107984120 |  | [107984120](https://www.ncbi.nlm.nih.gov/gene/?term=107984120) | -2.5 | 0.00089 | 76.7 | 220.9 |
| LOC101928788 |  | [101928788](https://www.ncbi.nlm.nih.gov/gene/?term=101928788) | -2.5 | 0.00465 | 4.5 | 12.7 |
| SECTM1 | secreted and transmembrane 1 | [6398](https://www.ncbi.nlm.nih.gov/gene/?term=6398) | 2.5 | 0.00001 | 2573.3 | 977.7 |
| PCBP2 | poly(rC) binding protein 2 | [5094](https://www.ncbi.nlm.nih.gov/gene/?term=5094) | -2.5 | 0.00002 | 5978.0 | 16054.7 |
| FFAR2 | free fatty acid receptor 2 | [2867](https://www.ncbi.nlm.nih.gov/gene/?term=2867) | 2.5 | 0.00005 | 5009.9 | 1860.2 |
| DRC1 | dynein regulatory complex subunit 1 | [92749](https://www.ncbi.nlm.nih.gov/gene/?term=92749) | 2.5 | 0.00264 | 32.5 | 10.9 |
| RUSC1-AS1 | RUSC1 antisense RNA 1 | [284618](https://www.ncbi.nlm.nih.gov/gene/?term=284618) | 2.5 | 0.00010 | 25.4 | 8.8 |
| NLRC4 | NLR family, CARD domain containing 4 | [58484](https://www.ncbi.nlm.nih.gov/gene/?term=58484) | 2.5 | 0.00001 | 2743.0 | 1036.7 |
| LMO2 | LIM domain only 2 | [4005](https://www.ncbi.nlm.nih.gov/gene/?term=4005) | 2.5 | 0.00000 | 3296.6 | 1281.1 |
| ALDH5A1 | aldehyde dehydrogenase 5 family member A1 | [7915](https://www.ncbi.nlm.nih.gov/gene/?term=7915) | -2.5 | 0.00009 | 3569.1 | 9743.5 |
| MIR3140 | microRNA 3140 | [100422896](https://www.ncbi.nlm.nih.gov/gene/?term=100422896) | -2.5 | 0.00626 | 18.3 | 58.3 |
| PLIN3 | perilipin 3 | [10226](https://www.ncbi.nlm.nih.gov/gene/?term=10226) | 2.5 | 0.00000 | 2150.3 | 829.4 |
| C1GALT1P1 | core 1 synthase, glycoprotein-N-acetylgalactosamine 3-beta-galactosyltransferase 1 pseudogene 1 | [729409](https://www.ncbi.nlm.nih.gov/gene/?term=729409) | -2.5 | 0.00335 | 10.5 | 32.2 |
| TMEM252 | transmembrane protein 252 | [169693](https://www.ncbi.nlm.nih.gov/gene/?term=169693) | 2.5 | 0.00055 | 177.2 | 64.0 |
| OSBP2 | oxysterol binding protein 2 | [23762](https://www.ncbi.nlm.nih.gov/gene/?term=23762) | -2.5 | 0.00259 | 7214.7 | 22648.3 |
| LOC101929984 |  | [101929984](https://www.ncbi.nlm.nih.gov/gene/?term=101929984) | 2.5 | 0.00011 | 364.6 | 135.7 |
| ST6GALNAC4 | ST6 N-acetylgalactosaminide alpha-2,6-sialyltransferase 4 | [27090](https://www.ncbi.nlm.nih.gov/gene/?term=27090) | -2.5 | 0.00077 | 683.9 | 1921.6 |
| CEBPB | CCAAT/enhancer binding protein beta | [1051](https://www.ncbi.nlm.nih.gov/gene/?term=1051) | 2.5 | 0.00003 | 4634.8 | 1746.9 |
| LOC105370513 |  | [105370513](https://www.ncbi.nlm.nih.gov/gene/?term=105370513) | -2.5 | 0.00876 | 25.2 | 82.5 |
| CEBPD | CCAAT/enhancer binding protein delta | [1052](https://www.ncbi.nlm.nih.gov/gene/?term=1052) | 2.5 | 0.00006 | 4257.7 | 1590.3 |
| ALMS1-IT1 | ALMS1 intronic transcript 1 | [100874291](https://www.ncbi.nlm.nih.gov/gene/?term=100874291) | -2.5 | 0.00012 | 14.7 | 40.0 |
| LOC101927522 |  | [101927522](https://www.ncbi.nlm.nih.gov/gene/?term=101927522) | 2.5 | 0.00233 | 156.1 | 53.8 |
| NPM1P44 | nucleophosmin 1 (nucleolar phosphoprotein B23, numatrin) pseudogene 44 | [100422270](https://www.ncbi.nlm.nih.gov/gene/?term=100422270) | -2.5 | 0.01413 | 2.3 | 8.7 |
| LOC107986348 |  | [107986348](https://www.ncbi.nlm.nih.gov/gene/?term=107986348) | -2.5 | 0.00096 | 15.9 | 44.8 |
| PPP1R14BP1 | protein phosphatase 1 regulatory inhibitor subunit 14B pseudogene 1 | [50516](https://www.ncbi.nlm.nih.gov/gene/?term=50516) | 2.5 | 0.00897 | 7.7 | 2.3 |
| CAPN5 | calpain 5 | [726](https://www.ncbi.nlm.nih.gov/gene/?term=726) | -2.5 | 0.00054 | 117.0 | 323.1 |
| NOMO1 | NODAL modulator 1 | [23420](https://www.ncbi.nlm.nih.gov/gene/?term=23420) | -2.5 | 0.00769 | 5.6 | 18.4 |
| LOC105373802 |  | [105373802](https://www.ncbi.nlm.nih.gov/gene/?term=105373802) | -2.5 | 0.00050 | 6.5 | 18.4 |
| LEP | leptin | [3952](https://www.ncbi.nlm.nih.gov/gene/?term=3952) | 2.5 | 0.01171 | 11.7 | 3.6 |
| LOC103156999 | dynein light chain Tctex-type 1 pseudogene | [103156999](https://www.ncbi.nlm.nih.gov/gene/?term=103156999) | -2.5 | 0.00043 | 15.0 | 42.6 |
| PALLD | palladin, cytoskeletal associated protein | [23022](https://www.ncbi.nlm.nih.gov/gene/?term=23022) | -2.4 | 0.00400 | 151.3 | 454.3 |
| RPL17P36 | ribosomal protein L17 pseudogene 36 | [729340](https://www.ncbi.nlm.nih.gov/gene/?term=729340) | -2.4 | 0.00977 | 1.4 | 3.8 |
| FGFR2 | fibroblast growth factor receptor 2 | [2263](https://www.ncbi.nlm.nih.gov/gene/?term=2263) | -2.4 | 0.00415 | 12.1 | 35.8 |
| DDIT3 | DNA damage inducible transcript 3 | [1649](https://www.ncbi.nlm.nih.gov/gene/?term=1649) | 2.4 | 0.00000 | 1001.7 | 386.1 |
| FUT7 | fucosyltransferase 7 | [2529](https://www.ncbi.nlm.nih.gov/gene/?term=2529) | 2.4 | 0.00004 | 1190.2 | 450.6 |
| LOC105378819 |  | [105378819](https://www.ncbi.nlm.nih.gov/gene/?term=105378819) | 2.4 | 0.00011 | 138.4 | 51.6 |
| LOC107985288 |  | [107985288](https://www.ncbi.nlm.nih.gov/gene/?term=107985288) | 2.4 | 0.00798 | 10.8 | 3.6 |
| NBEA | neurobeachin | [26960](https://www.ncbi.nlm.nih.gov/gene/?term=26960) | -2.4 | 0.00000 | 134.5 | 347.5 |
| FAM66B | family with sequence similarity 66 member B | [100128890](https://www.ncbi.nlm.nih.gov/gene/?term=100128890) | -2.4 | 0.00602 | 3.6 | 11.8 |
| LOC102725228 |  | [102725228](https://www.ncbi.nlm.nih.gov/gene/?term=102725228) | 2.4 | 0.00000 | 103.8 | 40.7 |
| TMEM245 | transmembrane protein 245 | [23731](https://www.ncbi.nlm.nih.gov/gene/?term=23731) | -2.4 | 0.00000 | 2240.5 | 5774.3 |
| LOC105375924 |  | [105375924](https://www.ncbi.nlm.nih.gov/gene/?term=105375924) | 2.4 | 0.00386 | 84.8 | 29.2 |
| LOC100507507 | uncharacterized LOC100507507 | [100507507](https://www.ncbi.nlm.nih.gov/gene/?term=100507507) | 2.4 | 0.00000 | 353.8 | 138.8 |
| SAT1 | spermidine/spermine N1-acetyltransferase 1 | [6303](https://www.ncbi.nlm.nih.gov/gene/?term=6303) | 2.4 | 0.00000 | 18804.7 | 7392.1 |
| IFIT5 | interferon induced protein with tetratricopeptide repeats 5 | [24138](https://www.ncbi.nlm.nih.gov/gene/?term=24138) | 2.4 | 0.00015 | 6691.8 | 2502.8 |
| KANK2 | KN motif and ankyrin repeat domains 2 | [25959](https://www.ncbi.nlm.nih.gov/gene/?term=25959) | -2.4 | 0.00436 | 870.3 | 2598.4 |
| IRF2BPL | interferon regulatory factor 2 binding protein like | [64207](https://www.ncbi.nlm.nih.gov/gene/?term=64207) | 2.4 | 0.00003 | 1302.0 | 499.2 |
| LINC00997 | long intergenic non-protein coding RNA 997 | [401321](https://www.ncbi.nlm.nih.gov/gene/?term=401321) | -2.4 | 0.00469 | 15.2 | 44.8 |
| SQRDL | sulfide quinone reductase-like (yeast) | [58472](https://www.ncbi.nlm.nih.gov/gene/?term=58472) | 2.4 | 0.00000 | 6102.4 | 2422.0 |
| LOC102725082 |  | [102725082](https://www.ncbi.nlm.nih.gov/gene/?term=102725082) | -2.4 | 0.00292 | 26.9 | 79.1 |
| RPS27AP12 | ribosomal protein S27a pseudogene 12 | [100130446](https://www.ncbi.nlm.nih.gov/gene/?term=100130446) | -2.4 | 0.00814 | 14.2 | 45.5 |
| LOC105375402 |  | [105375402](https://www.ncbi.nlm.nih.gov/gene/?term=105375402) | -2.4 | 0.00190 | 4.7 | 11.5 |
| ACKR2 | atypical chemokine receptor 2 | [1238](https://www.ncbi.nlm.nih.gov/gene/?term=1238) | -2.4 | 0.00096 | 24.3 | 64.6 |
| LOC107984788 |  | [107984788](https://www.ncbi.nlm.nih.gov/gene/?term=107984788) | 2.4 | 0.00340 | 23.5 | 8.2 |
| NFIX | nuclear factor I X | [4784](https://www.ncbi.nlm.nih.gov/gene/?term=4784) | -2.4 | 0.00110 | 697.5 | 1950.0 |
| LOC105378020 |  | [105378020](https://www.ncbi.nlm.nih.gov/gene/?term=105378020) | 2.4 | 0.01402 | 23.4 | 6.9 |
| OR2W3 | olfactory receptor family 2 subfamily W member 3 | [343171](https://www.ncbi.nlm.nih.gov/gene/?term=343171) | -2.4 | 0.01183 | 875.2 | 2885.0 |
| CLEC4G | C-type lectin domain family 4 member G | [339390](https://www.ncbi.nlm.nih.gov/gene/?term=339390) | 2.4 | 0.00685 | 17.6 | 5.5 |
| CCR1 | C-C motif chemokine receptor 1 | [1230](https://www.ncbi.nlm.nih.gov/gene/?term=1230) | 2.4 | 0.00027 | 5094.4 | 1901.0 |
| LOC102724231 | uncharacterized LOC102724231 | [102724231](https://www.ncbi.nlm.nih.gov/gene/?term=102724231) | 2.4 | 0.00121 | 120.4 | 43.6 |
| LOC107986755 |  | [107986755](https://www.ncbi.nlm.nih.gov/gene/?term=107986755) | -2.4 | 0.01048 | 9.3 | 29.3 |
| LOC101929964 |  | [101929964](https://www.ncbi.nlm.nih.gov/gene/?term=101929964) | -2.4 | 0.00576 | 7.2 | 22.5 |
| MILR1 | mast cell immunoglobulin like receptor 1 | [284021](https://www.ncbi.nlm.nih.gov/gene/?term=284021) | 2.4 | 0.00000 | 825.7 | 325.6 |
| ATP1B2 | ATPase Na+/K+ transporting subunit beta 2 | [482](https://www.ncbi.nlm.nih.gov/gene/?term=482) | -2.4 | 0.00133 | 27.2 | 75.5 |
| MYBL1 | MYB proto-oncogene like 1 | [4603](https://www.ncbi.nlm.nih.gov/gene/?term=4603) | -2.4 | 0.00001 | 364.0 | 934.9 |
| IFI30 | IFI30, lysosomal thiol reductase | [10437](https://www.ncbi.nlm.nih.gov/gene/?term=10437) | 2.4 | 0.00000 | 15293.6 | 6088.8 |
| IFNG-AS1 | IFNG antisense RNA 1 | [100885789](https://www.ncbi.nlm.nih.gov/gene/?term=100885789) | -2.4 | 0.00012 | 81.9 | 216.9 |
| MAP7 | microtubule associated protein 7 | [9053](https://www.ncbi.nlm.nih.gov/gene/?term=9053) | -2.4 | 0.00001 | 51.2 | 131.8 |
| ADGRE3 | adhesion G protein-coupled receptor E3 | [84658](https://www.ncbi.nlm.nih.gov/gene/?term=84658) | -2.4 | 0.00000 | 733.0 | 1852.0 |
| LOC101926994 |  | [101926994](https://www.ncbi.nlm.nih.gov/gene/?term=101926994) | 2.4 | 0.00152 | 48.5 | 17.8 |
| MIRLET7F1 | microRNA let-7f-1 | [406888](https://www.ncbi.nlm.nih.gov/gene/?term=406888) | -2.4 | 0.00109 | 5.2 | 15.4 |
| SEPT14P19 | septin 14 pseudogene 19 | [107105271](https://www.ncbi.nlm.nih.gov/gene/?term=107105271) | 2.4 | 0.00735 | 12.5 | 4.3 |
| LOC100421438 | DnaJ heat shock protein family (Hsp40) member C8 pseudogene | [100421438](https://www.ncbi.nlm.nih.gov/gene/?term=100421438) | -2.4 | 0.00402 | 8.4 | 25.4 |
| LOC107984880 |  | [107984880](https://www.ncbi.nlm.nih.gov/gene/?term=107984880) | 2.4 | 0.00253 | 20.0 | 7.4 |
| TPT1P10 |  | [101928715](https://www.ncbi.nlm.nih.gov/gene/?term=101928715) | -2.4 | 0.00102 | 22.6 | 62.6 |
| EIF1 | eukaryotic translation initiation factor 1 | [10209](https://www.ncbi.nlm.nih.gov/gene/?term=10209) | -2.4 | 0.00002 | 4697.5 | 12186.1 |
| SLC16A3 | solute carrier family 16 member 3 | [9123](https://www.ncbi.nlm.nih.gov/gene/?term=9123) | 2.4 | 0.00000 | 5663.8 | 2257.5 |
| YOD1 | YOD1 deubiquitinase | [55432](https://www.ncbi.nlm.nih.gov/gene/?term=55432) | -2.4 | 0.00181 | 89311.6 | 248941.0 |
| TRAV24 | T cell receptor alpha variable 24 | [28659](https://www.ncbi.nlm.nih.gov/gene/?term=28659) | 2.4 | 0.00338 | 30.5 | 10.8 |
| PSTPIP2 | proline-serine-threonine phosphatase interacting protein 2 | [9050](https://www.ncbi.nlm.nih.gov/gene/?term=9050) | 2.4 | 0.00013 | 2714.6 | 1039.5 |
| RNF11 | ring finger protein 11 | [26994](https://www.ncbi.nlm.nih.gov/gene/?term=26994) | -2.4 | 0.00070 | 25651.3 | 69113.2 |
| LOC107985522 |  | [107985522](https://www.ncbi.nlm.nih.gov/gene/?term=107985522) | -2.4 | 0.00175 | 16.0 | 45.1 |
| ZNF395 | zinc finger protein 395 | [55893](https://www.ncbi.nlm.nih.gov/gene/?term=55893) | -2.4 | 0.00001 | 94.9 | 242.4 |
| CENPM | centromere protein M | [79019](https://www.ncbi.nlm.nih.gov/gene/?term=79019) | 2.4 | 0.00105 | 42.3 | 15.7 |
| DYNLT1 | dynein light chain Tctex-type 1 | [6993](https://www.ncbi.nlm.nih.gov/gene/?term=6993) | 2.4 | 0.00000 | 2384.7 | 951.7 |
| PNPLA2 | patatin like phospholipase domain containing 2 | [57104](https://www.ncbi.nlm.nih.gov/gene/?term=57104) | -2.4 | 0.00005 | 935.8 | 2397.2 |
| ZNF787 | zinc finger protein 787 | [126208](https://www.ncbi.nlm.nih.gov/gene/?term=126208) | 2.4 | 0.00000 | 952.4 | 386.0 |
| RRM2P3 | ribonucleotide reductase M2 polypeptide pseudogene 3 | [100420159](https://www.ncbi.nlm.nih.gov/gene/?term=100420159) | -2.4 | 0.00003 | 35.6 | 88.6 |
| RETN | resistin | [56729](https://www.ncbi.nlm.nih.gov/gene/?term=56729) | 2.4 | 0.01501 | 292.1 | 88.3 |
| LIMK2 | LIM domain kinase 2 | [3985](https://www.ncbi.nlm.nih.gov/gene/?term=3985) | 2.4 | 0.00007 | 4120.3 | 1611.6 |
| RPS15AP10 | ribosomal protein S15a pseudogene 10 | [728963](https://www.ncbi.nlm.nih.gov/gene/?term=728963) | -2.4 | 0.00251 | 23.8 | 67.1 |
| KIAA0895L | KIAA0895-like | [653319](https://www.ncbi.nlm.nih.gov/gene/?term=653319) | 2.4 | 0.00003 | 54.4 | 21.1 |
| LOC105375035 |  | [105375035](https://www.ncbi.nlm.nih.gov/gene/?term=105375035) | 2.4 | 0.00418 | 22.1 | 8.1 |
| ATP1B1P1 | ATPase Na+/K+ transporting subunit beta 1 pseudogene 1 | [485](https://www.ncbi.nlm.nih.gov/gene/?term=485) | -2.4 | 0.00232 | 13.3 | 37.7 |
| TRT-CGT2-1 |  | [100189086](https://www.ncbi.nlm.nih.gov/gene/?term=100189086) | 2.4 | 0.00956 | 7.4 | 2.2 |
| KCND3 | potassium voltage-gated channel subfamily D member 3 | [3752](https://www.ncbi.nlm.nih.gov/gene/?term=3752) | -2.4 | 0.00199 | 18.8 | 51.7 |
| DYNC2H1 | dynein cytoplasmic 2 heavy chain 1 | [79659](https://www.ncbi.nlm.nih.gov/gene/?term=79659) | -2.4 | 0.00001 | 72.9 | 184.2 |
| LOC107984570 |  | [107984570](https://www.ncbi.nlm.nih.gov/gene/?term=107984570) | -2.4 | 0.00188 | 8.9 | 25.0 |
| ASGR1 | asialoglycoprotein receptor 1 | [432](https://www.ncbi.nlm.nih.gov/gene/?term=432) | 2.4 | 0.00000 | 316.2 | 126.3 |
| MB21D1 | Mab-21 domain containing 1 | [115004](https://www.ncbi.nlm.nih.gov/gene/?term=115004) | 2.4 | 0.00000 | 1055.7 | 427.1 |
| HSPA6 | heat shock protein family A (Hsp70) member 6 | [3310](https://www.ncbi.nlm.nih.gov/gene/?term=3310) | 2.3 | 0.00001 | 3602.3 | 1437.4 |
| ADGRE4P | adhesion G protein-coupled receptor E4, pseudogene | [326342](https://www.ncbi.nlm.nih.gov/gene/?term=326342) | -2.3 | 0.00327 | 72.0 | 202.2 |
| LOC100996741 |  | [100996741](https://www.ncbi.nlm.nih.gov/gene/?term=100996741) | -2.3 | 0.00017 | 91.0 | 235.3 |
| B4GALT5 | beta-1,4-galactosyltransferase 5 | [9334](https://www.ncbi.nlm.nih.gov/gene/?term=9334) | 2.3 | 0.00001 | 5679.0 | 2275.6 |
| GPX3 | glutathione peroxidase 3 | [2878](https://www.ncbi.nlm.nih.gov/gene/?term=2878) | -2.3 | 0.00617 | 4.3 | 10.9 |
| LGSN | lengsin, lens protein with glutamine synthetase domain | [51557](https://www.ncbi.nlm.nih.gov/gene/?term=51557) | 2.3 | 0.01163 | 51.0 | 16.6 |
| RAB24 | RAB24, member RAS oncogene family | [53917](https://www.ncbi.nlm.nih.gov/gene/?term=53917) | 2.3 | 0.00000 | 3372.1 | 1380.3 |
| LOC105378448 |  | [105378448](https://www.ncbi.nlm.nih.gov/gene/?term=105378448) | 2.3 | 0.00068 | 25.9 | 9.9 |
| TAS2R43 | taste 2 receptor member 43 | [259289](https://www.ncbi.nlm.nih.gov/gene/?term=259289) | -2.3 | 0.01798 | 3.4 | 11.8 |
| LOC107984617 |  | [107984617](https://www.ncbi.nlm.nih.gov/gene/?term=107984617) | -2.3 | 0.00008 | 26.8 | 70.2 |
| CEP55 | centrosomal protein 55 | [55165](https://www.ncbi.nlm.nih.gov/gene/?term=55165) | 2.3 | 0.00269 | 162.1 | 59.1 |
| LOC100128276 |  | [100128276](https://www.ncbi.nlm.nih.gov/gene/?term=100128276) | 2.3 | 0.00007 | 237.0 | 94.0 |
| PLIN5 | perilipin 5 | [440503](https://www.ncbi.nlm.nih.gov/gene/?term=440503) | 2.3 | 0.00031 | 133.4 | 52.0 |
| LOC107984258 |  | [107984258](https://www.ncbi.nlm.nih.gov/gene/?term=107984258) | -2.3 | 0.01031 | 4.5 | 14.2 |
| KCNJ2 | potassium voltage-gated channel subfamily J member 2 | [3759](https://www.ncbi.nlm.nih.gov/gene/?term=3759) | 2.3 | 0.00004 | 5670.6 | 2283.3 |
| WBP2 | WW domain binding protein 2 | [23558](https://www.ncbi.nlm.nih.gov/gene/?term=23558) | -2.3 | 0.00012 | 1458.0 | 3691.9 |
| NAMPT | nicotinamide phosphoribosyltransferase | [10135](https://www.ncbi.nlm.nih.gov/gene/?term=10135) | 2.3 | 0.00018 | 114882.8 | 44379.7 |
| LOC105369383 |  | [105369383](https://www.ncbi.nlm.nih.gov/gene/?term=105369383) | -2.3 | 0.00374 | 8.4 | 24.4 |
| NAT6 | N-acetyltransferase 6 | [24142](https://www.ncbi.nlm.nih.gov/gene/?term=24142) | -2.3 | 0.00320 | 3.5 | 10.3 |
| LOC105378560 |  | [105378560](https://www.ncbi.nlm.nih.gov/gene/?term=105378560) | -2.3 | 0.00385 | 13.2 | 38.4 |
| BLVRA | biliverdin reductase A | [644](https://www.ncbi.nlm.nih.gov/gene/?term=644) | 2.3 | 0.00000 | 793.2 | 327.8 |
| LOC105374102 |  | [105374102](https://www.ncbi.nlm.nih.gov/gene/?term=105374102) | 2.3 | 0.00041 | 293.9 | 113.9 |
| LOC100421523 | UDP-N-acetylglucosamine pyrophosphorylase 1 pseudogene | [100421523](https://www.ncbi.nlm.nih.gov/gene/?term=100421523) | -2.3 | 0.01037 | 9.4 | 29.3 |
| LOC101926963 | uncharacterized LOC101926963 | [101926963](https://www.ncbi.nlm.nih.gov/gene/?term=101926963) | 2.3 | 0.00000 | 95.6 | 38.5 |
| KREMEN1 | kringle containing transmembrane protein 1 | [83999](https://www.ncbi.nlm.nih.gov/gene/?term=83999) | 2.3 | 0.00035 | 1052.0 | 409.1 |
| SEMA5A | semaphorin 5A | [9037](https://www.ncbi.nlm.nih.gov/gene/?term=9037) | -2.3 | 0.00866 | 13.5 | 40.0 |
| RGPD4 | RANBP2-like and GRIP domain containing 4 | [285190](https://www.ncbi.nlm.nih.gov/gene/?term=285190) | -2.3 | 0.01645 | 4.2 | 14.2 |
| PPP1R3B | protein phosphatase 1 regulatory subunit 3B | [79660](https://www.ncbi.nlm.nih.gov/gene/?term=79660) | 2.3 | 0.00009 | 6567.6 | 2609.6 |
| LOC107985303 |  | [107985303](https://www.ncbi.nlm.nih.gov/gene/?term=107985303) | 2.3 | 0.00016 | 24.8 | 9.7 |
| ETS2 | ETS proto-oncogene 2, transcription factor | [2114](https://www.ncbi.nlm.nih.gov/gene/?term=2114) | 2.3 | 0.00002 | 3137.5 | 1275.6 |
| H3F3C | H3 histone, family 3C | [440093](https://www.ncbi.nlm.nih.gov/gene/?term=440093) | 2.3 | 0.00036 | 15.5 | 6.0 |
| GALK1 | galactokinase 1 | [2584](https://www.ncbi.nlm.nih.gov/gene/?term=2584) | 2.3 | 0.00000 | 428.6 | 179.0 |
| MRVI1-AS1 | MRVI1 antisense RNA 1 | [100129827](https://www.ncbi.nlm.nih.gov/gene/?term=100129827) | 2.3 | 0.00330 | 61.7 | 22.7 |
| IFITM3 | interferon induced transmembrane protein 3 | [10410](https://www.ncbi.nlm.nih.gov/gene/?term=10410) | 2.3 | 0.00397 | 3650.1 | 1321.1 |
| LOC107986422 |  | [107986422](https://www.ncbi.nlm.nih.gov/gene/?term=107986422) | -2.3 | 0.00014 | 33.1 | 84.7 |
| LOC107984023 |  | [107984023](https://www.ncbi.nlm.nih.gov/gene/?term=107984023) | -2.3 | 0.00504 | 3.9 | 10.9 |
| MARCKS | myristoylated alanine rich protein kinase C substrate | [4082](https://www.ncbi.nlm.nih.gov/gene/?term=4082) | 2.3 | 0.00002 | 4259.4 | 1727.4 |
| MIR451A | microRNA 451a | [574411](https://www.ncbi.nlm.nih.gov/gene/?term=574411) | -2.3 | 0.02658 | 1.7 | 7.5 |
| EGFL7 | EGF like domain multiple 7 | [51162](https://www.ncbi.nlm.nih.gov/gene/?term=51162) | 2.3 | 0.00099 | 129.6 | 49.6 |
| SAMSN1 | SAM domain, SH3 domain and nuclear localization signals 1 | [64092](https://www.ncbi.nlm.nih.gov/gene/?term=64092) | 2.3 | 0.00000 | 2471.6 | 1016.2 |
| PIF1 | PIF1 5'-to-3' DNA helicase | [80119](https://www.ncbi.nlm.nih.gov/gene/?term=80119) | 2.3 | 0.00157 | 42.8 | 16.2 |
| LPIN2 | lipin 2 | [9663](https://www.ncbi.nlm.nih.gov/gene/?term=9663) | -2.3 | 0.00005 | 8451.5 | 20935.4 |
| MXD3 | MAX dimerization protein 3 | [83463](https://www.ncbi.nlm.nih.gov/gene/?term=83463) | 2.3 | 0.00001 | 677.1 | 277.7 |
| SLPI | secretory leukocyte peptidase inhibitor | [6590](https://www.ncbi.nlm.nih.gov/gene/?term=6590) | 2.3 | 0.00271 | 464.5 | 173.5 |
| DSG2 | desmoglein 2 | [1829](https://www.ncbi.nlm.nih.gov/gene/?term=1829) | -2.3 | 0.00188 | 4.6 | 11.7 |
| SERPINB1 | serpin family B member 1 | [1992](https://www.ncbi.nlm.nih.gov/gene/?term=1992) | 2.3 | 0.00007 | 7298.9 | 2929.9 |
| SLC22A18 | solute carrier family 22 member 18 | [5002](https://www.ncbi.nlm.nih.gov/gene/?term=5002) | 2.3 | 0.00001 | 52.1 | 21.7 |
| INAFM1 | InaF motif containing 1 | [255783](https://www.ncbi.nlm.nih.gov/gene/?term=255783) | 2.3 | 0.00001 | 75.8 | 30.7 |
| IER5L | immediate early response 5-like | [389792](https://www.ncbi.nlm.nih.gov/gene/?term=389792) | 2.3 | 0.00008 | 248.5 | 99.6 |
| MT1E | metallothionein 1E | [4493](https://www.ncbi.nlm.nih.gov/gene/?term=4493) | 2.3 | 0.00470 | 62.1 | 22.5 |
| DDX58 | DEXD/H-box helicase 58 | [23586](https://www.ncbi.nlm.nih.gov/gene/?term=23586) | 2.3 | 0.00031 | 11275.0 | 4454.7 |
| GPR21 | G protein-coupled receptor 21 | [2844](https://www.ncbi.nlm.nih.gov/gene/?term=2844) | -2.3 | 0.00240 | 115.9 | 309.6 |
| PADI4 | peptidyl arginine deiminase 4 | [23569](https://www.ncbi.nlm.nih.gov/gene/?term=23569) | 2.3 | 0.00960 | 14.1 | 4.8 |
| LOC105375668 |  | [105375668](https://www.ncbi.nlm.nih.gov/gene/?term=105375668) | -2.3 | 0.00016 | 40.1 | 101.6 |
| TRIM21 | tripartite motif containing 21 | [6737](https://www.ncbi.nlm.nih.gov/gene/?term=6737) | 2.3 | 0.00000 | 2588.3 | 1079.8 |
| ATP2C2 | ATPase secretory pathway Ca2+ transporting 2 | [9914](https://www.ncbi.nlm.nih.gov/gene/?term=9914) | 2.3 | 0.01926 | 29.2 | 8.6 |
| JUNB | JunB proto-oncogene, AP-1 transcription factor subunit | [3726](https://www.ncbi.nlm.nih.gov/gene/?term=3726) | 2.3 | 0.00000 | 5572.6 | 2338.9 |
| FTO-IT1 | FTO intronic transcript 1 | [100505692](https://www.ncbi.nlm.nih.gov/gene/?term=100505692) | -2.3 | 0.00451 | 6.6 | 19.1 |
| MSR1 | macrophage scavenger receptor 1 | [4481](https://www.ncbi.nlm.nih.gov/gene/?term=4481) | 2.3 | 0.00270 | 490.0 | 183.2 |
| ATG9A | autophagy related 9A | [79065](https://www.ncbi.nlm.nih.gov/gene/?term=79065) | -2.3 | 0.00030 | 998.6 | 2533.0 |
| EMP1 | epithelial membrane protein 1 | [2012](https://www.ncbi.nlm.nih.gov/gene/?term=2012) | 2.3 | 0.00163 | 104.8 | 40.1 |
| HBG2 | hemoglobin subunit gamma 2 | [3048](https://www.ncbi.nlm.nih.gov/gene/?term=3048) | -2.3 | 0.02237 | 200.8 | 743.3 |
| CYTH4 | cytohesin 4 | [27128](https://www.ncbi.nlm.nih.gov/gene/?term=27128) | 2.3 | 0.00000 | 12929.5 | 5452.5 |
| SAMD4A | sterile alpha motif domain containing 4A | [23034](https://www.ncbi.nlm.nih.gov/gene/?term=23034) | 2.3 | 0.00088 | 288.0 | 111.8 |
| LTB4R | leukotriene B4 receptor | [1241](https://www.ncbi.nlm.nih.gov/gene/?term=1241) | 2.3 | 0.00002 | 1678.0 | 688.6 |
| KCTD9 | potassium channel tetramerization domain containing 9 | [54793](https://www.ncbi.nlm.nih.gov/gene/?term=54793) | -2.3 | 0.00000 | 882.0 | 2125.5 |
| C9orf106 | chromosome 9 open reading frame 106 | [414318](https://www.ncbi.nlm.nih.gov/gene/?term=414318) | 2.3 | 0.00714 | 14.3 | 5.2 |
| EDA | ectodysplasin A | [1896](https://www.ncbi.nlm.nih.gov/gene/?term=1896) | -2.3 | 0.00880 | 6.7 | 19.8 |
| LINC00487 | long intergenic non-protein coding RNA 487 | [400941](https://www.ncbi.nlm.nih.gov/gene/?term=400941) | 2.3 | 0.02980 | 43.0 | 9.4 |
| IL4R | interleukin 4 receptor | [3566](https://www.ncbi.nlm.nih.gov/gene/?term=3566) | 2.3 | 0.00003 | 5850.6 | 2410.0 |
| PABPC3 | poly(A) binding protein cytoplasmic 3 | [5042](https://www.ncbi.nlm.nih.gov/gene/?term=5042) | -2.3 | 0.00280 | 6.0 | 15.2 |
| SMPDL3A | sphingomyelin phosphodiesterase acid like 3A | [10924](https://www.ncbi.nlm.nih.gov/gene/?term=10924) | 2.3 | 0.00009 | 294.1 | 119.5 |
| GABRR2 | gamma-aminobutyric acid type A receptor rho2 subunit | [2570](https://www.ncbi.nlm.nih.gov/gene/?term=2570) | 2.3 | 0.00086 | 153.3 | 60.2 |
| E2F2 | E2F transcription factor 2 | [1870](https://www.ncbi.nlm.nih.gov/gene/?term=1870) | -2.3 | 0.00090 | 19.1 | 47.6 |
| LOC100509370 |  | [100509370](https://www.ncbi.nlm.nih.gov/gene/?term=100509370) | -2.3 | 0.01337 | 15.2 | 47.7 |
| KIT | KIT proto-oncogene receptor tyrosine kinase | [3815](https://www.ncbi.nlm.nih.gov/gene/?term=3815) | -2.3 | 0.00001 | 33.6 | 79.3 |
| SOX5 | SRY-box 5 | [6660](https://www.ncbi.nlm.nih.gov/gene/?term=6660) | -2.3 | 0.00206 | 35.7 | 94.6 |
| HYAL2 | hyaluronoglucosaminidase 2 | [8692](https://www.ncbi.nlm.nih.gov/gene/?term=8692) | 2.3 | 0.00000 | 313.2 | 132.9 |
| RPL21P60 | ribosomal protein L21 pseudogene 60 | [100271175](https://www.ncbi.nlm.nih.gov/gene/?term=100271175) | -2.3 | 0.01033 | 6.1 | 18.6 |
| ABCA1 | ATP binding cassette subfamily A member 1 | [19](https://www.ncbi.nlm.nih.gov/gene/?term=19) | 2.3 | 0.00008 | 8040.3 | 3279.7 |
| LOC105374843 |  | [105374843](https://www.ncbi.nlm.nih.gov/gene/?term=105374843) | 2.3 | 0.02451 | 11.2 | 3.2 |
| HIPK1 | homeodomain interacting protein kinase 1 | [204851](https://www.ncbi.nlm.nih.gov/gene/?term=204851) | -2.3 | 0.00001 | 11155.7 | 27001.9 |
| STAC3 | SH3 and cysteine rich domain 3 | [246329](https://www.ncbi.nlm.nih.gov/gene/?term=246329) | 2.3 | 0.00001 | 163.3 | 67.4 |
| LOC107985568 |  | [107985568](https://www.ncbi.nlm.nih.gov/gene/?term=107985568) | 2.3 | 0.00181 | 13.6 | 5.2 |
| LOC105372055 |  | [105372055](https://www.ncbi.nlm.nih.gov/gene/?term=105372055) | -2.3 | 0.00170 | 19.7 | 51.7 |
| LOC107984598 |  | [107984598](https://www.ncbi.nlm.nih.gov/gene/?term=107984598) | -2.3 | 0.00349 | 9.9 | 27.4 |
| LOC107985544 |  | [107985544](https://www.ncbi.nlm.nih.gov/gene/?term=107985544) | -2.3 | 0.00236 | 6.3 | 16.4 |
| LOC107985356 |  | [107985356](https://www.ncbi.nlm.nih.gov/gene/?term=107985356) | -2.3 | 0.00026 | 15.4 | 38.1 |
| LOC105369299 |  | [105369299](https://www.ncbi.nlm.nih.gov/gene/?term=105369299) | 2.3 | 0.00019 | 25.9 | 10.4 |
| LOC114224 |  | [114224](https://www.ncbi.nlm.nih.gov/gene/?term=114224) | -2.3 | 0.00473 | 23.6 | 65.2 |
| FPR2 | formyl peptide receptor 2 | [2358](https://www.ncbi.nlm.nih.gov/gene/?term=2358) | 2.3 | 0.00019 | 9179.9 | 3733.8 |
| LOC105375942 |  | [105375942](https://www.ncbi.nlm.nih.gov/gene/?term=105375942) | -2.3 | 0.00974 | 7.8 | 23.4 |
| KLHDC7B | kelch domain containing 7B | [113730](https://www.ncbi.nlm.nih.gov/gene/?term=113730) | 2.3 | 0.00062 | 90.6 | 36.1 |
| LRRC34 | leucine rich repeat containing 34 | [151827](https://www.ncbi.nlm.nih.gov/gene/?term=151827) | -2.3 | 0.00004 | 13.3 | 33.3 |
| LOC105375227 |  | [105375227](https://www.ncbi.nlm.nih.gov/gene/?term=105375227) | -2.3 | 0.00301 | 34.9 | 93.1 |
| SERTAD2 | SERTA domain containing 2 | [9792](https://www.ncbi.nlm.nih.gov/gene/?term=9792) | -2.3 | 0.00005 | 574.7 | 1390.9 |
| TNNT3 | troponin T3, fast skeletal type | [7140](https://www.ncbi.nlm.nih.gov/gene/?term=7140) | 2.2 | 0.00055 | 63.7 | 25.7 |
| GNA15 | G protein subunit alpha 15 | [2769](https://www.ncbi.nlm.nih.gov/gene/?term=2769) | 2.2 | 0.00000 | 655.8 | 278.3 |
| EXOSC4 | exosome component 4 | [54512](https://www.ncbi.nlm.nih.gov/gene/?term=54512) | 2.2 | 0.00001 | 271.0 | 113.4 |
| LOC105373148 |  | [105373148](https://www.ncbi.nlm.nih.gov/gene/?term=105373148) | 2.2 | 0.01705 | 40.1 | 13.0 |
| R3HDM4 | R3H domain containing 4 | [91300](https://www.ncbi.nlm.nih.gov/gene/?term=91300) | -2.2 | 0.00035 | 16759.7 | 41506.3 |
| GBP5 | guanylate binding protein 5 | [115362](https://www.ncbi.nlm.nih.gov/gene/?term=115362) | 2.2 | 0.00436 | 23418.4 | 8825.8 |
| RPS3AP34 | ribosomal protein S3a pseudogene 34 | [100271260](https://www.ncbi.nlm.nih.gov/gene/?term=100271260) | -2.2 | 0.02853 | 1.6 | 8.4 |
| HERC6 | HECT and RLD domain containing E3 ubiquitin protein ligase family member 6 | [55008](https://www.ncbi.nlm.nih.gov/gene/?term=55008) | 2.2 | 0.00170 | 1698.9 | 664.2 |
| LOC107985897 |  | [107985897](https://www.ncbi.nlm.nih.gov/gene/?term=107985897) | -2.2 | 0.01201 | 6.3 | 19.4 |
| LOC105374989 |  | [105374989](https://www.ncbi.nlm.nih.gov/gene/?term=105374989) | -2.2 | 0.00117 | 8.1 | 20.9 |
| YPEL4 | yippee like 4 | [219539](https://www.ncbi.nlm.nih.gov/gene/?term=219539) | -2.2 | 0.00115 | 99.9 | 251.9 |
| CD63 | CD63 molecule | [967](https://www.ncbi.nlm.nih.gov/gene/?term=967) | 2.2 | 0.00000 | 3742.6 | 1603.7 |
| LOC105369477 |  | [105369477](https://www.ncbi.nlm.nih.gov/gene/?term=105369477) | -2.2 | 0.00032 | 109.8 | 272.0 |
| DRAP1 | DR1 associated protein 1 | [10589](https://www.ncbi.nlm.nih.gov/gene/?term=10589) | 2.2 | 0.00000 | 980.7 | 425.8 |
| ADAM12 | ADAM metallopeptidase domain 12 | [8038](https://www.ncbi.nlm.nih.gov/gene/?term=8038) | -2.2 | 0.00863 | 11.2 | 32.3 |
| LOC105379250 |  | [105379250](https://www.ncbi.nlm.nih.gov/gene/?term=105379250) | -2.2 | 0.00850 | 11.0 | 31.3 |
| BACH2 | BTB domain and CNC homolog 2 | [60468](https://www.ncbi.nlm.nih.gov/gene/?term=60468) | -2.2 | 0.00014 | 217.8 | 534.4 |
| LOC107987084 |  | [107987084](https://www.ncbi.nlm.nih.gov/gene/?term=107987084) | -2.2 | 0.02463 | 4.2 | 15.4 |
| HIST1H2BB | histone cluster 1, H2bb | [3018](https://www.ncbi.nlm.nih.gov/gene/?term=3018) | 2.2 | 0.00194 | 393.4 | 153.9 |
| ATP6V0C | ATPase H+ transporting V0 subunit c | [527](https://www.ncbi.nlm.nih.gov/gene/?term=527) | -2.2 | 0.00008 | 1223.1 | 2948.4 |
| GNG5 | G protein subunit gamma 5 | [2787](https://www.ncbi.nlm.nih.gov/gene/?term=2787) | 2.2 | 0.00000 | 1887.4 | 807.0 |
| CDC45 | cell division cycle 45 | [8318](https://www.ncbi.nlm.nih.gov/gene/?term=8318) | 2.2 | 0.01051 | 59.3 | 20.9 |
| GBAP1 | glucosylceramidase beta pseudogene 1 | [2630](https://www.ncbi.nlm.nih.gov/gene/?term=2630) | 2.2 | 0.00276 | 15.3 | 5.8 |
| LOC107986415 |  | [107986415](https://www.ncbi.nlm.nih.gov/gene/?term=107986415) | -2.2 | 0.01721 | 2.8 | 9.5 |
| NAMPTP1 | nicotinamide phosphoribosyltransferase pseudogene 1 | [646309](https://www.ncbi.nlm.nih.gov/gene/?term=646309) | 2.2 | 0.00050 | 418.1 | 170.0 |
| LOC107984718 |  | [107984718](https://www.ncbi.nlm.nih.gov/gene/?term=107984718) | -2.2 | 0.00735 | 20.8 | 58.0 |
| CD14 | CD14 molecule | [929](https://www.ncbi.nlm.nih.gov/gene/?term=929) | 2.2 | 0.00000 | 9478.6 | 4051.8 |
| MAPK11 | mitogen-activated protein kinase 11 | [5600](https://www.ncbi.nlm.nih.gov/gene/?term=5600) | 2.2 | 0.00003 | 68.9 | 28.8 |
| LOC101241902 | chromosome 4 open reading frame 46 pseudogene | [101241902](https://www.ncbi.nlm.nih.gov/gene/?term=101241902) | -2.2 | 0.01176 | 3.3 | 9.7 |
| RNF152 | ring finger protein 152 | [220441](https://www.ncbi.nlm.nih.gov/gene/?term=220441) | -2.2 | 0.00657 | 110.6 | 298.9 |
| RPL7AP60 | ribosomal protein L7a pseudogene 60 | [100271538](https://www.ncbi.nlm.nih.gov/gene/?term=100271538) | -2.2 | 0.00202 | 24.6 | 61.0 |
| LRP10 | LDL receptor related protein 10 | [26020](https://www.ncbi.nlm.nih.gov/gene/?term=26020) | -2.2 | 0.00012 | 1883.3 | 4537.6 |
| RSPH9 | radial spoke head 9 homolog | [221421](https://www.ncbi.nlm.nih.gov/gene/?term=221421) | 2.2 | 0.00149 | 51.9 | 20.3 |
| H3F3B | H3 histone, family 3B (H3.3B) | [3021](https://www.ncbi.nlm.nih.gov/gene/?term=3021) | 2.2 | 0.00001 | 36097.4 | 15471.9 |
| LOC100288910 |  | [100288910](https://www.ncbi.nlm.nih.gov/gene/?term=100288910) | 2.2 | 0.00029 | 15.3 | 6.2 |
| GCH1 | GTP cyclohydrolase 1 | [2643](https://www.ncbi.nlm.nih.gov/gene/?term=2643) | 2.2 | 0.00000 | 2425.6 | 1047.5 |
| SMG7 | SMG7 nonsense mediated mRNA decay factor | [9887](https://www.ncbi.nlm.nih.gov/gene/?term=9887) | -2.2 | 0.00001 | 1043.3 | 2452.7 |
| RHEBP1 | Ras-homolog enriched in brain pseudogene 1 | [6008](https://www.ncbi.nlm.nih.gov/gene/?term=6008) | 2.2 | 0.00430 | 10.7 | 4.1 |
| SLC6A8 | solute carrier family 6 member 8 | [6535](https://www.ncbi.nlm.nih.gov/gene/?term=6535) | -2.2 | 0.00478 | 3236.7 | 8636.6 |
| TBC1D25 | TBC1 domain family member 25 | [4943](https://www.ncbi.nlm.nih.gov/gene/?term=4943) | -2.2 | 0.00006 | 222.7 | 529.4 |
| FAM46C | family with sequence similarity 46 member C | [54855](https://www.ncbi.nlm.nih.gov/gene/?term=54855) | -2.2 | 0.00296 | 259010.1 | 670990.1 |
| PCMTD2 | protein-L-isoaspartate (D-aspartate) O-methyltransferase domain containing 2 | [55251](https://www.ncbi.nlm.nih.gov/gene/?term=55251) | -2.2 | 0.00022 | 1401.4 | 3402.6 |
| CASS4 | Cas scaffolding protein family member 4 | [57091](https://www.ncbi.nlm.nih.gov/gene/?term=57091) | -2.2 | 0.00000 | 369.6 | 852.3 |
| MVP | major vault protein | [9961](https://www.ncbi.nlm.nih.gov/gene/?term=9961) | 2.2 | 0.00000 | 6809.1 | 2964.8 |
| BISPR | BST2 interferon stimulated positive regulator (non-protein coding) | [105221694](https://www.ncbi.nlm.nih.gov/gene/?term=105221694) | 2.2 | 0.00017 | 336.7 | 140.1 |
| PTTG2 | pituitary tumor-transforming 2 | [10744](https://www.ncbi.nlm.nih.gov/gene/?term=10744) | -2.2 | 0.00475 | 15.2 | 41.5 |
| QPCT | glutaminyl-peptide cyclotransferase | [25797](https://www.ncbi.nlm.nih.gov/gene/?term=25797) | 2.2 | 0.00046 | 4193.2 | 1722.2 |
| LOC107984209 |  | [107984209](https://www.ncbi.nlm.nih.gov/gene/?term=107984209) | -2.2 | 0.02090 | 5.3 | 17.7 |
| AATBC | apoptosis associated transcript in bladder cancer | [284837](https://www.ncbi.nlm.nih.gov/gene/?term=284837) | 2.2 | 0.00099 | 375.2 | 151.5 |
| WDFY3-AS2 | WDFY3 antisense RNA 2 | [404201](https://www.ncbi.nlm.nih.gov/gene/?term=404201) | 2.2 | 0.01211 | 12.9 | 4.7 |
| EMILIN2 | elastin microfibril interfacer 2 | [84034](https://www.ncbi.nlm.nih.gov/gene/?term=84034) | 2.2 | 0.00001 | 3409.0 | 1459.9 |
| HPGD | hydroxyprostaglandin dehydrogenase 15-(NAD) | [3248](https://www.ncbi.nlm.nih.gov/gene/?term=3248) | 2.2 | 0.00181 | 562.0 | 223.8 |
| EPS8L1 | EPS8 like 1 | [54869](https://www.ncbi.nlm.nih.gov/gene/?term=54869) | 2.2 | 0.00124 | 18.8 | 7.8 |
| GIPC3 | GIPC PDZ domain containing family member 3 | [126326](https://www.ncbi.nlm.nih.gov/gene/?term=126326) | -2.2 | 0.01556 | 6.2 | 19.0 |
| ZNF883 | zinc finger protein 883 | [169834](https://www.ncbi.nlm.nih.gov/gene/?term=169834) | -2.2 | 0.00318 | 7.8 | 20.8 |
| ADAM9 | ADAM metallopeptidase domain 9 | [8754](https://www.ncbi.nlm.nih.gov/gene/?term=8754) | 2.2 | 0.00001 | 1121.2 | 484.0 |
| FCER2 | Fc fragment of IgE receptor II | [2208](https://www.ncbi.nlm.nih.gov/gene/?term=2208) | -2.2 | 0.00019 | 53.1 | 126.8 |
| LOC105375034 |  | [105375034](https://www.ncbi.nlm.nih.gov/gene/?term=105375034) | 2.2 | 0.00998 | 14.4 | 5.4 |
| TOM1 | target of myb1 membrane trafficking protein | [10043](https://www.ncbi.nlm.nih.gov/gene/?term=10043) | 2.2 | 0.00000 | 1432.8 | 624.5 |
| ASAP1-IT2 | ASAP1 intronic transcript 2 | [100507117](https://www.ncbi.nlm.nih.gov/gene/?term=100507117) | -2.2 | 0.01461 | 7.6 | 23.7 |
| SLA | Src-like-adaptor | [6503](https://www.ncbi.nlm.nih.gov/gene/?term=6503) | 2.2 | 0.00004 | 15292.5 | 6576.2 |
| HIST1H2AB | histone cluster 1, H2ab | [8335](https://www.ncbi.nlm.nih.gov/gene/?term=8335) | 2.2 | 0.00407 | 432.4 | 167.6 |
| PLAC8 | placenta specific 8 | [51316](https://www.ncbi.nlm.nih.gov/gene/?term=51316) | 2.2 | 0.00001 | 5989.8 | 2577.9 |
| CHP1 | calcineurin like EF-hand protein 1 | [11261](https://www.ncbi.nlm.nih.gov/gene/?term=11261) | -2.2 | 0.00012 | 1518.5 | 3607.6 |
| LOC153893 | defective in cullin neddylation 1 domain containing 1 pseudogene | [153893](https://www.ncbi.nlm.nih.gov/gene/?term=153893) | -2.2 | 0.00208 | 8.1 | 18.9 |
| DTX4 | deltex 4, E3 ubiquitin ligase | [23220](https://www.ncbi.nlm.nih.gov/gene/?term=23220) | -2.2 | 0.00000 | 103.4 | 237.7 |
| KCNE1 | potassium voltage-gated channel subfamily E regulatory subunit 1 | [3753](https://www.ncbi.nlm.nih.gov/gene/?term=3753) | 2.2 | 0.00067 | 91.4 | 37.5 |
| RN7SL767P | RNA, 7SL, cytoplasmic 767, pseudogene | [106481131](https://www.ncbi.nlm.nih.gov/gene/?term=106481131) | -2.2 | 0.00095 | 7.7 | 16.4 |
| NOL3 | nucleolar protein 3 | [8996](https://www.ncbi.nlm.nih.gov/gene/?term=8996) | 2.2 | 0.00029 | 36.5 | 15.2 |
| LOC105371260 |  | [105371260](https://www.ncbi.nlm.nih.gov/gene/?term=105371260) | 2.2 | 0.00015 | 13.8 | 5.9 |
| HRASLS5 | HRAS like suppressor family member 5 | [117245](https://www.ncbi.nlm.nih.gov/gene/?term=117245) | -2.2 | 0.00428 | 7.5 | 19.5 |
| SMCO4 | single-pass membrane protein with coiled-coil domains 4 | [56935](https://www.ncbi.nlm.nih.gov/gene/?term=56935) | 2.2 | 0.00000 | 336.9 | 147.4 |
| IRAK2 | interleukin 1 receptor associated kinase 2 | [3656](https://www.ncbi.nlm.nih.gov/gene/?term=3656) | 2.2 | 0.00000 | 524.7 | 228.5 |
| BCL2L1 | BCL2 like 1 | [598](https://www.ncbi.nlm.nih.gov/gene/?term=598) | -2.2 | 0.00254 | 33256.8 | 85076.2 |
| ALG1L2 | ALG1, chitobiosyldiphosphodolichol beta-mannosyltransferase-like 2 | [644974](https://www.ncbi.nlm.nih.gov/gene/?term=644974) | 2.2 | 0.00772 | 16.1 | 5.7 |
| FCN1 | ficolin 1 | [2219](https://www.ncbi.nlm.nih.gov/gene/?term=2219) | 2.2 | 0.00001 | 22531.1 | 9731.7 |
| TIGD3 | tigger transposable element derived 3 | [220359](https://www.ncbi.nlm.nih.gov/gene/?term=220359) | -2.2 | 0.00011 | 42.5 | 101.1 |
| HIST1H2AD | histone cluster 1, H2ad | [3013](https://www.ncbi.nlm.nih.gov/gene/?term=3013) | 2.2 | 0.01378 | 8.1 | 3.2 |
| C7orf73 | chromosome 7 open reading frame 73 | [647087](https://www.ncbi.nlm.nih.gov/gene/?term=647087) | -2.2 | 0.00082 | 3283.1 | 8115.6 |
| LOC107986092 |  | [107986092](https://www.ncbi.nlm.nih.gov/gene/?term=107986092) | 2.2 | 0.00056 | 32.6 | 13.4 |
| SERTAD1 | SERTA domain containing 1 | [29950](https://www.ncbi.nlm.nih.gov/gene/?term=29950) | 2.2 | 0.00000 | 419.4 | 186.2 |
| TIMD4 | T-cell immunoglobulin and mucin domain containing 4 | [91937](https://www.ncbi.nlm.nih.gov/gene/?term=91937) | 2.2 | 0.00390 | 43.7 | 17.3 |
| NIPSNAP3B | nipsnap homolog 3B | [55335](https://www.ncbi.nlm.nih.gov/gene/?term=55335) | -2.2 | 0.00003 | 26.2 | 61.0 |
| IGFLR1 | IGF like family receptor 1 | [79713](https://www.ncbi.nlm.nih.gov/gene/?term=79713) | 2.2 | 0.00000 | 485.4 | 212.3 |
| OGFR | opioid growth factor receptor | [11054](https://www.ncbi.nlm.nih.gov/gene/?term=11054) | 2.2 | 0.00000 | 3053.6 | 1352.2 |
| LOC107984335 |  | [107984335](https://www.ncbi.nlm.nih.gov/gene/?term=107984335) | 2.2 | 0.00880 | 9.1 | 3.3 |
| ECRP | ribonuclease A family member 2 pseudogene | [643332](https://www.ncbi.nlm.nih.gov/gene/?term=643332) | 2.2 | 0.00283 | 29.2 | 11.4 |
| LOC101927963 |  | [101927963](https://www.ncbi.nlm.nih.gov/gene/?term=101927963) | 2.2 | 0.00006 | 151.5 | 65.0 |
| LOC107986698 |  | [107986698](https://www.ncbi.nlm.nih.gov/gene/?term=107986698) | -2.2 | 0.00211 | 115.2 | 287.9 |
| PKMYT1 | protein kinase, membrane associated tyrosine/threonine 1 | [9088](https://www.ncbi.nlm.nih.gov/gene/?term=9088) | 2.2 | 0.01594 | 14.9 | 5.3 |
| C2orf48 | chromosome 2 open reading frame 48 | [348738](https://www.ncbi.nlm.nih.gov/gene/?term=348738) | 2.2 | 0.00916 | 10.5 | 3.6 |
| IP6K1 | inositol hexakisphosphate kinase 1 | [9807](https://www.ncbi.nlm.nih.gov/gene/?term=9807) | -2.2 | 0.00000 | 896.5 | 2049.7 |
| CD300A | CD300a molecule | [11314](https://www.ncbi.nlm.nih.gov/gene/?term=11314) | 2.2 | 0.00000 | 3854.2 | 1696.3 |
| UHRF1 | ubiquitin like with PHD and ring finger domains 1 | [29128](https://www.ncbi.nlm.nih.gov/gene/?term=29128) | 2.2 | 0.00205 | 188.5 | 76.3 |
| DNAJB1 | DnaJ heat shock protein family (Hsp40) member B1 | [3337](https://www.ncbi.nlm.nih.gov/gene/?term=3337) | 2.2 | 0.00016 | 2569.1 | 1099.6 |
| LOC401052 | uncharacterized LOC401052 | [401052](https://www.ncbi.nlm.nih.gov/gene/?term=401052) | -2.2 | 0.01443 | 3.4 | 8.9 |
| LOC105372185 |  | [105372185](https://www.ncbi.nlm.nih.gov/gene/?term=105372185) | -2.2 | 0.01826 | 3.1 | 8.5 |
| APOL1 | apolipoprotein L1 | [8542](https://www.ncbi.nlm.nih.gov/gene/?term=8542) | 2.2 | 0.00029 | 1348.4 | 569.4 |
| PHOSPHO2 | phosphatase, orphan 2 | [493911](https://www.ncbi.nlm.nih.gov/gene/?term=493911) | -2.2 | 0.00003 | 15.3 | 36.4 |
| TMEM123 | transmembrane protein 123 | [114908](https://www.ncbi.nlm.nih.gov/gene/?term=114908) | 2.2 | 0.00001 | 20265.1 | 8890.8 |
| LOXL3 | lysyl oxidase like 3 | [84695](https://www.ncbi.nlm.nih.gov/gene/?term=84695) | 2.2 | 0.00000 | 272.2 | 120.0 |
| LOC107985002 |  | [107985002](https://www.ncbi.nlm.nih.gov/gene/?term=107985002) | -2.2 | 0.01218 | 9.9 | 28.1 |
| GIPC2 | GIPC PDZ domain containing family member 2 | [54810](https://www.ncbi.nlm.nih.gov/gene/?term=54810) | -2.2 | 0.00902 | 9.8 | 25.5 |
| UBTD1 | ubiquitin domain containing 1 | [80019](https://www.ncbi.nlm.nih.gov/gene/?term=80019) | 2.2 | 0.00001 | 131.3 | 57.7 |
| UBE2E2 | ubiquitin conjugating enzyme E2 E2 | [7325](https://www.ncbi.nlm.nih.gov/gene/?term=7325) | -2.2 | 0.00070 | 205.5 | 495.9 |
| BIRC5 | baculoviral IAP repeat containing 5 | [332](https://www.ncbi.nlm.nih.gov/gene/?term=332) | 2.2 | 0.00546 | 179.1 | 69.8 |
| MIR548AR | microRNA 548ar | [100847035](https://www.ncbi.nlm.nih.gov/gene/?term=100847035) | -2.2 | 0.00279 | 7.3 | 19.2 |
| SLC37A3 | solute carrier family 37 member 3 | [84255](https://www.ncbi.nlm.nih.gov/gene/?term=84255) | 2.2 | 0.00014 | 1122.2 | 481.6 |
| LOC105371026 |  | [105371026](https://www.ncbi.nlm.nih.gov/gene/?term=105371026) | 2.2 | 0.00037 | 91.0 | 38.7 |
| SAPCD2 | suppressor APC domain containing 2 | [89958](https://www.ncbi.nlm.nih.gov/gene/?term=89958) | 2.2 | 0.00126 | 97.6 | 40.4 |
| ASCL2 | achaete-scute family bHLH transcription factor 2 | [430](https://www.ncbi.nlm.nih.gov/gene/?term=430) | 2.2 | 0.00001 | 183.8 | 80.1 |
| OSCAR | osteoclast associated, immunoglobulin-like receptor | [126014](https://www.ncbi.nlm.nih.gov/gene/?term=126014) | 2.2 | 0.00121 | 43.5 | 17.9 |
| RPS14P3 | ribosomal protein S14 pseudogene 3 | [644068](https://www.ncbi.nlm.nih.gov/gene/?term=644068) | -2.2 | 0.00004 | 17.2 | 40.5 |
| LOC100421561 | family with sequence similarity 133 member B pseudogene | [100421561](https://www.ncbi.nlm.nih.gov/gene/?term=100421561) | -2.2 | 0.00240 | 46.9 | 116.8 |
| SCPEP1 | serine carboxypeptidase 1 | [59342](https://www.ncbi.nlm.nih.gov/gene/?term=59342) | 2.2 | 0.00000 | 3780.7 | 1681.3 |
| FAM89A | family with sequence similarity 89 member A | [375061](https://www.ncbi.nlm.nih.gov/gene/?term=375061) | 2.2 | 0.00018 | 88.0 | 37.5 |
| LOC105369315 |  | [105369315](https://www.ncbi.nlm.nih.gov/gene/?term=105369315) | -2.1 | 0.00085 | 206.5 | 493.4 |
| FOXO3 | forkhead box O3 | [2309](https://www.ncbi.nlm.nih.gov/gene/?term=2309) | -2.1 | 0.00410 | 48886.8 | 123545.7 |
| RALB | v-ral simian leukemia viral oncogene homolog B | [5899](https://www.ncbi.nlm.nih.gov/gene/?term=5899) | 2.1 | 0.00002 | 5995.3 | 2631.5 |
| LOC107985120 |  | [107985120](https://www.ncbi.nlm.nih.gov/gene/?term=107985120) | -2.1 | 0.00869 | 21.9 | 58.8 |
| LOC107987261 |  | [107987261](https://www.ncbi.nlm.nih.gov/gene/?term=107987261) | 2.1 | 0.00474 | 12.5 | 4.8 |
| IL17RC | interleukin 17 receptor C | [84818](https://www.ncbi.nlm.nih.gov/gene/?term=84818) | 2.1 | 0.00050 | 28.6 | 11.9 |
| PAQR4 | progestin and adipoQ receptor family member 4 | [124222](https://www.ncbi.nlm.nih.gov/gene/?term=124222) | 2.1 | 0.00000 | 104.3 | 46.4 |
| HPDL | 4-hydroxyphenylpyruvate dioxygenase like | [84842](https://www.ncbi.nlm.nih.gov/gene/?term=84842) | 2.1 | 0.02163 | 12.6 | 4.2 |
| FOXJ2 | forkhead box J2 | [55810](https://www.ncbi.nlm.nih.gov/gene/?term=55810) | -2.1 | 0.00005 | 357.3 | 816.9 |
| STAB1 | stabilin 1 | [23166](https://www.ncbi.nlm.nih.gov/gene/?term=23166) | 2.1 | 0.00038 | 2600.9 | 1113.0 |
| ALDH1A2 | aldehyde dehydrogenase 1 family member A2 | [8854](https://www.ncbi.nlm.nih.gov/gene/?term=8854) | 2.1 | 0.00989 | 13.2 | 5.0 |
| NETO1 | neuropilin and tolloid like 1 | [81832](https://www.ncbi.nlm.nih.gov/gene/?term=81832) | -2.1 | 0.00224 | 11.6 | 27.8 |
| TRIM5 | tripartite motif containing 5 | [85363](https://www.ncbi.nlm.nih.gov/gene/?term=85363) | 2.1 | 0.00005 | 1998.2 | 877.4 |
| CHMP5 | charged multivesicular body protein 5 | [51510](https://www.ncbi.nlm.nih.gov/gene/?term=51510) | 2.1 | 0.00000 | 4235.5 | 1893.2 |
| MAGEE1 | MAGE family member E1 | [57692](https://www.ncbi.nlm.nih.gov/gene/?term=57692) | -2.1 | 0.00103 | 10.2 | 25.3 |
| ITGB2 | integrin subunit beta 2 | [3689](https://www.ncbi.nlm.nih.gov/gene/?term=3689) | 2.1 | 0.00000 | 36045.6 | 16412.6 |
| FBXL19 | F-box and leucine-rich repeat protein 19 | [54620](https://www.ncbi.nlm.nih.gov/gene/?term=54620) | 2.1 | 0.00000 | 252.0 | 113.9 |
| LOC105370774 |  | [105370774](https://www.ncbi.nlm.nih.gov/gene/?term=105370774) | -2.1 | 0.01335 | 4.9 | 14.6 |
| LOC107985780 |  | [107985780](https://www.ncbi.nlm.nih.gov/gene/?term=107985780) | -2.1 | 0.00413 | 12.5 | 31.9 |
| GPC4 | glypican 4 | [2239](https://www.ncbi.nlm.nih.gov/gene/?term=2239) | -2.1 | 0.02324 | 5.3 | 16.9 |
| SHISA5 | shisa family member 5 | [51246](https://www.ncbi.nlm.nih.gov/gene/?term=51246) | 2.1 | 0.00004 | 6450.4 | 2845.2 |
| MICAL2 | microtubule associated monooxygenase, calponin and LIM domain containing 2 | [9645](https://www.ncbi.nlm.nih.gov/gene/?term=9645) | -2.1 | 0.00271 | 16507.0 | 40974.8 |
| USP12 | ubiquitin specific peptidase 12 | [219333](https://www.ncbi.nlm.nih.gov/gene/?term=219333) | -2.1 | 0.00266 | 33112.9 | 80927.1 |
| ZWINT | ZW10 interacting kinetochore protein | [11130](https://www.ncbi.nlm.nih.gov/gene/?term=11130) | 2.1 | 0.00180 | 194.3 | 81.0 |
| SMTN | smoothelin | [6525](https://www.ncbi.nlm.nih.gov/gene/?term=6525) | 2.1 | 0.00205 | 51.4 | 21.2 |
| ZMAT1 | zinc finger matrin-type 1 | [84460](https://www.ncbi.nlm.nih.gov/gene/?term=84460) | -2.1 | 0.00001 | 206.0 | 461.9 |
| RAD23A | RAD23 homolog A, nucleotide excision repair protein | [5886](https://www.ncbi.nlm.nih.gov/gene/?term=5886) | -2.1 | 0.00305 | 680.4 | 1672.2 |
| MTHFD2 | methylenetetrahydrofolate dehydrogenase (NADP+ dependent) 2, methenyltetrahydrofolate cyclohydrolase | [10797](https://www.ncbi.nlm.nih.gov/gene/?term=10797) | 2.1 | 0.00000 | 1585.1 | 718.1 |
| CXCL11 | C-X-C motif chemokine ligand 11 | [6373](https://www.ncbi.nlm.nih.gov/gene/?term=6373) | 2.1 | 0.01921 | 23.4 | 8.0 |
| GID4 | GID complex subunit 4 homolog | [79018](https://www.ncbi.nlm.nih.gov/gene/?term=79018) | -2.1 | 0.00133 | 1288.5 | 3077.3 |
| LOC105371795 | uncharacterized LOC105371795 | [105371795](https://www.ncbi.nlm.nih.gov/gene/?term=105371795) | 2.1 | 0.01052 | 12.2 | 4.6 |
| HLA-K | major histocompatibility complex, class I, K (pseudogene) | [3138](https://www.ncbi.nlm.nih.gov/gene/?term=3138) | 2.1 | 0.01079 | 33.0 | 12.5 |
| LOC107985276 |  | [107985276](https://www.ncbi.nlm.nih.gov/gene/?term=107985276) | -2.1 | 0.00174 | 14.3 | 35.2 |
| AMOT | angiomotin | [154796](https://www.ncbi.nlm.nih.gov/gene/?term=154796) | -2.1 | 0.00001 | 33.8 | 74.1 |
| UBE2D1 | ubiquitin conjugating enzyme E2D 1 | [7321](https://www.ncbi.nlm.nih.gov/gene/?term=7321) | 2.1 | 0.00013 | 3362.8 | 1478.7 |
| MLKL | mixed lineage kinase domain-like | [197259](https://www.ncbi.nlm.nih.gov/gene/?term=197259) | 2.1 | 0.00017 | 2127.3 | 933.6 |
| LOC105373264 |  | [105373264](https://www.ncbi.nlm.nih.gov/gene/?term=105373264) | -2.1 | 0.00423 | 24.0 | 60.4 |
| TMCC2 | transmembrane and coiled-coil domain family 2 | [9911](https://www.ncbi.nlm.nih.gov/gene/?term=9911) | -2.1 | 0.00413 | 4565.3 | 11207.4 |
| CATIP | ciliogenesis associated TTC17 interacting protein | [375307](https://www.ncbi.nlm.nih.gov/gene/?term=375307) | 2.1 | 0.00354 | 71.6 | 29.4 |
| PLCB1-IT1 |  | [100874337](https://www.ncbi.nlm.nih.gov/gene/?term=100874337) | -2.1 | 0.00681 | 20.1 | 51.7 |
| ADAM28 | ADAM metallopeptidase domain 28 | [10863](https://www.ncbi.nlm.nih.gov/gene/?term=10863) | -2.1 | 0.00001 | 430.3 | 955.7 |
| CLIC5 | chloride intracellular channel 5 | [53405](https://www.ncbi.nlm.nih.gov/gene/?term=53405) | -2.1 | 0.00016 | 26.9 | 62.7 |
| SGK223 | homolog of rat pragma of Rnd2 | [157285](https://www.ncbi.nlm.nih.gov/gene/?term=157285) | -2.1 | 0.00100 | 29.9 | 70.6 |
| CXCR1 | C-X-C motif chemokine receptor 1 | [3577](https://www.ncbi.nlm.nih.gov/gene/?term=3577) | 2.1 | 0.00026 | 14423.9 | 6311.8 |
| LOC107983997 |  | [107983997](https://www.ncbi.nlm.nih.gov/gene/?term=107983997) | -2.1 | 0.00006 | 83.7 | 189.5 |
| FOXN3-AS1 | FOXN3 antisense RNA 1 | [400236](https://www.ncbi.nlm.nih.gov/gene/?term=400236) | 2.1 | 0.00000 | 38.6 | 17.2 |
| VDAC2P2 | voltage dependent anion channel 2 pseudogene 2 | [643996](https://www.ncbi.nlm.nih.gov/gene/?term=643996) | -2.1 | 0.02893 | 6.4 | 22.8 |
| LOC105377907 |  | [105377907](https://www.ncbi.nlm.nih.gov/gene/?term=105377907) | -2.1 | 0.00109 | 25.8 | 61.9 |
| MARCH8 | membrane associated ring-CH-type finger 8 | [220972](https://www.ncbi.nlm.nih.gov/gene/?term=220972) | -2.1 | 0.00183 | 15914.2 | 37925.2 |
| LOC105375138 |  | [105375138](https://www.ncbi.nlm.nih.gov/gene/?term=105375138) | -2.1 | 0.00537 | 16.9 | 41.6 |
| HIST1H2AH | histone cluster 1, H2ah | [85235](https://www.ncbi.nlm.nih.gov/gene/?term=85235) | 2.1 | 0.00911 | 526.6 | 204.5 |
| HIST2H3D | histone cluster 2, H3d | [653604](https://www.ncbi.nlm.nih.gov/gene/?term=653604) | 2.1 | 0.00014 | 573.8 | 253.0 |
| HIST1H4I | histone cluster 1, H4i | [8294](https://www.ncbi.nlm.nih.gov/gene/?term=8294) | 2.1 | 0.00061 | 431.2 | 186.2 |
| CEP126 | centrosomal protein 126 | [57562](https://www.ncbi.nlm.nih.gov/gene/?term=57562) | -2.1 | 0.00019 | 35.2 | 80.3 |
| C9orf66 | chromosome 9 open reading frame 66 | [157983](https://www.ncbi.nlm.nih.gov/gene/?term=157983) | 2.1 | 0.00003 | 75.3 | 33.9 |
| MTX1 | metaxin 1 | [4580](https://www.ncbi.nlm.nih.gov/gene/?term=4580) | 2.1 | 0.00001 | 465.6 | 210.9 |
| PRSS35 | protease, serine 35 | [167681](https://www.ncbi.nlm.nih.gov/gene/?term=167681) | -2.1 | 0.01908 | 2.8 | 8.1 |
| LOC107984952 |  | [107984952](https://www.ncbi.nlm.nih.gov/gene/?term=107984952) | 2.1 | 0.00173 | 13.5 | 5.7 |
| FCGR2A | Fc fragment of IgG receptor IIa | [2212](https://www.ncbi.nlm.nih.gov/gene/?term=2212) | 2.1 | 0.00008 | 29956.3 | 13332.9 |
| PHF5GP | PHD finger protein 5G pseudogene | [450234](https://www.ncbi.nlm.nih.gov/gene/?term=450234) | -2.1 | 0.03213 | 2.0 | 8.0 |
| COLQ | collagen-like tail subunit (single strand of homotrimer) of asymmetric acetylcholinesterase | [8292](https://www.ncbi.nlm.nih.gov/gene/?term=8292) | -2.1 | 0.00153 | 54.5 | 127.4 |
| SNORD6 | small nucleolar RNA, C/D box 6 | [692075](https://www.ncbi.nlm.nih.gov/gene/?term=692075) | -2.1 | 0.00026 | 11.1 | 26.4 |
| MIR4802 | microRNA 4802 | [100616274](https://www.ncbi.nlm.nih.gov/gene/?term=100616274) | -2.1 | 0.02974 | 2.9 | 10.5 |
| LOC101928387 |  | [101928387](https://www.ncbi.nlm.nih.gov/gene/?term=101928387) | -2.1 | 0.00083 | 14.3 | 33.6 |
| SLC35E1 | solute carrier family 35 member E1 | [79939](https://www.ncbi.nlm.nih.gov/gene/?term=79939) | -2.1 | 0.00001 | 541.1 | 1189.6 |
| LOC105370569 |  | [105370569](https://www.ncbi.nlm.nih.gov/gene/?term=105370569) | -2.1 | 0.00289 | 31.1 | 75.6 |
| KLHL2 | kelch like family member 2 | [11275](https://www.ncbi.nlm.nih.gov/gene/?term=11275) | 2.1 | 0.00020 | 2945.8 | 1310.3 |
| TMEM117 | transmembrane protein 117 | [84216](https://www.ncbi.nlm.nih.gov/gene/?term=84216) | -2.1 | 0.00000 | 27.7 | 60.9 |
| SOX21-AS1 | SOX21 antisense RNA 1 (head to head) | [100507533](https://www.ncbi.nlm.nih.gov/gene/?term=100507533) | -2.1 | 0.02639 | 1.4 | 6.0 |
| LOC644090 |  | [644090](https://www.ncbi.nlm.nih.gov/gene/?term=644090) | 2.1 | 0.01611 | 8.8 | 3.2 |
| TGFB2 | transforming growth factor beta 2 | [7042](https://www.ncbi.nlm.nih.gov/gene/?term=7042) | -2.1 | 0.00083 | 13.3 | 30.7 |
| GBP2 | guanylate binding protein 2 | [2634](https://www.ncbi.nlm.nih.gov/gene/?term=2634) | 2.1 | 0.00009 | 18559.4 | 8344.0 |
| HIST1H3F | histone cluster 1, H3f | [8968](https://www.ncbi.nlm.nih.gov/gene/?term=8968) | 2.1 | 0.00674 | 1041.6 | 417.6 |
| TCP11L2 | t-complex 11 like 2 | [255394](https://www.ncbi.nlm.nih.gov/gene/?term=255394) | -2.1 | 0.00264 | 31499.9 | 79214.8 |
| MCOLN3 | mucolipin 3 | [55283](https://www.ncbi.nlm.nih.gov/gene/?term=55283) | -2.1 | 0.01364 | 4.6 | 12.6 |
| GPR75 | G protein-coupled receptor 75 | [10936](https://www.ncbi.nlm.nih.gov/gene/?term=10936) | -2.1 | 0.00420 | 12.8 | 31.9 |
| LOC107986997 |  | [107986997](https://www.ncbi.nlm.nih.gov/gene/?term=107986997) | -2.1 | 0.00151 | 44.6 | 106.0 |
| MBLAC1 | metallo-beta-lactamase domain containing 1 | [255374](https://www.ncbi.nlm.nih.gov/gene/?term=255374) | 2.1 | 0.00005 | 27.1 | 11.9 |
| ELMO2 | engulfment and cell motility 2 | [63916](https://www.ncbi.nlm.nih.gov/gene/?term=63916) | 2.1 | 0.00000 | 1999.8 | 927.6 |
| MIR4738 | microRNA 4738 | [100616282](https://www.ncbi.nlm.nih.gov/gene/?term=100616282) | 2.1 | 0.01798 | 10.4 | 3.8 |
| SKI | v-ski avian sarcoma viral oncogene homolog | [6497](https://www.ncbi.nlm.nih.gov/gene/?term=6497) | -2.1 | 0.00153 | 247.3 | 580.5 |
| LOC100130899 | uncharacterized LOC100130899 | [100130899](https://www.ncbi.nlm.nih.gov/gene/?term=100130899) | -2.1 | 0.00609 | 19.6 | 47.6 |
| CARD9 | caspase recruitment domain family member 9 | [64170](https://www.ncbi.nlm.nih.gov/gene/?term=64170) | 2.1 | 0.00008 | 500.1 | 224.0 |
| TREML3P | triggering receptor expressed on myeloid cells like 3, pseudogene | [340206](https://www.ncbi.nlm.nih.gov/gene/?term=340206) | -2.1 | 0.00106 | 30.2 | 70.2 |
| PCBP1 | poly(rC) binding protein 1 | [5093](https://www.ncbi.nlm.nih.gov/gene/?term=5093) | -2.1 | 0.00002 | 2584.2 | 5674.2 |
| IRF9 | interferon regulatory factor 9 | [10379](https://www.ncbi.nlm.nih.gov/gene/?term=10379) | 2.1 | 0.00000 | 4620.6 | 2126.2 |
| TECPR2 | tectonin beta-propeller repeat containing 2 | [9895](https://www.ncbi.nlm.nih.gov/gene/?term=9895) | 2.1 | 0.00001 | 3815.7 | 1736.3 |
| LOC105373711 |  | [105373711](https://www.ncbi.nlm.nih.gov/gene/?term=105373711) | -2.1 | 0.00344 | 51.9 | 125.4 |
| TFEC | transcription factor EC | [22797](https://www.ncbi.nlm.nih.gov/gene/?term=22797) | 2.1 | 0.00027 | 2819.9 | 1249.4 |
| TMEM231 | transmembrane protein 231 | [79583](https://www.ncbi.nlm.nih.gov/gene/?term=79583) | -2.1 | 0.00974 | 4.0 | 11.4 |
| LOC100335030 | FGFR1 oncogene partner 2 pseudogene | [100335030](https://www.ncbi.nlm.nih.gov/gene/?term=100335030) | -2.1 | 0.01353 | 7.6 | 20.9 |
| CPSF6 | cleavage and polyadenylation specific factor 6 | [11052](https://www.ncbi.nlm.nih.gov/gene/?term=11052) | -2.1 | 0.00000 | 1028.4 | 2232.4 |
| GOLGA8A | golgin A8 family member A | [23015](https://www.ncbi.nlm.nih.gov/gene/?term=23015) | -2.1 | 0.00015 | 303.0 | 677.8 |
| FOLR3 | folate receptor 3 (gamma) | [2352](https://www.ncbi.nlm.nih.gov/gene/?term=2352) | 2.1 | 0.00719 | 1414.0 | 569.6 |
| LOC105369464 |  | [105369464](https://www.ncbi.nlm.nih.gov/gene/?term=105369464) | -2.1 | 0.02267 | 3.2 | 9.8 |
| LOC105373730 |  | [105373730](https://www.ncbi.nlm.nih.gov/gene/?term=105373730) | -2.1 | 0.00271 | 18.4 | 44.8 |
| LOC107984127 |  | [107984127](https://www.ncbi.nlm.nih.gov/gene/?term=107984127) | -2.1 | 0.00002 | 74.8 | 164.0 |
| SIGLEC1 | sialic acid binding Ig like lectin 1 | [6614](https://www.ncbi.nlm.nih.gov/gene/?term=6614) | 2.1 | 0.03318 | 1023.2 | 281.1 |
| CD300E | CD300e molecule | [342510](https://www.ncbi.nlm.nih.gov/gene/?term=342510) | 2.1 | 0.00019 | 2346.0 | 1050.1 |
| RCOR3 | REST corepressor 3 | [55758](https://www.ncbi.nlm.nih.gov/gene/?term=55758) | -2.1 | 0.00007 | 1957.1 | 4320.2 |
| LOC101929007 |  | [101929007](https://www.ncbi.nlm.nih.gov/gene/?term=101929007) | -2.1 | 0.01554 | 1465.3 | 3959.8 |
| RRAS | related RAS viral (r-ras) oncogene homolog | [6237](https://www.ncbi.nlm.nih.gov/gene/?term=6237) | 2.1 | 0.00001 | 176.2 | 80.5 |
| VWF | von Willebrand factor | [7450](https://www.ncbi.nlm.nih.gov/gene/?term=7450) | -2.1 | 0.00566 | 56.5 | 138.6 |
| LOC100420110 | SCY1 like pseudokinase 2 pseudogene | [100420110](https://www.ncbi.nlm.nih.gov/gene/?term=100420110) | -2.1 | 0.00227 | 341.0 | 796.7 |
| RAB32 | RAB32, member RAS oncogene family | [10981](https://www.ncbi.nlm.nih.gov/gene/?term=10981) | 2.1 | 0.00011 | 1736.8 | 787.0 |
| BTBD6P1 | BTB domain containing 6 pseudogene 1 | [646330](https://www.ncbi.nlm.nih.gov/gene/?term=646330) | -2.1 | 0.00177 | 21.9 | 51.6 |
| PSME4 | proteasome activator subunit 4 | [23198](https://www.ncbi.nlm.nih.gov/gene/?term=23198) | -2.1 | 0.00289 | 13764.9 | 32564.6 |
| ZNF66 | zinc finger protein 66 | [7617](https://www.ncbi.nlm.nih.gov/gene/?term=7617) | -2.1 | 0.00001 | 38.6 | 84.6 |
| FRAT2 | frequently rearranged in advanced T-cell lymphomas 2 | [23401](https://www.ncbi.nlm.nih.gov/gene/?term=23401) | 2.1 | 0.00007 | 5571.6 | 2531.7 |
| LOC648771 |  | [648771](https://www.ncbi.nlm.nih.gov/gene/?term=648771) | -2.1 | 0.02824 | 0.8 | 2.3 |
| C4orf32 | chromosome 4 open reading frame 32 | [132720](https://www.ncbi.nlm.nih.gov/gene/?term=132720) | 2.1 | 0.00000 | 325.9 | 152.4 |
| ANKRD34B | ankyrin repeat domain 34B | [340120](https://www.ncbi.nlm.nih.gov/gene/?term=340120) | 2.1 | 0.02793 | 74.8 | 24.2 |
| CMPK2 | cytidine/uridine monophosphate kinase 2 | [129607](https://www.ncbi.nlm.nih.gov/gene/?term=129607) | 2.1 | 0.00650 | 7318.7 | 3010.9 |
| PYGL | phosphorylase, glycogen, liver | [5836](https://www.ncbi.nlm.nih.gov/gene/?term=5836) | 2.1 | 0.00049 | 11969.9 | 5312.9 |
| TNNI2 | troponin I2, fast skeletal type | [7136](https://www.ncbi.nlm.nih.gov/gene/?term=7136) | 2.1 | 0.00007 | 82.3 | 37.2 |
| LOC105377628 |  | [105377628](https://www.ncbi.nlm.nih.gov/gene/?term=105377628) | -2.1 | 0.00505 | 9.9 | 23.3 |
| RNF135 | ring finger protein 135 | [84282](https://www.ncbi.nlm.nih.gov/gene/?term=84282) | 2.1 | 0.00000 | 882.1 | 415.2 |
| CNIH4 | cornichon family AMPA receptor auxiliary protein 4 | [29097](https://www.ncbi.nlm.nih.gov/gene/?term=29097) | 2.1 | 0.00001 | 1031.2 | 475.4 |
| GRPR | gastrin releasing peptide receptor | [2925](https://www.ncbi.nlm.nih.gov/gene/?term=2925) | -2.1 | 0.00892 | 2.8 | 7.5 |
| CLEC7A | C-type lectin domain family 7 member A | [64581](https://www.ncbi.nlm.nih.gov/gene/?term=64581) | 2.1 | 0.00000 | 8249.9 | 3870.7 |
| HBA1 | hemoglobin subunit alpha 1 | [3039](https://www.ncbi.nlm.nih.gov/gene/?term=3039) | -2.1 | 0.01064 | 87.2 | 222.6 |
| ZHX2 | zinc fingers and homeoboxes 2 | [22882](https://www.ncbi.nlm.nih.gov/gene/?term=22882) | -2.1 | 0.00012 | 211.7 | 468.5 |
| AGTRAP | angiotensin II receptor associated protein | [57085](https://www.ncbi.nlm.nih.gov/gene/?term=57085) | 2.1 | 0.00000 | 791.8 | 372.5 |
| LOC102724979 |  | [102724979](https://www.ncbi.nlm.nih.gov/gene/?term=102724979) | 2.1 | 0.00143 | 43.4 | 18.6 |
| TTC26 | tetratricopeptide repeat domain 26 | [79989](https://www.ncbi.nlm.nih.gov/gene/?term=79989) | 2.1 | 0.00477 | 160.5 | 66.9 |
| KL | klotho | [9365](https://www.ncbi.nlm.nih.gov/gene/?term=9365) | 2.1 | 0.00974 | 32.4 | 12.7 |
| WBP11 | WW domain binding protein 11 | [51729](https://www.ncbi.nlm.nih.gov/gene/?term=51729) | -2.1 | 0.00063 | 136.8 | 312.3 |
| AMIGO1 | adhesion molecule with Ig-like domain 1 | [57463](https://www.ncbi.nlm.nih.gov/gene/?term=57463) | -2.1 | 0.00116 | 70.9 | 163.5 |
| WASF3 | WAS protein family member 3 | [10810](https://www.ncbi.nlm.nih.gov/gene/?term=10810) | -2.1 | 0.01131 | 20.4 | 51.9 |
| HELZ2 | helicase with zinc finger 2 | [85441](https://www.ncbi.nlm.nih.gov/gene/?term=85441) | 2.1 | 0.00217 | 3489.0 | 1522.9 |
| LOC105370978 |  | [105370978](https://www.ncbi.nlm.nih.gov/gene/?term=105370978) | 2.1 | 0.00887 | 12.6 | 5.2 |
| PABPC1 | poly(A) binding protein cytoplasmic 1 | [26986](https://www.ncbi.nlm.nih.gov/gene/?term=26986) | -2.1 | 0.00037 | 10383.5 | 23193.7 |
| LOC400682 |  | [400682](https://www.ncbi.nlm.nih.gov/gene/?term=400682) | -2.1 | 0.01794 | 19.3 | 52.8 |
| RPS27P7 | ribosomal protein S27 pseudogene 7 | [100131787](https://www.ncbi.nlm.nih.gov/gene/?term=100131787) | -2.1 | 0.03173 | 2.2 | 8.1 |
| ZNF777 | zinc finger protein 777 | [27153](https://www.ncbi.nlm.nih.gov/gene/?term=27153) | -2.1 | 0.00092 | 53.4 | 122.1 |
| TVP23C | trans-golgi network vesicle protein 23 homolog C (S. cerevisiae) | [201158](https://www.ncbi.nlm.nih.gov/gene/?term=201158) | -2.1 | 0.00107 | 35.5 | 82.1 |
| MYLK3 | myosin light chain kinase 3 | [91807](https://www.ncbi.nlm.nih.gov/gene/?term=91807) | 2.1 | 0.01845 | 19.0 | 7.0 |
| BAMBI | BMP and activin membrane-bound inhibitor | [25805](https://www.ncbi.nlm.nih.gov/gene/?term=25805) | 2.1 | 0.00638 | 77.6 | 31.9 |
| VEGFB | vascular endothelial growth factor B | [7423](https://www.ncbi.nlm.nih.gov/gene/?term=7423) | -2.1 | 0.00030 | 20.0 | 46.3 |
| SAMD12 | sterile alpha motif domain containing 12 | [401474](https://www.ncbi.nlm.nih.gov/gene/?term=401474) | -2.1 | 0.00319 | 66.9 | 157.8 |
| RAB3IL1 | RAB3A interacting protein like 1 | [5866](https://www.ncbi.nlm.nih.gov/gene/?term=5866) | -2.1 | 0.00415 | 112.7 | 266.4 |
| LAMP1 | lysosomal associated membrane protein 1 | [3916](https://www.ncbi.nlm.nih.gov/gene/?term=3916) | 2.1 | 0.00000 | 3639.2 | 1704.4 |
| RAB39A | RAB39A, member RAS oncogene family | [54734](https://www.ncbi.nlm.nih.gov/gene/?term=54734) | 2.1 | 0.00015 | 177.4 | 80.4 |
| PDE2A | phosphodiesterase 2A | [5138](https://www.ncbi.nlm.nih.gov/gene/?term=5138) | -2.1 | 0.00422 | 81.9 | 192.7 |
| ACVRL1 | activin A receptor like type 1 | [94](https://www.ncbi.nlm.nih.gov/gene/?term=94) | 2.1 | 0.00322 | 39.7 | 16.6 |
| ST3GAL1 | ST3 beta-galactoside alpha-2,3-sialyltransferase 1 | [6482](https://www.ncbi.nlm.nih.gov/gene/?term=6482) | -2.0 | 0.00031 | 3788.9 | 8338.6 |
| LOC100421597 | peptidylprolyl isomerase like 4 pseudogene | [100421597](https://www.ncbi.nlm.nih.gov/gene/?term=100421597) | -2.0 | 0.00595 | 21.3 | 52.4 |
| PCAT1 | prostate cancer associated transcript 1 (non-protein coding) | [100750225](https://www.ncbi.nlm.nih.gov/gene/?term=100750225) | -2.0 | 0.00934 | 5.3 | 13.3 |
| IER2 | immediate early response 2 | [9592](https://www.ncbi.nlm.nih.gov/gene/?term=9592) | 2.0 | 0.00000 | 1346.4 | 639.0 |
| LOC105378753 |  | [105378753](https://www.ncbi.nlm.nih.gov/gene/?term=105378753) | 2.0 | 0.01010 | 18.1 | 7.4 |
| LOC107983982 |  | [107983982](https://www.ncbi.nlm.nih.gov/gene/?term=107983982) | -2.0 | 0.03585 | 1.3 | 5.0 |
| RNF138P1 | ring finger protein 138 pseudogene 1 | [379013](https://www.ncbi.nlm.nih.gov/gene/?term=379013) | -2.0 | 0.00347 | 22.3 | 53.0 |
| CHSY1 | chondroitin sulfate synthase 1 | [22856](https://www.ncbi.nlm.nih.gov/gene/?term=22856) | 2.0 | 0.00010 | 3019.1 | 1387.9 |
| GLRX | glutaredoxin (thioltransferase) | [2745](https://www.ncbi.nlm.nih.gov/gene/?term=2745) | 2.0 | 0.00000 | 3202.5 | 1504.7 |
| HCK | HCK proto-oncogene, Src family tyrosine kinase | [3055](https://www.ncbi.nlm.nih.gov/gene/?term=3055) | 2.0 | 0.00006 | 15902.3 | 7336.8 |
| CCNA2 | cyclin A2 | [890](https://www.ncbi.nlm.nih.gov/gene/?term=890) | 2.0 | 0.00604 | 333.3 | 139.1 |
| STMN1P1 | stathmin 1 pseudogene 1 | [100506685](https://www.ncbi.nlm.nih.gov/gene/?term=100506685) | -2.0 | 0.00526 | 16.7 | 40.4 |
| NAF1 | nuclear assembly factor 1 ribonucleoprotein | [92345](https://www.ncbi.nlm.nih.gov/gene/?term=92345) | -2.0 | 0.00000 | 223.6 | 477.3 |
| TMEM60 | transmembrane protein 60 | [85025](https://www.ncbi.nlm.nih.gov/gene/?term=85025) | 2.0 | 0.00000 | 465.1 | 220.9 |
| UBE2J1 | ubiquitin conjugating enzyme E2 J1 | [51465](https://www.ncbi.nlm.nih.gov/gene/?term=51465) | 2.0 | 0.00001 | 8073.2 | 3779.2 |
| TLR2 | toll like receptor 2 | [7097](https://www.ncbi.nlm.nih.gov/gene/?term=7097) | 2.0 | 0.00013 | 16842.8 | 7716.7 |
| LOC105369653 |  | [105369653](https://www.ncbi.nlm.nih.gov/gene/?term=105369653) | -2.0 | 0.01479 | 5.4 | 15.0 |
| CLEC11A | C-type lectin domain family 11 member A | [6320](https://www.ncbi.nlm.nih.gov/gene/?term=6320) | 2.0 | 0.00383 | 33.1 | 14.0 |
| HMGB3 | high mobility group box 3 | [3149](https://www.ncbi.nlm.nih.gov/gene/?term=3149) | 2.0 | 0.00190 | 95.9 | 42.0 |
| ACOT9 | acyl-CoA thioesterase 9 | [23597](https://www.ncbi.nlm.nih.gov/gene/?term=23597) | 2.0 | 0.00000 | 1555.7 | 736.6 |
| GNG10 | G protein subunit gamma 10 | [2790](https://www.ncbi.nlm.nih.gov/gene/?term=2790) | 2.0 | 0.00638 | 11.9 | 5.3 |
| ST13 | suppression of tumorigenicity 13 (colon carcinoma) (Hsp70 interacting protein) | [6767](https://www.ncbi.nlm.nih.gov/gene/?term=6767) | -2.0 | 0.00091 | 5795.3 | 12906.8 |
| KLF8 | Kruppel-like factor 8 | [11279](https://www.ncbi.nlm.nih.gov/gene/?term=11279) | -2.0 | 0.00016 | 83.3 | 181.2 |
| LOC105375902 |  | [105375902](https://www.ncbi.nlm.nih.gov/gene/?term=105375902) | -2.0 | 0.00246 | 22.9 | 53.9 |
| AUTS2 | autism susceptibility candidate 2 | [26053](https://www.ncbi.nlm.nih.gov/gene/?term=26053) | -2.0 | 0.00266 | 110.6 | 256.0 |
| PLAU | plasminogen activator, urokinase | [5328](https://www.ncbi.nlm.nih.gov/gene/?term=5328) | 2.0 | 0.01134 | 37.0 | 14.9 |
| PLP2 | proteolipid protein 2 (colonic epithelium-enriched) | [5355](https://www.ncbi.nlm.nih.gov/gene/?term=5355) | 2.0 | 0.00000 | 1071.6 | 505.8 |
| NBN | nibrin | [4683](https://www.ncbi.nlm.nih.gov/gene/?term=4683) | 2.0 | 0.00000 | 6335.4 | 2999.9 |
| NDUFB3 | NADH:ubiquinone oxidoreductase subunit B3 | [4709](https://www.ncbi.nlm.nih.gov/gene/?term=4709) | 2.0 | 0.00003 | 1190.9 | 554.3 |
| TSKS | testis specific serine kinase substrate | [60385](https://www.ncbi.nlm.nih.gov/gene/?term=60385) | 2.0 | 0.01468 | 34.3 | 13.3 |
| SUMO4 | small ubiquitin-like modifier 4 | [387082](https://www.ncbi.nlm.nih.gov/gene/?term=387082) | -2.0 | 0.00143 | 34.1 | 77.3 |
| LOC105369904 |  | [105369904](https://www.ncbi.nlm.nih.gov/gene/?term=105369904) | -2.0 | 0.00145 | 7.0 | 15.9 |
| LOC441666 | zinc finger protein 91 pseudogene | [441666](https://www.ncbi.nlm.nih.gov/gene/?term=441666) | -2.0 | 0.01282 | 16.2 | 41.8 |
| UPB1 | ureidopropionase, beta | [51733](https://www.ncbi.nlm.nih.gov/gene/?term=51733) | 2.0 | 0.00379 | 81.4 | 35.5 |
| LOC100130550 | g2/M phase-specific E3 ubiquitin-protein ligase-like | [100130550](https://www.ncbi.nlm.nih.gov/gene/?term=100130550) | -2.0 | 0.01011 | 6.3 | 16.5 |
| METRNL | meteorin, glial cell differentiation regulator-like | [284207](https://www.ncbi.nlm.nih.gov/gene/?term=284207) | 2.0 | 0.00007 | 157.6 | 72.9 |
| LOC100288846 | uncharacterized LOC100288846 | [100288846](https://www.ncbi.nlm.nih.gov/gene/?term=100288846) | 2.0 | 0.00048 | 22.7 | 10.1 |
| LOC107985949 |  | [107985949](https://www.ncbi.nlm.nih.gov/gene/?term=107985949) | -2.0 | 0.00269 | 242.5 | 558.4 |
| TNIP1 | TNFAIP3 interacting protein 1 | [10318](https://www.ncbi.nlm.nih.gov/gene/?term=10318) | -2.0 | 0.00005 | 3339.8 | 7146.1 |
| NFKBIA | NFKB inhibitor alpha | [4792](https://www.ncbi.nlm.nih.gov/gene/?term=4792) | 2.0 | 0.00000 | 5025.5 | 2392.4 |
| PWAR5 | Prader Willi/Angelman region RNA 5 | [8123](https://www.ncbi.nlm.nih.gov/gene/?term=8123) | -2.0 | 0.00014 | 167.7 | 365.0 |
| EIF2AK1 | eukaryotic translation initiation factor 2 alpha kinase 1 | [27102](https://www.ncbi.nlm.nih.gov/gene/?term=27102) | -2.0 | 0.00454 | 11950.6 | 29145.5 |
| RN7SL481P | RNA, 7SL, cytoplasmic 481, pseudogene | [106481054](https://www.ncbi.nlm.nih.gov/gene/?term=106481054) | -2.0 | 0.01207 | 6.7 | 18.0 |
| ADAM20 | ADAM metallopeptidase domain 20 | [8748](https://www.ncbi.nlm.nih.gov/gene/?term=8748) | -2.0 | 0.00471 | 56.8 | 134.2 |
| FES | FES proto-oncogene, tyrosine kinase | [2242](https://www.ncbi.nlm.nih.gov/gene/?term=2242) | 2.0 | 0.00003 | 3257.5 | 1527.2 |
| CXorf21 | chromosome X open reading frame 21 | [80231](https://www.ncbi.nlm.nih.gov/gene/?term=80231) | 2.0 | 0.00001 | 1819.0 | 857.1 |
| COL8A2 | collagen type VIII alpha 2 | [1296](https://www.ncbi.nlm.nih.gov/gene/?term=1296) | 2.0 | 0.00018 | 77.9 | 35.7 |
| HYMAI | hydatidiform mole associated and imprinted (non-protein coding) | [57061](https://www.ncbi.nlm.nih.gov/gene/?term=57061) | -2.0 | 0.01400 | 9.7 | 25.1 |
| RORC | RAR related orphan receptor C | [6097](https://www.ncbi.nlm.nih.gov/gene/?term=6097) | -2.0 | 0.01143 | 18.7 | 47.3 |
| DBN1 | drebrin 1 | [1627](https://www.ncbi.nlm.nih.gov/gene/?term=1627) | 2.0 | 0.00019 | 166.7 | 77.0 |
| LINC01359 | long intergenic non-protein coding RNA 1359 | [101927084](https://www.ncbi.nlm.nih.gov/gene/?term=101927084) | -2.0 | 0.00454 | 39.3 | 92.0 |
| LOC105376033 |  | [105376033](https://www.ncbi.nlm.nih.gov/gene/?term=105376033) | -2.0 | 0.00002 | 89.5 | 189.9 |
| KIF20A | kinesin family member 20A | [10112](https://www.ncbi.nlm.nih.gov/gene/?term=10112) | 2.0 | 0.00744 | 126.5 | 53.1 |
| TNFRSF14 | tumor necrosis factor receptor superfamily member 14 | [8764](https://www.ncbi.nlm.nih.gov/gene/?term=8764) | 2.0 | 0.00000 | 43.5 | 20.4 |
| LOC400499 |  | [400499](https://www.ncbi.nlm.nih.gov/gene/?term=400499) | 2.0 | 0.00385 | 1228.4 | 535.4 |
| LINC00482 | long intergenic non-protein coding RNA 482 | [284185](https://www.ncbi.nlm.nih.gov/gene/?term=284185) | 2.0 | 0.00982 | 23.7 | 9.6 |
| THSD7A | thrombospondin type 1 domain containing 7A | [221981](https://www.ncbi.nlm.nih.gov/gene/?term=221981) | -2.0 | 0.00926 | 10.6 | 25.8 |
| STAT1 | signal transducer and activator of transcription 1 | [6772](https://www.ncbi.nlm.nih.gov/gene/?term=6772) | 2.0 | 0.00078 | 23802.3 | 10821.0 |
| CAMK1D | calcium/calmodulin dependent protein kinase ID | [57118](https://www.ncbi.nlm.nih.gov/gene/?term=57118) | -2.0 | 0.00031 | 1273.2 | 2766.3 |
| UPK3A | uroplakin 3A | [7380](https://www.ncbi.nlm.nih.gov/gene/?term=7380) | 2.0 | 0.00451 | 26.5 | 11.3 |
| BCL11A | B-cell CLL/lymphoma 11A | [53335](https://www.ncbi.nlm.nih.gov/gene/?term=53335) | -2.0 | 0.00012 | 277.0 | 594.2 |
| P3H2 | prolyl 3-hydroxylase 2 | [55214](https://www.ncbi.nlm.nih.gov/gene/?term=55214) | -2.0 | 0.00749 | 6.9 | 16.3 |
| STOML1 | stomatin like 1 | [9399](https://www.ncbi.nlm.nih.gov/gene/?term=9399) | 2.0 | 0.00000 | 179.5 | 85.6 |
| RGS6 | regulator of G-protein signaling 6 | [9628](https://www.ncbi.nlm.nih.gov/gene/?term=9628) | -2.0 | 0.00468 | 375.7 | 871.3 |
| ITGA1 | integrin subunit alpha 1 | [3672](https://www.ncbi.nlm.nih.gov/gene/?term=3672) | 2.0 | 0.00026 | 435.2 | 201.5 |
| MS4A6A | membrane spanning 4-domains A6A | [64231](https://www.ncbi.nlm.nih.gov/gene/?term=64231) | 2.0 | 0.00002 | 5243.6 | 2470.7 |
| CD79A | CD79a molecule | [973](https://www.ncbi.nlm.nih.gov/gene/?term=973) | -2.0 | 0.00023 | 176.8 | 382.2 |
| FCGR3B | Fc fragment of IgG receptor IIIb | [2215](https://www.ncbi.nlm.nih.gov/gene/?term=2215) | 2.0 | 0.02364 | 101939.1 | 37227.9 |
| LGALS2 | lectin, galactoside binding soluble 2 | [3957](https://www.ncbi.nlm.nih.gov/gene/?term=3957) | 2.0 | 0.01028 | 634.5 | 262.8 |
| EIF4EBP2 | eukaryotic translation initiation factor 4E binding protein 2 | [1979](https://www.ncbi.nlm.nih.gov/gene/?term=1979) | -2.0 | 0.00001 | 3250.6 | 6816.7 |
| FAM129B | family with sequence similarity 129 member B | [64855](https://www.ncbi.nlm.nih.gov/gene/?term=64855) | 2.0 | 0.00003 | 797.1 | 376.7 |
| IL10RB | interleukin 10 receptor subunit beta | [3588](https://www.ncbi.nlm.nih.gov/gene/?term=3588) | 2.0 | 0.00005 | 3414.7 | 1613.1 |
| LOC105377019 |  | [105377019](https://www.ncbi.nlm.nih.gov/gene/?term=105377019) | -2.0 | 0.00833 | 3.4 | 8.9 |
| LOC107986364 |  | [107986364](https://www.ncbi.nlm.nih.gov/gene/?term=107986364) | -2.0 | 0.00273 | 62.7 | 143.6 |
| TMEM140 | transmembrane protein 140 | [55281](https://www.ncbi.nlm.nih.gov/gene/?term=55281) | 2.0 | 0.00014 | 6044.3 | 2830.2 |
| LOC107987163 |  | [107987163](https://www.ncbi.nlm.nih.gov/gene/?term=107987163) | -2.0 | 0.00495 | 41.7 | 97.9 |
| IRF1 | interferon regulatory factor 1 | [3659](https://www.ncbi.nlm.nih.gov/gene/?term=3659) | 2.0 | 0.00005 | 15959.7 | 7535.2 |
| NT5DC4 |  | [284958](https://www.ncbi.nlm.nih.gov/gene/?term=284958) | 2.0 | 0.00968 | 20.2 | 8.7 |
| RAB30 | RAB30, member RAS oncogene family | [27314](https://www.ncbi.nlm.nih.gov/gene/?term=27314) | -2.0 | 0.00003 | 376.8 | 796.1 |
| SDHAF3 | succinate dehydrogenase complex assembly factor 3 | [57001](https://www.ncbi.nlm.nih.gov/gene/?term=57001) | 2.0 | 0.00021 | 250.3 | 116.3 |
| LOC105374811 |  | [105374811](https://www.ncbi.nlm.nih.gov/gene/?term=105374811) | -2.0 | 0.00851 | 6.2 | 14.6 |
| LGALS1 | lectin, galactoside binding soluble 1 | [3956](https://www.ncbi.nlm.nih.gov/gene/?term=3956) | 2.0 | 0.00003 | 3095.7 | 1469.8 |
| KCNK13 | potassium two pore domain channel subfamily K member 13 | [56659](https://www.ncbi.nlm.nih.gov/gene/?term=56659) | 2.0 | 0.00235 | 44.1 | 19.4 |
| IFI16 | interferon gamma inducible protein 16 | [3428](https://www.ncbi.nlm.nih.gov/gene/?term=3428) | 2.0 | 0.00046 | 10120.3 | 4682.7 |
| DUSP3 | dual specificity phosphatase 3 | [1845](https://www.ncbi.nlm.nih.gov/gene/?term=1845) | 2.0 | 0.00001 | 1809.1 | 864.7 |
| PAQR6 | progestin and adipoQ receptor family member 6 | [79957](https://www.ncbi.nlm.nih.gov/gene/?term=79957) | 2.0 | 0.00355 | 44.7 | 20.0 |
| LOC100289511 | uncharacterized LOC100289511 | [100289511](https://www.ncbi.nlm.nih.gov/gene/?term=100289511) | 2.0 | 0.01051 | 8.4 | 3.4 |
| LOC101928891 | uncharacterized LOC101928891 | [101928891](https://www.ncbi.nlm.nih.gov/gene/?term=101928891) | 2.0 | 0.00093 | 17.9 | 8.1 |
| CATIP-AS1 | CATIP antisense RNA 1 | [101928513](https://www.ncbi.nlm.nih.gov/gene/?term=101928513) | 2.0 | 0.00492 | 26.7 | 11.5 |
| SP2 | Sp2 transcription factor | [6668](https://www.ncbi.nlm.nih.gov/gene/?term=6668) | -2.0 | 0.00038 | 85.3 | 184.8 |
| LOC102724015 |  | [102724015](https://www.ncbi.nlm.nih.gov/gene/?term=102724015) | 2.0 | 0.00330 | 11.1 | 4.9 |
| SAMD9L | sterile alpha motif domain containing 9 like | [219285](https://www.ncbi.nlm.nih.gov/gene/?term=219285) | 2.0 | 0.00341 | 19928.1 | 8865.7 |
| PANX2 | pannexin 2 | [56666](https://www.ncbi.nlm.nih.gov/gene/?term=56666) | -2.0 | 0.00353 | 30.1 | 70.0 |
| FTH1 | ferritin, heavy polypeptide 1 | [2495](https://www.ncbi.nlm.nih.gov/gene/?term=2495) | -2.0 | 0.00059 | 12863.7 | 27707.3 |
| HEXIM1 | hexamethylene bis-acetamide inducible 1 | [10614](https://www.ncbi.nlm.nih.gov/gene/?term=10614) | -2.0 | 0.00054 | 1492.6 | 3230.1 |
| LACTB | lactamase beta | [114294](https://www.ncbi.nlm.nih.gov/gene/?term=114294) | 2.0 | 0.00000 | 1421.9 | 683.5 |
| LOC440434 | aminopeptidase puromycin sensitive pseudogene | [440434](https://www.ncbi.nlm.nih.gov/gene/?term=440434) | -2.0 | 0.00205 | 16.4 | 36.6 |
| CIZ1 | CDKN1A interacting zinc finger protein 1 | [25792](https://www.ncbi.nlm.nih.gov/gene/?term=25792) | -2.0 | 0.00071 | 200.5 | 436.1 |
| LOC102724591 |  | [102724591](https://www.ncbi.nlm.nih.gov/gene/?term=102724591) | 2.0 | 0.00113 | 32.5 | 15.1 |
| ADD1 | adducin 1 | [118](https://www.ncbi.nlm.nih.gov/gene/?term=118) | -2.0 | 0.00015 | 3476.2 | 7421.1 |
| UBE3B | ubiquitin protein ligase E3B | [89910](https://www.ncbi.nlm.nih.gov/gene/?term=89910) | -2.0 | 0.00002 | 1501.3 | 3154.0 |
| RTKN2 | rhotekin 2 | [219790](https://www.ncbi.nlm.nih.gov/gene/?term=219790) | -2.0 | 0.00038 | 201.4 | 435.3 |
| LOC107986211 |  | [107986211](https://www.ncbi.nlm.nih.gov/gene/?term=107986211) | -2.0 | 0.01509 | 12.6 | 32.8 |
| PTK2B | protein tyrosine kinase 2 beta | [2185](https://www.ncbi.nlm.nih.gov/gene/?term=2185) | -2.0 | 0.00008 | 1984.7 | 4208.4 |
| LINC00264 | long intergenic non-protein coding RNA 264 | [645528](https://www.ncbi.nlm.nih.gov/gene/?term=645528) | 2.0 | 0.00474 | 20.5 | 9.1 |
| LOC101927330 |  | [101927330](https://www.ncbi.nlm.nih.gov/gene/?term=101927330) | 2.0 | 0.00195 | 21.6 | 9.4 |
| SUGT1P2 | SGT1 homolog, MIS12 kinetochore complex assembly cochaperone pseudogene 2 | [728706](https://www.ncbi.nlm.nih.gov/gene/?term=728706) | -2.0 | 0.01925 | 6.2 | 17.0 |
| ZEB2-AS1 | ZEB2 antisense RNA 1 | [100303491](https://www.ncbi.nlm.nih.gov/gene/?term=100303491) | 2.0 | 0.00402 | 19.7 | 8.1 |
| LOC101928361 |  | [101928361](https://www.ncbi.nlm.nih.gov/gene/?term=101928361) | -2.0 | 0.00449 | 237.1 | 545.2 |
| CEBPA | CCAAT/enhancer binding protein alpha | [1050](https://www.ncbi.nlm.nih.gov/gene/?term=1050) | 2.0 | 0.00001 | 1228.0 | 589.8 |
| PNRC1 | proline rich nuclear receptor coactivator 1 | [10957](https://www.ncbi.nlm.nih.gov/gene/?term=10957) | -2.0 | 0.00005 | 5255.4 | 10948.8 |
| LOC107986015 |  | [107986015](https://www.ncbi.nlm.nih.gov/gene/?term=107986015) | -2.0 | 0.00692 | 7.7 | 18.9 |
| ZGPAT | zinc finger CCCH-type and G-patch domain containing | [84619](https://www.ncbi.nlm.nih.gov/gene/?term=84619) | 2.0 | 0.00000 | 588.5 | 284.1 |
| PGD | phosphogluconate dehydrogenase | [5226](https://www.ncbi.nlm.nih.gov/gene/?term=5226) | 2.0 | 0.00025 | 8732.9 | 4087.7 |
| PDZD2 | PDZ domain containing 2 | [23037](https://www.ncbi.nlm.nih.gov/gene/?term=23037) | -2.0 | 0.00669 | 18.2 | 42.2 |
| DNASE1L1 | deoxyribonuclease I-like 1 | [1774](https://www.ncbi.nlm.nih.gov/gene/?term=1774) | 2.0 | 0.00000 | 1070.7 | 523.3 |
| HIST1H2AJ | histone cluster 1, H2aj | [8331](https://www.ncbi.nlm.nih.gov/gene/?term=8331) | 2.0 | 0.01947 | 482.2 | 187.1 |
| BTF3P16 | basic transcription factor 3 pseudogene 16 | [643412](https://www.ncbi.nlm.nih.gov/gene/?term=643412) | -2.0 | 0.00020 | 39.4 | 84.4 |
| TP53I3 | tumor protein p53 inducible protein 3 | [9540](https://www.ncbi.nlm.nih.gov/gene/?term=9540) | 2.0 | 0.00003 | 188.6 | 90.1 |
| ANKRD36B | ankyrin repeat domain 36B | [57730](https://www.ncbi.nlm.nih.gov/gene/?term=57730) | -2.0 | 0.00016 | 315.8 | 670.2 |
| MAPK3 | mitogen-activated protein kinase 3 | [5595](https://www.ncbi.nlm.nih.gov/gene/?term=5595) | 2.0 | 0.00001 | 942.4 | 453.1 |
| FCGR3A | Fc fragment of IgG receptor IIIa | [2214](https://www.ncbi.nlm.nih.gov/gene/?term=2214) | 2.0 | 0.00302 | 15948.9 | 7152.0 |
| ANKRD36C | ankyrin repeat domain 36C | [400986](https://www.ncbi.nlm.nih.gov/gene/?term=400986) | -2.0 | 0.00000 | 579.7 | 1195.2 |
| TAS2R63P | taste 2 receptor member 63 pseudogene | [338413](https://www.ncbi.nlm.nih.gov/gene/?term=338413) | -2.0 | 0.03473 | 4.1 | 14.4 |
| ATF7 | activating transcription factor 7 | [11016](https://www.ncbi.nlm.nih.gov/gene/?term=11016) | -2.0 | 0.00035 | 288.3 | 617.1 |
| DRAM1 | DNA damage regulated autophagy modulator 1 | [55332](https://www.ncbi.nlm.nih.gov/gene/?term=55332) | 2.0 | 0.00001 | 841.5 | 406.7 |
| LOC107985954 |  | [107985954](https://www.ncbi.nlm.nih.gov/gene/?term=107985954) | -2.0 | 0.00921 | 10.3 | 25.2 |
| MSRB1 | methionine sulfoxide reductase B1 | [51734](https://www.ncbi.nlm.nih.gov/gene/?term=51734) | 2.0 | 0.00011 | 5036.6 | 2392.0 |
| MFI2-AS1 | MFI2 antisense RNA 1 | [100507057](https://www.ncbi.nlm.nih.gov/gene/?term=100507057) | 2.0 | 0.02853 | 4.8 | 1.9 |
| C5orf56 | chromosome 5 open reading frame 56 | [441108](https://www.ncbi.nlm.nih.gov/gene/?term=441108) | 2.0 | 0.00015 | 512.2 | 242.2 |
| LOC105379346 |  | [105379346](https://www.ncbi.nlm.nih.gov/gene/?term=105379346) | -2.0 | 0.01572 | 4.7 | 12.6 |
| DGAT2 | diacylglycerol O-acyltransferase 2 | [84649](https://www.ncbi.nlm.nih.gov/gene/?term=84649) | 2.0 | 0.00135 | 4355.9 | 1998.9 |
| HIST1H2BI | histone cluster 1, H2bi | [8346](https://www.ncbi.nlm.nih.gov/gene/?term=8346) | 2.0 | 0.00494 | 938.9 | 413.5 |
| EDEM2 | ER degradation enhancing alpha-mannosidase like protein 2 | [55741](https://www.ncbi.nlm.nih.gov/gene/?term=55741) | 2.0 | 0.00000 | 1503.7 | 738.9 |
| WDTC1 | WD and tetratricopeptide repeats 1 | [23038](https://www.ncbi.nlm.nih.gov/gene/?term=23038) | -2.0 | 0.00022 | 2391.3 | 5064.4 |
| LOC105374219 |  | [105374219](https://www.ncbi.nlm.nih.gov/gene/?term=105374219) | 2.0 | 0.03871 | 11.0 | 3.3 |
| LOC101930023 |  | [101930023](https://www.ncbi.nlm.nih.gov/gene/?term=101930023) | -2.0 | 0.00103 | 13.3 | 28.9 |
| LOC105372194 |  | [105372194](https://www.ncbi.nlm.nih.gov/gene/?term=105372194) | 2.0 | 0.00042 | 21.6 | 9.6 |
| LOC105377148 |  | [105377148](https://www.ncbi.nlm.nih.gov/gene/?term=105377148) | -2.0 | 0.00740 | 32.4 | 75.6 |
| SCARNA20 | small Cajal body-specific RNA 20 | [677681](https://www.ncbi.nlm.nih.gov/gene/?term=677681) | -2.0 | 0.00280 | 7.9 | 17.6 |
| RPS6KA5 | ribosomal protein S6 kinase A5 | [9252](https://www.ncbi.nlm.nih.gov/gene/?term=9252) | -2.0 | 0.00000 | 677.9 | 1374.5 |
| CTDNEP1 | CTD nuclear envelope phosphatase 1 | [23399](https://www.ncbi.nlm.nih.gov/gene/?term=23399) | -2.0 | 0.00019 | 430.8 | 911.0 |
| APOL6 | apolipoprotein L6 | [80830](https://www.ncbi.nlm.nih.gov/gene/?term=80830) | 2.0 | 0.00269 | 14512.8 | 6557.6 |
| PPP1R12B | protein phosphatase 1 regulatory subunit 12B | [4660](https://www.ncbi.nlm.nih.gov/gene/?term=4660) | -2.0 | 0.00007 | 968.3 | 2017.9 |
| LOC102723654 |  | [102723654](https://www.ncbi.nlm.nih.gov/gene/?term=102723654) | -2.0 | 0.00051 | 21.2 | 47.2 |
| FAM174B | family with sequence similarity 174 member B | [400451](https://www.ncbi.nlm.nih.gov/gene/?term=400451) | 2.0 | 0.00573 | 17.0 | 7.5 |
| EIF1B-AS1 | EIF1B antisense RNA 1 | [440952](https://www.ncbi.nlm.nih.gov/gene/?term=440952) | 2.0 | 0.00074 | 59.4 | 27.6 |
| GLIPR2 | GLI pathogenesis related 2 | [152007](https://www.ncbi.nlm.nih.gov/gene/?term=152007) | 2.0 | 0.00001 | 6559.0 | 3181.1 |
| APMAP | adipocyte plasma membrane associated protein | [57136](https://www.ncbi.nlm.nih.gov/gene/?term=57136) | 2.0 | 0.00061 | 4652.3 | 2181.5 |
| GLTPP1 | glycolipid transfer protein pseudogene 1 | [645312](https://www.ncbi.nlm.nih.gov/gene/?term=645312) | 2.0 | 0.03242 | 7.6 | 2.5 |
| YPEL3 | yippee like 3 | [83719](https://www.ncbi.nlm.nih.gov/gene/?term=83719) | -2.0 | 0.00091 | 4422.0 | 9486.5 |
| DDX60L | DEAD-box helicase 60-like | [91351](https://www.ncbi.nlm.nih.gov/gene/?term=91351) | 2.0 | 0.00274 | 17596.7 | 8033.8 |
| PA2G4P4 | proliferation-associated 2G4 pseudogene 4 | [647033](https://www.ncbi.nlm.nih.gov/gene/?term=647033) | -2.0 | 0.00177 | 36.7 | 80.6 |
| TAS2R46 | taste 2 receptor member 46 | [259292](https://www.ncbi.nlm.nih.gov/gene/?term=259292) | -2.0 | 0.03689 | 2.5 | 9.4 |
| PNMA3 | paraneoplastic Ma antigen 3 | [29944](https://www.ncbi.nlm.nih.gov/gene/?term=29944) | 2.0 | 0.01048 | 33.3 | 14.2 |
| COL7A1 | collagen type VII alpha 1 | [1294](https://www.ncbi.nlm.nih.gov/gene/?term=1294) | 2.0 | 0.00080 | 53.0 | 24.9 |
| ZNF573 | zinc finger protein 573 | [126231](https://www.ncbi.nlm.nih.gov/gene/?term=126231) | -2.0 | 0.00000 | 116.3 | 234.4 |
| PEG10 | paternally expressed 10 | [23089](https://www.ncbi.nlm.nih.gov/gene/?term=23089) | -2.0 | 0.01266 | 16.7 | 40.5 |
| HIST1H3B | histone cluster 1, H3b | [8358](https://www.ncbi.nlm.nih.gov/gene/?term=8358) | 2.0 | 0.01204 | 1562.9 | 656.8 |
| LOC100289061 |  | [100289061](https://www.ncbi.nlm.nih.gov/gene/?term=100289061) | 2.0 | 0.00879 | 194.5 | 84.1 |
| LOC105376066 |  | [105376066](https://www.ncbi.nlm.nih.gov/gene/?term=105376066) | -2.0 | 0.01694 | 10.7 | 27.5 |
| EGR1 | early growth response 1 | [1958](https://www.ncbi.nlm.nih.gov/gene/?term=1958) | -2.0 | 0.00888 | 11.0 | 25.5 |
| FKSG29 | FKSG29 | [100131561](https://www.ncbi.nlm.nih.gov/gene/?term=100131561) | -2.0 | 0.00817 | 18.3 | 43.7 |
| NMI | N-myc and STAT interactor | [9111](https://www.ncbi.nlm.nih.gov/gene/?term=9111) | 2.0 | 0.00004 | 3926.1 | 1904.1 |
| HIST1H2AL | histone cluster 1, H2al | [8332](https://www.ncbi.nlm.nih.gov/gene/?term=8332) | 2.0 | 0.00631 | 609.5 | 268.1 |
| LOC107987260 |  | [107987260](https://www.ncbi.nlm.nih.gov/gene/?term=107987260) | -2.0 | 0.00438 | 318.1 | 714.2 |
| LGALS8-AS1 | LGALS8 antisense RNA 1 | [100287902](https://www.ncbi.nlm.nih.gov/gene/?term=100287902) | 2.0 | 0.00604 | 22.1 | 9.4 |
| LOC107985887 |  | [107985887](https://www.ncbi.nlm.nih.gov/gene/?term=107985887) | -2.0 | 0.00626 | 89.1 | 203.4 |
| TMOD1 | tropomodulin 1 | [7111](https://www.ncbi.nlm.nih.gov/gene/?term=7111) | -2.0 | 0.00693 | 5741.1 | 13293.2 |
| CMTR1 | cap methyltransferase 1 | [23070](https://www.ncbi.nlm.nih.gov/gene/?term=23070) | 2.0 | 0.00001 | 3207.1 | 1569.0 |
| MT1F | metallothionein 1F | [4494](https://www.ncbi.nlm.nih.gov/gene/?term=4494) | 2.0 | 0.00024 | 63.7 | 30.2 |
| PPM1M | protein phosphatase, Mg2+/Mn2+ dependent 1M | [132160](https://www.ncbi.nlm.nih.gov/gene/?term=132160) | 2.0 | 0.00000 | 2085.4 | 1030.6 |
| TRIM58 | tripartite motif containing 58 | [25893](https://www.ncbi.nlm.nih.gov/gene/?term=25893) | -2.0 | 0.00709 | 45495.6 | 114132.0 |
| SLC4A10 | solute carrier family 4 member 10 | [57282](https://www.ncbi.nlm.nih.gov/gene/?term=57282) | -2.0 | 0.00933 | 106.3 | 249.3 |
| USF1 | upstream transcription factor 1 | [7391](https://www.ncbi.nlm.nih.gov/gene/?term=7391) | 2.0 | 0.00000 | 3172.6 | 1581.7 |
| HMBOX1 | homeobox containing 1 | [79618](https://www.ncbi.nlm.nih.gov/gene/?term=79618) | -2.0 | 0.00021 | 523.1 | 1091.3 |
| SRGN | serglycin | [5552](https://www.ncbi.nlm.nih.gov/gene/?term=5552) | 2.0 | 0.00050 | 61022.2 | 28932.3 |
| LOC107985489 |  | [107985489](https://www.ncbi.nlm.nih.gov/gene/?term=107985489) | -2.0 | 0.01422 | 6.0 | 15.3 |
| LINC00677 |  | [105370683](https://www.ncbi.nlm.nih.gov/gene/?term=105370683) | 2.0 | 0.00450 | 32.1 | 14.4 |
| SIGLEC8 | sialic acid binding Ig like lectin 8 | [27181](https://www.ncbi.nlm.nih.gov/gene/?term=27181) | -2.0 | 0.02447 | 38.8 | 102.7 |
| C4orf3 | chromosome 4 open reading frame 3 | [401152](https://www.ncbi.nlm.nih.gov/gene/?term=401152) | 2.0 | 0.00003 | 4586.7 | 2244.5 |
| HIST1H3J | histone cluster 1, H3j | [8356](https://www.ncbi.nlm.nih.gov/gene/?term=8356) | 2.0 | 0.01759 | 671.6 | 273.1 |
| LOC105374981 |  | [105374981](https://www.ncbi.nlm.nih.gov/gene/?term=105374981) | 2.0 | 0.00077 | 168.4 | 79.8 |
| LOC105373218 |  | [105373218](https://www.ncbi.nlm.nih.gov/gene/?term=105373218) | -2.0 | 0.00768 | 14.7 | 34.0 |
| LOC107984278 |  | [107984278](https://www.ncbi.nlm.nih.gov/gene/?term=107984278) | -2.0 | 0.02805 | 10.1 | 28.8 |
| IDH1 | isocitrate dehydrogenase (NADP(+)) 1, cytosolic | [3417](https://www.ncbi.nlm.nih.gov/gene/?term=3417) | 2.0 | 0.00000 | 1600.0 | 789.4 |
| RPSAP6 | ribosomal protein SA pseudogene 6 | [414254](https://www.ncbi.nlm.nih.gov/gene/?term=414254) | -2.0 | 0.02556 | 7.9 | 21.5 |
| TANC2 | tetratricopeptide repeat, ankyrin repeat and coiled-coil containing 2 | [26115](https://www.ncbi.nlm.nih.gov/gene/?term=26115) | -2.0 | 0.00004 | 270.6 | 557.7 |
| LOC105370707 |  | [105370707](https://www.ncbi.nlm.nih.gov/gene/?term=105370707) | 2.0 | 0.00078 | 34.3 | 16.4 |
| GMPR2 | guanosine monophosphate reductase 2 | [51292](https://www.ncbi.nlm.nih.gov/gene/?term=51292) | 2.0 | 0.00000 | 2308.3 | 1146.3 |
| ANXA5 | annexin A5 | [308](https://www.ncbi.nlm.nih.gov/gene/?term=308) | 2.0 | 0.00000 | 5477.8 | 2698.2 |
| VAV1 | vav 1 guanine nucleotide exchange factor | [7409](https://www.ncbi.nlm.nih.gov/gene/?term=7409) | 2.0 | 0.00000 | 5522.4 | 2751.5 |
| BIK | BCL2 interacting killer | [638](https://www.ncbi.nlm.nih.gov/gene/?term=638) | 1.9 | 0.00291 | 83.9 | 38.3 |
| IL23R | interleukin 23 receptor | [149233](https://www.ncbi.nlm.nih.gov/gene/?term=149233) | -1.9 | 0.02413 | 15.8 | 42.1 |
| CEP19 | centrosomal protein 19 | [84984](https://www.ncbi.nlm.nih.gov/gene/?term=84984) | 1.9 | 0.00013 | 1379.8 | 668.8 |
| C19orf66 | chromosome 19 open reading frame 66 | [55337](https://www.ncbi.nlm.nih.gov/gene/?term=55337) | 1.9 | 0.00001 | 752.4 | 369.7 |
| MIR938 | microRNA 938 | [100126327](https://www.ncbi.nlm.nih.gov/gene/?term=100126327) | -1.9 | 0.03585 | 2.3 | 8.1 |
| AP2A1 | adaptor related protein complex 2 alpha 1 subunit | [160](https://www.ncbi.nlm.nih.gov/gene/?term=160) | -1.9 | 0.00616 | 3267.2 | 7274.1 |
| ARAP3 | ArfGAP with RhoGAP domain, ankyrin repeat and PH domain 3 | [64411](https://www.ncbi.nlm.nih.gov/gene/?term=64411) | -1.9 | 0.00143 | 146.0 | 314.4 |
| LOC100419170 | toll like receptor 2 pseudogene | [100419170](https://www.ncbi.nlm.nih.gov/gene/?term=100419170) | 1.9 | 0.01837 | 88.2 | 35.6 |
| RCAN3 | RCAN family member 3 | [11123](https://www.ncbi.nlm.nih.gov/gene/?term=11123) | -1.9 | 0.00007 | 346.2 | 713.9 |
| LOC105379752 |  | [105379752](https://www.ncbi.nlm.nih.gov/gene/?term=105379752) | -1.9 | 0.01872 | 54.4 | 136.9 |
| LOC105378005 |  | [105378005](https://www.ncbi.nlm.nih.gov/gene/?term=105378005) | -1.9 | 0.00982 | 21.0 | 49.7 |
| SLC44A2 | solute carrier family 44 member 2 | [57153](https://www.ncbi.nlm.nih.gov/gene/?term=57153) | -1.9 | 0.00002 | 1439.2 | 2926.0 |
| LOC105374187 |  | [105374187](https://www.ncbi.nlm.nih.gov/gene/?term=105374187) | -1.9 | 0.00058 | 27.9 | 60.5 |
| CRIPAK | cysteine rich PAK1 inhibitor | [285464](https://www.ncbi.nlm.nih.gov/gene/?term=285464) | -1.9 | 0.01967 | 31.4 | 80.0 |
| MT1X | metallothionein 1X | [4501](https://www.ncbi.nlm.nih.gov/gene/?term=4501) | 1.9 | 0.00005 | 128.2 | 61.8 |
| DCUN1D1 | defective in cullin neddylation 1 domain containing 1 | [54165](https://www.ncbi.nlm.nih.gov/gene/?term=54165) | -1.9 | 0.00235 | 20435.4 | 44904.5 |
| LOC100131689 | pumilio domain-containing protein KIAA0020-like | [100131689](https://www.ncbi.nlm.nih.gov/gene/?term=100131689) | -1.9 | 0.02363 | 15.3 | 40.4 |
| LDHAL6FP | lactate dehydrogenase A like 6F, pseudogene | [100420477](https://www.ncbi.nlm.nih.gov/gene/?term=100420477) | -1.9 | 0.02992 | 4.7 | 14.1 |
| AIDA | axin interactor, dorsalization associated | [64853](https://www.ncbi.nlm.nih.gov/gene/?term=64853) | -1.9 | 0.00094 | 3996.1 | 8485.5 |
| NMRAL1 | NmrA-like family domain containing 1 | [57407](https://www.ncbi.nlm.nih.gov/gene/?term=57407) | 1.9 | 0.00859 | 9.0 | 4.2 |
| LOC105370461 |  | [105370461](https://www.ncbi.nlm.nih.gov/gene/?term=105370461) | -1.9 | 0.02806 | 2.5 | 7.3 |
| LOC101927851 | uncharacterized LOC101927851 | [101927851](https://www.ncbi.nlm.nih.gov/gene/?term=101927851) | 1.9 | 0.00060 | 65.4 | 31.3 |
| MKRN4P | makorin ring finger protein 4, pseudogene | [7682](https://www.ncbi.nlm.nih.gov/gene/?term=7682) | -1.9 | 0.02557 | 25.0 | 65.5 |
| LINC00861 | long intergenic non-protein coding RNA 861 | [100130231](https://www.ncbi.nlm.nih.gov/gene/?term=100130231) | -1.9 | 0.00820 | 546.3 | 1246.1 |
| ZFP36L2 | ZFP36 ring finger protein-like 2 | [678](https://www.ncbi.nlm.nih.gov/gene/?term=678) | -1.9 | 0.00010 | 1674.0 | 3413.8 |
| S100A5 | S100 calcium binding protein A5 | [6276](https://www.ncbi.nlm.nih.gov/gene/?term=6276) | 1.9 | 0.00805 | 17.3 | 7.6 |
| CORO1B | coronin 1B | [57175](https://www.ncbi.nlm.nih.gov/gene/?term=57175) | 1.9 | 0.00000 | 1179.5 | 591.2 |
| STK24-AS1 |  | [100874069](https://www.ncbi.nlm.nih.gov/gene/?term=100874069) | 1.9 | 0.00031 | 38.0 | 17.8 |
| F8A1 | coagulation factor VIII-associated 1 | [8263](https://www.ncbi.nlm.nih.gov/gene/?term=8263) | 1.9 | 0.00338 | 54.1 | 24.6 |
| LOC105379420 |  | [105379420](https://www.ncbi.nlm.nih.gov/gene/?term=105379420) | -1.9 | 0.01478 | 22.1 | 53.7 |
| OR2B6 | olfactory receptor family 2 subfamily B member 6 | [26212](https://www.ncbi.nlm.nih.gov/gene/?term=26212) | 1.9 | 0.01560 | 53.0 | 22.3 |
| LOC653631 | axin interactor, dorsalization associated pseudogene | [653631](https://www.ncbi.nlm.nih.gov/gene/?term=653631) | -1.9 | 0.03474 | 62.0 | 215.3 |
| GPR146 | G protein-coupled receptor 146 | [115330](https://www.ncbi.nlm.nih.gov/gene/?term=115330) | -1.9 | 0.01293 | 1243.8 | 2957.8 |
| YWHAZP4 | tyrosine 3-monooxygenase/tryptophan 5-monooxygenase activation protein zeta pseudogene 4 | [246737](https://www.ncbi.nlm.nih.gov/gene/?term=246737) | -1.9 | 0.00814 | 6.5 | 15.3 |
| LINC00963 | long intergenic non-protein coding RNA 963 | [100506190](https://www.ncbi.nlm.nih.gov/gene/?term=100506190) | 1.9 | 0.00010 | 580.5 | 283.7 |
| KLF5 | Kruppel-like factor 5 (intestinal) | [688](https://www.ncbi.nlm.nih.gov/gene/?term=688) | 1.9 | 0.00146 | 144.0 | 67.6 |
| ENPP1 | ectonucleotide pyrophosphatase/phosphodiesterase 1 | [5167](https://www.ncbi.nlm.nih.gov/gene/?term=5167) | -1.9 | 0.01504 | 6.7 | 16.2 |
| BAIAP3 | BAI1 associated protein 3 | [8938](https://www.ncbi.nlm.nih.gov/gene/?term=8938) | -1.9 | 0.01014 | 69.2 | 160.6 |
| LINC01270 | long intergenic non-protein coding RNA 1270 | [284751](https://www.ncbi.nlm.nih.gov/gene/?term=284751) | 1.9 | 0.01312 | 105.1 | 44.9 |
| CDK12 | cyclin-dependent kinase 12 | [51755](https://www.ncbi.nlm.nih.gov/gene/?term=51755) | -1.9 | 0.00000 | 2400.1 | 4842.8 |
| RAB33B | RAB33B, member RAS oncogene family | [83452](https://www.ncbi.nlm.nih.gov/gene/?term=83452) | 1.9 | 0.00003 | 1447.8 | 714.2 |
| ARF1P2 | ADP ribosylation factor 1 pseudogene 2 | [100420012](https://www.ncbi.nlm.nih.gov/gene/?term=100420012) | -1.9 | 0.03662 | 3.9 | 13.0 |
| LOC105372343 |  | [105372343](https://www.ncbi.nlm.nih.gov/gene/?term=105372343) | 1.9 | 0.03674 | 13.7 | 4.5 |
| GIMAP4 | GTPase, IMAP family member 4 | [55303](https://www.ncbi.nlm.nih.gov/gene/?term=55303) | 1.9 | 0.00000 | 14897.4 | 7474.0 |
| LOC105378120 |  | [105378120](https://www.ncbi.nlm.nih.gov/gene/?term=105378120) | -1.9 | 0.00289 | 64.6 | 141.2 |
| ACTG1P3 | actin gamma 1 pseudogene 3 | [75](https://www.ncbi.nlm.nih.gov/gene/?term=75) | -1.9 | 0.01684 | 7.9 | 20.1 |
| FBXL5 | F-box and leucine-rich repeat protein 5 | [26234](https://www.ncbi.nlm.nih.gov/gene/?term=26234) | 1.9 | 0.00006 | 21627.0 | 10605.7 |
| SMG1P6 | SMG1 pseudogene 6 | [100422558](https://www.ncbi.nlm.nih.gov/gene/?term=100422558) | -1.9 | 0.00091 | 106.3 | 223.6 |
| SCAI | suppressor of cancer cell invasion | [286205](https://www.ncbi.nlm.nih.gov/gene/?term=286205) | -1.9 | 0.00000 | 219.6 | 439.6 |
| LOC105373656 | uncharacterized LOC105373656 | [105373656](https://www.ncbi.nlm.nih.gov/gene/?term=105373656) | 1.9 | 0.00522 | 9.9 | 4.7 |
| GNS | glucosamine (N-acetyl)-6-sulfatase | [2799](https://www.ncbi.nlm.nih.gov/gene/?term=2799) | 1.9 | 0.00000 | 11360.1 | 5706.9 |
| HAVCR2 | hepatitis A virus cellular receptor 2 | [84868](https://www.ncbi.nlm.nih.gov/gene/?term=84868) | 1.9 | 0.00000 | 988.0 | 495.0 |
| TSPO | translocator protein | [706](https://www.ncbi.nlm.nih.gov/gene/?term=706) | 1.9 | 0.00019 | 4809.4 | 2367.2 |
| MED25 | mediator complex subunit 25 | [81857](https://www.ncbi.nlm.nih.gov/gene/?term=81857) | -1.9 | 0.00043 | 927.2 | 1902.8 |
| MGC16275 | uncharacterized protein MGC16275 | [85001](https://www.ncbi.nlm.nih.gov/gene/?term=85001) | 1.9 | 0.00343 | 42.5 | 19.5 |
| CPEB4 | cytoplasmic polyadenylation element binding protein 4 | [80315](https://www.ncbi.nlm.nih.gov/gene/?term=80315) | -1.9 | 0.00175 | 10442.2 | 22081.4 |
| MBD5 | methyl-CpG binding domain protein 5 | [55777](https://www.ncbi.nlm.nih.gov/gene/?term=55777) | -1.9 | 0.00000 | 661.4 | 1312.3 |
| ZDHHC21 | zinc finger DHHC-type containing 21 | [340481](https://www.ncbi.nlm.nih.gov/gene/?term=340481) | -1.9 | 0.00006 | 660.3 | 1334.4 |
| LOC105373582 |  | [105373582](https://www.ncbi.nlm.nih.gov/gene/?term=105373582) | 1.9 | 0.03579 | 137.7 | 46.9 |
| SART1 | squamous cell carcinoma antigen recognized by T-cells 1 | [9092](https://www.ncbi.nlm.nih.gov/gene/?term=9092) | 1.9 | 0.00000 | 1304.5 | 668.8 |
| LOC107986874 |  | [107986874](https://www.ncbi.nlm.nih.gov/gene/?term=107986874) | -1.9 | 0.00414 | 141.5 | 307.8 |
| STAT5A | signal transducer and activator of transcription 5A | [6776](https://www.ncbi.nlm.nih.gov/gene/?term=6776) | 1.9 | 0.00000 | 3418.6 | 1727.8 |
| UBE2V1 | ubiquitin conjugating enzyme E2 V1 | [7335](https://www.ncbi.nlm.nih.gov/gene/?term=7335) | -1.9 | 0.00001 | 77.6 | 156.3 |
| RILPL2 | Rab interacting lysosomal protein like 2 | [196383](https://www.ncbi.nlm.nih.gov/gene/?term=196383) | 1.9 | 0.00000 | 1737.4 | 875.8 |
| LOC107986515 |  | [107986515](https://www.ncbi.nlm.nih.gov/gene/?term=107986515) | -1.9 | 0.01642 | 30.7 | 74.0 |
| RFPL2 | ret finger protein like 2 | [10739](https://www.ncbi.nlm.nih.gov/gene/?term=10739) | -1.9 | 0.01010 | 17.8 | 39.4 |
| JUND | JunD proto-oncogene, AP-1 transcription factor subunit | [3727](https://www.ncbi.nlm.nih.gov/gene/?term=3727) | -1.9 | 0.00353 | 2758.1 | 5942.5 |
| SLC38A5 | solute carrier family 38 member 5 | [92745](https://www.ncbi.nlm.nih.gov/gene/?term=92745) | -1.9 | 0.00673 | 577.4 | 1275.1 |
| SRGAP2 | SLIT-ROBO Rho GTPase activating protein 2 | [23380](https://www.ncbi.nlm.nih.gov/gene/?term=23380) | -1.9 | 0.00179 | 907.0 | 1908.6 |
| PARP15 | poly(ADP-ribose) polymerase family member 15 | [165631](https://www.ncbi.nlm.nih.gov/gene/?term=165631) | -1.9 | 0.00215 | 492.8 | 1047.7 |
| SLC11A1 | solute carrier family 11 member 1 | [6556](https://www.ncbi.nlm.nih.gov/gene/?term=6556) | 1.9 | 0.00104 | 4853.3 | 2325.3 |
| LOC105375112 |  | [105375112](https://www.ncbi.nlm.nih.gov/gene/?term=105375112) | 1.9 | 0.00525 | 151.5 | 69.9 |
| GZMH | granzyme H | [2999](https://www.ncbi.nlm.nih.gov/gene/?term=2999) | 1.9 | 0.01527 | 868.1 | 369.0 |
| ADAP1 | ArfGAP with dual PH domains 1 | [11033](https://www.ncbi.nlm.nih.gov/gene/?term=11033) | 1.9 | 0.00000 | 1016.8 | 517.1 |
| IDS | iduronate 2-sulfatase | [3423](https://www.ncbi.nlm.nih.gov/gene/?term=3423) | -1.9 | 0.00131 | 1943.5 | 4064.1 |
| PRSS36 | protease, serine 36 | [146547](https://www.ncbi.nlm.nih.gov/gene/?term=146547) | 1.9 | 0.00019 | 38.3 | 18.3 |
| CBR1 | carbonyl reductase 1 | [873](https://www.ncbi.nlm.nih.gov/gene/?term=873) | 1.9 | 0.00001 | 669.8 | 336.9 |
| DOK1 | docking protein 1 | [1796](https://www.ncbi.nlm.nih.gov/gene/?term=1796) | 1.9 | 0.00000 | 731.3 | 371.2 |
| LOC105378077 |  | [105378077](https://www.ncbi.nlm.nih.gov/gene/?term=105378077) | -1.9 | 0.01403 | 11.1 | 26.4 |
| NFKBIE | NFKB inhibitor epsilon | [4794](https://www.ncbi.nlm.nih.gov/gene/?term=4794) | 1.9 | 0.00000 | 782.8 | 397.4 |
| JAG1 | jagged 1 | [182](https://www.ncbi.nlm.nih.gov/gene/?term=182) | 1.9 | 0.00006 | 305.4 | 152.0 |
| RPL7P49 | ribosomal protein L7 pseudogene 49 | [100271221](https://www.ncbi.nlm.nih.gov/gene/?term=100271221) | -1.9 | 0.02703 | 7.3 | 19.7 |
| LOC107987020 |  | [107987020](https://www.ncbi.nlm.nih.gov/gene/?term=107987020) | 1.9 | 0.00681 | 44.1 | 20.2 |
| TNFSF13 | tumor necrosis factor superfamily member 13 | [8741](https://www.ncbi.nlm.nih.gov/gene/?term=8741) | 1.9 | 0.00004 | 193.8 | 96.5 |
| LOC107987242 |  | [107987242](https://www.ncbi.nlm.nih.gov/gene/?term=107987242) | -1.9 | 0.00003 | 265.7 | 531.0 |
| CASC3 | cancer susceptibility candidate 3 | [22794](https://www.ncbi.nlm.nih.gov/gene/?term=22794) | -1.9 | 0.00007 | 2214.7 | 4454.5 |
| PDZK1IP1 | PDZK1 interacting protein 1 | [10158](https://www.ncbi.nlm.nih.gov/gene/?term=10158) | -1.9 | 0.01324 | 724.6 | 1681.4 |
| HIST1H1B | histone cluster 1, H1b | [3009](https://www.ncbi.nlm.nih.gov/gene/?term=3009) | 1.9 | 0.01622 | 2703.9 | 1147.7 |
| TBL1XR1 | transducin (beta)-like 1 X-linked receptor 1 | [79718](https://www.ncbi.nlm.nih.gov/gene/?term=79718) | -1.9 | 0.00008 | 10411.6 | 20902.4 |
| CNOT6 | CCR4-NOT transcription complex subunit 6 | [57472](https://www.ncbi.nlm.nih.gov/gene/?term=57472) | -1.9 | 0.00022 | 2676.1 | 5444.7 |
| TRIM7 | tripartite motif containing 7 | [81786](https://www.ncbi.nlm.nih.gov/gene/?term=81786) | 1.9 | 0.00273 | 47.0 | 22.0 |
| RPL12P27 | ribosomal protein L12 pseudogene 27 | [644202](https://www.ncbi.nlm.nih.gov/gene/?term=644202) | 1.9 | 0.01330 | 13.2 | 6.1 |
| LOC105371711 |  | [105371711](https://www.ncbi.nlm.nih.gov/gene/?term=105371711) | -1.9 | 0.02606 | 5.9 | 15.0 |
| LOC105378808 |  | [105378808](https://www.ncbi.nlm.nih.gov/gene/?term=105378808) | -1.9 | 0.01133 | 12.6 | 29.8 |
| TRNL1 |  | [4567](https://www.ncbi.nlm.nih.gov/gene/?term=4567) | -1.9 | 0.00186 | 174.8 | 362.9 |
| FAM229B | family with sequence similarity 229 member B | [619208](https://www.ncbi.nlm.nih.gov/gene/?term=619208) | -1.9 | 0.00344 | 10.9 | 23.3 |
| LINC01004 | long intergenic non-protein coding RNA 1004 | [100216546](https://www.ncbi.nlm.nih.gov/gene/?term=100216546) | -1.9 | 0.00314 | 280.8 | 597.0 |
| CRIP3 | cysteine rich protein 3 | [401262](https://www.ncbi.nlm.nih.gov/gene/?term=401262) | -1.9 | 0.00286 | 22.3 | 48.0 |
| ZNF860 | zinc finger protein 860 | [344787](https://www.ncbi.nlm.nih.gov/gene/?term=344787) | -1.9 | 0.00249 | 150.6 | 316.5 |
| LINC01547 | long intergenic non-protein coding RNA 1547 | [84536](https://www.ncbi.nlm.nih.gov/gene/?term=84536) | 1.9 | 0.00719 | 9.4 | 4.6 |
| NMNAT3 | nicotinamide nucleotide adenylyltransferase 3 | [349565](https://www.ncbi.nlm.nih.gov/gene/?term=349565) | -1.9 | 0.00167 | 82.1 | 170.8 |
| KIAA0101 | KIAA0101 | [9768](https://www.ncbi.nlm.nih.gov/gene/?term=9768) | 1.9 | 0.00862 | 130.3 | 59.0 |
| LOC101929243 | uncharacterized LOC101929243 | [101929243](https://www.ncbi.nlm.nih.gov/gene/?term=101929243) | 1.9 | 0.00011 | 62.6 | 30.8 |
| CCDC141 | coiled-coil domain containing 141 | [285025](https://www.ncbi.nlm.nih.gov/gene/?term=285025) | -1.9 | 0.00133 | 186.5 | 387.0 |
| C1orf116 | chromosome 1 open reading frame 116 | [79098](https://www.ncbi.nlm.nih.gov/gene/?term=79098) | -1.9 | 0.01713 | 81.3 | 188.8 |
| HUWE1 | HECT, UBA and WWE domain containing 1, E3 ubiquitin protein ligase | [10075](https://www.ncbi.nlm.nih.gov/gene/?term=10075) | -1.9 | 0.00007 | 3705.2 | 7396.2 |
| FDXR | ferredoxin reductase | [2232](https://www.ncbi.nlm.nih.gov/gene/?term=2232) | 1.9 | 0.00032 | 143.9 | 71.3 |
| SEMA4A | semaphorin 4A | [64218](https://www.ncbi.nlm.nih.gov/gene/?term=64218) | 1.9 | 0.00007 | 2789.6 | 1401.0 |
| HIST1H2BL | histone cluster 1, H2bl | [8340](https://www.ncbi.nlm.nih.gov/gene/?term=8340) | 1.9 | 0.00914 | 434.0 | 196.5 |
| STX10 | syntaxin 10 | [8677](https://www.ncbi.nlm.nih.gov/gene/?term=8677) | 1.9 | 0.00000 | 2186.2 | 1121.5 |
| MS4A7 | membrane spanning 4-domains A7 | [58475](https://www.ncbi.nlm.nih.gov/gene/?term=58475) | 1.9 | 0.00036 | 2422.6 | 1196.8 |
| NRCAM | neuronal cell adhesion molecule | [4897](https://www.ncbi.nlm.nih.gov/gene/?term=4897) | -1.9 | 0.02944 | 40.1 | 106.2 |
| PCGF5 | polycomb group ring finger 5 | [84333](https://www.ncbi.nlm.nih.gov/gene/?term=84333) | -1.9 | 0.00064 | 16740.9 | 34141.4 |
| NQO2 | NAD(P)H quinone dehydrogenase 2 | [4835](https://www.ncbi.nlm.nih.gov/gene/?term=4835) | 1.9 | 0.00086 | 1317.2 | 642.7 |
| TMEM51 | transmembrane protein 51 | [55092](https://www.ncbi.nlm.nih.gov/gene/?term=55092) | 1.9 | 0.02268 | 19.6 | 7.9 |
| PLBD2 | phospholipase B domain containing 2 | [196463](https://www.ncbi.nlm.nih.gov/gene/?term=196463) | -1.9 | 0.00436 | 226.8 | 482.5 |
| DUSP1 | dual specificity phosphatase 1 | [1843](https://www.ncbi.nlm.nih.gov/gene/?term=1843) | 1.9 | 0.00091 | 6602.6 | 3229.8 |
| TRAJ33 | T cell receptor alpha joining 33 | [28722](https://www.ncbi.nlm.nih.gov/gene/?term=28722) | -1.9 | 0.01550 | 10.7 | 25.6 |
| LINC00528 | long intergenic non-protein coding RNA 528 | [200298](https://www.ncbi.nlm.nih.gov/gene/?term=200298) | 1.9 | 0.00003 | 159.0 | 81.0 |
| HEBP2 | heme binding protein 2 | [23593](https://www.ncbi.nlm.nih.gov/gene/?term=23593) | 1.9 | 0.00006 | 1380.6 | 694.4 |
| FBXL17 | F-box and leucine-rich repeat protein 17 | [64839](https://www.ncbi.nlm.nih.gov/gene/?term=64839) | -1.9 | 0.00002 | 348.0 | 688.0 |
| SIK3 | SIK family kinase 3 | [23387](https://www.ncbi.nlm.nih.gov/gene/?term=23387) | -1.9 | 0.00023 | 1464.1 | 2939.6 |
| FPR1 | formyl peptide receptor 1 | [2357](https://www.ncbi.nlm.nih.gov/gene/?term=2357) | 1.9 | 0.00220 | 21013.9 | 10202.3 |
| FURIN | furin, paired basic amino acid cleaving enzyme | [5045](https://www.ncbi.nlm.nih.gov/gene/?term=5045) | -1.9 | 0.00514 | 3069.9 | 6619.9 |
| LOC101927923 |  | [101927923](https://www.ncbi.nlm.nih.gov/gene/?term=101927923) | -1.9 | 0.02237 | 26.2 | 64.8 |
| RHOU | ras homolog family member U | [58480](https://www.ncbi.nlm.nih.gov/gene/?term=58480) | 1.9 | 0.00041 | 896.6 | 445.2 |
| SLC4A1 | solute carrier family 4 member 1 (Diego blood group) | [6521](https://www.ncbi.nlm.nih.gov/gene/?term=6521) | -1.9 | 0.01772 | 52947.4 | 123077.2 |
| CDCA7 | cell division cycle associated 7 | [83879](https://www.ncbi.nlm.nih.gov/gene/?term=83879) | 1.9 | 0.00892 | 140.7 | 64.4 |
| LOC107986853 |  | [107986853](https://www.ncbi.nlm.nih.gov/gene/?term=107986853) | -1.9 | 0.00363 | 235.1 | 502.1 |
| AKAP11 | A-kinase anchoring protein 11 | [11215](https://www.ncbi.nlm.nih.gov/gene/?term=11215) | -1.9 | 0.00045 | 1209.0 | 2442.8 |
| NLRP3 | NLR family, pyrin domain containing 3 | [114548](https://www.ncbi.nlm.nih.gov/gene/?term=114548) | 1.9 | 0.00048 | 1536.0 | 759.8 |
| LOC101928573 |  | [101928573](https://www.ncbi.nlm.nih.gov/gene/?term=101928573) | 1.9 | 0.02615 | 7.3 | 3.0 |
| STXBP1 | syntaxin binding protein 1 | [6812](https://www.ncbi.nlm.nih.gov/gene/?term=6812) | -1.9 | 0.01220 | 60.9 | 135.6 |
| STAU1 | staufen double-stranded RNA binding protein 1 | [6780](https://www.ncbi.nlm.nih.gov/gene/?term=6780) | -1.9 | 0.00026 | 2493.2 | 5013.8 |
| RNF26 | ring finger protein 26 | [79102](https://www.ncbi.nlm.nih.gov/gene/?term=79102) | -1.9 | 0.00216 | 79.9 | 166.3 |
| TOMM40L | translocase of outer mitochondrial membrane 40 like | [84134](https://www.ncbi.nlm.nih.gov/gene/?term=84134) | 1.9 | 0.00000 | 296.6 | 153.1 |
| EHF | ETS homologous factor | [26298](https://www.ncbi.nlm.nih.gov/gene/?term=26298) | -1.9 | 0.01045 | 5.1 | 11.3 |
| SPTLC1P1 | serine palmitoyltransferase long chain base subunit 1 pseudogene 1 | [100874511](https://www.ncbi.nlm.nih.gov/gene/?term=100874511) | -1.9 | 0.01399 | 7.4 | 17.6 |
| LOC102724955 |  | [102724955](https://www.ncbi.nlm.nih.gov/gene/?term=102724955) | 1.9 | 0.00768 | 65.9 | 30.9 |
| LOC105377016 |  | [105377016](https://www.ncbi.nlm.nih.gov/gene/?term=105377016) | -1.9 | 0.00216 | 692.5 | 1436.9 |
| RGS7 | regulator of G-protein signaling 7 | [6000](https://www.ncbi.nlm.nih.gov/gene/?term=6000) | -1.9 | 0.02216 | 3.9 | 9.2 |
| CALML4 | calmodulin like 4 | [91860](https://www.ncbi.nlm.nih.gov/gene/?term=91860) | 1.9 | 0.00000 | 498.2 | 256.8 |
| IL5RA | interleukin 5 receptor subunit alpha | [3568](https://www.ncbi.nlm.nih.gov/gene/?term=3568) | -1.9 | 0.01694 | 214.7 | 505.0 |
| LINC01127 | long intergenic non-protein coding RNA 1127 | [100506328](https://www.ncbi.nlm.nih.gov/gene/?term=100506328) | 1.9 | 0.00712 | 172.3 | 80.0 |
| SIGLEC17P | sialic acid binding Ig like lectin 17, pseudogene | [284367](https://www.ncbi.nlm.nih.gov/gene/?term=284367) | -1.9 | 0.02728 | 2.7 | 7.6 |
| HNRNPLL | heterogeneous nuclear ribonucleoprotein L like | [92906](https://www.ncbi.nlm.nih.gov/gene/?term=92906) | 1.9 | 0.00000 | 1324.4 | 679.1 |
| MGST1 | microsomal glutathione S-transferase 1 | [4257](https://www.ncbi.nlm.nih.gov/gene/?term=4257) | 1.9 | 0.00188 | 246.1 | 119.1 |
| LOC105371087 |  | [105371087](https://www.ncbi.nlm.nih.gov/gene/?term=105371087) | -1.9 | 0.03806 | 7.0 | 22.2 |
| SH3BGRL3 | SH3 domain binding glutamate rich protein like 3 | [83442](https://www.ncbi.nlm.nih.gov/gene/?term=83442) | -1.9 | 0.00095 | 585.8 | 1192.2 |
| LOC107986318 |  | [107986318](https://www.ncbi.nlm.nih.gov/gene/?term=107986318) | -1.9 | 0.03272 | 4.0 | 11.2 |
| PDPR | pyruvate dehydrogenase phosphatase regulatory subunit | [55066](https://www.ncbi.nlm.nih.gov/gene/?term=55066) | -1.9 | 0.00285 | 263.7 | 551.2 |
| LOC101927613 |  | [101927613](https://www.ncbi.nlm.nih.gov/gene/?term=101927613) | -1.9 | 0.00693 | 13.4 | 29.7 |
| ACSL4 | acyl-CoA synthetase long-chain family member 4 | [2182](https://www.ncbi.nlm.nih.gov/gene/?term=2182) | 1.9 | 0.00021 | 7334.4 | 3680.8 |
| LINC00239 | long intergenic non-protein coding RNA 239 | [145200](https://www.ncbi.nlm.nih.gov/gene/?term=145200) | 1.9 | 0.00469 | 17.7 | 8.5 |
| CD248 | CD248 molecule | [57124](https://www.ncbi.nlm.nih.gov/gene/?term=57124) | -1.9 | 0.02695 | 8.2 | 20.9 |
| LOC107986432 |  | [107986432](https://www.ncbi.nlm.nih.gov/gene/?term=107986432) | -1.9 | 0.00437 | 111.8 | 237.8 |
| RAB34 | RAB34, member RAS oncogene family | [83871](https://www.ncbi.nlm.nih.gov/gene/?term=83871) | 1.9 | 0.00005 | 170.1 | 85.9 |
| SPATA6 | spermatogenesis associated 6 | [54558](https://www.ncbi.nlm.nih.gov/gene/?term=54558) | -1.9 | 0.00195 | 64.0 | 133.4 |
| ZNF513 | zinc finger protein 513 | [130557](https://www.ncbi.nlm.nih.gov/gene/?term=130557) | 1.9 | 0.00001 | 243.3 | 123.7 |
| HLF | HLF, PAR bZIP transcription factor | [3131](https://www.ncbi.nlm.nih.gov/gene/?term=3131) | -1.9 | 0.02216 | 4.6 | 11.6 |
| P2RX4 | purinergic receptor P2X 4 | [5025](https://www.ncbi.nlm.nih.gov/gene/?term=5025) | 1.9 | 0.00000 | 605.7 | 312.7 |
| LOC105372698 |  | [105372698](https://www.ncbi.nlm.nih.gov/gene/?term=105372698) | -1.9 | 0.00447 | 66.2 | 140.9 |
| HMGN2P3 | high mobility group nucleosomal binding domain 2 pseudogene 3 | [728632](https://www.ncbi.nlm.nih.gov/gene/?term=728632) | 1.9 | 0.00035 | 34.7 | 17.5 |
| CD3E | CD3e molecule | [916](https://www.ncbi.nlm.nih.gov/gene/?term=916) | -1.9 | 0.00870 | 135.8 | 296.6 |
| LOC105372754 |  | [105372754](https://www.ncbi.nlm.nih.gov/gene/?term=105372754) | -1.9 | 0.03080 | 6.3 | 17.1 |
| LOC105371716 |  | [105371716](https://www.ncbi.nlm.nih.gov/gene/?term=105371716) | 1.9 | 0.01468 | 14.5 | 6.7 |
| LOC105370092 |  | [105370092](https://www.ncbi.nlm.nih.gov/gene/?term=105370092) | -1.9 | 0.02420 | 6.5 | 15.8 |
| LOC107985228 |  | [107985228](https://www.ncbi.nlm.nih.gov/gene/?term=107985228) | -1.9 | 0.01789 | 12.1 | 29.1 |
| METTL16 | methyltransferase like 16 | [79066](https://www.ncbi.nlm.nih.gov/gene/?term=79066) | -1.9 | 0.00000 | 158.6 | 308.6 |
| TESPA1 | thymocyte expressed, positive selection associated 1 | [9840](https://www.ncbi.nlm.nih.gov/gene/?term=9840) | -1.9 | 0.00079 | 426.9 | 867.6 |
| LRPAP1 | LDL receptor related protein associated protein 1 | [4043](https://www.ncbi.nlm.nih.gov/gene/?term=4043) | 1.9 | 0.00000 | 3271.3 | 1693.2 |
| LOC107984312 |  | [107984312](https://www.ncbi.nlm.nih.gov/gene/?term=107984312) | -1.9 | 0.00214 | 121.9 | 249.8 |
| CPSF7 | cleavage and polyadenylation specific factor 7 | [79869](https://www.ncbi.nlm.nih.gov/gene/?term=79869) | -1.9 | 0.00194 | 186.6 | 387.2 |
| LOC101928020 | uncharacterized LOC101928020 | [101928020](https://www.ncbi.nlm.nih.gov/gene/?term=101928020) | -1.9 | 0.00011 | 69.3 | 136.5 |
| MAD2L2 | MAD2 mitotic arrest deficient-like 2 (yeast) | [10459](https://www.ncbi.nlm.nih.gov/gene/?term=10459) | 1.9 | 0.00000 | 476.0 | 247.8 |
| ZNF784 | zinc finger protein 784 | [147808](https://www.ncbi.nlm.nih.gov/gene/?term=147808) | 1.9 | 0.00075 | 83.4 | 41.2 |
| GAPDH | glyceraldehyde-3-phosphate dehydrogenase | [2597](https://www.ncbi.nlm.nih.gov/gene/?term=2597) | 1.9 | 0.00001 | 30345.8 | 15631.9 |
| GALNT3 | polypeptide N-acetylgalactosaminyltransferase 3 | [2591](https://www.ncbi.nlm.nih.gov/gene/?term=2591) | 1.9 | 0.00013 | 1555.5 | 791.0 |
| LOC102725121 |  | [102725121](https://www.ncbi.nlm.nih.gov/gene/?term=102725121) | -1.9 | 0.02741 | 2.7 | 6.4 |
| LOC105376090 |  | [105376090](https://www.ncbi.nlm.nih.gov/gene/?term=105376090) | -1.9 | 0.00891 | 112.2 | 245.1 |
| KIAA1191 | KIAA1191 | [57179](https://www.ncbi.nlm.nih.gov/gene/?term=57179) | -1.9 | 0.00183 | 1956.3 | 3995.5 |
| AGO2 | argonaute RISC catalytic component 2 | [27161](https://www.ncbi.nlm.nih.gov/gene/?term=27161) | -1.9 | 0.00127 | 4470.9 | 9063.4 |
| SFRP2 | secreted frizzled-related protein 2 | [6423](https://www.ncbi.nlm.nih.gov/gene/?term=6423) | -1.9 | 0.02715 | 253.7 | 636.8 |
| IER5 | immediate early response 5 | [51278](https://www.ncbi.nlm.nih.gov/gene/?term=51278) | 1.9 | 0.00002 | 1477.1 | 759.0 |
| SPEF2 | sperm flagellar 2 | [79925](https://www.ncbi.nlm.nih.gov/gene/?term=79925) | -1.9 | 0.00004 | 67.6 | 132.3 |
| LOC653503 | guanine nucleotide binding protein (G protein), gamma 10 pseudogene | [653503](https://www.ncbi.nlm.nih.gov/gene/?term=653503) | 1.9 | 0.02017 | 1011.5 | 435.3 |
| SLC3A2 | solute carrier family 3 member 2 | [6520](https://www.ncbi.nlm.nih.gov/gene/?term=6520) | 1.9 | 0.00000 | 1650.9 | 865.6 |
| KLHDC8A | kelch domain containing 8A | [55220](https://www.ncbi.nlm.nih.gov/gene/?term=55220) | -1.9 | 0.01240 | 37.7 | 82.0 |
| KCNK5 | potassium two pore domain channel subfamily K member 5 | [8645](https://www.ncbi.nlm.nih.gov/gene/?term=8645) | 1.9 | 0.01364 | 28.6 | 12.9 |
| CD200 | CD200 molecule | [4345](https://www.ncbi.nlm.nih.gov/gene/?term=4345) | -1.9 | 0.00053 | 167.5 | 334.1 |
| C20orf24 | chromosome 20 open reading frame 24 | [55969](https://www.ncbi.nlm.nih.gov/gene/?term=55969) | 1.9 | 0.00006 | 453.0 | 231.6 |
| CDC16 | cell division cycle 16 | [8881](https://www.ncbi.nlm.nih.gov/gene/?term=8881) | -1.9 | 0.00185 | 2279.0 | 4647.2 |
| TIMM10 | translocase of inner mitochondrial membrane 10 homolog (yeast) | [26519](https://www.ncbi.nlm.nih.gov/gene/?term=26519) | 1.9 | 0.00013 | 227.3 | 114.9 |
| CASP7 | caspase 7 | [840](https://www.ncbi.nlm.nih.gov/gene/?term=840) | 1.9 | 0.00000 | 867.8 | 451.3 |
| C18orf65 | chromosome 18 open reading frame 65 | [400658](https://www.ncbi.nlm.nih.gov/gene/?term=400658) | 1.9 | 0.02443 | 7.1 | 3.1 |
| ELOVL3 | ELOVL fatty acid elongase 3 | [83401](https://www.ncbi.nlm.nih.gov/gene/?term=83401) | 1.9 | 0.02389 | 9.6 | 3.9 |
| LOC107987108 |  | [107987108](https://www.ncbi.nlm.nih.gov/gene/?term=107987108) | -1.9 | 0.02931 | 4.8 | 12.2 |
| LOC102723324 |  | [102723324](https://www.ncbi.nlm.nih.gov/gene/?term=102723324) | -1.9 | 0.00428 | 174.2 | 363.0 |
| GSK3A | glycogen synthase kinase 3 alpha | [2931](https://www.ncbi.nlm.nih.gov/gene/?term=2931) | -1.9 | 0.00083 | 327.9 | 658.6 |
| VIM | vimentin | [7431](https://www.ncbi.nlm.nih.gov/gene/?term=7431) | 1.9 | 0.00008 | 33868.7 | 17320.4 |
| PPFIA3 | PTPRF interacting protein alpha 3 | [8541](https://www.ncbi.nlm.nih.gov/gene/?term=8541) | 1.9 | 0.00345 | 14.1 | 6.6 |
| GATA2 | GATA binding protein 2 | [2624](https://www.ncbi.nlm.nih.gov/gene/?term=2624) | -1.9 | 0.00291 | 63.7 | 131.5 |
| POR | cytochrome p450 oxidoreductase | [5447](https://www.ncbi.nlm.nih.gov/gene/?term=5447) | 1.9 | 0.00032 | 1120.6 | 567.4 |
| POC1A | POC1 centriolar protein A | [25886](https://www.ncbi.nlm.nih.gov/gene/?term=25886) | 1.9 | 0.00069 | 70.8 | 35.6 |
| RBM26 | RNA binding motif protein 26 | [64062](https://www.ncbi.nlm.nih.gov/gene/?term=64062) | -1.9 | 0.00001 | 1063.0 | 2052.2 |
| UBE2E3 | ubiquitin conjugating enzyme E2 E3 | [10477](https://www.ncbi.nlm.nih.gov/gene/?term=10477) | -1.9 | 0.00000 | 758.8 | 1458.7 |
| C19orf38 | chromosome 19 open reading frame 38 | [255809](https://www.ncbi.nlm.nih.gov/gene/?term=255809) | 1.9 | 0.00008 | 1119.4 | 573.0 |
| FBXL4 | F-box and leucine-rich repeat protein 4 | [26235](https://www.ncbi.nlm.nih.gov/gene/?term=26235) | -1.9 | 0.00198 | 4515.1 | 9180.3 |
| HN1 | hematological and neurological expressed 1 | [51155](https://www.ncbi.nlm.nih.gov/gene/?term=51155) | 1.9 | 0.00007 | 2855.0 | 1466.2 |
| FILIP1L | filamin A interacting protein 1-like | [11259](https://www.ncbi.nlm.nih.gov/gene/?term=11259) | -1.9 | 0.01030 | 10.3 | 22.4 |
| NAGK | N-acetylglucosamine kinase | [55577](https://www.ncbi.nlm.nih.gov/gene/?term=55577) | 1.9 | 0.00000 | 2452.7 | 1276.7 |
| NR3C2 | nuclear receptor subfamily 3 group C member 2 | [4306](https://www.ncbi.nlm.nih.gov/gene/?term=4306) | -1.9 | 0.00014 | 140.5 | 275.8 |
| SYVN1 | synoviolin 1 | [84447](https://www.ncbi.nlm.nih.gov/gene/?term=84447) | -1.9 | 0.00022 | 192.1 | 379.5 |
| ACAP1 | ArfGAP with coiled-coil, ankyrin repeat and PH domains 1 | [9744](https://www.ncbi.nlm.nih.gov/gene/?term=9744) | 1.9 | 0.00001 | 3119.5 | 1621.9 |
| LOC107985392 |  | [107985392](https://www.ncbi.nlm.nih.gov/gene/?term=107985392) | 1.9 | 0.00810 | 30.6 | 14.5 |
| CLEC4E | C-type lectin domain family 4 member E | [26253](https://www.ncbi.nlm.nih.gov/gene/?term=26253) | 1.9 | 0.00340 | 3226.0 | 1565.7 |
| CARD8-AS1 | CARD8 antisense RNA 1 | [100505812](https://www.ncbi.nlm.nih.gov/gene/?term=100505812) | 1.9 | 0.00001 | 1320.1 | 683.6 |
| COA6 | cytochrome c oxidase assembly factor 6 | [388753](https://www.ncbi.nlm.nih.gov/gene/?term=388753) | 1.9 | 0.00000 | 324.0 | 169.9 |
| NFIB | nuclear factor I B | [4781](https://www.ncbi.nlm.nih.gov/gene/?term=4781) | -1.9 | 0.01850 | 18.0 | 41.1 |
| RRBP1 | ribosome binding protein 1 | [6238](https://www.ncbi.nlm.nih.gov/gene/?term=6238) | 1.9 | 0.00002 | 1742.8 | 904.2 |
| MLLT4 | myeloid/lymphoid or mixed-lineage leukemia; translocated to, 4 | [4301](https://www.ncbi.nlm.nih.gov/gene/?term=4301) | -1.9 | 0.00271 | 113.8 | 234.1 |
| PRKAG2-AS1 | PRKAG2 antisense RNA 1 | [100505483](https://www.ncbi.nlm.nih.gov/gene/?term=100505483) | -1.9 | 0.01117 | 4.2 | 9.4 |
| BRMS1 | breast cancer metastasis suppressor 1 | [25855](https://www.ncbi.nlm.nih.gov/gene/?term=25855) | 1.9 | 0.00000 | 924.6 | 485.8 |
| DST | dystonin | [667](https://www.ncbi.nlm.nih.gov/gene/?term=667) | -1.9 | 0.00005 | 386.7 | 750.2 |
| USP9X | ubiquitin specific peptidase 9, X-linked | [8239](https://www.ncbi.nlm.nih.gov/gene/?term=8239) | -1.9 | 0.00018 | 9769.8 | 19172.2 |
| ZC3H12A | zinc finger CCCH-type containing 12A | [80149](https://www.ncbi.nlm.nih.gov/gene/?term=80149) | 1.9 | 0.00002 | 433.6 | 225.0 |
| LOC107986707 |  | [107986707](https://www.ncbi.nlm.nih.gov/gene/?term=107986707) | -1.9 | 0.02358 | 15.4 | 37.3 |
| SLC7A1 | solute carrier family 7 member 1 | [6541](https://www.ncbi.nlm.nih.gov/gene/?term=6541) | -1.9 | 0.00186 | 510.6 | 1035.2 |
| ADAM22 | ADAM metallopeptidase domain 22 | [53616](https://www.ncbi.nlm.nih.gov/gene/?term=53616) | -1.9 | 0.00067 | 40.2 | 80.7 |
| LOC105371632 |  | [105371632](https://www.ncbi.nlm.nih.gov/gene/?term=105371632) | -1.9 | 0.02612 | 4.4 | 11.6 |
| BANK1 | B-cell scaffold protein with ankyrin repeats 1 | [55024](https://www.ncbi.nlm.nih.gov/gene/?term=55024) | -1.9 | 0.00014 | 1073.9 | 2097.0 |
| CCDC24 | coiled-coil domain containing 24 | [149473](https://www.ncbi.nlm.nih.gov/gene/?term=149473) | 1.9 | 0.00503 | 41.0 | 20.1 |
| SNORD3B-1 | small nucleolar RNA, C/D box 3B-1 | [26851](https://www.ncbi.nlm.nih.gov/gene/?term=26851) | 1.9 | 0.02054 | 10.6 | 4.5 |
| HIST1H3G | histone cluster 1, H3g | [8355](https://www.ncbi.nlm.nih.gov/gene/?term=8355) | 1.9 | 0.03152 | 869.6 | 347.8 |
| CARMIL3 | capping protein regulator and myosin 1 linker 3 | [90668](https://www.ncbi.nlm.nih.gov/gene/?term=90668) | 1.9 | 0.02832 | 27.6 | 11.5 |
| LOC107986460 |  | [107986460](https://www.ncbi.nlm.nih.gov/gene/?term=107986460) | -1.9 | 0.02671 | 18.0 | 44.5 |
| DGCR8 | DGCR8 microprocessor complex subunit | [54487](https://www.ncbi.nlm.nih.gov/gene/?term=54487) | -1.8 | 0.00006 | 322.0 | 623.1 |
| FHDC1 | FH2 domain containing 1 | [85462](https://www.ncbi.nlm.nih.gov/gene/?term=85462) | -1.8 | 0.01060 | 352.3 | 761.9 |
| MOV10 | Mov10 RISC complex RNA helicase | [4343](https://www.ncbi.nlm.nih.gov/gene/?term=4343) | 1.8 | 0.00563 | 682.1 | 327.0 |
| CD151 | CD151 molecule (Raph blood group) | [977](https://www.ncbi.nlm.nih.gov/gene/?term=977) | 1.8 | 0.00036 | 923.0 | 470.5 |
| GCA | grancalcin | [25801](https://www.ncbi.nlm.nih.gov/gene/?term=25801) | 1.8 | 0.00308 | 16927.4 | 8292.5 |
| LOC100422414 | proteasome (prosome, macropain) 26S subunit, non-ATPase, 7 pseudogene | [100422414](https://www.ncbi.nlm.nih.gov/gene/?term=100422414) | -1.8 | 0.01720 | 4.0 | 9.6 |
| POF1B | premature ovarian failure, 1B | [79983](https://www.ncbi.nlm.nih.gov/gene/?term=79983) | -1.8 | 0.04188 | 1.3 | 3.7 |
| LYSMD2 | LysM domain containing 2 | [256586](https://www.ncbi.nlm.nih.gov/gene/?term=256586) | 1.8 | 0.00001 | 745.9 | 388.7 |
| LOC730069 | nuclear receptor binding factor 2 pseudogene | [730069](https://www.ncbi.nlm.nih.gov/gene/?term=730069) | -1.8 | 0.00746 | 11.7 | 25.6 |
| SULT1A1 | sulfotransferase family 1A member 1 | [6817](https://www.ncbi.nlm.nih.gov/gene/?term=6817) | 1.8 | 0.00698 | 1782.5 | 854.9 |
| ALDH3B1 | aldehyde dehydrogenase 3 family member B1 | [221](https://www.ncbi.nlm.nih.gov/gene/?term=221) | 1.8 | 0.00006 | 518.0 | 267.3 |
| UBE2O | ubiquitin conjugating enzyme E2 O | [63893](https://www.ncbi.nlm.nih.gov/gene/?term=63893) | -1.8 | 0.01577 | 5653.4 | 13060.6 |
| CTSZ | cathepsin Z | [1522](https://www.ncbi.nlm.nih.gov/gene/?term=1522) | 1.8 | 0.00001 | 5999.2 | 3145.6 |
| LOC107985289 |  | [107985289](https://www.ncbi.nlm.nih.gov/gene/?term=107985289) | -1.8 | 0.02113 | 10.3 | 24.1 |
| STX11 | syntaxin 11 | [8676](https://www.ncbi.nlm.nih.gov/gene/?term=8676) | 1.8 | 0.00100 | 4984.8 | 2509.6 |
| ZNF571-AS1 | ZNF571 antisense RNA 1 | [100507433](https://www.ncbi.nlm.nih.gov/gene/?term=100507433) | -1.8 | 0.01034 | 5.7 | 12.0 |
| LOC107984271 |  | [107984271](https://www.ncbi.nlm.nih.gov/gene/?term=107984271) | -1.8 | 0.00737 | 20.6 | 42.6 |
| ITGB8 | integrin subunit beta 8 | [3696](https://www.ncbi.nlm.nih.gov/gene/?term=3696) | -1.8 | 0.00658 | 50.0 | 104.9 |
| ARPC1B | actin related protein 2/3 complex subunit 1B | [10095](https://www.ncbi.nlm.nih.gov/gene/?term=10095) | 1.8 | 0.00000 | 14261.8 | 7589.4 |
| TRAJ9 | T cell receptor alpha joining 9 | [28746](https://www.ncbi.nlm.nih.gov/gene/?term=28746) | -1.8 | 0.02179 | 8.8 | 21.6 |
| ZNF721 | zinc finger protein 721 | [170960](https://www.ncbi.nlm.nih.gov/gene/?term=170960) | -1.8 | 0.00001 | 531.3 | 1016.6 |
| SNORD49A | small nucleolar RNA, C/D box 49A | [26800](https://www.ncbi.nlm.nih.gov/gene/?term=26800) | 1.8 | 0.00139 | 61.0 | 30.1 |
| PTPRS | protein tyrosine phosphatase, receptor type S | [5802](https://www.ncbi.nlm.nih.gov/gene/?term=5802) | -1.8 | 0.00617 | 58.7 | 123.4 |
| VASP | vasodilator-stimulated phosphoprotein | [7408](https://www.ncbi.nlm.nih.gov/gene/?term=7408) | 1.8 | 0.00004 | 10899.7 | 5679.0 |
| MDM4 | MDM4, p53 regulator | [4194](https://www.ncbi.nlm.nih.gov/gene/?term=4194) | -1.8 | 0.00002 | 2021.6 | 3873.6 |
| TTN | titin | [7273](https://www.ncbi.nlm.nih.gov/gene/?term=7273) | -1.8 | 0.00276 | 1742.4 | 3575.0 |
| NEXN | nexilin F-actin binding protein | [91624](https://www.ncbi.nlm.nih.gov/gene/?term=91624) | 1.8 | 0.00300 | 324.2 | 160.0 |
| LOC105377205 |  | [105377205](https://www.ncbi.nlm.nih.gov/gene/?term=105377205) | 1.8 | 0.01047 | 11.9 | 5.5 |
| LOC441455 | makorin ring finger protein 1 pseudogene | [441455](https://www.ncbi.nlm.nih.gov/gene/?term=441455) | -1.8 | 0.03759 | 10.8 | 29.9 |
| LOC101928813 |  | [101928813](https://www.ncbi.nlm.nih.gov/gene/?term=101928813) | 1.8 | 0.02065 | 32.7 | 14.2 |
| LOC100421109 | karyopherin subunit alpha 4 pseudogene | [100421109](https://www.ncbi.nlm.nih.gov/gene/?term=100421109) | -1.8 | 0.03173 | 5.4 | 14.5 |
| LINC00189 | long intergenic non-protein coding RNA 189 | [193629](https://www.ncbi.nlm.nih.gov/gene/?term=193629) | 1.8 | 0.02721 | 99.8 | 41.8 |
| C12orf76 | chromosome 12 open reading frame 76 | [400073](https://www.ncbi.nlm.nih.gov/gene/?term=400073) | 1.8 | 0.00151 | 615.9 | 307.7 |
| PRKD1 | protein kinase D1 | [5587](https://www.ncbi.nlm.nih.gov/gene/?term=5587) | -1.8 | 0.04210 | 3.7 | 12.1 |
| LOC105369436 |  | [105369436](https://www.ncbi.nlm.nih.gov/gene/?term=105369436) | -1.8 | 0.03515 | 4.9 | 12.1 |
| HOOK1 | hook microtubule-tethering protein 1 | [51361](https://www.ncbi.nlm.nih.gov/gene/?term=51361) | -1.8 | 0.00003 | 210.3 | 402.9 |
| FAM171A1 | family with sequence similarity 171 member A1 | [221061](https://www.ncbi.nlm.nih.gov/gene/?term=221061) | -1.8 | 0.01112 | 15.5 | 33.5 |
| LYRM1 | LYR motif containing 1 | [57149](https://www.ncbi.nlm.nih.gov/gene/?term=57149) | 1.8 | 0.00002 | 957.4 | 501.6 |
| BTF3P3 | basic transcription factor 3, pseudogene 3 | [652964](https://www.ncbi.nlm.nih.gov/gene/?term=652964) | -1.8 | 0.02728 | 14.8 | 36.3 |
| IFI27L1 | interferon alpha inducible protein 27 like 1 | [122509](https://www.ncbi.nlm.nih.gov/gene/?term=122509) | 1.8 | 0.02146 | 12.1 | 5.4 |
| LINC00987 | long intergenic non-protein coding RNA 987 | [100499405](https://www.ncbi.nlm.nih.gov/gene/?term=100499405) | -1.8 | 0.02258 | 11.9 | 28.4 |
| LOC727896 | cysteine and histidine rich domain containing 1 pseudogene | [727896](https://www.ncbi.nlm.nih.gov/gene/?term=727896) | -1.8 | 0.00245 | 120.2 | 242.0 |
| FBXO46 | F-box protein 46 | [23403](https://www.ncbi.nlm.nih.gov/gene/?term=23403) | -1.8 | 0.00034 | 49.8 | 98.0 |
| VPS37C | VPS37C, ESCRT-I subunit | [55048](https://www.ncbi.nlm.nih.gov/gene/?term=55048) | -1.8 | 0.00091 | 30.3 | 60.7 |
| MIR4432HG | MIR4432 host gene | [106660609](https://www.ncbi.nlm.nih.gov/gene/?term=106660609) | -1.8 | 0.01347 | 9.5 | 21.0 |
| SMPD3 | sphingomyelin phosphodiesterase 3 | [55512](https://www.ncbi.nlm.nih.gov/gene/?term=55512) | -1.8 | 0.02025 | 75.0 | 172.0 |
| LOC107987161 |  | [107987161](https://www.ncbi.nlm.nih.gov/gene/?term=107987161) | 1.8 | 0.00047 | 49.2 | 24.8 |
| KCNE3 | potassium voltage-gated channel subfamily E regulatory subunit 3 | [10008](https://www.ncbi.nlm.nih.gov/gene/?term=10008) | 1.8 | 0.00008 | 4766.7 | 2503.9 |
| CCR10 | C-C motif chemokine receptor 10 | [2826](https://www.ncbi.nlm.nih.gov/gene/?term=2826) | 1.8 | 0.02815 | 13.9 | 5.9 |
| LOC101926907 |  | [101926907](https://www.ncbi.nlm.nih.gov/gene/?term=101926907) | -1.8 | 0.01343 | 61.9 | 135.3 |
| FAM117B | family with sequence similarity 117 member B | [150864](https://www.ncbi.nlm.nih.gov/gene/?term=150864) | -1.8 | 0.00019 | 617.0 | 1193.4 |
| FKBP9 | FK506 binding protein 9 | [11328](https://www.ncbi.nlm.nih.gov/gene/?term=11328) | 1.8 | 0.00126 | 92.6 | 46.9 |
| LOC107984356 |  | [107984356](https://www.ncbi.nlm.nih.gov/gene/?term=107984356) | -1.8 | 0.02083 | 3.0 | 7.7 |
| GINS2 | GINS complex subunit 2 | [51659](https://www.ncbi.nlm.nih.gov/gene/?term=51659) | 1.8 | 0.02382 | 91.3 | 39.8 |
| RENBP | renin binding protein | [5973](https://www.ncbi.nlm.nih.gov/gene/?term=5973) | 1.8 | 0.00001 | 249.7 | 131.9 |
| AIM1L | absent in melanoma 1-like | [55057](https://www.ncbi.nlm.nih.gov/gene/?term=55057) | 1.8 | 0.00674 | 18.2 | 8.6 |
| TCIRG1 | T-cell immune regulator 1, ATPase H+ transporting V0 subunit a3 | [10312](https://www.ncbi.nlm.nih.gov/gene/?term=10312) | 1.8 | 0.00003 | 5317.9 | 2804.2 |
| LOC102724965 |  | [102724965](https://www.ncbi.nlm.nih.gov/gene/?term=102724965) | 1.8 | 0.00039 | 75.7 | 38.4 |
| TRIM25 | tripartite motif containing 25 | [7706](https://www.ncbi.nlm.nih.gov/gene/?term=7706) | 1.8 | 0.00250 | 5033.2 | 2512.6 |
| SLC15A3 | solute carrier family 15 member 3 | [51296](https://www.ncbi.nlm.nih.gov/gene/?term=51296) | 1.8 | 0.00001 | 1070.5 | 564.9 |
| HIST1H2AG | histone cluster 1, H2ag | [8969](https://www.ncbi.nlm.nih.gov/gene/?term=8969) | 1.8 | 0.00335 | 1467.0 | 728.5 |
| NAPA | NSF attachment protein alpha | [8775](https://www.ncbi.nlm.nih.gov/gene/?term=8775) | -1.8 | 0.00189 | 1604.7 | 3196.6 |
| HIST1H2BO | histone cluster 1, H2bo | [8348](https://www.ncbi.nlm.nih.gov/gene/?term=8348) | 1.8 | 0.00386 | 673.2 | 332.4 |
| FAXDC2 | fatty acid hydroxylase domain containing 2 | [10826](https://www.ncbi.nlm.nih.gov/gene/?term=10826) | -1.8 | 0.01685 | 7790.4 | 18483.6 |
| RNF187 | ring finger protein 187 | [149603](https://www.ncbi.nlm.nih.gov/gene/?term=149603) | -1.8 | 0.00535 | 720.5 | 1475.7 |
| LOC107986765 |  | [107986765](https://www.ncbi.nlm.nih.gov/gene/?term=107986765) | -1.8 | 0.03416 | 4.0 | 10.7 |
| SNORA22 | small nucleolar RNA, H/ACA box 22 | [677807](https://www.ncbi.nlm.nih.gov/gene/?term=677807) | 1.8 | 0.01148 | 16.0 | 7.3 |
| LOC107986143 |  | [107986143](https://www.ncbi.nlm.nih.gov/gene/?term=107986143) | -1.8 | 0.00169 | 27.5 | 55.9 |
| JMJD6 | arginine demethylase and lysine hydroxylase | [23210](https://www.ncbi.nlm.nih.gov/gene/?term=23210) | 1.8 | 0.00000 | 885.5 | 472.4 |
| PPP1R3D | protein phosphatase 1 regulatory subunit 3D | [5509](https://www.ncbi.nlm.nih.gov/gene/?term=5509) | 1.8 | 0.00040 | 1115.1 | 576.3 |
| LOC101927898 |  | [101927898](https://www.ncbi.nlm.nih.gov/gene/?term=101927898) | 1.8 | 0.00224 | 66.9 | 33.9 |
| HIST2H2AB | histone cluster 2, H2ab | [317772](https://www.ncbi.nlm.nih.gov/gene/?term=317772) | 1.8 | 0.00092 | 612.8 | 312.4 |
| FGR | FGR proto-oncogene, Src family tyrosine kinase | [2268](https://www.ncbi.nlm.nih.gov/gene/?term=2268) | 1.8 | 0.00002 | 10867.2 | 5719.6 |
| AQP9 | aquaporin 9 | [366](https://www.ncbi.nlm.nih.gov/gene/?term=366) | 1.8 | 0.00398 | 19166.9 | 9546.5 |
| LRRC75A | leucine rich repeat containing 75A | [388341](https://www.ncbi.nlm.nih.gov/gene/?term=388341) | -1.8 | 0.00932 | 373.8 | 786.0 |
| C12orf42 | chromosome 12 open reading frame 42 | [374470](https://www.ncbi.nlm.nih.gov/gene/?term=374470) | -1.8 | 0.00006 | 56.3 | 107.7 |
| YWHABP2 | tyrosine 3-monooxygenase/tryptophan 5-monooxygenase activation protein beta pseudogene 2 | [100422666](https://www.ncbi.nlm.nih.gov/gene/?term=100422666) | -1.8 | 0.02088 | 10.6 | 24.7 |
| GLUD1P3 | glutamate dehydrogenase 1 pseudogene 3 | [2749](https://www.ncbi.nlm.nih.gov/gene/?term=2749) | -1.8 | 0.02509 | 4.7 | 11.2 |
| RNF31 | ring finger protein 31 | [55072](https://www.ncbi.nlm.nih.gov/gene/?term=55072) | 1.8 | 0.00000 | 1249.9 | 669.0 |
| NPM1P37 | nucleophosmin 1 (nucleolar phosphoprotein B23, numatrin) pseudogene 37 | [100422261](https://www.ncbi.nlm.nih.gov/gene/?term=100422261) | -1.8 | 0.00129 | 30.9 | 62.3 |
| SPHK1 | sphingosine kinase 1 | [8877](https://www.ncbi.nlm.nih.gov/gene/?term=8877) | 1.8 | 0.00367 | 199.4 | 98.6 |
| EML2 | echinoderm microtubule associated protein like 2 | [24139](https://www.ncbi.nlm.nih.gov/gene/?term=24139) | 1.8 | 0.00000 | 450.1 | 242.5 |
| MIR197 | microRNA 197 | [406974](https://www.ncbi.nlm.nih.gov/gene/?term=406974) | -1.8 | 0.01262 | 10.1 | 23.4 |
| LOC101928096 |  | [101928096](https://www.ncbi.nlm.nih.gov/gene/?term=101928096) | -1.8 | 0.00696 | 37.0 | 77.6 |
| LOC105369890 |  | [105369890](https://www.ncbi.nlm.nih.gov/gene/?term=105369890) | -1.8 | 0.00762 | 12.2 | 25.0 |
| IFNGR2 | interferon gamma receptor 2 (interferon gamma transducer 1) | [3460](https://www.ncbi.nlm.nih.gov/gene/?term=3460) | 1.8 | 0.00037 | 558.4 | 289.4 |
| SKP1P1 | S-phase kinase-associated protein 1 pseudogene 1 | [6922](https://www.ncbi.nlm.nih.gov/gene/?term=6922) | -1.8 | 0.00048 | 41.5 | 78.6 |
| PNPT1 | polyribonucleotide nucleotidyltransferase 1 | [87178](https://www.ncbi.nlm.nih.gov/gene/?term=87178) | 1.8 | 0.00061 | 1532.1 | 787.7 |
| SEZ6L | seizure related 6 homolog like | [23544](https://www.ncbi.nlm.nih.gov/gene/?term=23544) | -1.8 | 0.01885 | 8.2 | 18.4 |
| OTUD4 | OTU deubiquitinase 4 | [54726](https://www.ncbi.nlm.nih.gov/gene/?term=54726) | -1.8 | 0.00000 | 1379.6 | 2585.4 |
| LOC107985424 |  | [107985424](https://www.ncbi.nlm.nih.gov/gene/?term=107985424) | 1.8 | 0.03157 | 31.4 | 13.3 |
| AFF2 | AF4/FMR2 family member 2 | [2334](https://www.ncbi.nlm.nih.gov/gene/?term=2334) | 1.8 | 0.00314 | 1965.2 | 989.4 |
| LOC102723750 |  | [102723750](https://www.ncbi.nlm.nih.gov/gene/?term=102723750) | -1.8 | 0.02151 | 1232.7 | 2809.1 |
| LOC105379392 |  | [105379392](https://www.ncbi.nlm.nih.gov/gene/?term=105379392) | -1.8 | 0.00724 | 1345.2 | 2769.1 |
| PBK | PDZ binding kinase | [55872](https://www.ncbi.nlm.nih.gov/gene/?term=55872) | 1.8 | 0.02622 | 51.8 | 22.4 |
| SYT1 | synaptotagmin 1 | [6857](https://www.ncbi.nlm.nih.gov/gene/?term=6857) | -1.8 | 0.03734 | 3.3 | 8.6 |
| FAM214B | family with sequence similarity 214 member B | [80256](https://www.ncbi.nlm.nih.gov/gene/?term=80256) | -1.8 | 0.01486 | 2543.9 | 5561.4 |
| CDH23 | cadherin-related 23 | [64072](https://www.ncbi.nlm.nih.gov/gene/?term=64072) | 1.8 | 0.00080 | 512.5 | 263.9 |
| TRAJ22 | T cell receptor alpha joining 22 | [28733](https://www.ncbi.nlm.nih.gov/gene/?term=28733) | -1.8 | 0.02262 | 18.9 | 43.7 |
| BORCS8 | BLOC-1 related complex subunit 8 | [729991](https://www.ncbi.nlm.nih.gov/gene/?term=729991) | 1.8 | 0.00001 | 109.5 | 58.5 |
| MAD2L1BP | MAD2L1 binding protein | [9587](https://www.ncbi.nlm.nih.gov/gene/?term=9587) | 1.8 | 0.00000 | 815.8 | 438.3 |
| CLEC2B | C-type lectin domain family 2 member B | [9976](https://www.ncbi.nlm.nih.gov/gene/?term=9976) | 1.8 | 0.00073 | 4698.9 | 2428.2 |
| SCGB1C1 | secretoglobin family 1C member 1 | [147199](https://www.ncbi.nlm.nih.gov/gene/?term=147199) | 1.8 | 0.04151 | 21.9 | 7.6 |
| ROPN1L | rhophilin associated tail protein 1 like | [83853](https://www.ncbi.nlm.nih.gov/gene/?term=83853) | 1.8 | 0.00611 | 341.7 | 168.1 |
| HDGFRP2 | hepatoma-derived growth factor-related protein 2 | [84717](https://www.ncbi.nlm.nih.gov/gene/?term=84717) | 1.8 | 0.00001 | 596.7 | 317.0 |
| LOC105369313 |  | [105369313](https://www.ncbi.nlm.nih.gov/gene/?term=105369313) | -1.8 | 0.00929 | 25.3 | 54.2 |
| UBE2S | ubiquitin conjugating enzyme E2 S | [27338](https://www.ncbi.nlm.nih.gov/gene/?term=27338) | 1.8 | 0.00004 | 486.0 | 256.1 |
| CEP112 | centrosomal protein 112 | [201134](https://www.ncbi.nlm.nih.gov/gene/?term=201134) | -1.8 | 0.00225 | 13.3 | 27.0 |
| LOC644924 | glutamic-oxaloacetic transaminase 2, mitochondrial (aspartate aminotransferase 2) pseudogene | [644924](https://www.ncbi.nlm.nih.gov/gene/?term=644924) | -1.8 | 0.03805 | 3.2 | 8.5 |
| HMGB2 | high mobility group box 2 | [3148](https://www.ncbi.nlm.nih.gov/gene/?term=3148) | 1.8 | 0.00019 | 4847.2 | 2535.3 |
| SATB1 | SATB homeobox 1 | [6304](https://www.ncbi.nlm.nih.gov/gene/?term=6304) | -1.8 | 0.00017 | 1414.6 | 2706.7 |
| KDM7A | lysine demethylase 7A | [80853](https://www.ncbi.nlm.nih.gov/gene/?term=80853) | -1.8 | 0.00231 | 14348.4 | 28503.0 |
| HSD3B7 | hydroxy-delta-5-steroid dehydrogenase, 3 beta- and steroid delta-isomerase 7 | [80270](https://www.ncbi.nlm.nih.gov/gene/?term=80270) | 1.8 | 0.00289 | 50.1 | 25.7 |
| LOC101929707 |  | [101929707](https://www.ncbi.nlm.nih.gov/gene/?term=101929707) | 1.8 | 0.01599 | 159.1 | 74.0 |
| FSTL3 | follistatin like 3 | [10272](https://www.ncbi.nlm.nih.gov/gene/?term=10272) | 1.8 | 0.00134 | 43.8 | 22.6 |
| RASGEF1A | RasGEF domain family member 1A | [221002](https://www.ncbi.nlm.nih.gov/gene/?term=221002) | 1.8 | 0.00032 | 149.5 | 78.3 |
| GALE | UDP-galactose-4-epimerase | [2582](https://www.ncbi.nlm.nih.gov/gene/?term=2582) | 1.8 | 0.00004 | 163.6 | 86.6 |
| LOC105375785 |  | [105375785](https://www.ncbi.nlm.nih.gov/gene/?term=105375785) | -1.8 | 0.01152 | 40.5 | 86.2 |
| LOC105373110 |  | [105373110](https://www.ncbi.nlm.nih.gov/gene/?term=105373110) | 1.8 | 0.00176 | 21.1 | 10.5 |
| LOC101927974 |  | [101927974](https://www.ncbi.nlm.nih.gov/gene/?term=101927974) | 1.8 | 0.00096 | 109.6 | 56.5 |
| EP400 | E1A binding protein p400 | [57634](https://www.ncbi.nlm.nih.gov/gene/?term=57634) | -1.8 | 0.00803 | 293.7 | 607.2 |
| LOC105379045 |  | [105379045](https://www.ncbi.nlm.nih.gov/gene/?term=105379045) | -1.8 | 0.00158 | 144.9 | 285.1 |
| SNORD56B | small nucleolar RNA, C/D box 56B | [319139](https://www.ncbi.nlm.nih.gov/gene/?term=319139) | -1.8 | 0.04304 | 3.3 | 10.8 |
| PTCH1 | patched 1 | [5727](https://www.ncbi.nlm.nih.gov/gene/?term=5727) | -1.8 | 0.01547 | 37.6 | 81.9 |
| CYP19A1 | cytochrome P450 family 19 subfamily A member 1 | [1588](https://www.ncbi.nlm.nih.gov/gene/?term=1588) | 1.8 | 0.04367 | 19.5 | 5.9 |
| FAM214A | family with sequence similarity 214 member A | [56204](https://www.ncbi.nlm.nih.gov/gene/?term=56204) | -1.8 | 0.00000 | 1281.1 | 2364.0 |
| UHMK1 | U2AF homology motif (UHM) kinase 1 | [127933](https://www.ncbi.nlm.nih.gov/gene/?term=127933) | -1.8 | 0.00036 | 9921.6 | 18927.1 |
| TYROBP | TYRO protein tyrosine kinase binding protein | [7305](https://www.ncbi.nlm.nih.gov/gene/?term=7305) | 1.8 | 0.00008 | 4020.5 | 2136.8 |
| NFKBIZ | NFKB inhibitor zeta | [64332](https://www.ncbi.nlm.nih.gov/gene/?term=64332) | 1.8 | 0.00002 | 3378.1 | 1798.6 |
| CLDN23 | claudin 23 | [137075](https://www.ncbi.nlm.nih.gov/gene/?term=137075) | 1.8 | 0.00595 | 32.4 | 16.1 |
| DAAM2 | dishevelled associated activator of morphogenesis 2 | [23500](https://www.ncbi.nlm.nih.gov/gene/?term=23500) | -1.8 | 0.03988 | 17.0 | 53.8 |
| HOOK2 | hook microtubule-tethering protein 2 | [29911](https://www.ncbi.nlm.nih.gov/gene/?term=29911) | 1.8 | 0.00011 | 99.4 | 52.4 |
| CSNK1G2 | casein kinase 1 gamma 2 | [1455](https://www.ncbi.nlm.nih.gov/gene/?term=1455) | 1.8 | 0.00000 | 2086.1 | 1121.8 |
| CEP68 | centrosomal protein 68 | [23177](https://www.ncbi.nlm.nih.gov/gene/?term=23177) | -1.8 | 0.00005 | 199.1 | 376.2 |
| LOC101928044 |  | [101928044](https://www.ncbi.nlm.nih.gov/gene/?term=101928044) | -1.8 | 0.01624 | 22.4 | 49.4 |
| MIR576 | microRNA 576 | [693161](https://www.ncbi.nlm.nih.gov/gene/?term=693161) | -1.8 | 0.04383 | 2.7 | 8.4 |
| PATJ | PATJ, crumbs cell polarity complex component | [10207](https://www.ncbi.nlm.nih.gov/gene/?term=10207) | -1.8 | 0.00002 | 393.8 | 738.3 |
| IMMP2L | IMP2 inner mitochondrial membrane peptidase-like (S. cerevisiae) | [83943](https://www.ncbi.nlm.nih.gov/gene/?term=83943) | -1.8 | 0.00001 | 154.5 | 287.7 |
| PTGS2 | prostaglandin-endoperoxide synthase 2 | [5743](https://www.ncbi.nlm.nih.gov/gene/?term=5743) | -1.8 | 0.00045 | 420.1 | 807.5 |
| SLC15A4 | solute carrier family 15 member 4 | [121260](https://www.ncbi.nlm.nih.gov/gene/?term=121260) | 1.8 | 0.00004 | 3412.6 | 1820.4 |
| LOC105379461 |  | [105379461](https://www.ncbi.nlm.nih.gov/gene/?term=105379461) | -1.8 | 0.02254 | 10.1 | 21.8 |
| COL19A1 | collagen type XIX alpha 1 | [1310](https://www.ncbi.nlm.nih.gov/gene/?term=1310) | -1.8 | 0.00316 | 415.8 | 823.3 |
| BRI3 | brain protein I3 | [25798](https://www.ncbi.nlm.nih.gov/gene/?term=25798) | 1.8 | 0.00006 | 1112.8 | 591.7 |
| NUDT14 | nudix hydrolase 14 | [256281](https://www.ncbi.nlm.nih.gov/gene/?term=256281) | 1.8 | 0.00021 | 80.3 | 41.2 |
| NT5DC1 | 5'-nucleotidase domain containing 1 | [221294](https://www.ncbi.nlm.nih.gov/gene/?term=221294) | -1.8 | 0.00003 | 175.6 | 331.1 |
| KPNA6 | karyopherin subunit alpha 6 | [23633](https://www.ncbi.nlm.nih.gov/gene/?term=23633) | -1.8 | 0.00151 | 3290.6 | 6399.7 |
| FMN1 | formin 1 | [342184](https://www.ncbi.nlm.nih.gov/gene/?term=342184) | -1.8 | 0.01299 | 47.5 | 100.3 |
| CDK1 | cyclin-dependent kinase 1 | [983](https://www.ncbi.nlm.nih.gov/gene/?term=983) | 1.8 | 0.01106 | 182.9 | 88.1 |
| CDK5RAP2 | CDK5 regulatory subunit associated protein 2 | [55755](https://www.ncbi.nlm.nih.gov/gene/?term=55755) | 1.8 | 0.00016 | 3490.4 | 1844.9 |
| SUPT3H | SPT3 homolog, SAGA and STAGA complex component | [8464](https://www.ncbi.nlm.nih.gov/gene/?term=8464) | -1.8 | 0.00122 | 130.1 | 253.8 |
| LOC105369370 |  | [105369370](https://www.ncbi.nlm.nih.gov/gene/?term=105369370) | -1.8 | 0.02393 | 6.1 | 13.7 |
| SMYD5 | SMYD family member 5 | [10322](https://www.ncbi.nlm.nih.gov/gene/?term=10322) | -1.8 | 0.01209 | 28.4 | 60.8 |
| TLR8 | toll like receptor 8 | [51311](https://www.ncbi.nlm.nih.gov/gene/?term=51311) | 1.8 | 0.00072 | 10562.4 | 5537.4 |
| PRPF31 | pre-mRNA processing factor 31 | [26121](https://www.ncbi.nlm.nih.gov/gene/?term=26121) | 1.8 | 0.03929 | 5.5 | 2.1 |
| SMAD1 | SMAD family member 1 | [4086](https://www.ncbi.nlm.nih.gov/gene/?term=4086) | 1.8 | 0.00012 | 132.0 | 69.6 |
| STX16 | syntaxin 16 | [8675](https://www.ncbi.nlm.nih.gov/gene/?term=8675) | -1.8 | 0.00056 | 694.7 | 1331.4 |
| KLRC4 | killer cell lectin like receptor C4 | [8302](https://www.ncbi.nlm.nih.gov/gene/?term=8302) | -1.8 | 0.01357 | 33.4 | 71.7 |
| TRAJ14 | T cell receptor alpha joining 14 | [28741](https://www.ncbi.nlm.nih.gov/gene/?term=28741) | -1.8 | 0.01913 | 14.2 | 32.4 |
| UGCG | UDP-glucose ceramide glucosyltransferase | [7357](https://www.ncbi.nlm.nih.gov/gene/?term=7357) | 1.8 | 0.00341 | 2645.9 | 1340.3 |
| PTPN13 | protein tyrosine phosphatase, non-receptor type 13 | [5783](https://www.ncbi.nlm.nih.gov/gene/?term=5783) | -1.8 | 0.00247 | 92.8 | 182.3 |
| CILP | cartilage intermediate layer protein | [8483](https://www.ncbi.nlm.nih.gov/gene/?term=8483) | -1.8 | 0.01904 | 9.8 | 20.6 |
| RFX3 | regulatory factor X3 | [5991](https://www.ncbi.nlm.nih.gov/gene/?term=5991) | -1.8 | 0.00001 | 895.7 | 1667.2 |
| PBX2 | pre-B-cell leukemia homeobox 2 | [5089](https://www.ncbi.nlm.nih.gov/gene/?term=5089) | 1.8 | 0.01201 | 25.4 | 12.2 |
| TLR4 | toll like receptor 4 | [7099](https://www.ncbi.nlm.nih.gov/gene/?term=7099) | 1.8 | 0.00170 | 16550.7 | 8609.8 |
| FAM222B | family with sequence similarity 222 member B | [55731](https://www.ncbi.nlm.nih.gov/gene/?term=55731) | -1.8 | 0.00499 | 50.5 | 101.8 |
| HSPA4L | heat shock protein family A (Hsp70) member 4 like | [22824](https://www.ncbi.nlm.nih.gov/gene/?term=22824) | 1.8 | 0.03801 | 31.7 | 12.6 |
| GPR135 | G protein-coupled receptor 135 | [64582](https://www.ncbi.nlm.nih.gov/gene/?term=64582) | -1.8 | 0.01624 | 30.3 | 65.6 |
| VAMP5 | vesicle associated membrane protein 5 | [10791](https://www.ncbi.nlm.nih.gov/gene/?term=10791) | 1.8 | 0.00046 | 323.2 | 169.5 |
| PMP22 | peripheral myelin protein 22 | [5376](https://www.ncbi.nlm.nih.gov/gene/?term=5376) | -1.8 | 0.01554 | 13.6 | 27.1 |
| LOC105370460 |  | [105370460](https://www.ncbi.nlm.nih.gov/gene/?term=105370460) | -1.8 | 0.02664 | 9.9 | 22.6 |
| HECTD3 | HECT domain E3 ubiquitin protein ligase 3 | [79654](https://www.ncbi.nlm.nih.gov/gene/?term=79654) | -1.8 | 0.00083 | 523.3 | 999.9 |
| FAM117A | family with sequence similarity 117 member A | [81558](https://www.ncbi.nlm.nih.gov/gene/?term=81558) | -1.8 | 0.00761 | 4179.2 | 8487.8 |
| LINC00968 | long intergenic non-protein coding RNA 968 | [100507632](https://www.ncbi.nlm.nih.gov/gene/?term=100507632) | 1.8 | 0.00601 | 71.6 | 35.2 |
| LOC105378954 |  | [105378954](https://www.ncbi.nlm.nih.gov/gene/?term=105378954) | 1.8 | 0.00384 | 35.0 | 17.4 |
| PHTF1 | putative homeodomain transcription factor 1 | [10745](https://www.ncbi.nlm.nih.gov/gene/?term=10745) | 1.8 | 0.00017 | 1053.8 | 558.8 |
| MKL2 | MKL1/myocardin like 2 | [57496](https://www.ncbi.nlm.nih.gov/gene/?term=57496) | -1.8 | 0.00083 | 197.5 | 380.3 |
| FRAT1 | frequently rearranged in advanced T-cell lymphomas 1 | [10023](https://www.ncbi.nlm.nih.gov/gene/?term=10023) | 1.8 | 0.00023 | 1810.3 | 958.2 |
| NEO1 | neogenin 1 | [4756](https://www.ncbi.nlm.nih.gov/gene/?term=4756) | -1.8 | 0.00072 | 51.6 | 99.4 |
| TRAFD1 | TRAF-type zinc finger domain containing 1 | [10906](https://www.ncbi.nlm.nih.gov/gene/?term=10906) | 1.8 | 0.00088 | 2369.5 | 1243.4 |
| HFE | hemochromatosis | [3077](https://www.ncbi.nlm.nih.gov/gene/?term=3077) | 1.8 | 0.00106 | 152.8 | 79.2 |
| PSRC1 | proline and serine rich coiled-coil 1 | [84722](https://www.ncbi.nlm.nih.gov/gene/?term=84722) | 1.8 | 0.00008 | 110.4 | 58.9 |
| PAK2 | p21 (RAC1) activated kinase 2 | [5062](https://www.ncbi.nlm.nih.gov/gene/?term=5062) | -1.8 | 0.00118 | 2638.6 | 5079.9 |
| SNORA12 | small nucleolar RNA, H/ACA box 12 | [677800](https://www.ncbi.nlm.nih.gov/gene/?term=677800) | 1.8 | 0.01127 | 416.8 | 202.3 |
| NPC2 | NPC intracellular cholesterol transporter 2 | [10577](https://www.ncbi.nlm.nih.gov/gene/?term=10577) | 1.8 | 0.00016 | 1791.9 | 954.2 |
| FBXW8 | F-box and WD repeat domain containing 8 | [26259](https://www.ncbi.nlm.nih.gov/gene/?term=26259) | -1.8 | 0.00109 | 86.8 | 167.8 |
| LOC158781 | tyrosine 3-monooxygenase/tryptophan 5-monooxygenase activation protein zeta pseudogene | [158781](https://www.ncbi.nlm.nih.gov/gene/?term=158781) | -1.8 | 0.01262 | 10.4 | 22.4 |
| KRT8P50 | keratin 8 pseudogene 50 | [102724430](https://www.ncbi.nlm.nih.gov/gene/?term=102724430) | -1.8 | 0.01675 | 7.0 | 14.7 |
| GALNT5 | polypeptide N-acetylgalactosaminyltransferase 5 | [11227](https://www.ncbi.nlm.nih.gov/gene/?term=11227) | -1.8 | 0.02362 | 600.3 | 1327.9 |
| CCDC124 | coiled-coil domain containing 124 | [115098](https://www.ncbi.nlm.nih.gov/gene/?term=115098) | -1.8 | 0.00152 | 111.6 | 213.8 |
| ADAMDEC1 | ADAM-like, decysin 1 | [27299](https://www.ncbi.nlm.nih.gov/gene/?term=27299) | 1.8 | 0.01356 | 17.6 | 8.5 |
| ITIH4 | inter-alpha-trypsin inhibitor heavy chain family member 4 | [3700](https://www.ncbi.nlm.nih.gov/gene/?term=3700) | 1.8 | 0.00481 | 147.0 | 74.4 |
| SRRM1 | serine and arginine repetitive matrix 1 | [10250](https://www.ncbi.nlm.nih.gov/gene/?term=10250) | -1.8 | 0.00009 | 596.5 | 1118.0 |
| SNORA11B | small nucleolar RNA, H/ACA box 11B | [100124539](https://www.ncbi.nlm.nih.gov/gene/?term=100124539) | -1.8 | 0.03213 | 4.3 | 10.9 |
| LINC01311 | long intergenic non-protein coding RNA 1311 | [100652736](https://www.ncbi.nlm.nih.gov/gene/?term=100652736) | 1.8 | 0.00485 | 19.0 | 9.6 |
| TNFRSF10C | tumor necrosis factor receptor superfamily member 10c | [8794](https://www.ncbi.nlm.nih.gov/gene/?term=8794) | 1.8 | 0.00180 | 7998.6 | 4138.4 |
| SH2D1B | SH2 domain containing 1B | [117157](https://www.ncbi.nlm.nih.gov/gene/?term=117157) | -1.8 | 0.00972 | 82.9 | 170.8 |
| ISG20 | interferon stimulated exonuclease gene 20kDa | [3669](https://www.ncbi.nlm.nih.gov/gene/?term=3669) | 1.8 | 0.00022 | 4073.5 | 2167.1 |
| PSKH1 | protein serine kinase H1 | [5681](https://www.ncbi.nlm.nih.gov/gene/?term=5681) | -1.8 | 0.00018 | 158.0 | 296.4 |
| LOC101928075 | uncharacterized LOC101928075 | [101928075](https://www.ncbi.nlm.nih.gov/gene/?term=101928075) | -1.8 | 0.03025 | 5.1 | 11.9 |
| SCAMP1-AS1 | SCAMP1 antisense RNA 1 | [728769](https://www.ncbi.nlm.nih.gov/gene/?term=728769) | 1.8 | 0.00007 | 135.6 | 72.1 |
| OSGIN1 | oxidative stress induced growth inhibitor 1 | [29948](https://www.ncbi.nlm.nih.gov/gene/?term=29948) | 1.8 | 0.02083 | 18.9 | 9.1 |
| HIST1H3A | histone cluster 1, H3a | [8350](https://www.ncbi.nlm.nih.gov/gene/?term=8350) | 1.8 | 0.00249 | 289.4 | 149.0 |
| SNORD116-13 | small nucleolar RNA, C/D box 116-13 | [100033425](https://www.ncbi.nlm.nih.gov/gene/?term=100033425) | 1.8 | 0.01363 | 13.7 | 6.7 |
| CNIH3 | cornichon family AMPA receptor auxiliary protein 3 | [149111](https://www.ncbi.nlm.nih.gov/gene/?term=149111) | -1.8 | 0.01426 | 186.1 | 389.0 |
| MMP9 | matrix metallopeptidase 9 | [4318](https://www.ncbi.nlm.nih.gov/gene/?term=4318) | 1.8 | 0.02927 | 1002.9 | 439.4 |
| LOC107986369 |  | [107986369](https://www.ncbi.nlm.nih.gov/gene/?term=107986369) | -1.8 | 0.01883 | 8.5 | 18.8 |
| ABCA13 | ATP binding cassette subfamily A member 13 | [154664](https://www.ncbi.nlm.nih.gov/gene/?term=154664) | 1.8 | 0.01559 | 666.4 | 317.8 |
| FAM21C | family with sequence similarity 21 member C | [253725](https://www.ncbi.nlm.nih.gov/gene/?term=253725) | -1.8 | 0.00083 | 1359.8 | 2588.9 |
| RPS12P27 | ribosomal protein S12 pseudogene 27 | [100271552](https://www.ncbi.nlm.nih.gov/gene/?term=100271552) | -1.8 | 0.04166 | 3.2 | 9.1 |
| BST1 | bone marrow stromal cell antigen 1 | [683](https://www.ncbi.nlm.nih.gov/gene/?term=683) | 1.8 | 0.00082 | 2143.3 | 1136.2 |
| HIST1H4D | histone cluster 1, H4d | [8360](https://www.ncbi.nlm.nih.gov/gene/?term=8360) | 1.8 | 0.00350 | 1013.5 | 518.0 |
| PIK3C2B | phosphatidylinositol-4-phosphate 3-kinase catalytic subunit type 2 beta | [5287](https://www.ncbi.nlm.nih.gov/gene/?term=5287) | -1.8 | 0.00161 | 173.3 | 333.6 |
| COBLL1 | cordon-bleu WH2 repeat protein like 1 | [22837](https://www.ncbi.nlm.nih.gov/gene/?term=22837) | -1.8 | 0.00018 | 475.3 | 887.5 |
| IGIP | IgA-inducing protein | [492311](https://www.ncbi.nlm.nih.gov/gene/?term=492311) | -1.8 | 0.00006 | 130.0 | 243.3 |
| SLC39A1 | solute carrier family 39 member 1 | [27173](https://www.ncbi.nlm.nih.gov/gene/?term=27173) | 1.8 | 0.00000 | 881.3 | 481.8 |
| MNDA | myeloid cell nuclear differentiation antigen | [4332](https://www.ncbi.nlm.nih.gov/gene/?term=4332) | 1.8 | 0.00101 | 58034.2 | 30496.1 |
| ZBTB20 | zinc finger and BTB domain containing 20 | [26137](https://www.ncbi.nlm.nih.gov/gene/?term=26137) | -1.8 | 0.00000 | 1318.8 | 2412.9 |
| GOLGA8B | golgin A8 family member B | [440270](https://www.ncbi.nlm.nih.gov/gene/?term=440270) | -1.8 | 0.00518 | 323.0 | 633.1 |
| POMP | proteasome maturation protein | [51371](https://www.ncbi.nlm.nih.gov/gene/?term=51371) | 1.8 | 0.00001 | 1193.2 | 649.8 |
| HDAC2 | histone deacetylase 2 | [3066](https://www.ncbi.nlm.nih.gov/gene/?term=3066) | -1.8 | 0.00021 | 397.3 | 743.9 |
| SLC6A12 | solute carrier family 6 member 12 | [6539](https://www.ncbi.nlm.nih.gov/gene/?term=6539) | 1.8 | 0.00492 | 99.8 | 50.7 |
| GPR63 | G protein-coupled receptor 63 | [81491](https://www.ncbi.nlm.nih.gov/gene/?term=81491) | -1.8 | 0.01664 | 7.3 | 16.0 |
| TSHZ2 | teashirt zinc finger homeobox 2 | [128553](https://www.ncbi.nlm.nih.gov/gene/?term=128553) | -1.8 | 0.00968 | 37.2 | 76.2 |
| DMD | dystrophin | [1756](https://www.ncbi.nlm.nih.gov/gene/?term=1756) | -1.8 | 0.01577 | 18.5 | 38.7 |
| LYG1 | lysozyme g1 | [129530](https://www.ncbi.nlm.nih.gov/gene/?term=129530) | 1.8 | 0.00019 | 40.0 | 21.2 |
| SGMS1-AS1 | SGMS1 antisense RNA 1 | [104355295](https://www.ncbi.nlm.nih.gov/gene/?term=104355295) | -1.8 | 0.00001 | 85.5 | 157.0 |
| SNORA72 | small nucleolar RNA, H/ACA box 72 | [26775](https://www.ncbi.nlm.nih.gov/gene/?term=26775) | -1.8 | 0.00073 | 8.8 | 17.1 |
| CD300C | CD300c molecule | [10871](https://www.ncbi.nlm.nih.gov/gene/?term=10871) | 1.8 | 0.00069 | 318.8 | 168.9 |
| RSC1A1 | regulatory solute carrier protein, family 1, member 1 | [6248](https://www.ncbi.nlm.nih.gov/gene/?term=6248) | -1.8 | 0.00216 | 671.0 | 1293.2 |
| LOC105373832 |  | [105373832](https://www.ncbi.nlm.nih.gov/gene/?term=105373832) | -1.8 | 0.02606 | 10.3 | 23.5 |
| ANXA2 | annexin A2 | [302](https://www.ncbi.nlm.nih.gov/gene/?term=302) | 1.8 | 0.00011 | 7745.2 | 4187.0 |
| TUFT1 | tuftelin 1 | [7286](https://www.ncbi.nlm.nih.gov/gene/?term=7286) | 1.8 | 0.00214 | 167.8 | 87.6 |
| MIB2 | mindbomb E3 ubiquitin protein ligase 2 | [142678](https://www.ncbi.nlm.nih.gov/gene/?term=142678) | 1.8 | 0.00022 | 438.9 | 235.3 |
| ANXA1 | annexin A1 | [301](https://www.ncbi.nlm.nih.gov/gene/?term=301) | 1.8 | 0.00109 | 11596.2 | 6168.3 |
| KMT2A | lysine (K)-specific methyltransferase 2A | [4297](https://www.ncbi.nlm.nih.gov/gene/?term=4297) | -1.8 | 0.00156 | 1693.1 | 3225.3 |
| LOC105370124 |  | [105370124](https://www.ncbi.nlm.nih.gov/gene/?term=105370124) | -1.8 | 0.03325 | 65.8 | 155.7 |
| ADAM23 | ADAM metallopeptidase domain 23 | [8745](https://www.ncbi.nlm.nih.gov/gene/?term=8745) | -1.8 | 0.00870 | 24.8 | 49.7 |
| AP2B1 | adaptor related protein complex 2 beta 1 subunit | [163](https://www.ncbi.nlm.nih.gov/gene/?term=163) | -1.8 | 0.00480 | 10477.4 | 20639.5 |
| HIST1H2BM | histone cluster 1, H2bm | [8342](https://www.ncbi.nlm.nih.gov/gene/?term=8342) | 1.8 | 0.00817 | 541.2 | 271.2 |
| LOC105373175 |  | [105373175](https://www.ncbi.nlm.nih.gov/gene/?term=105373175) | -1.8 | 0.01004 | 19.9 | 40.6 |
| LOC101927245 |  | [101927245](https://www.ncbi.nlm.nih.gov/gene/?term=101927245) | 1.8 | 0.02854 | 10.7 | 5.1 |
| IL4I1 | interleukin 4 induced 1 | [259307](https://www.ncbi.nlm.nih.gov/gene/?term=259307) | 1.8 | 0.01765 | 28.0 | 13.3 |
| TUBA1B | tubulin alpha 1b | [10376](https://www.ncbi.nlm.nih.gov/gene/?term=10376) | 1.8 | 0.00000 | 6318.2 | 3478.4 |
| SLC9C1 | solute carrier family 9 member C1 | [285335](https://www.ncbi.nlm.nih.gov/gene/?term=285335) | -1.8 | 0.03262 | 23.5 | 54.1 |
| CD82 | CD82 molecule | [3732](https://www.ncbi.nlm.nih.gov/gene/?term=3732) | 1.8 | 0.00097 | 1687.0 | 899.3 |
| AIFM3 | apoptosis inducing factor, mitochondria associated 3 | [150209](https://www.ncbi.nlm.nih.gov/gene/?term=150209) | 1.8 | 0.00968 | 80.1 | 39.5 |
| LOC105369628 |  | [105369628](https://www.ncbi.nlm.nih.gov/gene/?term=105369628) | 1.8 | 0.03134 | 24.1 | 10.8 |
| FAM26F | family with sequence similarity 26 member F | [441168](https://www.ncbi.nlm.nih.gov/gene/?term=441168) | 1.8 | 0.01527 | 815.0 | 394.6 |
| DDX6 | DEAD-box helicase 6 | [1656](https://www.ncbi.nlm.nih.gov/gene/?term=1656) | -1.8 | 0.00004 | 14975.9 | 27482.9 |
| C3orf14 | chromosome 3 open reading frame 14 | [57415](https://www.ncbi.nlm.nih.gov/gene/?term=57415) | 1.8 | 0.00641 | 40.5 | 20.5 |
| LOC101928389 | uncharacterized LOC101928389 | [101928389](https://www.ncbi.nlm.nih.gov/gene/?term=101928389) | -1.8 | 0.01256 | 11.9 | 25.0 |
| EMILIN1 | elastin microfibril interfacer 1 | [11117](https://www.ncbi.nlm.nih.gov/gene/?term=11117) | 1.8 | 0.01117 | 45.8 | 22.5 |
| CCDC88B | coiled-coil domain containing 88B | [283234](https://www.ncbi.nlm.nih.gov/gene/?term=283234) | 1.8 | 0.00111 | 3888.3 | 2059.2 |
| DLGAP4 | discs large homolog associated protein 4 | [22839](https://www.ncbi.nlm.nih.gov/gene/?term=22839) | -1.8 | 0.00860 | 42.9 | 86.6 |
| SNX10 | sorting nexin 10 | [29887](https://www.ncbi.nlm.nih.gov/gene/?term=29887) | 1.8 | 0.00021 | 4525.4 | 2439.7 |
| AGBL3 | ATP/GTP binding protein-like 3 | [340351](https://www.ncbi.nlm.nih.gov/gene/?term=340351) | -1.8 | 0.00114 | 35.0 | 66.9 |
| LOC107985000 |  | [107985000](https://www.ncbi.nlm.nih.gov/gene/?term=107985000) | -1.8 | 0.01133 | 7.8 | 14.7 |
| BCORL1 | BCL6 corepressor-like 1 | [63035](https://www.ncbi.nlm.nih.gov/gene/?term=63035) | -1.8 | 0.01087 | 43.2 | 88.9 |
| WI2-87327B8.2 |  | [105372712](https://www.ncbi.nlm.nih.gov/gene/?term=105372712) | 1.8 | 0.02483 | 12.5 | 5.4 |
| TRNS1 |  | [4574](https://www.ncbi.nlm.nih.gov/gene/?term=4574) | -1.8 | 0.01926 | 16.8 | 34.3 |
| ST3GAL5-AS1 | ST3GAL5 antisense RNA 1 (head to head) | [101928113](https://www.ncbi.nlm.nih.gov/gene/?term=101928113) | 1.8 | 0.01558 | 20.6 | 10.1 |
| ADCK4 | aarF domain containing kinase 4 | [79934](https://www.ncbi.nlm.nih.gov/gene/?term=79934) | 1.8 | 0.00000 | 388.9 | 215.6 |
| NDUFAF4P1 | NADH:ubiquinone oxidoreductase complex assembly factor 4 pseudogene 1 | [100306975](https://www.ncbi.nlm.nih.gov/gene/?term=100306975) | -1.8 | 0.00105 | 19.6 | 38.6 |
| C9orf139 | chromosome 9 open reading frame 139 | [401563](https://www.ncbi.nlm.nih.gov/gene/?term=401563) | 1.8 | 0.00128 | 207.5 | 109.6 |
| LOC105379177 |  | [105379177](https://www.ncbi.nlm.nih.gov/gene/?term=105379177) | -1.8 | 0.01983 | 21.5 | 46.2 |
| CLEC4A | C-type lectin domain family 4 member A | [50856](https://www.ncbi.nlm.nih.gov/gene/?term=50856) | 1.8 | 0.00007 | 2373.0 | 1295.1 |
| BRAF | B-Raf proto-oncogene, serine/threonine kinase | [673](https://www.ncbi.nlm.nih.gov/gene/?term=673) | -1.8 | 0.00000 | 1622.1 | 2908.7 |
| LMO7 | LIM domain 7 | [4008](https://www.ncbi.nlm.nih.gov/gene/?term=4008) | -1.8 | 0.00014 | 128.8 | 238.4 |
| TMPPE | transmembrane protein with metallophosphoesterase domain | [643853](https://www.ncbi.nlm.nih.gov/gene/?term=643853) | -1.8 | 0.00027 | 301.1 | 558.0 |
| LOC107985977 |  | [107985977](https://www.ncbi.nlm.nih.gov/gene/?term=107985977) | -1.8 | 0.01596 | 26.1 | 54.5 |
| LPL | lipoprotein lipase | [4023](https://www.ncbi.nlm.nih.gov/gene/?term=4023) | -1.8 | 0.04510 | 12.2 | 36.3 |
| LOC105369360 |  | [105369360](https://www.ncbi.nlm.nih.gov/gene/?term=105369360) | -1.8 | 0.01135 | 5.1 | 10.6 |
| RHBDD3 | rhomboid domain containing 3 | [25807](https://www.ncbi.nlm.nih.gov/gene/?term=25807) | 1.8 | 0.00001 | 147.9 | 81.6 |
| THEMIS2 | thymocyte selection associated family member 2 | [9473](https://www.ncbi.nlm.nih.gov/gene/?term=9473) | 1.8 | 0.00004 | 9135.4 | 5003.9 |
| NCAPGP1 | non-SMC condensin I complex subunit G pseudogene 1 | [100421096](https://www.ncbi.nlm.nih.gov/gene/?term=100421096) | -1.8 | 0.03671 | 6.4 | 16.1 |
| LOC100506071 | uncharacterized LOC100506071 | [100506071](https://www.ncbi.nlm.nih.gov/gene/?term=100506071) | -1.8 | 0.00517 | 12.3 | 24.5 |
| TERB1 | coiled-coil domain containing 79 | [283847](https://www.ncbi.nlm.nih.gov/gene/?term=283847) | -1.8 | 0.00911 | 6.6 | 13.8 |
| SEC14L2 | SEC14 like lipid binding 2 | [23541](https://www.ncbi.nlm.nih.gov/gene/?term=23541) | 1.8 | 0.01626 | 85.8 | 41.2 |
| ZMAT5 | zinc finger matrin-type 5 | [55954](https://www.ncbi.nlm.nih.gov/gene/?term=55954) | 1.8 | 0.00000 | 292.5 | 161.6 |
| ZNF805 | zinc finger protein 805 | [390980](https://www.ncbi.nlm.nih.gov/gene/?term=390980) | -1.8 | 0.00000 | 731.7 | 1311.8 |
| NASP | nuclear autoantigenic sperm protein | [4678](https://www.ncbi.nlm.nih.gov/gene/?term=4678) | 1.8 | 0.00000 | 1380.3 | 767.5 |
| FAM98B | family with sequence similarity 98 member B | [283742](https://www.ncbi.nlm.nih.gov/gene/?term=283742) | -1.8 | 0.00035 | 188.9 | 350.8 |
| FOXP3 | forkhead box P3 | [50943](https://www.ncbi.nlm.nih.gov/gene/?term=50943) | -1.8 | 0.02834 | 6.6 | 15.6 |
| LOC102723694 |  | [102723694](https://www.ncbi.nlm.nih.gov/gene/?term=102723694) | -1.8 | 0.00339 | 142.7 | 275.3 |
| BICD1 | BICD cargo adaptor 1 | [636](https://www.ncbi.nlm.nih.gov/gene/?term=636) | -1.8 | 0.00027 | 121.9 | 224.8 |
| MAP2K5 | mitogen-activated protein kinase kinase 5 | [5607](https://www.ncbi.nlm.nih.gov/gene/?term=5607) | -1.8 | 0.00011 | 174.6 | 321.3 |
| ZNF827 | zinc finger protein 827 | [152485](https://www.ncbi.nlm.nih.gov/gene/?term=152485) | -1.8 | 0.01034 | 73.8 | 149.0 |
| SLITRK4 | SLIT and NTRK like family member 4 | [139065](https://www.ncbi.nlm.nih.gov/gene/?term=139065) | 1.8 | 0.00031 | 228.1 | 123.6 |
| RPS10P1 | ribosomal protein S10 pseudogene 1 | [394255](https://www.ncbi.nlm.nih.gov/gene/?term=394255) | 1.8 | 0.00468 | 80.6 | 41.8 |
| PFKFB4 | 6-phosphofructo-2-kinase/fructose-2,6-biphosphatase 4 | [5210](https://www.ncbi.nlm.nih.gov/gene/?term=5210) | 1.8 | 0.00015 | 1959.1 | 1066.0 |
| ARHGAP17 | Rho GTPase activating protein 17 | [55114](https://www.ncbi.nlm.nih.gov/gene/?term=55114) | -1.8 | 0.00893 | 281.8 | 557.1 |
| HIST1H2BH | histone cluster 1, H2bh | [8345](https://www.ncbi.nlm.nih.gov/gene/?term=8345) | 1.8 | 0.00421 | 1183.8 | 614.5 |
| LOC105376577 |  | [105376577](https://www.ncbi.nlm.nih.gov/gene/?term=105376577) | -1.8 | 0.00178 | 53.2 | 101.7 |
| NDUFAF1 | NADH:ubiquinone oxidoreductase complex assembly factor 1 | [51103](https://www.ncbi.nlm.nih.gov/gene/?term=51103) | 1.8 | 0.00000 | 313.4 | 174.3 |
| FLOT1 | flotillin 1 | [10211](https://www.ncbi.nlm.nih.gov/gene/?term=10211) | 1.8 | 0.02398 | 22.3 | 10.7 |
| GMFG | glia maturation factor gamma | [9535](https://www.ncbi.nlm.nih.gov/gene/?term=9535) | 1.8 | 0.00013 | 7081.6 | 3867.4 |
| SMARCD3 | SWI/SNF related, matrix associated, actin dependent regulator of chromatin, subfamily d, member 3 | [6604](https://www.ncbi.nlm.nih.gov/gene/?term=6604) | 1.8 | 0.00011 | 334.8 | 182.8 |
| ENPEP | glutamyl aminopeptidase | [2028](https://www.ncbi.nlm.nih.gov/gene/?term=2028) | -1.8 | 0.04360 | 4.2 | 13.3 |
| ZNF391 | zinc finger protein 391 | [346157](https://www.ncbi.nlm.nih.gov/gene/?term=346157) | -1.8 | 0.02118 | 12.1 | 26.5 |
| SMAD6 | SMAD family member 6 | [4091](https://www.ncbi.nlm.nih.gov/gene/?term=4091) | 1.7 | 0.02978 | 9.4 | 4.1 |
| LOC107983962 |  | [107983962](https://www.ncbi.nlm.nih.gov/gene/?term=107983962) | -1.7 | 0.01685 | 191.4 | 394.7 |
| ZNF83 | zinc finger protein 83 | [55769](https://www.ncbi.nlm.nih.gov/gene/?term=55769) | -1.7 | 0.00000 | 389.8 | 698.6 |
| LOC107986202 |  | [107986202](https://www.ncbi.nlm.nih.gov/gene/?term=107986202) | -1.7 | 0.03769 | 5.9 | 14.4 |
| MFHAS1 | malignant fibrous histiocytoma amplified sequence 1 | [9258](https://www.ncbi.nlm.nih.gov/gene/?term=9258) | -1.7 | 0.00432 | 1114.8 | 2139.3 |
| PRR34 | proline rich 34 | [55267](https://www.ncbi.nlm.nih.gov/gene/?term=55267) | 1.7 | 0.01784 | 8.0 | 3.9 |
| PBX1 | pre-B-cell leukemia homeobox 1 | [5087](https://www.ncbi.nlm.nih.gov/gene/?term=5087) | -1.7 | 0.02336 | 905.9 | 1918.3 |
| LAG3 | lymphocyte activating 3 | [3902](https://www.ncbi.nlm.nih.gov/gene/?term=3902) | 1.7 | 0.03927 | 26.8 | 11.6 |
| GPR35 | G protein-coupled receptor 35 | [2859](https://www.ncbi.nlm.nih.gov/gene/?term=2859) | 1.7 | 0.00549 | 95.1 | 49.4 |
| DHRS9 | dehydrogenase/reductase (SDR family) member 9 | [10170](https://www.ncbi.nlm.nih.gov/gene/?term=10170) | 1.7 | 0.00993 | 1256.3 | 636.7 |
| MAP2K6 | mitogen-activated protein kinase kinase 6 | [5608](https://www.ncbi.nlm.nih.gov/gene/?term=5608) | 1.7 | 0.00219 | 1770.5 | 938.3 |
| E2F1 | E2F transcription factor 1 | [1869](https://www.ncbi.nlm.nih.gov/gene/?term=1869) | 1.7 | 0.00545 | 316.2 | 163.0 |
| CD68 | CD68 molecule | [968](https://www.ncbi.nlm.nih.gov/gene/?term=968) | 1.7 | 0.00023 | 4197.7 | 2285.0 |
| FOXC1 | forkhead box C1 | [2296](https://www.ncbi.nlm.nih.gov/gene/?term=2296) | 1.7 | 0.04301 | 8.2 | 3.3 |
| C16orf86 | chromosome 16 open reading frame 86 | [388284](https://www.ncbi.nlm.nih.gov/gene/?term=388284) | 1.7 | 0.00075 | 71.4 | 38.0 |
| THEM5 | thioesterase superfamily member 5 | [284486](https://www.ncbi.nlm.nih.gov/gene/?term=284486) | -1.7 | 0.04290 | 98.3 | 254.3 |
| COLGALT1 | collagen beta(1-O)galactosyltransferase 1 | [79709](https://www.ncbi.nlm.nih.gov/gene/?term=79709) | 1.7 | 0.00001 | 1616.1 | 898.8 |
| PCED1B | PC-esterase domain containing 1B | [91523](https://www.ncbi.nlm.nih.gov/gene/?term=91523) | -1.7 | 0.00139 | 176.3 | 331.3 |
| POU2AF1 | POU class 2 associating factor 1 | [5450](https://www.ncbi.nlm.nih.gov/gene/?term=5450) | -1.7 | 0.01278 | 108.8 | 219.3 |
| ARPC5 | actin related protein 2/3 complex subunit 5 | [10092](https://www.ncbi.nlm.nih.gov/gene/?term=10092) | 1.7 | 0.00016 | 15787.5 | 8628.3 |
| PRDX3 | peroxiredoxin 3 | [10935](https://www.ncbi.nlm.nih.gov/gene/?term=10935) | 1.7 | 0.00001 | 2068.0 | 1146.4 |
| LOC105369779 |  | [105369779](https://www.ncbi.nlm.nih.gov/gene/?term=105369779) | 1.7 | 0.00009 | 90.4 | 49.0 |
| FAM133DP | family with sequence similarity 133 member D, pseudogene | [728066](https://www.ncbi.nlm.nih.gov/gene/?term=728066) | -1.7 | 0.00014 | 29.2 | 53.9 |
| LOC107984875 |  | [107984875](https://www.ncbi.nlm.nih.gov/gene/?term=107984875) | 1.7 | 0.00007 | 43.8 | 24.6 |
| MEFV | Mediterranean fever | [4210](https://www.ncbi.nlm.nih.gov/gene/?term=4210) | 1.7 | 0.00047 | 5576.7 | 3042.7 |
| NFIL3 | nuclear factor, interleukin 3 regulated | [4783](https://www.ncbi.nlm.nih.gov/gene/?term=4783) | 1.7 | 0.00122 | 1583.5 | 851.4 |
| CDCA5 | cell division cycle associated 5 | [113130](https://www.ncbi.nlm.nih.gov/gene/?term=113130) | 1.7 | 0.03778 | 38.6 | 16.8 |
| ZNF438 | zinc finger protein 438 | [220929](https://www.ncbi.nlm.nih.gov/gene/?term=220929) | 1.7 | 0.00308 | 924.3 | 488.6 |
| CNR2 | cannabinoid receptor 2 (macrophage) | [1269](https://www.ncbi.nlm.nih.gov/gene/?term=1269) | -1.7 | 0.00137 | 158.3 | 294.9 |
| FAM46A | family with sequence similarity 46 member A | [55603](https://www.ncbi.nlm.nih.gov/gene/?term=55603) | 1.7 | 0.00059 | 2778.3 | 1508.9 |
| AFF3 | AF4/FMR2 family member 3 | [3899](https://www.ncbi.nlm.nih.gov/gene/?term=3899) | -1.7 | 0.00178 | 453.5 | 851.8 |
| LOC101929356 |  | [101929356](https://www.ncbi.nlm.nih.gov/gene/?term=101929356) | -1.7 | 0.00248 | 109.3 | 206.6 |
| LOC107986032 |  | [107986032](https://www.ncbi.nlm.nih.gov/gene/?term=107986032) | -1.7 | 0.02818 | 37.7 | 82.1 |
| GNA12 | G protein subunit alpha 12 | [2768](https://www.ncbi.nlm.nih.gov/gene/?term=2768) | -1.7 | 0.02483 | 778.4 | 1657.1 |
| LTF | lactotransferrin | [4057](https://www.ncbi.nlm.nih.gov/gene/?term=4057) | -1.7 | 0.04515 | 163.8 | 449.7 |
| CNEP1R1 | CTD nuclear envelope phosphatase 1 regulatory subunit 1 | [255919](https://www.ncbi.nlm.nih.gov/gene/?term=255919) | 1.7 | 0.00004 | 1051.1 | 582.9 |
| GTSE1-AS1 | GTSE1 antisense RNA 1 (head to head) | [150384](https://www.ncbi.nlm.nih.gov/gene/?term=150384) | 1.7 | 0.01004 | 16.6 | 8.4 |
| DHRS7B | dehydrogenase/reductase (SDR family) member 7B | [25979](https://www.ncbi.nlm.nih.gov/gene/?term=25979) | 1.7 | 0.00000 | 313.3 | 174.8 |
| GNB1L | G protein subunit beta 1 like | [54584](https://www.ncbi.nlm.nih.gov/gene/?term=54584) | 1.7 | 0.00016 | 67.2 | 36.7 |
| ZFP3 | ZFP3 zinc finger protein | [124961](https://www.ncbi.nlm.nih.gov/gene/?term=124961) | -1.7 | 0.00140 | 85.9 | 161.1 |
| ITPKC | inositol-trisphosphate 3-kinase C | [80271](https://www.ncbi.nlm.nih.gov/gene/?term=80271) | 1.7 | 0.00001 | 273.1 | 152.9 |
| LOC102724745 |  | [102724745](https://www.ncbi.nlm.nih.gov/gene/?term=102724745) | -1.7 | 0.00206 | 25.6 | 47.9 |
| CD74 | CD74 molecule | [972](https://www.ncbi.nlm.nih.gov/gene/?term=972) | -1.7 | 0.00628 | 3769.8 | 7218.1 |
| MTMR11 | myotubularin related protein 11 | [10903](https://www.ncbi.nlm.nih.gov/gene/?term=10903) | 1.7 | 0.00070 | 374.8 | 202.8 |
| CEBPA-AS1 | CEBPA antisense RNA 1 (head to head) | [80054](https://www.ncbi.nlm.nih.gov/gene/?term=80054) | 1.7 | 0.00413 | 37.5 | 19.6 |
| LRIT3 | leucine-rich repeat, Ig-like and transmembrane domains 3 | [345193](https://www.ncbi.nlm.nih.gov/gene/?term=345193) | -1.7 | 0.04667 | 3.1 | 8.2 |
| UBE2L6 | ubiquitin conjugating enzyme E2 L6 | [9246](https://www.ncbi.nlm.nih.gov/gene/?term=9246) | 1.7 | 0.00563 | 2417.0 | 1261.2 |
| EPHB2 | EPH receptor B2 | [2048](https://www.ncbi.nlm.nih.gov/gene/?term=2048) | 1.7 | 0.02789 | 85.8 | 40.4 |
| SNORD55 | small nucleolar RNA, C/D box 55 | [26811](https://www.ncbi.nlm.nih.gov/gene/?term=26811) | 1.7 | 0.00174 | 26.9 | 13.7 |
| RALGAPA1P1 | Ral GTPase activating protein catalytic alpha subunit 1 pseudogene 1 | [26134](https://www.ncbi.nlm.nih.gov/gene/?term=26134) | -1.7 | 0.00158 | 28.5 | 53.7 |
| LOC102546294 | uncharacterized LOC102546294 | [102546294](https://www.ncbi.nlm.nih.gov/gene/?term=102546294) | 1.7 | 0.02128 | 18.2 | 8.9 |
| ZNF248 | zinc finger protein 248 | [57209](https://www.ncbi.nlm.nih.gov/gene/?term=57209) | -1.7 | 0.00000 | 171.9 | 306.9 |
| UBXN10 | UBX domain protein 10 | [127733](https://www.ncbi.nlm.nih.gov/gene/?term=127733) | -1.7 | 0.01154 | 390.4 | 766.8 |
| TRK-TTT3-1 |  | [100189122](https://www.ncbi.nlm.nih.gov/gene/?term=100189122) | 1.7 | 0.01472 | 12.8 | 6.2 |
| IMPG2 | interphotoreceptor matrix proteoglycan 2 | [50939](https://www.ncbi.nlm.nih.gov/gene/?term=50939) | -1.7 | 0.00002 | 69.9 | 124.0 |
| RUNX2 | runt related transcription factor 2 | [860](https://www.ncbi.nlm.nih.gov/gene/?term=860) | -1.7 | 0.00491 | 473.4 | 905.1 |
| PSMA4 | proteasome subunit alpha 4 | [5685](https://www.ncbi.nlm.nih.gov/gene/?term=5685) | 1.7 | 0.00000 | 2573.4 | 1447.7 |
| CDC25A | cell division cycle 25A | [993](https://www.ncbi.nlm.nih.gov/gene/?term=993) | 1.7 | 0.02927 | 82.4 | 38.3 |
| AAK1 | AP2 associated kinase 1 | [22848](https://www.ncbi.nlm.nih.gov/gene/?term=22848) | -1.7 | 0.00801 | 1075.7 | 2086.7 |
| MYO5C | myosin VC | [55930](https://www.ncbi.nlm.nih.gov/gene/?term=55930) | -1.7 | 0.00283 | 12.7 | 24.8 |
| BAG6 | BCL2 associated athanogene 6 | [7917](https://www.ncbi.nlm.nih.gov/gene/?term=7917) | -1.7 | 0.00628 | 46.1 | 88.6 |
| RNF10 | ring finger protein 10 | [9921](https://www.ncbi.nlm.nih.gov/gene/?term=9921) | -1.7 | 0.02013 | 37989.1 | 81636.6 |
| LOC105369140 |  | [105369140](https://www.ncbi.nlm.nih.gov/gene/?term=105369140) | 1.7 | 0.04701 | 32.3 | 12.7 |
| ZNF346 | zinc finger protein 346 | [23567](https://www.ncbi.nlm.nih.gov/gene/?term=23567) | -1.7 | 0.00506 | 995.0 | 1891.5 |
| CATSPER1 | cation channel sperm associated 1 | [117144](https://www.ncbi.nlm.nih.gov/gene/?term=117144) | 1.7 | 0.00026 | 87.7 | 47.7 |
| PAX5 | paired box 5 | [5079](https://www.ncbi.nlm.nih.gov/gene/?term=5079) | -1.7 | 0.00929 | 368.5 | 717.7 |
| ZNF519 | zinc finger protein 519 | [162655](https://www.ncbi.nlm.nih.gov/gene/?term=162655) | -1.7 | 0.00264 | 80.2 | 151.7 |
| SLC2A6 | solute carrier family 2 member 6 | [11182](https://www.ncbi.nlm.nih.gov/gene/?term=11182) | 1.7 | 0.02988 | 12.5 | 5.6 |
| FBXO48 | F-box protein 48 | [554251](https://www.ncbi.nlm.nih.gov/gene/?term=554251) | -1.7 | 0.00080 | 228.0 | 416.2 |
| PELO | pelota homolog (Drosophila) | [53918](https://www.ncbi.nlm.nih.gov/gene/?term=53918) | 1.7 | 0.00003 | 438.8 | 245.9 |
| AGO3 | argonaute 3, RISC catalytic component | [192669](https://www.ncbi.nlm.nih.gov/gene/?term=192669) | -1.7 | 0.00006 | 891.6 | 1606.1 |
| LOC107986115 |  | [107986115](https://www.ncbi.nlm.nih.gov/gene/?term=107986115) | -1.7 | 0.01680 | 13.8 | 29.3 |
| LOC107984316 |  | [107984316](https://www.ncbi.nlm.nih.gov/gene/?term=107984316) | -1.7 | 0.03740 | 8.7 | 20.5 |
| NHS | NHS actin remodeling regulator | [4810](https://www.ncbi.nlm.nih.gov/gene/?term=4810) | -1.7 | 0.00216 | 96.4 | 180.7 |
| LOC105378122 |  | [105378122](https://www.ncbi.nlm.nih.gov/gene/?term=105378122) | -1.7 | 0.01837 | 15.3 | 31.0 |
| LOC101929289 |  | [101929289](https://www.ncbi.nlm.nih.gov/gene/?term=101929289) | 1.7 | 0.00005 | 39.3 | 21.2 |
| ABHD14B | abhydrolase domain containing 14B | [84836](https://www.ncbi.nlm.nih.gov/gene/?term=84836) | -1.7 | 0.00023 | 135.8 | 247.1 |
| ARMC12 | armadillo repeat containing 12 | [221481](https://www.ncbi.nlm.nih.gov/gene/?term=221481) | 1.7 | 0.01182 | 15.6 | 7.9 |
| LOC107984778 |  | [107984778](https://www.ncbi.nlm.nih.gov/gene/?term=107984778) | 1.7 | 0.03497 | 34.9 | 15.9 |
| CHAMP1 | chromosome alignment maintaining phosphoprotein 1 | [283489](https://www.ncbi.nlm.nih.gov/gene/?term=283489) | -1.7 | 0.01514 | 69.9 | 141.4 |
| LOC107986006 |  | [107986006](https://www.ncbi.nlm.nih.gov/gene/?term=107986006) | -1.7 | 0.03429 | 4.6 | 10.8 |
| IKZF5 | IKAROS family zinc finger 5 | [64376](https://www.ncbi.nlm.nih.gov/gene/?term=64376) | -1.7 | 0.00025 | 386.7 | 702.4 |
| GK | glycerol kinase | [2710](https://www.ncbi.nlm.nih.gov/gene/?term=2710) | 1.7 | 0.00690 | 5252.9 | 2747.7 |
| WDPCP | WD repeat containing planar cell polarity effector | [51057](https://www.ncbi.nlm.nih.gov/gene/?term=51057) | -1.7 | 0.00000 | 167.8 | 296.4 |
| TPD52L2 | tumor protein D52 like 2 | [7165](https://www.ncbi.nlm.nih.gov/gene/?term=7165) | 1.7 | 0.00005 | 4094.2 | 2281.0 |
| GGH | gamma-glutamyl hydrolase | [8836](https://www.ncbi.nlm.nih.gov/gene/?term=8836) | 1.7 | 0.00689 | 106.7 | 55.8 |
| AGPAT2 | 1-acylglycerol-3-phosphate O-acyltransferase 2 | [10555](https://www.ncbi.nlm.nih.gov/gene/?term=10555) | 1.7 | 0.00005 | 487.1 | 272.2 |
| TMEM241 | transmembrane protein 241 | [85019](https://www.ncbi.nlm.nih.gov/gene/?term=85019) | -1.7 | 0.00101 | 66.7 | 122.4 |
| STYXL1 | serine/threonine/tyrosine interacting-like 1 | [51657](https://www.ncbi.nlm.nih.gov/gene/?term=51657) | 1.7 | 0.00005 | 399.9 | 223.1 |
| NUDT22 | nudix hydrolase 22 | [84304](https://www.ncbi.nlm.nih.gov/gene/?term=84304) | 1.7 | 0.00000 | 369.1 | 209.4 |
| B9D2 | B9 protein domain 2 | [80776](https://www.ncbi.nlm.nih.gov/gene/?term=80776) | 1.7 | 0.00017 | 281.7 | 156.4 |
| SIGLEC15 | sialic acid binding Ig like lectin 15 | [284266](https://www.ncbi.nlm.nih.gov/gene/?term=284266) | 1.7 | 0.02815 | 14.3 | 7.0 |
| PTGIR | prostaglandin I2 (prostacyclin) receptor (IP) | [5739](https://www.ncbi.nlm.nih.gov/gene/?term=5739) | 1.7 | 0.00115 | 283.1 | 153.7 |
| TRNP1 | TMF1-regulated nuclear protein 1 | [388610](https://www.ncbi.nlm.nih.gov/gene/?term=388610) | 1.7 | 0.02953 | 14.6 | 6.8 |
| SSTR3 | somatostatin receptor 3 | [6753](https://www.ncbi.nlm.nih.gov/gene/?term=6753) | 1.7 | 0.00575 | 172.0 | 90.9 |
| UBBP1 | ubiquitin B pseudogene 1 | [7315](https://www.ncbi.nlm.nih.gov/gene/?term=7315) | -1.7 | 0.03772 | 3.5 | 6.9 |
| SLC12A9 | solute carrier family 12 member 9 | [56996](https://www.ncbi.nlm.nih.gov/gene/?term=56996) | 1.7 | 0.00083 | 1078.6 | 590.7 |
| IGFBP7 | insulin like growth factor binding protein 7 | [3490](https://www.ncbi.nlm.nih.gov/gene/?term=3490) | 1.7 | 0.00151 | 358.0 | 194.0 |
| NT5C3A | 5'-nucleotidase, cytosolic IIIA | [51251](https://www.ncbi.nlm.nih.gov/gene/?term=51251) | 1.7 | 0.00146 | 7260.6 | 3959.1 |
| FZD2 | frizzled class receptor 2 | [2535](https://www.ncbi.nlm.nih.gov/gene/?term=2535) | 1.7 | 0.00426 | 150.0 | 79.5 |
| IFNGR1 | interferon gamma receptor 1 | [3459](https://www.ncbi.nlm.nih.gov/gene/?term=3459) | 1.7 | 0.00068 | 10247.1 | 5611.2 |
| LOC101926886 |  | [101926886](https://www.ncbi.nlm.nih.gov/gene/?term=101926886) | -1.7 | 0.00344 | 10.9 | 21.1 |
| LILRA2 | leukocyte immunoglobulin like receptor A2 | [11027](https://www.ncbi.nlm.nih.gov/gene/?term=11027) | 1.7 | 0.04188 | 24.0 | 10.7 |
| NOP10 | NOP10 ribonucleoprotein | [55505](https://www.ncbi.nlm.nih.gov/gene/?term=55505) | 1.7 | 0.00106 | 2775.3 | 1516.1 |
| TRNY |  | [4579](https://www.ncbi.nlm.nih.gov/gene/?term=4579) | -1.7 | 0.02945 | 5.5 | 11.7 |
| LOC107984220 |  | [107984220](https://www.ncbi.nlm.nih.gov/gene/?term=107984220) | -1.7 | 0.03025 | 13.3 | 29.6 |
| ARID1A | AT-rich interaction domain 1A | [8289](https://www.ncbi.nlm.nih.gov/gene/?term=8289) | -1.7 | 0.00888 | 681.6 | 1326.2 |
| EFHD2 | EF-hand domain family member D2 | [79180](https://www.ncbi.nlm.nih.gov/gene/?term=79180) | 1.7 | 0.00014 | 4025.3 | 2237.5 |
| RASSF4 | Ras association domain family member 4 | [83937](https://www.ncbi.nlm.nih.gov/gene/?term=83937) | 1.7 | 0.00060 | 1013.9 | 556.2 |
| ZNF775 | zinc finger protein 775 | [285971](https://www.ncbi.nlm.nih.gov/gene/?term=285971) | 1.7 | 0.00009 | 87.3 | 48.6 |
| CYP1B1 | cytochrome P450 family 1 subfamily B member 1 | [1545](https://www.ncbi.nlm.nih.gov/gene/?term=1545) | 1.7 | 0.01611 | 1717.2 | 863.5 |
| SPATA2L | spermatogenesis associated 2 like | [124044](https://www.ncbi.nlm.nih.gov/gene/?term=124044) | 1.7 | 0.00014 | 159.2 | 89.4 |
| CCNB2 | cyclin B2 | [9133](https://www.ncbi.nlm.nih.gov/gene/?term=9133) | 1.7 | 0.02574 | 161.7 | 77.8 |
| BNIP3L | BCL2/adenovirus E1B 19kDa interacting protein 3-like | [665](https://www.ncbi.nlm.nih.gov/gene/?term=665) | -1.7 | 0.01726 | 358046.8 | 720160.4 |
| HIST1H2AK | histone cluster 1, H2ak | [8330](https://www.ncbi.nlm.nih.gov/gene/?term=8330) | 1.7 | 0.00260 | 271.6 | 146.5 |
| PPP3CB | protein phosphatase 3 catalytic subunit beta | [5532](https://www.ncbi.nlm.nih.gov/gene/?term=5532) | -1.7 | 0.00001 | 1482.7 | 2630.7 |
| BAK1 | BCL2 antagonist/killer 1 | [578](https://www.ncbi.nlm.nih.gov/gene/?term=578) | 1.7 | 0.00022 | 832.5 | 461.8 |
| CD22 | CD22 molecule | [933](https://www.ncbi.nlm.nih.gov/gene/?term=933) | -1.7 | 0.00476 | 401.6 | 756.7 |
| HIST1H2AM | histone cluster 1, H2am | [8336](https://www.ncbi.nlm.nih.gov/gene/?term=8336) | 1.7 | 0.00485 | 924.5 | 491.7 |
| CDC6 | cell division cycle 6 | [990](https://www.ncbi.nlm.nih.gov/gene/?term=990) | 1.7 | 0.01579 | 168.8 | 85.2 |
| MAP3K9 | mitogen-activated protein kinase kinase kinase 9 | [4293](https://www.ncbi.nlm.nih.gov/gene/?term=4293) | -1.7 | 0.00821 | 37.0 | 71.3 |
| TMPRSS5 | transmembrane protease, serine 5 | [80975](https://www.ncbi.nlm.nih.gov/gene/?term=80975) | -1.7 | 0.02256 | 5.6 | 12.5 |
| TNIP2 | TNFAIP3 interacting protein 2 | [79155](https://www.ncbi.nlm.nih.gov/gene/?term=79155) | 1.7 | 0.00000 | 847.6 | 483.5 |
| HIST1H4K | histone cluster 1, H4k | [8362](https://www.ncbi.nlm.nih.gov/gene/?term=8362) | 1.7 | 0.00127 | 86.0 | 46.7 |
| RABGAP1L | RAB GTPase activating protein 1-like | [9910](https://www.ncbi.nlm.nih.gov/gene/?term=9910) | -1.7 | 0.00097 | 10929.6 | 19934.7 |
| ADAMTSL4-AS1 | ADAMTSL4 antisense RNA 1 | [574406](https://www.ncbi.nlm.nih.gov/gene/?term=574406) | 1.7 | 0.01014 | 371.9 | 192.9 |
| SIRT7 | sirtuin 7 | [51547](https://www.ncbi.nlm.nih.gov/gene/?term=51547) | 1.7 | 0.00000 | 1011.1 | 577.7 |
| S100A13 | S100 calcium binding protein A13 | [6284](https://www.ncbi.nlm.nih.gov/gene/?term=6284) | 1.7 | 0.00035 | 46.2 | 25.6 |
| LOC100996740 |  | [100996740](https://www.ncbi.nlm.nih.gov/gene/?term=100996740) | 1.7 | 0.00059 | 55.2 | 29.9 |
| FTH1P16 | ferritin, heavy polypeptide 1 pseudogene 16 | [2508](https://www.ncbi.nlm.nih.gov/gene/?term=2508) | -1.7 | 0.02013 | 9.6 | 19.2 |
| FAM129A | family with sequence similarity 129 member A | [116496](https://www.ncbi.nlm.nih.gov/gene/?term=116496) | 1.7 | 0.00566 | 25621.9 | 13617.2 |
| LITAF | lipopolysaccharide induced TNF factor | [9516](https://www.ncbi.nlm.nih.gov/gene/?term=9516) | 1.7 | 0.00419 | 20392.4 | 10881.0 |
| SLC9A7P1 | solute carrier family 9 member 7 pseudogene 1 | [121456](https://www.ncbi.nlm.nih.gov/gene/?term=121456) | 1.7 | 0.00463 | 212.1 | 113.0 |
| CAPG | capping actin protein, gelsolin like | [822](https://www.ncbi.nlm.nih.gov/gene/?term=822) | 1.7 | 0.00099 | 1231.5 | 677.3 |
| SHPRH | SNF2 histone linker PHD RING helicase | [257218](https://www.ncbi.nlm.nih.gov/gene/?term=257218) | -1.7 | 0.00000 | 1103.1 | 1925.3 |
| TPRG1L | tumor protein p63 regulated 1-like | [127262](https://www.ncbi.nlm.nih.gov/gene/?term=127262) | -1.7 | 0.00265 | 2599.9 | 4804.9 |
| MRPL28 | mitochondrial ribosomal protein L28 | [10573](https://www.ncbi.nlm.nih.gov/gene/?term=10573) | 1.7 | 0.00000 | 674.9 | 387.1 |
| CNP | 2',3'-cyclic nucleotide 3' phosphodiesterase | [1267](https://www.ncbi.nlm.nih.gov/gene/?term=1267) | 1.7 | 0.00003 | 616.4 | 347.2 |
| SLC48A1 | solute carrier family 48 member 1 | [55652](https://www.ncbi.nlm.nih.gov/gene/?term=55652) | -1.7 | 0.01282 | 192.9 | 376.2 |
| C6orf163 | chromosome 6 open reading frame 163 | [206412](https://www.ncbi.nlm.nih.gov/gene/?term=206412) | -1.7 | 0.00047 | 176.1 | 317.3 |
| VNN2 | vanin 2 | [8875](https://www.ncbi.nlm.nih.gov/gene/?term=8875) | 1.7 | 0.01353 | 28161.4 | 14455.9 |
| AMPD3 | adenosine monophosphate deaminase 3 | [272](https://www.ncbi.nlm.nih.gov/gene/?term=272) | -1.7 | 0.00015 | 725.1 | 1297.1 |
| LOC107986918 |  | [107986918](https://www.ncbi.nlm.nih.gov/gene/?term=107986918) | -1.7 | 0.00761 | 22.1 | 42.8 |
| C1QTNF3 | C1q and tumor necrosis factor related protein 3 | [114899](https://www.ncbi.nlm.nih.gov/gene/?term=114899) | -1.7 | 0.00297 | 17.7 | 33.7 |
| CUBN | cubilin | [8029](https://www.ncbi.nlm.nih.gov/gene/?term=8029) | -1.7 | 0.00274 | 138.5 | 256.7 |
| CBL | Cbl proto-oncogene | [867](https://www.ncbi.nlm.nih.gov/gene/?term=867) | -1.7 | 0.00873 | 1833.6 | 3505.3 |
| TENM4 | teneurin transmembrane protein 4 | [26011](https://www.ncbi.nlm.nih.gov/gene/?term=26011) | -1.7 | 0.03304 | 3.0 | 14.5 |
| ZKSCAN8 | zinc finger with KRAB and SCAN domains 8 | [7745](https://www.ncbi.nlm.nih.gov/gene/?term=7745) | -1.7 | 0.00001 | 887.4 | 1560.8 |
| FLJ21408 | uncharacterized LOC400512 | [400512](https://www.ncbi.nlm.nih.gov/gene/?term=400512) | 1.7 | 0.04139 | 25.0 | 11.2 |
| SEC16A | SEC16 homolog A, endoplasmic reticulum export factor | [9919](https://www.ncbi.nlm.nih.gov/gene/?term=9919) | -1.7 | 0.00808 | 575.5 | 1091.4 |
| SYNE2 | spectrin repeat containing nuclear envelope protein 2 | [23224](https://www.ncbi.nlm.nih.gov/gene/?term=23224) | -1.7 | 0.00331 | 6053.9 | 11069.1 |
| LAMTOR2 | late endosomal/lysosomal adaptor, MAPK and MTOR activator 2 | [28956](https://www.ncbi.nlm.nih.gov/gene/?term=28956) | 1.7 | 0.00000 | 532.7 | 303.3 |
| MYO7A | myosin VIIA | [4647](https://www.ncbi.nlm.nih.gov/gene/?term=4647) | 1.7 | 0.01641 | 39.7 | 20.2 |
| FAM151B | family with sequence similarity 151 member B | [167555](https://www.ncbi.nlm.nih.gov/gene/?term=167555) | 1.7 | 0.00083 | 130.8 | 72.6 |
| FTH1P8 | ferritin, heavy polypeptide 1 pseudogene 8 | [2501](https://www.ncbi.nlm.nih.gov/gene/?term=2501) | -1.7 | 0.02220 | 10.0 | 20.5 |
| LOC105376059 |  | [105376059](https://www.ncbi.nlm.nih.gov/gene/?term=105376059) | -1.7 | 0.00004 | 79.6 | 142.8 |
| GNAO1 | G protein subunit alpha o1 | [2775](https://www.ncbi.nlm.nih.gov/gene/?term=2775) | -1.7 | 0.00866 | 20.3 | 38.2 |
| LOC102724470 |  | [102724470](https://www.ncbi.nlm.nih.gov/gene/?term=102724470) | 1.7 | 0.03981 | 19.8 | 8.9 |
| IL2RB | interleukin 2 receptor subunit beta | [3560](https://www.ncbi.nlm.nih.gov/gene/?term=3560) | -1.7 | 0.03224 | 218.3 | 470.6 |
| ZNF529 | zinc finger protein 529 | [57711](https://www.ncbi.nlm.nih.gov/gene/?term=57711) | -1.7 | 0.00000 | 298.2 | 523.4 |
| ARHGAP30 | Rho GTPase activating protein 30 | [257106](https://www.ncbi.nlm.nih.gov/gene/?term=257106) | 1.7 | 0.00000 | 12257.3 | 7032.1 |
| PIP5K1B | phosphatidylinositol-4-phosphate 5-kinase type 1 beta | [8395](https://www.ncbi.nlm.nih.gov/gene/?term=8395) | -1.7 | 0.01138 | 1539.0 | 2965.5 |
| CTH | cystathionine gamma-lyase | [1491](https://www.ncbi.nlm.nih.gov/gene/?term=1491) | -1.7 | 0.01544 | 75.8 | 147.8 |
| NRXN1 | neurexin 1 | [9378](https://www.ncbi.nlm.nih.gov/gene/?term=9378) | -1.7 | 0.01258 | 11.8 | 23.2 |
| LOC105369151 |  | [105369151](https://www.ncbi.nlm.nih.gov/gene/?term=105369151) | -1.7 | 0.01174 | 41.4 | 81.3 |
| LOC107983984 |  | [107983984](https://www.ncbi.nlm.nih.gov/gene/?term=107983984) | -1.7 | 0.00598 | 27.4 | 52.9 |
| YIPF1 | Yip1 domain family member 1 | [54432](https://www.ncbi.nlm.nih.gov/gene/?term=54432) | 1.7 | 0.00022 | 1000.5 | 561.4 |
| SEC14L1 | SEC14 like lipid binding 1 | [6397](https://www.ncbi.nlm.nih.gov/gene/?term=6397) | -1.7 | 0.00247 | 8200.1 | 14888.8 |
| LOC107985900 |  | [107985900](https://www.ncbi.nlm.nih.gov/gene/?term=107985900) | -1.7 | 0.01726 | 21.6 | 43.2 |
| USF3 | upstream transcription factor family member 3 | [205717](https://www.ncbi.nlm.nih.gov/gene/?term=205717) | -1.7 | 0.00018 | 2199.2 | 3940.1 |
| EBPL | emopamil binding protein like | [84650](https://www.ncbi.nlm.nih.gov/gene/?term=84650) | 1.7 | 0.00022 | 228.0 | 127.9 |
| KCTD1 | potassium channel tetramerization domain containing 1 | [284252](https://www.ncbi.nlm.nih.gov/gene/?term=284252) | 1.7 | 0.00144 | 42.8 | 24.0 |
| BRPF3 | bromodomain and PHD finger containing 3 | [27154](https://www.ncbi.nlm.nih.gov/gene/?term=27154) | -1.7 | 0.00064 | 270.9 | 488.4 |
| UBALD1 | UBA like domain containing 1 | [124402](https://www.ncbi.nlm.nih.gov/gene/?term=124402) | -1.7 | 0.00687 | 111.4 | 210.7 |
| CTRL | chymotrypsin like | [1506](https://www.ncbi.nlm.nih.gov/gene/?term=1506) | 1.7 | 0.00360 | 85.3 | 46.2 |
| CDK5RAP3 | CDK5 regulatory subunit associated protein 3 | [80279](https://www.ncbi.nlm.nih.gov/gene/?term=80279) | 1.7 | 0.00006 | 2487.6 | 1406.5 |
| FADD | Fas associated via death domain | [8772](https://www.ncbi.nlm.nih.gov/gene/?term=8772) | 1.7 | 0.00013 | 890.6 | 501.3 |
| ZNF404 | zinc finger protein 404 | [342908](https://www.ncbi.nlm.nih.gov/gene/?term=342908) | -1.7 | 0.00573 | 25.0 | 46.9 |
| LOC105378909 |  | [105378909](https://www.ncbi.nlm.nih.gov/gene/?term=105378909) | -1.7 | 0.01694 | 77.8 | 153.0 |
| SF3A1 | splicing factor 3a subunit 1 | [10291](https://www.ncbi.nlm.nih.gov/gene/?term=10291) | -1.7 | 0.00083 | 853.9 | 1542.9 |
| LOC105374897 |  | [105374897](https://www.ncbi.nlm.nih.gov/gene/?term=105374897) | -1.7 | 0.02935 | 31.2 | 66.2 |
| LYVE1 | lymphatic vessel endothelial hyaluronan receptor 1 | [10894](https://www.ncbi.nlm.nih.gov/gene/?term=10894) | 1.7 | 0.02359 | 24.5 | 12.4 |
| SCARB2 | scavenger receptor class B, member 2 | [950](https://www.ncbi.nlm.nih.gov/gene/?term=950) | 1.7 | 0.00014 | 1798.1 | 1013.2 |
| BTLA | B and T lymphocyte associated | [151888](https://www.ncbi.nlm.nih.gov/gene/?term=151888) | -1.7 | 0.00011 | 1048.7 | 1858.7 |
| PUM2 | pumilio RNA binding family member 2 | [23369](https://www.ncbi.nlm.nih.gov/gene/?term=23369) | -1.7 | 0.00000 | 5560.2 | 9699.8 |
| PPP1CB | protein phosphatase 1 catalytic subunit beta | [5500](https://www.ncbi.nlm.nih.gov/gene/?term=5500) | -1.7 | 0.00417 | 19164.1 | 35550.1 |
| ADPGK-AS1 | ADPGK antisense RNA 1 | [100287559](https://www.ncbi.nlm.nih.gov/gene/?term=100287559) | 1.7 | 0.01479 | 12.9 | 6.8 |
| KPNA1 | karyopherin subunit alpha 1 | [3836](https://www.ncbi.nlm.nih.gov/gene/?term=3836) | -1.7 | 0.00156 | 3258.8 | 5911.4 |
| MAK | male germ cell associated kinase | [4117](https://www.ncbi.nlm.nih.gov/gene/?term=4117) | 1.7 | 0.00828 | 557.0 | 295.1 |
| GAPDHP43 | glyceraldehyde 3 phosphate dehydrogenase pseudogene 43 | [100240708](https://www.ncbi.nlm.nih.gov/gene/?term=100240708) | -1.7 | 0.02400 | 15.1 | 31.5 |
| SLC30A4 | solute carrier family 30 member 4 | [7782](https://www.ncbi.nlm.nih.gov/gene/?term=7782) | -1.7 | 0.00003 | 150.6 | 265.3 |
| TSPAN18 | tetraspanin 18 | [90139](https://www.ncbi.nlm.nih.gov/gene/?term=90139) | -1.7 | 0.00781 | 53.5 | 101.4 |
| PAK3 | p21 (RAC1) activated kinase 3 | [5063](https://www.ncbi.nlm.nih.gov/gene/?term=5063) | -1.7 | 0.02529 | 6.5 | 13.9 |
| MCOLN1 | mucolipin 1 | [57192](https://www.ncbi.nlm.nih.gov/gene/?term=57192) | -1.7 | 0.01913 | 858.7 | 1698.5 |
| TRAJ10 | T cell receptor alpha joining 10 | [28745](https://www.ncbi.nlm.nih.gov/gene/?term=28745) | -1.7 | 0.03592 | 12.7 | 28.4 |
| LOC105369205 |  | [105369205](https://www.ncbi.nlm.nih.gov/gene/?term=105369205) | -1.7 | 0.04771 | 3.6 | 9.0 |
| DAPP1 | dual adaptor of phosphotyrosine and 3-phosphoinositides | [27071](https://www.ncbi.nlm.nih.gov/gene/?term=27071) | 1.7 | 0.00029 | 7954.2 | 4494.2 |
| TUBA1A | tubulin alpha 1a | [7846](https://www.ncbi.nlm.nih.gov/gene/?term=7846) | 1.7 | 0.00341 | 7062.1 | 3835.5 |
| ZNF524 | zinc finger protein 524 | [147807](https://www.ncbi.nlm.nih.gov/gene/?term=147807) | 1.7 | 0.00002 | 245.2 | 139.3 |
| SQSTM1 | sequestosome 1 | [8878](https://www.ncbi.nlm.nih.gov/gene/?term=8878) | 1.7 | 0.00783 | 25.7 | 13.3 |
| RFFL | ring finger and FYVE-like domain containing E3 ubiquitin protein ligase | [117584](https://www.ncbi.nlm.nih.gov/gene/?term=117584) | -1.7 | 0.00007 | 195.8 | 342.1 |
| RNASEK | ribonuclease K | [440400](https://www.ncbi.nlm.nih.gov/gene/?term=440400) | 1.7 | 0.01312 | 12.3 | 6.8 |
| RILPL1 | Rab interacting lysosomal protein-like 1 | [353116](https://www.ncbi.nlm.nih.gov/gene/?term=353116) | 1.7 | 0.00597 | 57.4 | 31.4 |
| ZBTB17 | zinc finger and BTB domain containing 17 | [7709](https://www.ncbi.nlm.nih.gov/gene/?term=7709) | 1.7 | 0.00000 | 629.8 | 364.0 |
| FABP5 | fatty acid binding protein 5 | [2171](https://www.ncbi.nlm.nih.gov/gene/?term=2171) | 1.7 | 0.00754 | 82.9 | 44.3 |
| TPMT | thiopurine S-methyltransferase | [7172](https://www.ncbi.nlm.nih.gov/gene/?term=7172) | 1.7 | 0.00001 | 585.3 | 334.8 |
| BLCAP | bladder cancer associated protein | [10904](https://www.ncbi.nlm.nih.gov/gene/?term=10904) | -1.7 | 0.00235 | 381.0 | 690.9 |
| SNORA49 | small nucleolar RNA, H/ACA box 49 | [677829](https://www.ncbi.nlm.nih.gov/gene/?term=677829) | 1.7 | 0.00208 | 58.9 | 32.1 |
| LOC728084 | uncharacterized LOC728084 | [728084](https://www.ncbi.nlm.nih.gov/gene/?term=728084) | -1.7 | 0.01837 | 20.4 | 39.7 |
| SPTLC2 | serine palmitoyltransferase long chain base subunit 2 | [9517](https://www.ncbi.nlm.nih.gov/gene/?term=9517) | 1.7 | 0.00025 | 8230.7 | 4655.5 |
| LOC105372278 |  | [105372278](https://www.ncbi.nlm.nih.gov/gene/?term=105372278) | 1.7 | 0.04654 | 9.7 | 4.3 |
| RNPEPL1 | arginyl aminopeptidase (aminopeptidase B)-like 1 | [57140](https://www.ncbi.nlm.nih.gov/gene/?term=57140) | -1.7 | 0.00179 | 563.8 | 1021.8 |
| LOC105371388 |  | [105371388](https://www.ncbi.nlm.nih.gov/gene/?term=105371388) | -1.7 | 0.02212 | 7.9 | 15.1 |
| LOC100128288 | uncharacterized LOC100128288 | [100128288](https://www.ncbi.nlm.nih.gov/gene/?term=100128288) | -1.7 | 0.04950 | 5.0 | 13.2 |
| C5orf63 | chromosome 5 open reading frame 63 | [401207](https://www.ncbi.nlm.nih.gov/gene/?term=401207) | -1.7 | 0.00103 | 78.1 | 140.3 |
| MYBPHL | myosin binding protein H-like | [343263](https://www.ncbi.nlm.nih.gov/gene/?term=343263) | 1.7 | 0.03417 | 13.6 | 6.4 |
| EEF2K | eukaryotic elongation factor 2 kinase | [29904](https://www.ncbi.nlm.nih.gov/gene/?term=29904) | -1.7 | 0.00231 | 186.3 | 337.7 |
| CCR3 | C-C motif chemokine receptor 3 | [1232](https://www.ncbi.nlm.nih.gov/gene/?term=1232) | -1.7 | 0.01180 | 337.9 | 644.2 |
| LOC105377359 |  | [105377359](https://www.ncbi.nlm.nih.gov/gene/?term=105377359) | -1.7 | 0.01790 | 15.2 | 30.5 |
| DHRS13 | dehydrogenase/reductase (SDR family) member 13 | [147015](https://www.ncbi.nlm.nih.gov/gene/?term=147015) | 1.7 | 0.00675 | 1446.8 | 780.1 |
| SLC25A24 | solute carrier family 25 member 24 | [29957](https://www.ncbi.nlm.nih.gov/gene/?term=29957) | 1.7 | 0.00010 | 1432.2 | 814.7 |
| SIL1 | SIL1 nucleotide exchange factor | [64374](https://www.ncbi.nlm.nih.gov/gene/?term=64374) | 1.7 | 0.00000 | 372.1 | 214.2 |
| TTC9 | tetratricopeptide repeat domain 9 | [23508](https://www.ncbi.nlm.nih.gov/gene/?term=23508) | -1.7 | 0.00275 | 160.9 | 293.3 |
| SLC2A3P2 | solute carrier family 2 member 3 pseudogene 2 | [391045](https://www.ncbi.nlm.nih.gov/gene/?term=391045) | -1.7 | 0.03151 | 46.6 | 97.9 |
| RRS1-AS1 | RRS1 antisense RNA 1 (head to head) | [100505676](https://www.ncbi.nlm.nih.gov/gene/?term=100505676) | -1.7 | 0.04069 | 6.5 | 15.3 |
| ATF7IP | activating transcription factor 7 interacting protein | [55729](https://www.ncbi.nlm.nih.gov/gene/?term=55729) | -1.7 | 0.00019 | 2842.0 | 5034.9 |
| LOC101928114 |  | [101928114](https://www.ncbi.nlm.nih.gov/gene/?term=101928114) | -1.7 | 0.04949 | 4.0 | 9.8 |
| TRNM |  | [4569](https://www.ncbi.nlm.nih.gov/gene/?term=4569) | -1.7 | 0.00745 | 190.7 | 353.9 |
| HIST1H4J | histone cluster 1, H4j | [8363](https://www.ncbi.nlm.nih.gov/gene/?term=8363) | 1.7 | 0.00308 | 78.7 | 43.1 |
| HLA-B | major histocompatibility complex, class I, B | [3106](https://www.ncbi.nlm.nih.gov/gene/?term=3106) | 1.7 | 0.00478 | 41871.4 | 22788.3 |
| CYFIP1 | cytoplasmic FMR1 interacting protein 1 | [23191](https://www.ncbi.nlm.nih.gov/gene/?term=23191) | 1.7 | 0.00046 | 714.7 | 402.5 |
| AP5B1 | adaptor related protein complex 5 beta 1 subunit | [91056](https://www.ncbi.nlm.nih.gov/gene/?term=91056) | 1.7 | 0.00437 | 2035.9 | 1115.8 |
| SNX29 | sorting nexin 29 | [92017](https://www.ncbi.nlm.nih.gov/gene/?term=92017) | -1.7 | 0.00160 | 1922.0 | 3462.5 |
| MKNK1 | MAP kinase interacting serine/threonine kinase 1 | [8569](https://www.ncbi.nlm.nih.gov/gene/?term=8569) | 1.7 | 0.00069 | 2036.6 | 1143.7 |
| MKLN1-AS | MKLN1 antisense RNA | [100506881](https://www.ncbi.nlm.nih.gov/gene/?term=100506881) | -1.7 | 0.00868 | 40.6 | 75.0 |
| SRRM2 | serine/arginine repetitive matrix 2 | [23524](https://www.ncbi.nlm.nih.gov/gene/?term=23524) | -1.7 | 0.01705 | 2317.5 | 4494.9 |
| GPHN | gephyrin | [10243](https://www.ncbi.nlm.nih.gov/gene/?term=10243) | -1.7 | 0.00127 | 50.7 | 91.9 |
| ANKRD35 | ankyrin repeat domain 35 | [148741](https://www.ncbi.nlm.nih.gov/gene/?term=148741) | 1.7 | 0.05573 | 7.7 | 3.0 |
| ZNF891 | zinc finger protein 891 | [101060200](https://www.ncbi.nlm.nih.gov/gene/?term=101060200) | -1.7 | 0.00122 | 79.9 | 143.5 |
| LOC107986465 |  | [107986465](https://www.ncbi.nlm.nih.gov/gene/?term=107986465) | -1.7 | 0.02670 | 863.6 | 1739.5 |
| TAB3 | TGF-beta activated kinase 1/MAP3K7 binding protein 3 | [257397](https://www.ncbi.nlm.nih.gov/gene/?term=257397) | -1.7 | 0.01019 | 2646.1 | 4994.1 |
| ME3 | malic enzyme 3 | [10873](https://www.ncbi.nlm.nih.gov/gene/?term=10873) | 1.7 | 0.00606 | 42.0 | 22.6 |
| ZNF398 | zinc finger protein 398 | [57541](https://www.ncbi.nlm.nih.gov/gene/?term=57541) | -1.7 | 0.00002 | 346.5 | 605.1 |
| ZNF138 | zinc finger protein 138 | [7697](https://www.ncbi.nlm.nih.gov/gene/?term=7697) | -1.7 | 0.00000 | 234.8 | 405.9 |
| GLA | galactosidase alpha | [2717](https://www.ncbi.nlm.nih.gov/gene/?term=2717) | 1.7 | 0.00012 | 752.9 | 428.5 |
| STMN1 | stathmin 1 | [3925](https://www.ncbi.nlm.nih.gov/gene/?term=3925) | 1.7 | 0.00697 | 886.9 | 479.1 |
| JMY | junction mediating and regulatory protein, p53 cofactor | [133746](https://www.ncbi.nlm.nih.gov/gene/?term=133746) | -1.7 | 0.00018 | 486.7 | 855.6 |
| C10orf12 | chromosome 10 open reading frame 12 | [26148](https://www.ncbi.nlm.nih.gov/gene/?term=26148) | -1.7 | 0.00003 | 815.1 | 1417.4 |
| ZNF433 | zinc finger protein 433 | [163059](https://www.ncbi.nlm.nih.gov/gene/?term=163059) | -1.7 | 0.00341 | 13.2 | 24.5 |
| ITIH1 | inter-alpha-trypsin inhibitor heavy chain 1 | [3697](https://www.ncbi.nlm.nih.gov/gene/?term=3697) | 1.7 | 0.04601 | 15.0 | 6.6 |
| FCHSD2 | FCH and double SH3 domains 2 | [9873](https://www.ncbi.nlm.nih.gov/gene/?term=9873) | -1.7 | 0.00055 | 664.2 | 1176.9 |
| ANK1 | ankyrin 1 | [286](https://www.ncbi.nlm.nih.gov/gene/?term=286) | -1.7 | 0.02762 | 8648.8 | 17895.5 |
| ZNF800 | zinc finger protein 800 | [168850](https://www.ncbi.nlm.nih.gov/gene/?term=168850) | -1.7 | 0.00004 | 1847.3 | 3212.8 |
| PACS1 | phosphofurin acidic cluster sorting protein 1 | [55690](https://www.ncbi.nlm.nih.gov/gene/?term=55690) | -1.7 | 0.00303 | 947.5 | 1718.5 |
| LOC105375713 | uncharacterized LOC105375713 | [105375713](https://www.ncbi.nlm.nih.gov/gene/?term=105375713) | 1.7 | 0.00626 | 108.1 | 59.0 |
| PTGES3 | prostaglandin E synthase 3 | [10728](https://www.ncbi.nlm.nih.gov/gene/?term=10728) | -1.7 | 0.00121 | 2572.5 | 4590.8 |
| LOC107986871 |  | [107986871](https://www.ncbi.nlm.nih.gov/gene/?term=107986871) | -1.7 | 0.00661 | 45.5 | 84.6 |
| EPM2A | epilepsy, progressive myoclonus type 2A, Lafora disease (laforin) | [7957](https://www.ncbi.nlm.nih.gov/gene/?term=7957) | -1.7 | 0.01184 | 190.3 | 362.4 |
| BTG1 | B-cell translocation gene 1, anti-proliferative | [694](https://www.ncbi.nlm.nih.gov/gene/?term=694) | -1.7 | 0.00004 | 2965.9 | 5156.5 |
| SMOX | spermine oxidase | [54498](https://www.ncbi.nlm.nih.gov/gene/?term=54498) | -1.7 | 0.02216 | 1312.3 | 2631.0 |
| MFSD14B | major facilitator superfamily domain containing 14B | [84641](https://www.ncbi.nlm.nih.gov/gene/?term=84641) | 1.7 | 0.00210 | 3460.8 | 1923.2 |
| CDK5 | cyclin-dependent kinase 5 | [1020](https://www.ncbi.nlm.nih.gov/gene/?term=1020) | 1.7 | 0.00004 | 182.4 | 104.4 |
| MERTK | MER proto-oncogene, tyrosine kinase | [10461](https://www.ncbi.nlm.nih.gov/gene/?term=10461) | 1.7 | 0.01019 | 147.9 | 79.2 |
| SLC7A7 | solute carrier family 7 member 7 | [9056](https://www.ncbi.nlm.nih.gov/gene/?term=9056) | 1.7 | 0.00019 | 2557.9 | 1460.1 |
| LOC101928227 | uncharacterized LOC101928227 | [101928227](https://www.ncbi.nlm.nih.gov/gene/?term=101928227) | -1.7 | 0.04416 | 2.9 | 7.1 |
| HIST1H3C | histone cluster 1, H3c | [8352](https://www.ncbi.nlm.nih.gov/gene/?term=8352) | 1.7 | 0.03226 | 1042.8 | 507.5 |
| SKAP1 | src kinase associated phosphoprotein 1 | [8631](https://www.ncbi.nlm.nih.gov/gene/?term=8631) | -1.7 | 0.00657 | 228.6 | 423.1 |
| ZNF10 | zinc finger protein 10 | [7556](https://www.ncbi.nlm.nih.gov/gene/?term=7556) | -1.7 | 0.00012 | 97.6 | 170.8 |
| RNASEL | ribonuclease L | [6041](https://www.ncbi.nlm.nih.gov/gene/?term=6041) | 1.7 | 0.00020 | 2581.7 | 1473.7 |
| LOC101928465 |  | [101928465](https://www.ncbi.nlm.nih.gov/gene/?term=101928465) | 1.7 | 0.00001 | 346.3 | 200.1 |
| PRDM11 | PR domain 11 | [56981](https://www.ncbi.nlm.nih.gov/gene/?term=56981) | -1.7 | 0.00413 | 36.2 | 66.6 |
| WASL | Wiskott-Aldrich syndrome-like | [8976](https://www.ncbi.nlm.nih.gov/gene/?term=8976) | -1.7 | 0.00061 | 213.8 | 379.8 |
| MSANTD2 | Myb/SANT DNA binding domain containing 2 | [79684](https://www.ncbi.nlm.nih.gov/gene/?term=79684) | -1.7 | 0.00258 | 273.4 | 494.0 |
| ZNF385D | zinc finger protein 385D | [79750](https://www.ncbi.nlm.nih.gov/gene/?term=79750) | -1.7 | 0.04603 | 9.3 | 21.3 |
| HIST1H1A | histone cluster 1, H1a | [3024](https://www.ncbi.nlm.nih.gov/gene/?term=3024) | 1.7 | 0.05069 | 13.1 | 5.7 |
| CCR5 | C-C motif chemokine receptor 5 (gene/pseudogene) | [1234](https://www.ncbi.nlm.nih.gov/gene/?term=1234) | 1.7 | 0.00817 | 748.9 | 403.9 |
| PITPNB | phosphatidylinositol transfer protein beta | [23760](https://www.ncbi.nlm.nih.gov/gene/?term=23760) | -1.7 | 0.00001 | 352.7 | 611.7 |
| PLEKHJ1 | pleckstrin homology domain containing J1 | [55111](https://www.ncbi.nlm.nih.gov/gene/?term=55111) | 1.7 | 0.00002 | 594.4 | 343.3 |
| LTBR | lymphotoxin beta receptor | [4055](https://www.ncbi.nlm.nih.gov/gene/?term=4055) | 1.7 | 0.00374 | 495.3 | 274.0 |
| ABHD17A | abhydrolase domain containing 17A | [81926](https://www.ncbi.nlm.nih.gov/gene/?term=81926) | 1.7 | 0.00024 | 2653.5 | 1514.7 |
| BACE1-AS | BACE1 antisense RNA | [100379571](https://www.ncbi.nlm.nih.gov/gene/?term=100379571) | -1.7 | 0.03829 | 7.1 | 15.2 |
| AKAP6 | A-kinase anchoring protein 6 | [9472](https://www.ncbi.nlm.nih.gov/gene/?term=9472) | -1.7 | 0.01584 | 26.4 | 51.2 |
| LOC105374333 |  | [105374333](https://www.ncbi.nlm.nih.gov/gene/?term=105374333) | -1.7 | 0.02783 | 28.3 | 58.1 |
| HSPB1 | heat shock protein family B (small) member 1 | [3315](https://www.ncbi.nlm.nih.gov/gene/?term=3315) | 1.7 | 0.03834 | 68.2 | 32.2 |
| SOWAHD | sosondowah ankyrin repeat domain family member D | [347454](https://www.ncbi.nlm.nih.gov/gene/?term=347454) | 1.7 | 0.00014 | 246.3 | 141.2 |
| RMST | rhabdomyosarcoma 2 associated transcript (non-protein coding) | [196475](https://www.ncbi.nlm.nih.gov/gene/?term=196475) | -1.7 | 0.01150 | 19.3 | 35.4 |
| HTATIP2 | HIV-1 Tat interactive protein 2 | [10553](https://www.ncbi.nlm.nih.gov/gene/?term=10553) | 1.7 | 0.00054 | 1568.3 | 890.9 |
| LOC105369440 |  | [105369440](https://www.ncbi.nlm.nih.gov/gene/?term=105369440) | -1.7 | 0.04563 | 4.0 | 9.7 |
| PGK1 | phosphoglycerate kinase 1 | [5230](https://www.ncbi.nlm.nih.gov/gene/?term=5230) | 1.7 | 0.00016 | 12257.2 | 7056.8 |
| LOC107984035 |  | [107984035](https://www.ncbi.nlm.nih.gov/gene/?term=107984035) | -1.7 | 0.02241 | 15.1 | 29.7 |
| CYB5R3 | cytochrome b5 reductase 3 | [1727](https://www.ncbi.nlm.nih.gov/gene/?term=1727) | -1.7 | 0.00665 | 545.1 | 999.6 |
| STXBP4 | syntaxin binding protein 4 | [252983](https://www.ncbi.nlm.nih.gov/gene/?term=252983) | -1.7 | 0.00012 | 179.9 | 311.2 |
| NINL | ninein like | [22981](https://www.ncbi.nlm.nih.gov/gene/?term=22981) | 1.7 | 0.01814 | 38.5 | 20.1 |
| MCTP2 | multiple C2 domains, transmembrane 2 | [55784](https://www.ncbi.nlm.nih.gov/gene/?term=55784) | 1.7 | 0.00659 | 6207.0 | 3392.7 |
| LOC399715 | uncharacterized LOC399715 | [399715](https://www.ncbi.nlm.nih.gov/gene/?term=399715) | 1.7 | 0.03872 | 104.0 | 49.5 |
| TIPARP | TCDD inducible poly(ADP-ribose) polymerase | [25976](https://www.ncbi.nlm.nih.gov/gene/?term=25976) | 1.7 | 0.00061 | 1990.9 | 1130.1 |
| MKRN5P | makorin ring finger protein 5, pseudogene | [7683](https://www.ncbi.nlm.nih.gov/gene/?term=7683) | -1.7 | 0.03065 | 13.5 | 27.5 |
| ZNF462 | zinc finger protein 462 | [58499](https://www.ncbi.nlm.nih.gov/gene/?term=58499) | -1.7 | 0.03663 | 13.2 | 28.3 |
| ALPL | alkaline phosphatase, liver/bone/kidney | [249](https://www.ncbi.nlm.nih.gov/gene/?term=249) | 1.7 | 0.03268 | 2658.9 | 1303.6 |
| CCDC154 | coiled-coil domain containing 154 | [645811](https://www.ncbi.nlm.nih.gov/gene/?term=645811) | 1.7 | 0.01764 | 29.4 | 15.8 |
| CHIC1 | cysteine rich hydrophobic domain 1 | [53344](https://www.ncbi.nlm.nih.gov/gene/?term=53344) | -1.7 | 0.00021 | 333.8 | 584.1 |
| ESRRA | estrogen related receptor alpha | [2101](https://www.ncbi.nlm.nih.gov/gene/?term=2101) | 1.7 | 0.00001 | 931.1 | 541.9 |
| LOC101928377 |  | [101928377](https://www.ncbi.nlm.nih.gov/gene/?term=101928377) | 1.7 | 0.01149 | 18.2 | 9.7 |
| GSPT1 | G1 to S phase transition 1 | [2935](https://www.ncbi.nlm.nih.gov/gene/?term=2935) | -1.7 | 0.02893 | 68541.6 | 143049.9 |
| LOC644285 | uncharacterized LOC644285 | [644285](https://www.ncbi.nlm.nih.gov/gene/?term=644285) | -1.7 | 0.03531 | 47.9 | 101.0 |
| APPL2 | adaptor protein, phosphotyrosine interacting with PH domain and leucine zipper 2 | [55198](https://www.ncbi.nlm.nih.gov/gene/?term=55198) | -1.7 | 0.00008 | 857.3 | 1488.7 |
| ABTB1 | ankyrin repeat and BTB domain containing 1 | [80325](https://www.ncbi.nlm.nih.gov/gene/?term=80325) | -1.7 | 0.00103 | 2716.8 | 4766.5 |
| IDO1 | indoleamine 2,3-dioxygenase 1 | [3620](https://www.ncbi.nlm.nih.gov/gene/?term=3620) | 1.7 | 0.05178 | 500.4 | 209.7 |
| TBC1D17 | TBC1 domain family member 17 | [79735](https://www.ncbi.nlm.nih.gov/gene/?term=79735) | -1.7 | 0.00485 | 312.5 | 566.1 |
| ZFP2 | ZFP2 zinc finger protein | [80108](https://www.ncbi.nlm.nih.gov/gene/?term=80108) | -1.7 | 0.00510 | 9.6 | 18.2 |
| PMF1 | polyamine-modulated factor 1 | [11243](https://www.ncbi.nlm.nih.gov/gene/?term=11243) | 1.7 | 0.00019 | 301.1 | 172.7 |
| CDK15 | cyclin-dependent kinase 15 | [65061](https://www.ncbi.nlm.nih.gov/gene/?term=65061) | -1.7 | 0.05289 | 5.4 | 14.8 |
| SNORA50A | small nucleolar RNA, H/ACA box 50A | [677830](https://www.ncbi.nlm.nih.gov/gene/?term=677830) | -1.7 | 0.00416 | 20.1 | 37.0 |
| KLRC2 | killer cell lectin like receptor C2 | [3822](https://www.ncbi.nlm.nih.gov/gene/?term=3822) | -1.7 | 0.02832 | 71.0 | 143.9 |
| SYP | synaptophysin | [6855](https://www.ncbi.nlm.nih.gov/gene/?term=6855) | 1.7 | 0.02015 | 12.0 | 6.3 |
| RHOT2 | ras homolog family member T2 | [89941](https://www.ncbi.nlm.nih.gov/gene/?term=89941) | 1.7 | 0.00044 | 807.4 | 460.2 |
| PLEKHA7 | pleckstrin homology domain containing A7 | [144100](https://www.ncbi.nlm.nih.gov/gene/?term=144100) | -1.7 | 0.03096 | 11.2 | 23.1 |
| C17orf58 | chromosome 17 open reading frame 58 | [284018](https://www.ncbi.nlm.nih.gov/gene/?term=284018) | 1.7 | 0.00240 | 37.3 | 20.9 |
| LOC105374985 |  | [105374985](https://www.ncbi.nlm.nih.gov/gene/?term=105374985) | 1.7 | 0.01105 | 361.1 | 193.8 |
| RN7SL128P | RNA, 7SL, cytoplasmic 128, pseudogene | [106481826](https://www.ncbi.nlm.nih.gov/gene/?term=106481826) | -1.7 | 0.02656 | 34.4 | 66.8 |
| AP5Z1 | adaptor related protein complex 5 zeta 1 subunit | [9907](https://www.ncbi.nlm.nih.gov/gene/?term=9907) | 1.7 | 0.00000 | 720.3 | 421.8 |
| FAM157B | family with sequence similarity 157 member B | [100132403](https://www.ncbi.nlm.nih.gov/gene/?term=100132403) | 1.7 | 0.02040 | 595.5 | 308.5 |
| IL1RL1 | interleukin 1 receptor like 1 | [9173](https://www.ncbi.nlm.nih.gov/gene/?term=9173) | -1.7 | 0.02951 | 76.2 | 154.0 |
| LOC105369170 |  | [105369170](https://www.ncbi.nlm.nih.gov/gene/?term=105369170) | -1.7 | 0.02503 | 233.5 | 461.9 |
| LOC107986673 |  | [107986673](https://www.ncbi.nlm.nih.gov/gene/?term=107986673) | -1.7 | 0.04921 | 4.3 | 12.4 |
| LOC105371620 |  | [105371620](https://www.ncbi.nlm.nih.gov/gene/?term=105371620) | 1.7 | 0.05596 | 8.8 | 3.3 |
| JAKMIP3 | Janus kinase and microtubule interacting protein 3 | [282973](https://www.ncbi.nlm.nih.gov/gene/?term=282973) | 1.7 | 0.03527 | 11.7 | 5.8 |
| PNKD | paroxysmal nonkinesigenic dyskinesia | [25953](https://www.ncbi.nlm.nih.gov/gene/?term=25953) | -1.7 | 0.00349 | 267.1 | 480.6 |
| LOC105372491 |  | [105372491](https://www.ncbi.nlm.nih.gov/gene/?term=105372491) | -1.7 | 0.01973 | 28.1 | 55.6 |
| SNX21 | sorting nexin family member 21 | [90203](https://www.ncbi.nlm.nih.gov/gene/?term=90203) | 1.7 | 0.00421 | 121.2 | 67.2 |
| MID1IP1 | MID1 interacting protein 1 | [58526](https://www.ncbi.nlm.nih.gov/gene/?term=58526) | 1.7 | 0.00025 | 1771.2 | 1021.0 |
| GPBP1L1 | GC-rich promoter binding protein 1 like 1 | [60313](https://www.ncbi.nlm.nih.gov/gene/?term=60313) | -1.7 | 0.00008 | 3730.6 | 6433.4 |
| MAP1LC3B2 | microtubule associated protein 1 light chain 3 beta 2 | [643246](https://www.ncbi.nlm.nih.gov/gene/?term=643246) | -1.7 | 0.01443 | 28.1 | 52.1 |
| ARHGAP4 | Rho GTPase activating protein 4 | [393](https://www.ncbi.nlm.nih.gov/gene/?term=393) | 1.7 | 0.00098 | 3164.4 | 1801.3 |
| GIMAP2 | GTPase, IMAP family member 2 | [26157](https://www.ncbi.nlm.nih.gov/gene/?term=26157) | 1.7 | 0.00004 | 3221.9 | 1873.0 |
| CDCA3 | cell division cycle associated 3 | [83461](https://www.ncbi.nlm.nih.gov/gene/?term=83461) | 1.7 | 0.01229 | 46.5 | 25.1 |
| PCBD1 | pterin-4 alpha-carbinolamine dehydratase 1 | [5092](https://www.ncbi.nlm.nih.gov/gene/?term=5092) | 1.7 | 0.00012 | 101.5 | 58.3 |
| ZFAND5 | zinc finger AN1-type containing 5 | [7763](https://www.ncbi.nlm.nih.gov/gene/?term=7763) | -1.7 | 0.00430 | 10303.9 | 18497.3 |
| MCL1 | myeloid cell leukemia 1 | [4170](https://www.ncbi.nlm.nih.gov/gene/?term=4170) | 1.7 | 0.00009 | 52724.6 | 30556.1 |
| SLC25A10 | solute carrier family 25 member 10 | [1468](https://www.ncbi.nlm.nih.gov/gene/?term=1468) | 1.7 | 0.01211 | 22.3 | 11.9 |
| SMG1P3 | SMG1 pseudogene 3 | [100271836](https://www.ncbi.nlm.nih.gov/gene/?term=100271836) | -1.7 | 0.00067 | 1369.1 | 2397.7 |
| MPPED2 | metallophosphoesterase domain containing 2 | [744](https://www.ncbi.nlm.nih.gov/gene/?term=744) | -1.7 | 0.04081 | 5.9 | 12.5 |
| MOB3B | MOB kinase activator 3B | [79817](https://www.ncbi.nlm.nih.gov/gene/?term=79817) | -1.7 | 0.00061 | 110.0 | 193.5 |
| UBE2Q1 | ubiquitin conjugating enzyme E2 Q1 | [55585](https://www.ncbi.nlm.nih.gov/gene/?term=55585) | 1.7 | 0.00000 | 1676.5 | 980.7 |
| NAGS | N-acetylglutamate synthase | [162417](https://www.ncbi.nlm.nih.gov/gene/?term=162417) | 1.7 | 0.00220 | 33.9 | 18.6 |
| B4GALT3 | beta-1,4-galactosyltransferase 3 | [8703](https://www.ncbi.nlm.nih.gov/gene/?term=8703) | -1.7 | 0.01515 | 878.5 | 1649.0 |
| PEX11G | peroxisomal biogenesis factor 11 gamma | [92960](https://www.ncbi.nlm.nih.gov/gene/?term=92960) | 1.7 | 0.04801 | 14.7 | 6.8 |
| PHF23 | PHD finger protein 23 | [79142](https://www.ncbi.nlm.nih.gov/gene/?term=79142) | 1.7 | 0.00001 | 1083.7 | 635.3 |
| SSPN | sarcospan | [8082](https://www.ncbi.nlm.nih.gov/gene/?term=8082) | -1.7 | 0.01399 | 34.8 | 66.0 |
| SNORD110 | small nucleolar RNA, C/D box 110 | [692213](https://www.ncbi.nlm.nih.gov/gene/?term=692213) | 1.7 | 0.02976 | 12.6 | 6.1 |
| BAX | BCL2-associated X protein | [581](https://www.ncbi.nlm.nih.gov/gene/?term=581) | 1.7 | 0.00000 | 404.5 | 239.7 |
| HIST1H4A | histone cluster 1, H4a | [8359](https://www.ncbi.nlm.nih.gov/gene/?term=8359) | 1.7 | 0.01895 | 276.0 | 144.4 |
| CPVL | carboxypeptidase, vitellogenic like | [54504](https://www.ncbi.nlm.nih.gov/gene/?term=54504) | 1.7 | 0.00401 | 4086.9 | 2268.2 |
| ATHL1 | ATH1, acid trehalase-like 1 (yeast) | [80162](https://www.ncbi.nlm.nih.gov/gene/?term=80162) | 1.7 | 0.00264 | 1670.2 | 935.9 |
| BPTF | bromodomain PHD finger transcription factor | [2186](https://www.ncbi.nlm.nih.gov/gene/?term=2186) | -1.7 | 0.00000 | 5019.3 | 8514.4 |
| PSMA6 | proteasome subunit alpha 6 | [5687](https://www.ncbi.nlm.nih.gov/gene/?term=5687) | 1.7 | 0.00001 | 2105.3 | 1236.7 |
| SLC15A2 | solute carrier family 15 member 2 | [6565](https://www.ncbi.nlm.nih.gov/gene/?term=6565) | -1.7 | 0.00496 | 268.1 | 483.9 |
| CAPZA2 | capping actin protein of muscle Z-line alpha subunit 2 | [830](https://www.ncbi.nlm.nih.gov/gene/?term=830) | 1.7 | 0.00031 | 8705.2 | 5039.8 |
| GORASP1 | golgi reassembly stacking protein 1 | [64689](https://www.ncbi.nlm.nih.gov/gene/?term=64689) | 1.7 | 0.00002 | 513.1 | 299.6 |
| APOBEC3G | apolipoprotein B mRNA editing enzyme catalytic subunit 3G | [60489](https://www.ncbi.nlm.nih.gov/gene/?term=60489) | 1.7 | 0.00040 | 1574.0 | 904.8 |
| OLIG2 | oligodendrocyte lineage transcription factor 2 | [10215](https://www.ncbi.nlm.nih.gov/gene/?term=10215) | -1.7 | 0.05052 | 23.5 | 55.8 |
| TTC25 | tetratricopeptide repeat domain 25 | [83538](https://www.ncbi.nlm.nih.gov/gene/?term=83538) | -1.7 | 0.03989 | 310.2 | 658.0 |
| FAM198B | family with sequence similarity 198 member B | [51313](https://www.ncbi.nlm.nih.gov/gene/?term=51313) | 1.7 | 0.00786 | 1573.0 | 864.8 |
| FOXO3B | forkhead box O3B pseudogene | [2310](https://www.ncbi.nlm.nih.gov/gene/?term=2310) | -1.7 | 0.00668 | 305.9 | 552.8 |
| PIGQ | phosphatidylinositol glycan anchor biosynthesis class Q | [9091](https://www.ncbi.nlm.nih.gov/gene/?term=9091) | -1.7 | 0.01024 | 470.3 | 866.3 |
| CDKN2B-AS1 | CDKN2B antisense RNA 1 | [100048912](https://www.ncbi.nlm.nih.gov/gene/?term=100048912) | -1.7 | 0.02101 | 11.3 | 22.8 |
| MRPL44 | mitochondrial ribosomal protein L44 | [65080](https://www.ncbi.nlm.nih.gov/gene/?term=65080) | 1.7 | 0.00000 | 924.9 | 544.6 |
| ZNF257 | zinc finger protein 257 | [113835](https://www.ncbi.nlm.nih.gov/gene/?term=113835) | -1.7 | 0.00417 | 75.4 | 136.3 |
| DCLK2 | doublecortin like kinase 2 | [166614](https://www.ncbi.nlm.nih.gov/gene/?term=166614) | -1.7 | 0.04508 | 7.5 | 16.6 |
| ATP6V0D1 | ATPase H+ transporting V0 subunit d1 | [9114](https://www.ncbi.nlm.nih.gov/gene/?term=9114) | 1.7 | 0.00009 | 4048.4 | 2354.3 |
| B2M | beta-2-microglobulin | [567](https://www.ncbi.nlm.nih.gov/gene/?term=567) | 1.7 | 0.00028 | 50831.6 | 29361.6 |
| CNOT4 | CCR4-NOT transcription complex subunit 4 | [4850](https://www.ncbi.nlm.nih.gov/gene/?term=4850) | -1.7 | 0.00005 | 407.4 | 698.2 |
| HSBP1 | heat shock factor binding protein 1 | [3281](https://www.ncbi.nlm.nih.gov/gene/?term=3281) | 1.7 | 0.00004 | 2038.4 | 1188.2 |
| UBAP1 | ubiquitin associated protein 1 | [51271](https://www.ncbi.nlm.nih.gov/gene/?term=51271) | -1.7 | 0.01092 | 2644.6 | 4885.5 |
| TPM3P7 | tropomyosin 3 pseudogene 7 | [751599](https://www.ncbi.nlm.nih.gov/gene/?term=751599) | -1.7 | 0.04744 | 5.7 | 13.4 |
| DHRS12 | dehydrogenase/reductase (SDR family) member 12 | [79758](https://www.ncbi.nlm.nih.gov/gene/?term=79758) | 1.7 | 0.00102 | 498.0 | 285.0 |
| POC1B | POC1 centriolar protein B | [282809](https://www.ncbi.nlm.nih.gov/gene/?term=282809) | -1.7 | 0.02174 | 4299.5 | 8306.1 |
| MIR548E | microRNA 548e | [100313921](https://www.ncbi.nlm.nih.gov/gene/?term=100313921) | -1.7 | 0.02767 | 12.7 | 25.7 |
| TLR9 | toll like receptor 9 | [54106](https://www.ncbi.nlm.nih.gov/gene/?term=54106) | -1.7 | 0.03905 | 12.7 | 27.4 |
| FLJ37201 | tigger transposable element derived 2 pseudogene | [283011](https://www.ncbi.nlm.nih.gov/gene/?term=283011) | -1.7 | 0.03437 | 12.9 | 27.4 |
| ANGPT1 | angiopoietin 1 | [284](https://www.ncbi.nlm.nih.gov/gene/?term=284) | -1.7 | 0.01325 | 166.8 | 309.3 |
| ZNF621 | zinc finger protein 621 | [285268](https://www.ncbi.nlm.nih.gov/gene/?term=285268) | -1.7 | 0.00096 | 221.0 | 388.2 |
| PET117 | PET117 homolog | [100303755](https://www.ncbi.nlm.nih.gov/gene/?term=100303755) | 1.7 | 0.01098 | 11.2 | 6.1 |
| EMB | embigin | [133418](https://www.ncbi.nlm.nih.gov/gene/?term=133418) | 1.7 | 0.00024 | 8584.5 | 4968.4 |
| LOC101928403 | uncharacterized LOC101928403 | [101928403](https://www.ncbi.nlm.nih.gov/gene/?term=101928403) | -1.7 | 0.00541 | 8.2 | 16.0 |
| PABPN1 | poly(A) binding protein nuclear 1 | [8106](https://www.ncbi.nlm.nih.gov/gene/?term=8106) | 1.7 | 0.00000 | 100.1 | 59.9 |
| DEGS1 | delta(4)-desaturase, sphingolipid 1 | [8560](https://www.ncbi.nlm.nih.gov/gene/?term=8560) | 1.7 | 0.00007 | 1863.9 | 1085.3 |
| CADM4 | cell adhesion molecule 4 | [199731](https://www.ncbi.nlm.nih.gov/gene/?term=199731) | 1.7 | 0.00463 | 67.2 | 37.4 |
| SCARNA18 | small Cajal body-specific RNA 18 | [677765](https://www.ncbi.nlm.nih.gov/gene/?term=677765) | -1.7 | 0.02298 | 5.7 | 11.7 |
| RGS14 | regulator of G-protein signaling 14 | [10636](https://www.ncbi.nlm.nih.gov/gene/?term=10636) | 1.7 | 0.00021 | 1431.0 | 830.2 |
| MAP9 | microtubule associated protein 9 | [79884](https://www.ncbi.nlm.nih.gov/gene/?term=79884) | -1.7 | 0.00058 | 112.3 | 195.7 |
| DEF6 | DEF6, guanine nucleotide exchange factor | [50619](https://www.ncbi.nlm.nih.gov/gene/?term=50619) | 1.7 | 0.00001 | 3372.8 | 1983.1 |
| ADAM19 | ADAM metallopeptidase domain 19 | [8728](https://www.ncbi.nlm.nih.gov/gene/?term=8728) | -1.7 | 0.00180 | 1144.0 | 2014.0 |
| EPB41L3 | erythrocyte membrane protein band 4.1 like 3 | [23136](https://www.ncbi.nlm.nih.gov/gene/?term=23136) | 1.7 | 0.00127 | 1122.0 | 640.6 |
| MMACHC | methylmalonic aciduria (cobalamin deficiency) cblC type, with homocystinuria | [25974](https://www.ncbi.nlm.nih.gov/gene/?term=25974) | -1.7 | 0.02580 | 12.6 | 25.9 |
| CD2BP2 | CD2 (cytoplasmic tail) binding protein 2 | [10421](https://www.ncbi.nlm.nih.gov/gene/?term=10421) | 1.7 | 0.00000 | 1474.6 | 875.7 |
| MYO6 | myosin VI | [4646](https://www.ncbi.nlm.nih.gov/gene/?term=4646) | -1.7 | 0.02744 | 20.5 | 41.4 |
| ENO1 | enolase 1 | [2023](https://www.ncbi.nlm.nih.gov/gene/?term=2023) | 1.7 | 0.00003 | 14039.9 | 8241.6 |
| GATS | GATS, stromal antigen 3 opposite strand | [352954](https://www.ncbi.nlm.nih.gov/gene/?term=352954) | -1.7 | 0.00081 | 110.5 | 193.7 |
| SPATA13 | spermatogenesis associated 13 | [221178](https://www.ncbi.nlm.nih.gov/gene/?term=221178) | 1.7 | 0.00007 | 5965.0 | 3488.3 |
| INO80E | INO80 complex subunit E | [283899](https://www.ncbi.nlm.nih.gov/gene/?term=283899) | 1.7 | 0.00540 | 435.5 | 242.1 |
| ATG2A | autophagy related 2A | [23130](https://www.ncbi.nlm.nih.gov/gene/?term=23130) | -1.7 | 0.00338 | 364.6 | 648.7 |
| R3HDM2 | R3H domain containing 2 | [22864](https://www.ncbi.nlm.nih.gov/gene/?term=22864) | -1.7 | 0.00185 | 398.5 | 704.6 |
| ICAM1 | intercellular adhesion molecule 1 | [3383](https://www.ncbi.nlm.nih.gov/gene/?term=3383) | 1.7 | 0.00622 | 835.1 | 466.7 |
| B3GNT5 | UDP-GlcNAc:betaGal beta-1,3-N-acetylglucosaminyltransferase 5 | [84002](https://www.ncbi.nlm.nih.gov/gene/?term=84002) | 1.7 | 0.00599 | 853.6 | 475.5 |
| PLAUR | plasminogen activator, urokinase receptor | [5329](https://www.ncbi.nlm.nih.gov/gene/?term=5329) | 1.7 | 0.00028 | 1446.8 | 839.5 |
| SOCS1 | suppressor of cytokine signaling 1 | [8651](https://www.ncbi.nlm.nih.gov/gene/?term=8651) | 1.7 | 0.03226 | 44.2 | 22.2 |
| LOC101926893 |  | [101926893](https://www.ncbi.nlm.nih.gov/gene/?term=101926893) | -1.7 | 0.01456 | 254.8 | 474.4 |
| HEY1 | hes related family bHLH transcription factor with YRPW motif 1 | [23462](https://www.ncbi.nlm.nih.gov/gene/?term=23462) | -1.7 | 0.05046 | 3.4 | 7.9 |
| DZIP3 | DAZ interacting zinc finger protein 3 | [9666](https://www.ncbi.nlm.nih.gov/gene/?term=9666) | -1.7 | 0.00659 | 696.2 | 1252.0 |
| RFX7 | regulatory factor X7 | [64864](https://www.ncbi.nlm.nih.gov/gene/?term=64864) | -1.7 | 0.00366 | 601.3 | 1073.8 |
| CACNG8 | calcium voltage-gated channel auxiliary subunit gamma 8 | [59283](https://www.ncbi.nlm.nih.gov/gene/?term=59283) | -1.7 | 0.05302 | 3.7 | 9.1 |
| FAM102A | family with sequence similarity 102 member A | [399665](https://www.ncbi.nlm.nih.gov/gene/?term=399665) | -1.7 | 0.01942 | 151.1 | 286.8 |
| PACSIN2 | protein kinase C and casein kinase substrate in neurons 2 | [11252](https://www.ncbi.nlm.nih.gov/gene/?term=11252) | 1.7 | 0.00042 | 4512.4 | 2608.8 |
| MBD2 | methyl-CpG binding domain protein 2 | [8932](https://www.ncbi.nlm.nih.gov/gene/?term=8932) | 1.7 | 0.00000 | 3429.1 | 2030.1 |
| HRASLS | HRAS like suppressor | [57110](https://www.ncbi.nlm.nih.gov/gene/?term=57110) | -1.7 | 0.04075 | 10.5 | 21.1 |
| SYCP2L | synaptonemal complex protein 2 like | [221711](https://www.ncbi.nlm.nih.gov/gene/?term=221711) | -1.7 | 0.02979 | 23.3 | 45.5 |
| MAP3K14 | mitogen-activated protein kinase kinase kinase 14 | [9020](https://www.ncbi.nlm.nih.gov/gene/?term=9020) | -1.7 | 0.00368 | 121.7 | 217.7 |
| TMEM168 | transmembrane protein 168 | [64418](https://www.ncbi.nlm.nih.gov/gene/?term=64418) | -1.7 | 0.00014 | 609.4 | 1047.0 |
| ZNF808 | zinc finger protein 808 | [388558](https://www.ncbi.nlm.nih.gov/gene/?term=388558) | -1.7 | 0.00000 | 552.7 | 935.1 |
| LOC107984026 |  | [107984026](https://www.ncbi.nlm.nih.gov/gene/?term=107984026) | -1.7 | 0.02021 | 26.6 | 49.6 |
| EHBP1L1 | EH domain binding protein 1 like 1 | [254102](https://www.ncbi.nlm.nih.gov/gene/?term=254102) | 1.7 | 0.00054 | 6232.5 | 3598.7 |
| PLCB1 | phospholipase C beta 1 | [23236](https://www.ncbi.nlm.nih.gov/gene/?term=23236) | -1.7 | 0.00020 | 161.2 | 278.2 |
| ZMYND15 | zinc finger MYND-type containing 15 | [84225](https://www.ncbi.nlm.nih.gov/gene/?term=84225) | 1.7 | 0.02739 | 18.3 | 9.7 |
| PARP14 | poly(ADP-ribose) polymerase family member 14 | [54625](https://www.ncbi.nlm.nih.gov/gene/?term=54625) | 1.7 | 0.01597 | 29543.2 | 16039.4 |
| SIK2 | salt inducible kinase 2 | [23235](https://www.ncbi.nlm.nih.gov/gene/?term=23235) | -1.7 | 0.00156 | 150.2 | 264.7 |
| LYPLA1 | lysophospholipase I | [10434](https://www.ncbi.nlm.nih.gov/gene/?term=10434) | 1.7 | 0.00002 | 1810.5 | 1071.4 |
| ZNF222 | zinc finger protein 222 | [7673](https://www.ncbi.nlm.nih.gov/gene/?term=7673) | 1.7 | 0.00007 | 184.4 | 108.3 |
| RPL21P51 | ribosomal protein L21 pseudogene 51 | [100271169](https://www.ncbi.nlm.nih.gov/gene/?term=100271169) | -1.7 | 0.05868 | 1.8 | 4.8 |
| RNH1 | ribonuclease/angiogenin inhibitor 1 | [6050](https://www.ncbi.nlm.nih.gov/gene/?term=6050) | 1.7 | 0.01922 | 24.3 | 12.4 |
| LOC105371472 |  | [105371472](https://www.ncbi.nlm.nih.gov/gene/?term=105371472) | 1.6 | 0.04593 | 16.5 | 8.0 |
| CD48 | CD48 molecule | [962](https://www.ncbi.nlm.nih.gov/gene/?term=962) | 1.6 | 0.00004 | 8390.2 | 4934.7 |
| UBE2B | ubiquitin conjugating enzyme E2 B | [7320](https://www.ncbi.nlm.nih.gov/gene/?term=7320) | -1.6 | 0.02009 | 7578.1 | 14645.2 |
| EXPH5 | exophilin 5 | [23086](https://www.ncbi.nlm.nih.gov/gene/?term=23086) | -1.6 | 0.00380 | 44.0 | 78.7 |
| LOC107985971 |  | [107985971](https://www.ncbi.nlm.nih.gov/gene/?term=107985971) | -1.6 | 0.00004 | 753.0 | 1283.3 |
| GPR153 | G protein-coupled receptor 153 | [387509](https://www.ncbi.nlm.nih.gov/gene/?term=387509) | -1.6 | 0.04303 | 4.8 | 10.0 |
| ZNF230 | zinc finger protein 230 | [7773](https://www.ncbi.nlm.nih.gov/gene/?term=7773) | 1.6 | 0.00155 | 831.0 | 475.7 |
| NAP1L1 | nucleosome assembly protein 1 like 1 | [4673](https://www.ncbi.nlm.nih.gov/gene/?term=4673) | -1.6 | 0.00011 | 3142.3 | 5371.2 |
| MEGF9 | multiple EGF like domains 9 | [1955](https://www.ncbi.nlm.nih.gov/gene/?term=1955) | -1.6 | 0.00353 | 7560.2 | 13309.5 |
| SLC39A13 | solute carrier family 39 member 13 | [91252](https://www.ncbi.nlm.nih.gov/gene/?term=91252) | 1.6 | 0.00000 | 148.3 | 88.7 |
| PLCH1 | phospholipase C eta 1 | [23007](https://www.ncbi.nlm.nih.gov/gene/?term=23007) | -1.6 | 0.03673 | 31.9 | 64.2 |
| LOC553103 | uncharacterized LOC553103 | [553103](https://www.ncbi.nlm.nih.gov/gene/?term=553103) | -1.6 | 0.01584 | 10.4 | 19.7 |
| ATP6V0E1 | ATPase H+ transporting V0 subunit e1 | [8992](https://www.ncbi.nlm.nih.gov/gene/?term=8992) | 1.6 | 0.00010 | 5669.4 | 3326.1 |
| PAQR8 | progestin and adipoQ receptor family member 8 | [85315](https://www.ncbi.nlm.nih.gov/gene/?term=85315) | -1.6 | 0.00034 | 244.0 | 421.5 |
| LGALS8 | lectin, galactoside binding soluble 8 | [3964](https://www.ncbi.nlm.nih.gov/gene/?term=3964) | 1.6 | 0.00026 | 4033.8 | 2350.1 |
| CEBPB-AS1 | CEBPB antisense RNA 1 | [101927559](https://www.ncbi.nlm.nih.gov/gene/?term=101927559) | 1.6 | 0.00619 | 50.6 | 28.2 |
| ZNF571 | zinc finger protein 571 | [51276](https://www.ncbi.nlm.nih.gov/gene/?term=51276) | -1.6 | 0.00000 | 86.9 | 146.2 |
| LOC105373785 |  | [105373785](https://www.ncbi.nlm.nih.gov/gene/?term=105373785) | -1.6 | 0.00781 | 36.4 | 66.6 |
| VIT | vitrin | [5212](https://www.ncbi.nlm.nih.gov/gene/?term=5212) | 1.6 | 0.05948 | 9.3 | 3.8 |
| SLCO4A1 | solute carrier organic anion transporter family member 4A1 | [28231](https://www.ncbi.nlm.nih.gov/gene/?term=28231) | 1.6 | 0.03074 | 55.0 | 28.2 |
| ENTPD5 | ectonucleoside triphosphate diphosphohydrolase 5 | [957](https://www.ncbi.nlm.nih.gov/gene/?term=957) | -1.6 | 0.01805 | 1301.4 | 2449.7 |
| DCAF12 | DDB1 and CUL4 associated factor 12 | [25853](https://www.ncbi.nlm.nih.gov/gene/?term=25853) | -1.6 | 0.03396 | 139578.6 | 296789.9 |
| YLPM1 | YLP motif containing 1 | [56252](https://www.ncbi.nlm.nih.gov/gene/?term=56252) | -1.6 | 0.00033 | 648.0 | 1114.2 |
| ACTA2-AS1 | ACTA2 antisense RNA 1 | [100132116](https://www.ncbi.nlm.nih.gov/gene/?term=100132116) | -1.6 | 0.04555 | 17.1 | 37.5 |
| RPUSD1 | RNA pseudouridylate synthase domain containing 1 | [113000](https://www.ncbi.nlm.nih.gov/gene/?term=113000) | 1.6 | 0.00093 | 89.4 | 51.2 |
| RPA1 | replication protein A1 | [6117](https://www.ncbi.nlm.nih.gov/gene/?term=6117) | -1.6 | 0.00203 | 2118.8 | 3709.0 |
| STAT2 | signal transducer and activator of transcription 2 | [6773](https://www.ncbi.nlm.nih.gov/gene/?term=6773) | 1.6 | 0.01410 | 4449.3 | 2409.9 |
| QSOX1 | quiescin sulfhydryl oxidase 1 | [5768](https://www.ncbi.nlm.nih.gov/gene/?term=5768) | 1.6 | 0.00027 | 728.6 | 426.1 |
| MIR573 | microRNA 573 | [693158](https://www.ncbi.nlm.nih.gov/gene/?term=693158) | -1.6 | 0.04794 | 4.9 | 11.0 |
| LINC01136 | long intergenic non-protein coding RNA 1136 | [730227](https://www.ncbi.nlm.nih.gov/gene/?term=730227) | 1.6 | 0.00594 | 64.0 | 35.5 |
| LOC107985876 |  | [107985876](https://www.ncbi.nlm.nih.gov/gene/?term=107985876) | -1.6 | 0.05364 | 6.4 | 16.2 |
| LOC105371461 |  | [105371461](https://www.ncbi.nlm.nih.gov/gene/?term=105371461) | 1.6 | 0.04687 | 40.4 | 18.9 |
| PCDH9 | protocadherin 9 | [5101](https://www.ncbi.nlm.nih.gov/gene/?term=5101) | -1.6 | 0.00673 | 334.3 | 599.0 |
| CARD19 | caspase recruitment domain family member 19 | [84270](https://www.ncbi.nlm.nih.gov/gene/?term=84270) | 1.6 | 0.00045 | 778.6 | 451.8 |
| SELL | selectin L | [6402](https://www.ncbi.nlm.nih.gov/gene/?term=6402) | 1.6 | 0.00204 | 32609.7 | 18717.2 |
| DDIAS | DNA damage induced apoptosis suppressor | [220042](https://www.ncbi.nlm.nih.gov/gene/?term=220042) | 1.6 | 0.00382 | 249.5 | 141.3 |
| LOC101927300 |  | [101927300](https://www.ncbi.nlm.nih.gov/gene/?term=101927300) | -1.6 | 0.01577 | 22.2 | 42.0 |
| AGL | amylo-alpha-1, 6-glucosidase, 4-alpha-glucanotransferase | [178](https://www.ncbi.nlm.nih.gov/gene/?term=178) | -1.6 | 0.00009 | 562.9 | 959.8 |
| SNORA70 | small nucleolar RNA, H/ACA box 70 | [26778](https://www.ncbi.nlm.nih.gov/gene/?term=26778) | 1.6 | 0.00936 | 79.5 | 43.9 |
| MANEA-AS1 | MANEA antisense RNA 1 (head to head) | [101927288](https://www.ncbi.nlm.nih.gov/gene/?term=101927288) | -1.6 | 0.00132 | 62.2 | 109.8 |
| S100A6 | S100 calcium binding protein A6 | [6277](https://www.ncbi.nlm.nih.gov/gene/?term=6277) | 1.6 | 0.00016 | 3271.9 | 1916.5 |
| SIPA1L3 | signal induced proliferation associated 1 like 3 | [23094](https://www.ncbi.nlm.nih.gov/gene/?term=23094) | -1.6 | 0.00931 | 159.9 | 290.5 |
| MAN1B1-AS1 | MAN1B1 antisense RNA 1 (head to head) | [100289341](https://www.ncbi.nlm.nih.gov/gene/?term=100289341) | 1.6 | 0.03483 | 11.1 | 5.5 |
| MAST3 | microtubule associated serine/threonine kinase 3 | [23031](https://www.ncbi.nlm.nih.gov/gene/?term=23031) | -1.6 | 0.00268 | 933.6 | 1638.9 |
| LINC00884 | long intergenic non-protein coding RNA 884 | [401106](https://www.ncbi.nlm.nih.gov/gene/?term=401106) | 1.6 | 0.05823 | 9.2 | 4.0 |
| LOC105372991 |  | [105372991](https://www.ncbi.nlm.nih.gov/gene/?term=105372991) | -1.6 | 0.03360 | 312.4 | 623.8 |
| PGM1 | phosphoglucomutase 1 | [5236](https://www.ncbi.nlm.nih.gov/gene/?term=5236) | 1.6 | 0.00008 | 632.9 | 373.0 |
| JDP2 | Jun dimerization protein 2 | [122953](https://www.ncbi.nlm.nih.gov/gene/?term=122953) | 1.6 | 0.00120 | 719.0 | 415.8 |
| BAZ1A | bromodomain adjacent to zinc finger domain 1A | [11177](https://www.ncbi.nlm.nih.gov/gene/?term=11177) | 1.6 | 0.00092 | 11246.4 | 6515.3 |
| PGAM1P8 | phosphoglycerate mutase 1 pseudogene 8 | [440043](https://www.ncbi.nlm.nih.gov/gene/?term=440043) | -1.6 | 0.04028 | 6.0 | 12.4 |
| RETSAT | retinol saturase (all-trans-retinol 13,14-reductase) | [54884](https://www.ncbi.nlm.nih.gov/gene/?term=54884) | 1.6 | 0.00000 | 482.0 | 287.6 |
| B3GAT3 | beta-1,3-glucuronyltransferase 3 | [26229](https://www.ncbi.nlm.nih.gov/gene/?term=26229) | 1.6 | 0.00001 | 277.1 | 164.1 |
| RPL17P50 | ribosomal protein L17 pseudogene 50 | [729046](https://www.ncbi.nlm.nih.gov/gene/?term=729046) | -1.6 | 0.01772 | 9.7 | 19.4 |
| MOB1B | MOB kinase activator 1B | [92597](https://www.ncbi.nlm.nih.gov/gene/?term=92597) | -1.6 | 0.00020 | 2214.7 | 3769.0 |
| LOC105369720 |  | [105369720](https://www.ncbi.nlm.nih.gov/gene/?term=105369720) | -1.6 | 0.03988 | 8.2 | 18.0 |
| TMEM56-RWDD3 | TMEM56-RWDD3 readthrough | [100527978](https://www.ncbi.nlm.nih.gov/gene/?term=100527978) | -1.6 | 0.02224 | 175.4 | 329.8 |
| WIZ | widely interspaced zinc finger motifs | [58525](https://www.ncbi.nlm.nih.gov/gene/?term=58525) | -1.6 | 0.01364 | 81.6 | 151.4 |
| CREBBP | CREB binding protein | [1387](https://www.ncbi.nlm.nih.gov/gene/?term=1387) | -1.6 | 0.00121 | 2039.9 | 3522.9 |
| LOC105376060 |  | [105376060](https://www.ncbi.nlm.nih.gov/gene/?term=105376060) | -1.6 | 0.03726 | 17.5 | 35.8 |
| RAB43 | RAB43, member RAS oncogene family | [339122](https://www.ncbi.nlm.nih.gov/gene/?term=339122) | 1.6 | 0.00002 | 111.2 | 66.7 |
| GRAMD1B | GRAM domain containing 1B | [57476](https://www.ncbi.nlm.nih.gov/gene/?term=57476) | 1.6 | 0.03801 | 477.4 | 237.0 |
| HRH4 | histamine receptor H4 | [59340](https://www.ncbi.nlm.nih.gov/gene/?term=59340) | -1.6 | 0.01876 | 106.3 | 196.7 |
| ZDHHC2 | zinc finger DHHC-type containing 2 | [51201](https://www.ncbi.nlm.nih.gov/gene/?term=51201) | -1.6 | 0.01113 | 3590.6 | 6438.3 |
| LOC107985893 |  | [107985893](https://www.ncbi.nlm.nih.gov/gene/?term=107985893) | 1.6 | 0.03569 | 18.1 | 9.3 |
| TMEM56 | transmembrane protein 56 | [148534](https://www.ncbi.nlm.nih.gov/gene/?term=148534) | -1.6 | 0.02964 | 3572.6 | 6979.6 |
| PTGFRN | prostaglandin F2 receptor inhibitor | [5738](https://www.ncbi.nlm.nih.gov/gene/?term=5738) | 1.6 | 0.02237 | 59.1 | 31.3 |
| ZCCHC11 | zinc finger CCHC-type containing 11 | [23318](https://www.ncbi.nlm.nih.gov/gene/?term=23318) | -1.6 | 0.00000 | 1215.1 | 2032.7 |
| MYL12A | myosin light chain 12A | [10627](https://www.ncbi.nlm.nih.gov/gene/?term=10627) | 1.6 | 0.00014 | 14029.5 | 8281.7 |
| OSTF1 | osteoclast stimulating factor 1 | [26578](https://www.ncbi.nlm.nih.gov/gene/?term=26578) | 1.6 | 0.00026 | 4979.3 | 2927.5 |
| SNORD116-6 | small nucleolar RNA, C/D box 116-6 | [100033418](https://www.ncbi.nlm.nih.gov/gene/?term=100033418) | 1.6 | 0.05785 | 6.4 | 2.7 |
| TBCC | tubulin folding cofactor C | [6903](https://www.ncbi.nlm.nih.gov/gene/?term=6903) | 1.6 | 0.00004 | 1309.6 | 778.4 |
| VPS16 | VPS16, CORVET/HOPS core subunit | [64601](https://www.ncbi.nlm.nih.gov/gene/?term=64601) | 1.6 | 0.00000 | 809.9 | 488.0 |
| TMIGD3 | transmembrane and immunoglobulin domain containing 3 | [57413](https://www.ncbi.nlm.nih.gov/gene/?term=57413) | 1.6 | 0.03170 | 18.1 | 9.3 |
| HINT3 | histidine triad nucleotide binding protein 3 | [135114](https://www.ncbi.nlm.nih.gov/gene/?term=135114) | 1.6 | 0.00010 | 1178.9 | 697.1 |
| ANKDD1A | ankyrin repeat and death domain containing 1A | [348094](https://www.ncbi.nlm.nih.gov/gene/?term=348094) | 1.6 | 0.04921 | 85.1 | 39.6 |
| PARD3B | par-3 family cell polarity regulator beta | [117583](https://www.ncbi.nlm.nih.gov/gene/?term=117583) | -1.6 | 0.05506 | 7.7 | 18.3 |
| FOXO1 | forkhead box O1 | [2308](https://www.ncbi.nlm.nih.gov/gene/?term=2308) | -1.6 | 0.00002 | 1196.7 | 2016.3 |
| KCNH8 | potassium voltage-gated channel subfamily H member 8 | [131096](https://www.ncbi.nlm.nih.gov/gene/?term=131096) | -1.6 | 0.01838 | 52.6 | 97.4 |
| CLDND2 | claudin domain containing 2 | [125875](https://www.ncbi.nlm.nih.gov/gene/?term=125875) | 1.6 | 0.03996 | 47.0 | 23.3 |
| GLT1D1 | glycosyltransferase 1 domain containing 1 | [144423](https://www.ncbi.nlm.nih.gov/gene/?term=144423) | 1.6 | 0.00428 | 2494.4 | 1418.3 |
| FAM72D | family with sequence similarity 72 member D | [728833](https://www.ncbi.nlm.nih.gov/gene/?term=728833) | 1.6 | 0.02815 | 11.7 | 5.9 |
| SLC29A1 | solute carrier family 29 member 1 (Augustine blood group) | [2030](https://www.ncbi.nlm.nih.gov/gene/?term=2030) | -1.6 | 0.03180 | 45.3 | 88.9 |
| TAF6 | TATA-box binding protein associated factor 6 | [6878](https://www.ncbi.nlm.nih.gov/gene/?term=6878) | -1.6 | 0.00384 | 110.4 | 195.0 |
| IDH2 | isocitrate dehydrogenase (NADP(+)) 2, mitochondrial | [3418](https://www.ncbi.nlm.nih.gov/gene/?term=3418) | 1.6 | 0.00011 | 1457.4 | 861.2 |
| LOC107985180 |  | [107985180](https://www.ncbi.nlm.nih.gov/gene/?term=107985180) | 1.6 | 0.04001 | 12.4 | 6.3 |
| LRFN4 | leucine rich repeat and fibronectin type III domain containing 4 | [78999](https://www.ncbi.nlm.nih.gov/gene/?term=78999) | 1.6 | 0.00661 | 51.7 | 28.5 |
| APH1A | aph-1 homolog A, gamma secretase subunit | [51107](https://www.ncbi.nlm.nih.gov/gene/?term=51107) | 1.6 | 0.00001 | 2054.1 | 1226.0 |
| MPDU1 | mannose-P-dolichol utilization defect 1 | [9526](https://www.ncbi.nlm.nih.gov/gene/?term=9526) | 1.6 | 0.00000 | 479.6 | 285.5 |
| ALDH2 | aldehyde dehydrogenase 2 family (mitochondrial) | [217](https://www.ncbi.nlm.nih.gov/gene/?term=217) | 1.6 | 0.00507 | 889.0 | 503.3 |
| MARCKSL1 | MARCKS-like 1 | [65108](https://www.ncbi.nlm.nih.gov/gene/?term=65108) | -1.6 | 0.00120 | 66.5 | 116.1 |
| POLD1 | polymerase (DNA) delta 1, catalytic subunit | [5424](https://www.ncbi.nlm.nih.gov/gene/?term=5424) | 1.6 | 0.00004 | 475.9 | 282.3 |
| SLC35E4 | solute carrier family 35 member E4 | [339665](https://www.ncbi.nlm.nih.gov/gene/?term=339665) | 1.6 | 0.00044 | 43.1 | 25.1 |
| EME1 | essential meiotic structure-specific endonuclease 1 | [146956](https://www.ncbi.nlm.nih.gov/gene/?term=146956) | 1.6 | 0.03673 | 36.0 | 18.3 |
| PLA2G4C | phospholipase A2 group IVC | [8605](https://www.ncbi.nlm.nih.gov/gene/?term=8605) | 1.6 | 0.04050 | 107.7 | 53.0 |
| SMAP2 | small ArfGAP2 | [64744](https://www.ncbi.nlm.nih.gov/gene/?term=64744) | -1.6 | 0.00115 | 2964.4 | 5103.1 |
| NUDT18 | nudix hydrolase 18 | [79873](https://www.ncbi.nlm.nih.gov/gene/?term=79873) | 1.6 | 0.00009 | 223.4 | 132.1 |
| LOC107984561 |  | [107984561](https://www.ncbi.nlm.nih.gov/gene/?term=107984561) | 1.6 | 0.00306 | 77.5 | 44.6 |
| CIDEB | cell death-inducing DFFA-like effector b | [27141](https://www.ncbi.nlm.nih.gov/gene/?term=27141) | 1.6 | 0.00028 | 608.1 | 356.9 |
| LOC107984543 |  | [107984543](https://www.ncbi.nlm.nih.gov/gene/?term=107984543) | -1.6 | 0.01002 | 8.2 | 13.4 |
| ST8SIA6 | ST8 alpha-N-acetylneuraminate alpha-2,8-sialyltransferase 6 | [338596](https://www.ncbi.nlm.nih.gov/gene/?term=338596) | -1.6 | 0.00161 | 93.3 | 161.2 |
| GALNT6 | polypeptide N-acetylgalactosaminyltransferase 6 | [11226](https://www.ncbi.nlm.nih.gov/gene/?term=11226) | -1.6 | 0.03018 | 719.9 | 1397.1 |
| HEXB | hexosaminidase subunit beta | [3074](https://www.ncbi.nlm.nih.gov/gene/?term=3074) | 1.6 | 0.00004 | 2221.1 | 1321.6 |
| LOC100507577 | uncharacterized LOC100507577 | [100507577](https://www.ncbi.nlm.nih.gov/gene/?term=100507577) | -1.6 | 0.00194 | 100.4 | 175.2 |
| FIBP | FGF1 intracellular binding protein | [9158](https://www.ncbi.nlm.nih.gov/gene/?term=9158) | 1.6 | 0.00000 | 503.9 | 303.7 |
| SYNJ1 | synaptojanin 1 | [8867](https://www.ncbi.nlm.nih.gov/gene/?term=8867) | -1.6 | 0.00289 | 644.9 | 1125.4 |
| CMTM6 | CKLF like MARVEL transmembrane domain containing 6 | [54918](https://www.ncbi.nlm.nih.gov/gene/?term=54918) | 1.6 | 0.00093 | 15403.6 | 8989.0 |
| WHSC1L1 | Wolf-Hirschhorn syndrome candidate 1-like 1 | [54904](https://www.ncbi.nlm.nih.gov/gene/?term=54904) | -1.6 | 0.00013 | 4229.8 | 7132.8 |
| PYCARD | PYD and CARD domain containing | [29108](https://www.ncbi.nlm.nih.gov/gene/?term=29108) | 1.6 | 0.00061 | 2250.7 | 1316.7 |
| MPZ | myelin protein zero | [4359](https://www.ncbi.nlm.nih.gov/gene/?term=4359) | 1.6 | 0.00473 | 125.5 | 72.0 |
| FCMR | Fc fragment of IgM receptor | [9214](https://www.ncbi.nlm.nih.gov/gene/?term=9214) | -1.6 | 0.00595 | 562.3 | 994.7 |
| CORO2B | coronin 2B | [10391](https://www.ncbi.nlm.nih.gov/gene/?term=10391) | -1.6 | 0.04746 | 9.5 | 20.7 |
| LOC100507642 | uncharacterized LOC100507642 | [100507642](https://www.ncbi.nlm.nih.gov/gene/?term=100507642) | 1.6 | 0.03211 | 12.3 | 6.1 |
| CCDC6 | coiled-coil domain containing 6 | [8030](https://www.ncbi.nlm.nih.gov/gene/?term=8030) | -1.6 | 0.00110 | 604.9 | 1039.7 |
| NIPBL | NIPBL, cohesin loading factor | [25836](https://www.ncbi.nlm.nih.gov/gene/?term=25836) | -1.6 | 0.00002 | 6635.5 | 11101.7 |
| ZNF652 | zinc finger protein 652 | [22834](https://www.ncbi.nlm.nih.gov/gene/?term=22834) | -1.6 | 0.00053 | 2260.1 | 3851.0 |
| IFNG | interferon, gamma | [3458](https://www.ncbi.nlm.nih.gov/gene/?term=3458) | 1.6 | 0.04188 | 40.1 | 19.9 |
| LOC107985030 |  | [107985030](https://www.ncbi.nlm.nih.gov/gene/?term=107985030) | 1.6 | 0.01387 | 21.0 | 11.6 |
| IDNK | idnK, gluconokinase homolog (E. coli) | [414328](https://www.ncbi.nlm.nih.gov/gene/?term=414328) | 1.6 | 0.00004 | 190.1 | 113.1 |
| C7orf31 | chromosome 7 open reading frame 31 | [136895](https://www.ncbi.nlm.nih.gov/gene/?term=136895) | -1.6 | 0.00263 | 34.9 | 62.2 |
| THBS3 | thrombospondin 3 | [7059](https://www.ncbi.nlm.nih.gov/gene/?term=7059) | 1.6 | 0.00016 | 362.2 | 214.5 |
| LRBA | LPS responsive beige-like anchor protein | [987](https://www.ncbi.nlm.nih.gov/gene/?term=987) | -1.6 | 0.00004 | 4040.2 | 6765.6 |
| MX2 | MX dynamin like GTPase 2 | [4600](https://www.ncbi.nlm.nih.gov/gene/?term=4600) | 1.6 | 0.01481 | 6110.5 | 3363.5 |
| PSTPIP1 | proline-serine-threonine phosphatase interacting protein 1 | [9051](https://www.ncbi.nlm.nih.gov/gene/?term=9051) | 1.6 | 0.00021 | 1719.7 | 1016.8 |
| LOC285074 | anaphase promoting complex subunit 1 pseudogene | [285074](https://www.ncbi.nlm.nih.gov/gene/?term=285074) | -1.6 | 0.00525 | 614.3 | 1077.5 |
| FAM218A | family with sequence similarity 218 member A | [152756](https://www.ncbi.nlm.nih.gov/gene/?term=152756) | -1.6 | 0.02046 | 10.4 | 20.0 |
| LOC105371268 |  | [105371268](https://www.ncbi.nlm.nih.gov/gene/?term=105371268) | 1.6 | 0.05847 | 8.9 | 3.9 |
| SMARCD2 | SWI/SNF related, matrix associated, actin dependent regulator of chromatin, subfamily d, member 2 | [6603](https://www.ncbi.nlm.nih.gov/gene/?term=6603) | 1.6 | 0.00000 | 2692.3 | 1620.2 |
| CETP | cholesteryl ester transfer protein | [1071](https://www.ncbi.nlm.nih.gov/gene/?term=1071) | 1.6 | 0.01829 | 28.4 | 15.3 |
| SLC27A2 | solute carrier family 27 member 2 | [11001](https://www.ncbi.nlm.nih.gov/gene/?term=11001) | 1.6 | 0.03837 | 47.4 | 24.0 |
| CDT1 | chromatin licensing and DNA replication factor 1 | [81620](https://www.ncbi.nlm.nih.gov/gene/?term=81620) | 1.6 | 0.05382 | 38.7 | 17.6 |
| LMOD1 | leiomodin 1 | [25802](https://www.ncbi.nlm.nih.gov/gene/?term=25802) | -1.6 | 0.05082 | 37.2 | 110.9 |
| CORIN | corin, serine peptidase | [10699](https://www.ncbi.nlm.nih.gov/gene/?term=10699) | 1.6 | 0.05622 | 59.9 | 26.3 |
| AURKAIP1 | aurora kinase A interacting protein 1 | [54998](https://www.ncbi.nlm.nih.gov/gene/?term=54998) | 1.6 | 0.00000 | 1074.0 | 647.3 |
| MMADHC | methylmalonic aciduria and homocystinuria, cblD type | [27249](https://www.ncbi.nlm.nih.gov/gene/?term=27249) | 1.6 | 0.00009 | 2762.7 | 1646.0 |
| NDUFB3P2 | NADH:ubiquinone oxidoreductase subunit B3 pseudogene 2 | [93994](https://www.ncbi.nlm.nih.gov/gene/?term=93994) | -1.6 | 0.05511 | 2.7 | 6.7 |
| ZNF727 | zinc finger protein 727 | [442319](https://www.ncbi.nlm.nih.gov/gene/?term=442319) | -1.6 | 0.05056 | 9.4 | 20.5 |
| GTF2F1 | general transcription factor IIF subunit 1 | [2962](https://www.ncbi.nlm.nih.gov/gene/?term=2962) | 1.6 | 0.00000 | 1162.5 | 698.5 |
| LMAN2 | lectin, mannose binding 2 | [10960](https://www.ncbi.nlm.nih.gov/gene/?term=10960) | 1.6 | 0.00001 | 2962.3 | 1787.4 |
| ARMC3 | armadillo repeat containing 3 | [219681](https://www.ncbi.nlm.nih.gov/gene/?term=219681) | -1.6 | 0.01630 | 35.3 | 63.8 |
| B4GALT6 | beta-1,4-galactosyltransferase 6 | [9331](https://www.ncbi.nlm.nih.gov/gene/?term=9331) | -1.6 | 0.00211 | 56.1 | 95.8 |
| CHEK1 | checkpoint kinase 1 | [1111](https://www.ncbi.nlm.nih.gov/gene/?term=1111) | 1.6 | 0.00388 | 192.7 | 110.8 |
| LOC102723663 |  | [102723663](https://www.ncbi.nlm.nih.gov/gene/?term=102723663) | 1.6 | 0.03359 | 24.8 | 12.6 |
| LOC107985217 |  | [107985217](https://www.ncbi.nlm.nih.gov/gene/?term=107985217) | 1.6 | 0.05869 | 12.5 | 5.5 |
| TNFRSF11A | tumor necrosis factor receptor superfamily member 11a | [8792](https://www.ncbi.nlm.nih.gov/gene/?term=8792) | -1.6 | 0.01335 | 13.5 | 25.0 |
| AKR1C1 | aldo-keto reductase family 1, member C1 | [1645](https://www.ncbi.nlm.nih.gov/gene/?term=1645) | 1.6 | 0.04593 | 86.4 | 41.4 |
| TMEM121 | transmembrane protein 121 | [80757](https://www.ncbi.nlm.nih.gov/gene/?term=80757) | 1.6 | 0.04577 | 11.7 | 5.9 |
| LOC104968399 | uncharacterized LOC104968399 | [104968399](https://www.ncbi.nlm.nih.gov/gene/?term=104968399) | 1.6 | 0.00934 | 28.5 | 16.0 |
| RAB2B | RAB2B, member RAS oncogene family | [84932](https://www.ncbi.nlm.nih.gov/gene/?term=84932) | -1.6 | 0.01996 | 14897.1 | 27759.9 |
| RNU6-5P | RNA, U6 small nuclear 5, pseudogene | [106478913](https://www.ncbi.nlm.nih.gov/gene/?term=106478913) | -1.6 | 0.01742 | 34.4 | 64.0 |
| LOC101926967 |  | [101926967](https://www.ncbi.nlm.nih.gov/gene/?term=101926967) | -1.6 | 0.00923 | 12.9 | 24.2 |
| EIF4G1 | eukaryotic translation initiation factor 4 gamma 1 | [1981](https://www.ncbi.nlm.nih.gov/gene/?term=1981) | -1.6 | 0.00184 | 2109.2 | 3626.0 |
| LOC107985986 |  | [107985986](https://www.ncbi.nlm.nih.gov/gene/?term=107985986) | -1.6 | 0.05711 | 4.4 | 10.8 |
| TPPP | tubulin polymerization promoting protein | [11076](https://www.ncbi.nlm.nih.gov/gene/?term=11076) | -1.6 | 0.02742 | 11.9 | 23.0 |
| LOC105376103 |  | [105376103](https://www.ncbi.nlm.nih.gov/gene/?term=105376103) | -1.6 | 0.05491 | 10.4 | 24.3 |
| NCF1B | neutrophil cytosolic factor 1B pseudogene | [654816](https://www.ncbi.nlm.nih.gov/gene/?term=654816) | 1.6 | 0.00229 | 990.4 | 575.5 |
| LOC107986433 |  | [107986433](https://www.ncbi.nlm.nih.gov/gene/?term=107986433) | -1.6 | 0.01897 | 60.7 | 112.9 |
| STX12 | syntaxin 12 | [23673](https://www.ncbi.nlm.nih.gov/gene/?term=23673) | 1.6 | 0.00001 | 1515.0 | 911.7 |
| GTF2IP18 |  | [102723643](https://www.ncbi.nlm.nih.gov/gene/?term=102723643) | 1.6 | 0.03873 | 16.1 | 8.5 |
| LINC00342 | long intergenic non-protein coding RNA 342 | [150759](https://www.ncbi.nlm.nih.gov/gene/?term=150759) | -1.6 | 0.02088 | 91.1 | 171.9 |
| LGALS3BP | lectin, galactoside binding soluble 3 binding protein | [3959](https://www.ncbi.nlm.nih.gov/gene/?term=3959) | 1.6 | 0.04067 | 214.7 | 107.4 |
| SOWAHC | sosondowah ankyrin repeat domain family member C | [65124](https://www.ncbi.nlm.nih.gov/gene/?term=65124) | 1.6 | 0.00595 | 63.5 | 36.3 |
| NCF2 | neutrophil cytosolic factor 2 | [4688](https://www.ncbi.nlm.nih.gov/gene/?term=4688) | 1.6 | 0.00132 | 22431.2 | 13122.3 |
| USP34 | ubiquitin specific peptidase 34 | [9736](https://www.ncbi.nlm.nih.gov/gene/?term=9736) | -1.6 | 0.00000 | 8918.0 | 14758.3 |
| ZXDB | zinc finger, X-linked, duplicated B | [158586](https://www.ncbi.nlm.nih.gov/gene/?term=158586) | -1.6 | 0.00805 | 94.4 | 167.7 |
| ARID5A | AT-rich interaction domain 5A | [10865](https://www.ncbi.nlm.nih.gov/gene/?term=10865) | 1.6 | 0.00224 | 624.4 | 363.0 |
| LOC102724714 | uncharacterized LOC102724714 | [102724714](https://www.ncbi.nlm.nih.gov/gene/?term=102724714) | -1.6 | 0.02022 | 23.2 | 43.1 |
| FECH | ferrochelatase | [2235](https://www.ncbi.nlm.nih.gov/gene/?term=2235) | -1.6 | 0.06396 | 48477.5 | 97152.9 |
| H1FX-AS1 | H1FX antisense RNA 1 | [339942](https://www.ncbi.nlm.nih.gov/gene/?term=339942) | 1.6 | 0.02751 | 18.1 | 9.6 |
| PCNA | proliferating cell nuclear antigen | [5111](https://www.ncbi.nlm.nih.gov/gene/?term=5111) | 1.6 | 0.00166 | 998.0 | 582.7 |
| UBE2C | ubiquitin conjugating enzyme E2 C | [11065](https://www.ncbi.nlm.nih.gov/gene/?term=11065) | 1.6 | 0.01213 | 124.3 | 69.5 |
| MYLIP | myosin regulatory light chain interacting protein | [29116](https://www.ncbi.nlm.nih.gov/gene/?term=29116) | 1.6 | 0.00017 | 3093.3 | 1835.0 |
| SMG1P1 | SMG1 pseudogene 1 | [641298](https://www.ncbi.nlm.nih.gov/gene/?term=641298) | -1.6 | 0.00607 | 325.5 | 571.9 |
| GPATCH8 | G-patch domain containing 8 | [23131](https://www.ncbi.nlm.nih.gov/gene/?term=23131) | -1.6 | 0.00044 | 761.7 | 1292.2 |
| EVI2A | ecotropic viral integration site 2A | [2123](https://www.ncbi.nlm.nih.gov/gene/?term=2123) | 1.6 | 0.00077 | 8282.7 | 4870.2 |
| LOC100996342 | uncharacterized LOC100996342 | [100996342](https://www.ncbi.nlm.nih.gov/gene/?term=100996342) | 1.6 | 0.05812 | 10.8 | 4.7 |
| CRK | v-crk avian sarcoma virus CT10 oncogene homolog | [1398](https://www.ncbi.nlm.nih.gov/gene/?term=1398) | 1.6 | 0.00007 | 218.9 | 130.8 |
| APOBEC3H | apolipoprotein B mRNA editing enzyme catalytic subunit 3H | [164668](https://www.ncbi.nlm.nih.gov/gene/?term=164668) | 1.6 | 0.03360 | 31.3 | 16.6 |
| ADK | adenosine kinase | [132](https://www.ncbi.nlm.nih.gov/gene/?term=132) | -1.6 | 0.00000 | 413.1 | 681.9 |
| LRRC8A | leucine-rich repeat containing 8 family member A | [56262](https://www.ncbi.nlm.nih.gov/gene/?term=56262) | -1.6 | 0.02701 | 1813.2 | 3449.3 |
| RNASE4 | ribonuclease A family member 4 | [6038](https://www.ncbi.nlm.nih.gov/gene/?term=6038) | 1.6 | 0.01668 | 112.6 | 61.7 |
| RAB8B | RAB8B, member RAS oncogene family | [51762](https://www.ncbi.nlm.nih.gov/gene/?term=51762) | 1.6 | 0.00001 | 9314.9 | 5611.3 |
| PIP5KL1 | phosphatidylinositol-4-phosphate 5-kinase like 1 | [138429](https://www.ncbi.nlm.nih.gov/gene/?term=138429) | -1.6 | 0.02759 | 65.5 | 122.5 |
| CHMP1B2P | charged multivesicular body protein 1B2, pseudogene | [101060146](https://www.ncbi.nlm.nih.gov/gene/?term=101060146) | -1.6 | 0.04515 | 36.5 | 74.5 |
| COL4A3 | collagen type IV alpha 3 | [1285](https://www.ncbi.nlm.nih.gov/gene/?term=1285) | -1.6 | 0.04690 | 28.6 | 59.9 |
| TWF2 | twinfilin actin binding protein 2 | [11344](https://www.ncbi.nlm.nih.gov/gene/?term=11344) | 1.6 | 0.00008 | 2261.9 | 1352.8 |
| APOL4 | apolipoprotein L4 | [80832](https://www.ncbi.nlm.nih.gov/gene/?term=80832) | 1.6 | 0.06034 | 67.0 | 28.7 |
| CCDC168 | coiled-coil domain containing 168 | [643677](https://www.ncbi.nlm.nih.gov/gene/?term=643677) | -1.6 | 0.04047 | 6.7 | 14.0 |
| PTTG1IP | pituitary tumor-transforming 1 interacting protein | [754](https://www.ncbi.nlm.nih.gov/gene/?term=754) | 1.6 | 0.00018 | 6085.8 | 3623.6 |
| RPS12P14 | ribosomal protein S12 pseudogene 14 | [100271058](https://www.ncbi.nlm.nih.gov/gene/?term=100271058) | -1.6 | 0.05424 | 4.5 | 14.3 |
| OGFOD2 | 2-oxoglutarate and iron dependent oxygenase domain containing 2 | [79676](https://www.ncbi.nlm.nih.gov/gene/?term=79676) | 1.6 | 0.00013 | 371.4 | 221.0 |
| TNFAIP8L2 | TNF alpha induced protein 8 like 2 | [79626](https://www.ncbi.nlm.nih.gov/gene/?term=79626) | 1.6 | 0.00003 | 1702.6 | 1024.1 |
| CNKSR2 | connector enhancer of kinase suppressor of Ras 2 | [22866](https://www.ncbi.nlm.nih.gov/gene/?term=22866) | -1.6 | 0.00114 | 132.6 | 225.9 |
| NIT1 | nitrilase 1 | [4817](https://www.ncbi.nlm.nih.gov/gene/?term=4817) | 1.6 | 0.00000 | 464.8 | 280.9 |
| LOC107986930 |  | [107986930](https://www.ncbi.nlm.nih.gov/gene/?term=107986930) | -1.6 | 0.03023 | 6.3 | 11.9 |
| UCKL1-AS1 | UCKL1 antisense RNA 1 | [100113386](https://www.ncbi.nlm.nih.gov/gene/?term=100113386) | 1.6 | 0.03437 | 16.3 | 8.9 |
| WARS | tryptophanyl-tRNA synthetase | [7453](https://www.ncbi.nlm.nih.gov/gene/?term=7453) | 1.6 | 0.00913 | 6550.2 | 3722.9 |
| C12orf75 | chromosome 12 open reading frame 75 | [387882](https://www.ncbi.nlm.nih.gov/gene/?term=387882) | 1.6 | 0.00178 | 914.5 | 534.2 |
| SRPK2 | SRSF protein kinase 2 | [6733](https://www.ncbi.nlm.nih.gov/gene/?term=6733) | -1.6 | 0.00010 | 1702.6 | 2855.5 |
| GNA11 | G protein subunit alpha 11 | [2767](https://www.ncbi.nlm.nih.gov/gene/?term=2767) | 1.6 | 0.00249 | 66.4 | 38.6 |
| LAT2 | linker for activation of T-cells family member 2 | [7462](https://www.ncbi.nlm.nih.gov/gene/?term=7462) | 1.6 | 0.00146 | 3666.6 | 2164.0 |
| NAB1 | NGFI-A binding protein 1 | [4664](https://www.ncbi.nlm.nih.gov/gene/?term=4664) | 1.6 | 0.00003 | 2556.6 | 1542.0 |
| CD86 | CD86 molecule | [942](https://www.ncbi.nlm.nih.gov/gene/?term=942) | 1.6 | 0.00096 | 1691.9 | 997.0 |
| SFXN5 | sideroflexin 5 | [94097](https://www.ncbi.nlm.nih.gov/gene/?term=94097) | 1.6 | 0.00014 | 504.6 | 301.4 |
| FEN1 | flap structure-specific endonuclease 1 | [2237](https://www.ncbi.nlm.nih.gov/gene/?term=2237) | 1.6 | 0.00657 | 389.6 | 222.9 |
| SHROOM1 | shroom family member 1 | [134549](https://www.ncbi.nlm.nih.gov/gene/?term=134549) | 1.6 | 0.04716 | 59.9 | 29.1 |
| SMC5-AS1 | SMC5 antisense RNA 1 (head to head) | [100507299](https://www.ncbi.nlm.nih.gov/gene/?term=100507299) | -1.6 | 0.03826 | 4.7 | 8.9 |
| COMMD4 | COMM domain containing 4 | [54939](https://www.ncbi.nlm.nih.gov/gene/?term=54939) | 1.6 | 0.00010 | 212.0 | 126.7 |
| DSE | dermatan sulfate epimerase | [29940](https://www.ncbi.nlm.nih.gov/gene/?term=29940) | 1.6 | 0.00026 | 2600.1 | 1550.2 |
| C11orf84 | chromosome 11 open reading frame 84 | [144097](https://www.ncbi.nlm.nih.gov/gene/?term=144097) | 1.6 | 0.00407 | 51.4 | 29.6 |
| PKDCC | protein kinase domain containing, cytoplasmic | [91461](https://www.ncbi.nlm.nih.gov/gene/?term=91461) | 1.6 | 0.05858 | 7.5 | 3.0 |
| PRELID1 | PRELI domain containing 1 | [27166](https://www.ncbi.nlm.nih.gov/gene/?term=27166) | 1.6 | 0.00022 | 3556.8 | 2118.8 |
| DYRK3 | dual specificity tyrosine phosphorylation regulated kinase 3 | [8444](https://www.ncbi.nlm.nih.gov/gene/?term=8444) | -1.6 | 0.03681 | 1488.7 | 2926.0 |
| LOC101927438 | uncharacterized LOC101927438 | [101927438](https://www.ncbi.nlm.nih.gov/gene/?term=101927438) | -1.6 | 0.03594 | 10.2 | 19.4 |
| CDHR1 | cadherin related family member 1 | [92211](https://www.ncbi.nlm.nih.gov/gene/?term=92211) | -1.6 | 0.04188 | 9.1 | 17.9 |
| SLC2A1-AS1 | SLC2A1 antisense RNA 1 | [440584](https://www.ncbi.nlm.nih.gov/gene/?term=440584) | -1.6 | 0.03095 | 91.4 | 173.0 |
| IRS2 | insulin receptor substrate 2 | [8660](https://www.ncbi.nlm.nih.gov/gene/?term=8660) | -1.6 | 0.00628 | 416.9 | 727.4 |
| SORT1 | sortilin 1 | [6272](https://www.ncbi.nlm.nih.gov/gene/?term=6272) | 1.6 | 0.00484 | 2220.8 | 1279.3 |
| GPLD1 | glycosylphosphatidylinositol specific phospholipase D1 | [2822](https://www.ncbi.nlm.nih.gov/gene/?term=2822) | -1.6 | 0.02678 | 302.4 | 567.4 |
| CPT2 | carnitine palmitoyltransferase 2 | [1376](https://www.ncbi.nlm.nih.gov/gene/?term=1376) | 1.6 | 0.00000 | 435.4 | 265.4 |
| CUEDC2 | CUE domain containing 2 | [79004](https://www.ncbi.nlm.nih.gov/gene/?term=79004) | 1.6 | 0.00000 | 408.1 | 247.6 |
| FBXO32 | F-box protein 32 | [114907](https://www.ncbi.nlm.nih.gov/gene/?term=114907) | -1.6 | 0.00740 | 126.3 | 222.7 |
| SFT2D1 | SFT2 domain containing 1 | [113402](https://www.ncbi.nlm.nih.gov/gene/?term=113402) | 1.6 | 0.00000 | 993.2 | 603.8 |
| ZHX3 | zinc fingers and homeoboxes 3 | [23051](https://www.ncbi.nlm.nih.gov/gene/?term=23051) | -1.6 | 0.00030 | 102.9 | 173.4 |
| MALAT1 |  | [378938](https://www.ncbi.nlm.nih.gov/gene/?term=378938) | -1.6 | 0.01508 | 67602.8 | 121853.5 |
| VAMP2 | vesicle associated membrane protein 2 | [6844](https://www.ncbi.nlm.nih.gov/gene/?term=6844) | -1.6 | 0.00943 | 103.3 | 183.9 |
| ATG13 | autophagy related 13 | [9776](https://www.ncbi.nlm.nih.gov/gene/?term=9776) | -1.6 | 0.00006 | 739.6 | 1227.3 |
| NECTIN2 | nectin cell adhesion molecule 2 | [5819](https://www.ncbi.nlm.nih.gov/gene/?term=5819) | 1.6 | 0.05734 | 221.2 | 73.2 |
| ZC3H10 | zinc finger CCCH-type containing 10 | [84872](https://www.ncbi.nlm.nih.gov/gene/?term=84872) | 1.6 | 0.00001 | 307.7 | 186.0 |
| ANKRD36 | ankyrin repeat domain 36 | [375248](https://www.ncbi.nlm.nih.gov/gene/?term=375248) | -1.6 | 0.00005 | 781.6 | 1303.2 |
| RNF149 | ring finger protein 149 | [284996](https://www.ncbi.nlm.nih.gov/gene/?term=284996) | 1.6 | 0.00494 | 18681.4 | 10867.7 |
| CCDC159 | coiled-coil domain containing 159 | [126075](https://www.ncbi.nlm.nih.gov/gene/?term=126075) | 1.6 | 0.00015 | 148.9 | 89.5 |
| NHSL2 | NHS like 2 | [340527](https://www.ncbi.nlm.nih.gov/gene/?term=340527) | -1.6 | 0.00950 | 1902.9 | 3350.6 |
| CNOT3 | CCR4-NOT transcription complex subunit 3 | [4849](https://www.ncbi.nlm.nih.gov/gene/?term=4849) | -1.6 | 0.04515 | 5.1 | 11.1 |
| BBS5 | Bardet-Biedl syndrome 5 | [129880](https://www.ncbi.nlm.nih.gov/gene/?term=129880) | -1.6 | 0.01657 | 11.4 | 20.5 |
| PSMB10 | proteasome subunit beta 10 | [5699](https://www.ncbi.nlm.nih.gov/gene/?term=5699) | 1.6 | 0.00008 | 3011.3 | 1813.0 |
| CAPZA1 | capping actin protein of muscle Z-line alpha subunit 1 | [829](https://www.ncbi.nlm.nih.gov/gene/?term=829) | 1.6 | 0.00010 | 13748.7 | 8284.5 |
| LOC107987245 |  | [107987245](https://www.ncbi.nlm.nih.gov/gene/?term=107987245) | -1.6 | 0.00786 | 226.8 | 398.4 |
| RNF114 | ring finger protein 114 | [55905](https://www.ncbi.nlm.nih.gov/gene/?term=55905) | 1.6 | 0.00000 | 2021.5 | 1234.5 |
| ARNTL2 | aryl hydrocarbon receptor nuclear translocator like 2 | [56938](https://www.ncbi.nlm.nih.gov/gene/?term=56938) | 1.6 | 0.00101 | 176.1 | 104.5 |
| LOC107984443 |  | [107984443](https://www.ncbi.nlm.nih.gov/gene/?term=107984443) | 1.6 | 0.06247 | 8.0 | 3.2 |
| WDR31 | WD repeat domain 31 | [114987](https://www.ncbi.nlm.nih.gov/gene/?term=114987) | -1.6 | 0.01325 | 112.9 | 198.8 |
| ARRDC1 | arrestin domain containing 1 | [92714](https://www.ncbi.nlm.nih.gov/gene/?term=92714) | 1.6 | 0.00020 | 599.7 | 359.0 |
| MIR5692C2 | microRNA 5692c-2 | [100847017](https://www.ncbi.nlm.nih.gov/gene/?term=100847017) | -1.6 | 0.04363 | 9.5 | 19.6 |
| BAG4 | BCL2 associated athanogene 4 | [9530](https://www.ncbi.nlm.nih.gov/gene/?term=9530) | -1.6 | 0.00069 | 492.0 | 827.5 |
| INHBB | inhibin beta B | [3625](https://www.ncbi.nlm.nih.gov/gene/?term=3625) | 1.6 | 0.05553 | 15.2 | 7.3 |
| C1orf54 | chromosome 1 open reading frame 54 | [79630](https://www.ncbi.nlm.nih.gov/gene/?term=79630) | 1.6 | 0.02249 | 39.3 | 21.6 |
| ENG | endoglin | [2022](https://www.ncbi.nlm.nih.gov/gene/?term=2022) | -1.6 | 0.05040 | 17.1 | 37.0 |
| PTPRN | protein tyrosine phosphatase, receptor type N | [5798](https://www.ncbi.nlm.nih.gov/gene/?term=5798) | -1.6 | 0.05592 | 27.3 | 62.7 |
| RASAL2 | RAS protein activator like 2 | [9462](https://www.ncbi.nlm.nih.gov/gene/?term=9462) | -1.6 | 0.01499 | 19.3 | 34.5 |
| LOC105372989 |  | [105372989](https://www.ncbi.nlm.nih.gov/gene/?term=105372989) | -1.6 | 0.02931 | 13.1 | 25.5 |
| ZNF84 | zinc finger protein 84 | [7637](https://www.ncbi.nlm.nih.gov/gene/?term=7637) | -1.6 | 0.00002 | 404.9 | 670.6 |
| LOC102724389 |  | [102724389](https://www.ncbi.nlm.nih.gov/gene/?term=102724389) | 1.6 | 0.06247 | 10.5 | 4.6 |
| LRRC69 | leucine rich repeat containing 69 | [100130742](https://www.ncbi.nlm.nih.gov/gene/?term=100130742) | -1.6 | 0.00245 | 29.7 | 50.8 |
| HJURP | Holliday junction recognition protein | [55355](https://www.ncbi.nlm.nih.gov/gene/?term=55355) | 1.6 | 0.01281 | 128.3 | 72.3 |
| ZNF540 | zinc finger protein 540 | [163255](https://www.ncbi.nlm.nih.gov/gene/?term=163255) | -1.6 | 0.00108 | 110.5 | 186.1 |
| LDHA | lactate dehydrogenase A | [3939](https://www.ncbi.nlm.nih.gov/gene/?term=3939) | 1.6 | 0.00018 | 3689.6 | 2218.9 |
| KLF12 | Kruppel-like factor 12 | [11278](https://www.ncbi.nlm.nih.gov/gene/?term=11278) | -1.6 | 0.00276 | 1579.9 | 2693.2 |
| LOC100507670 | uncharacterized LOC100507670 | [100507670](https://www.ncbi.nlm.nih.gov/gene/?term=100507670) | 1.6 | 0.02356 | 18.4 | 10.1 |
| MCF2L2 | MCF.2 cell line derived transforming sequence-like 2 | [23101](https://www.ncbi.nlm.nih.gov/gene/?term=23101) | -1.6 | 0.00103 | 45.3 | 76.0 |
| ANK3 | ankyrin 3, node of Ranvier (ankyrin G) | [288](https://www.ncbi.nlm.nih.gov/gene/?term=288) | -1.6 | 0.01201 | 259.5 | 461.2 |
| CYBA | cytochrome b-245 alpha chain | [1535](https://www.ncbi.nlm.nih.gov/gene/?term=1535) | 1.6 | 0.00013 | 7644.2 | 4595.3 |
| ABI2 | abl-interactor 2 | [10152](https://www.ncbi.nlm.nih.gov/gene/?term=10152) | -1.6 | 0.00005 | 298.0 | 492.2 |
| TAB2 | TGF-beta activated kinase 1/MAP3K7 binding protein 2 | [23118](https://www.ncbi.nlm.nih.gov/gene/?term=23118) | -1.6 | 0.00040 | 4607.2 | 7699.2 |
| ZNF276 | zinc finger protein 276 | [92822](https://www.ncbi.nlm.nih.gov/gene/?term=92822) | 1.6 | 0.00028 | 2378.1 | 1426.3 |
| WSB2 | WD repeat and SOCS box containing 2 | [55884](https://www.ncbi.nlm.nih.gov/gene/?term=55884) | 1.6 | 0.00012 | 1708.4 | 1033.3 |
| CCNK | cyclin K | [8812](https://www.ncbi.nlm.nih.gov/gene/?term=8812) | -1.6 | 0.00248 | 355.6 | 606.2 |
| FAM45BP | family with sequence similarity 45 member B, pseudogene | [55855](https://www.ncbi.nlm.nih.gov/gene/?term=55855) | 1.6 | 0.01051 | 32.3 | 18.4 |
| MYO1C | myosin IC | [4641](https://www.ncbi.nlm.nih.gov/gene/?term=4641) | -1.6 | 0.00555 | 140.7 | 243.7 |
| SLC25A28 | solute carrier family 25 member 28 | [81894](https://www.ncbi.nlm.nih.gov/gene/?term=81894) | 1.6 | 0.00059 | 895.0 | 533.8 |
| CEP290 | centrosomal protein 290 | [80184](https://www.ncbi.nlm.nih.gov/gene/?term=80184) | -1.6 | 0.00000 | 671.6 | 1102.1 |
| DBI | diazepam binding inhibitor (GABA receptor modulator, acyl-CoA binding protein) | [1622](https://www.ncbi.nlm.nih.gov/gene/?term=1622) | 1.6 | 0.00003 | 1072.6 | 652.1 |
| LOC105377347 |  | [105377347](https://www.ncbi.nlm.nih.gov/gene/?term=105377347) | -1.6 | 0.03750 | 13.5 | 26.8 |
| IRF5 | interferon regulatory factor 5 | [3663](https://www.ncbi.nlm.nih.gov/gene/?term=3663) | 1.6 | 0.00194 | 703.1 | 414.9 |
| HECTD4 | HECT domain E3 ubiquitin protein ligase 4 | [283450](https://www.ncbi.nlm.nih.gov/gene/?term=283450) | -1.6 | 0.01876 | 7303.2 | 13188.5 |
| SLC26A6 | solute carrier family 26 member 6 | [65010](https://www.ncbi.nlm.nih.gov/gene/?term=65010) | 1.6 | 0.00009 | 176.2 | 105.8 |
| YES1 | YES proto-oncogene 1, Src family tyrosine kinase | [7525](https://www.ncbi.nlm.nih.gov/gene/?term=7525) | -1.6 | 0.00546 | 293.8 | 506.1 |
| HIST1H2BK | histone cluster 1, H2bk | [85236](https://www.ncbi.nlm.nih.gov/gene/?term=85236) | 1.6 | 0.00042 | 4378.3 | 2626.3 |
| EFCAB5 | EF-hand calcium binding domain 5 | [374786](https://www.ncbi.nlm.nih.gov/gene/?term=374786) | -1.6 | 0.01136 | 19.7 | 34.1 |
| HSP90AA2P | heat shock protein 90kDa alpha family class A member 2, pseudogene | [3324](https://www.ncbi.nlm.nih.gov/gene/?term=3324) | 1.6 | 0.05193 | 9.5 | 4.7 |
| ZBTB7B | zinc finger and BTB domain containing 7B | [51043](https://www.ncbi.nlm.nih.gov/gene/?term=51043) | 1.6 | 0.00012 | 2028.8 | 1225.3 |
| LY86 | lymphocyte antigen 86 | [9450](https://www.ncbi.nlm.nih.gov/gene/?term=9450) | 1.6 | 0.00208 | 847.2 | 499.2 |
| TBC1D19 | TBC1 domain family member 19 | [55296](https://www.ncbi.nlm.nih.gov/gene/?term=55296) | -1.6 | 0.00005 | 132.6 | 220.4 |
| RNU6ATAC | RNA, U6atac small nuclear (U12-dependent splicing) | [100151684](https://www.ncbi.nlm.nih.gov/gene/?term=100151684) | 1.6 | 0.03904 | 20.0 | 10.1 |
| UBA7 | ubiquitin like modifier activating enzyme 7 | [7318](https://www.ncbi.nlm.nih.gov/gene/?term=7318) | 1.6 | 0.00002 | 1624.6 | 988.2 |
| EMP3 | epithelial membrane protein 3 | [2014](https://www.ncbi.nlm.nih.gov/gene/?term=2014) | 1.6 | 0.00002 | 2527.9 | 1536.5 |
| ZNF460 | zinc finger protein 460 | [10794](https://www.ncbi.nlm.nih.gov/gene/?term=10794) | -1.6 | 0.00015 | 1426.5 | 2374.0 |
| PGLYRP2 | peptidoglycan recognition protein 2 | [114770](https://www.ncbi.nlm.nih.gov/gene/?term=114770) | 1.6 | 0.04530 | 8.7 | 4.6 |
| MIR8075 | microRNA 8075 | [102465874](https://www.ncbi.nlm.nih.gov/gene/?term=102465874) | -1.6 | 0.05935 | 6.8 | 16.0 |
| C15orf39 | chromosome 15 open reading frame 39 | [56905](https://www.ncbi.nlm.nih.gov/gene/?term=56905) | -1.6 | 0.01862 | 572.7 | 1031.9 |
| SNORA38B | small nucleolar RNA, H/ACA box 38B | [100124536](https://www.ncbi.nlm.nih.gov/gene/?term=100124536) | 1.6 | 0.05785 | 6.8 | 2.9 |
| KIAA1462 | KIAA1462 | [57608](https://www.ncbi.nlm.nih.gov/gene/?term=57608) | -1.6 | 0.05173 | 9.4 | 19.5 |
| LOC102723811 |  | [102723811](https://www.ncbi.nlm.nih.gov/gene/?term=102723811) | 1.6 | 0.05711 | 4.7 | 2.5 |
| GPR108 | G protein-coupled receptor 108 | [56927](https://www.ncbi.nlm.nih.gov/gene/?term=56927) | 1.6 | 0.00000 | 801.1 | 495.4 |
| ZNF267 | zinc finger protein 267 | [10308](https://www.ncbi.nlm.nih.gov/gene/?term=10308) | 1.6 | 0.00120 | 5032.7 | 2993.3 |
| GLIS3 | GLIS family zinc finger 3 | [169792](https://www.ncbi.nlm.nih.gov/gene/?term=169792) | -1.6 | 0.05536 | 9.2 | 20.1 |
| ECSIT | ECSIT signalling integrator | [51295](https://www.ncbi.nlm.nih.gov/gene/?term=51295) | -1.6 | 0.01433 | 309.8 | 547.4 |
| LOC107985675 |  | [107985675](https://www.ncbi.nlm.nih.gov/gene/?term=107985675) | -1.6 | 0.01117 | 16.2 | 28.9 |
| SNORA73A | small nucleolar RNA, H/ACA box 73A | [6080](https://www.ncbi.nlm.nih.gov/gene/?term=6080) | 1.6 | 0.01018 | 3367.3 | 1923.7 |
| ABCF2 | ATP binding cassette subfamily F member 2 | [10061](https://www.ncbi.nlm.nih.gov/gene/?term=10061) | -1.6 | 0.00004 | 268.3 | 441.5 |
| LTA4H | leukotriene A4 hydrolase | [4048](https://www.ncbi.nlm.nih.gov/gene/?term=4048) | 1.6 | 0.00672 | 6684.2 | 3893.6 |
| LOC105373456 |  | [105373456](https://www.ncbi.nlm.nih.gov/gene/?term=105373456) | -1.6 | 0.01152 | 32.6 | 58.2 |
| LOC107984345 |  | [107984345](https://www.ncbi.nlm.nih.gov/gene/?term=107984345) | 1.6 | 0.05428 | 28.2 | 13.6 |
| LOC107984913 |  | [107984913](https://www.ncbi.nlm.nih.gov/gene/?term=107984913) | 1.6 | 0.01705 | 23.7 | 13.1 |
| MICA | MHC class I polypeptide-related sequence A | [100507436](https://www.ncbi.nlm.nih.gov/gene/?term=100507436) | 1.6 | 0.03333 | 171.0 | 91.0 |
| MIR223 | microRNA 223 | [407008](https://www.ncbi.nlm.nih.gov/gene/?term=407008) | 1.6 | 0.00886 | 724.7 | 416.7 |
| SCAF4 | SR-related CTD associated factor 4 | [57466](https://www.ncbi.nlm.nih.gov/gene/?term=57466) | -1.6 | 0.00117 | 295.7 | 496.6 |
| RRAGD | Ras related GTP binding D | [58528](https://www.ncbi.nlm.nih.gov/gene/?term=58528) | 1.6 | 0.00240 | 1406.7 | 831.6 |
| RCBTB2P1 | RCC1 and BTB domain containing protein 2 pseudogene 1 | [100130253](https://www.ncbi.nlm.nih.gov/gene/?term=100130253) | -1.6 | 0.06290 | 2.3 | 5.9 |
| LOC100133985 | uncharacterized LOC100133985 | [100133985](https://www.ncbi.nlm.nih.gov/gene/?term=100133985) | 1.6 | 0.05056 | 10.3 | 5.3 |
| HTRA2 | HtrA serine peptidase 2 | [27429](https://www.ncbi.nlm.nih.gov/gene/?term=27429) | 1.6 | 0.00000 | 699.6 | 428.5 |
| CLK2 | CDC like kinase 2 | [1196](https://www.ncbi.nlm.nih.gov/gene/?term=1196) | 1.6 | 0.00897 | 18.3 | 10.5 |
| FRMD4B | FERM domain containing 4B | [23150](https://www.ncbi.nlm.nih.gov/gene/?term=23150) | 1.6 | 0.00232 | 1284.5 | 761.0 |
| MPG | N-methylpurine DNA glycosylase | [4350](https://www.ncbi.nlm.nih.gov/gene/?term=4350) | 1.6 | 0.00006 | 570.8 | 346.6 |
| ZNF678 | zinc finger protein 678 | [339500](https://www.ncbi.nlm.nih.gov/gene/?term=339500) | -1.6 | 0.00004 | 363.0 | 596.3 |
| ZNF692 | zinc finger protein 692 | [55657](https://www.ncbi.nlm.nih.gov/gene/?term=55657) | 1.6 | 0.00369 | 290.9 | 170.8 |
| AGPAT3 | 1-acylglycerol-3-phosphate O-acyltransferase 3 | [56894](https://www.ncbi.nlm.nih.gov/gene/?term=56894) | -1.6 | 0.00622 | 1395.9 | 2406.4 |
| HECA | hdc homolog, cell cycle regulator | [51696](https://www.ncbi.nlm.nih.gov/gene/?term=51696) | -1.6 | 0.00068 | 9715.5 | 16180.1 |
| DUSP5 | dual specificity phosphatase 5 | [1847](https://www.ncbi.nlm.nih.gov/gene/?term=1847) | 1.6 | 0.00485 | 488.9 | 286.3 |
| LOC105370567 |  | [105370567](https://www.ncbi.nlm.nih.gov/gene/?term=105370567) | -1.6 | 0.02056 | 77.3 | 141.1 |
| LOC101928383 |  | [101928383](https://www.ncbi.nlm.nih.gov/gene/?term=101928383) | -1.6 | 0.00275 | 101.4 | 170.6 |
| TBC1D22B | TBC1 domain family member 22B | [55633](https://www.ncbi.nlm.nih.gov/gene/?term=55633) | -1.6 | 0.03013 | 1807.8 | 3345.7 |
| SPNS3 | spinster homolog 3 (Drosophila) | [201305](https://www.ncbi.nlm.nih.gov/gene/?term=201305) | -1.6 | 0.02740 | 53.4 | 98.0 |
| FAM109B | family with sequence similarity 109 member B | [150368](https://www.ncbi.nlm.nih.gov/gene/?term=150368) | 1.6 | 0.01768 | 19.0 | 10.3 |
| LOC102724008 |  | [102724008](https://www.ncbi.nlm.nih.gov/gene/?term=102724008) | 1.6 | 0.04990 | 14.6 | 7.4 |
| CDC42EP3 | CDC42 effector protein 3 | [10602](https://www.ncbi.nlm.nih.gov/gene/?term=10602) | 1.6 | 0.00483 | 4737.6 | 2791.1 |
| LOC102724881 |  | [102724881](https://www.ncbi.nlm.nih.gov/gene/?term=102724881) | 1.6 | 0.03446 | 20.9 | 11.3 |
| GNB4 | G protein subunit beta 4 | [59345](https://www.ncbi.nlm.nih.gov/gene/?term=59345) | 1.6 | 0.00103 | 4883.7 | 2926.2 |
| ACACB | acetyl-CoA carboxylase beta | [32](https://www.ncbi.nlm.nih.gov/gene/?term=32) | -1.6 | 0.00202 | 96.0 | 162.5 |
| TRBV9 | T cell receptor beta variable 9 | [28586](https://www.ncbi.nlm.nih.gov/gene/?term=28586) | 1.6 | 0.02502 | 43.1 | 23.8 |
| NUBPL | nucleotide binding protein like | [80224](https://www.ncbi.nlm.nih.gov/gene/?term=80224) | -1.6 | 0.00111 | 121.2 | 203.7 |
| SF3B6 | splicing factor 3b subunit 6 | [51639](https://www.ncbi.nlm.nih.gov/gene/?term=51639) | 1.6 | 0.00021 | 1369.1 | 832.9 |
| TPST2 | tyrosylprotein sulfotransferase 2 | [8459](https://www.ncbi.nlm.nih.gov/gene/?term=8459) | 1.6 | 0.00007 | 2653.8 | 1627.4 |
| DNAJA1 | DnaJ heat shock protein family (Hsp40) member A1 | [3301](https://www.ncbi.nlm.nih.gov/gene/?term=3301) | 1.6 | 0.00377 | 8315.9 | 4899.9 |
| ZNF385B | zinc finger protein 385B | [151126](https://www.ncbi.nlm.nih.gov/gene/?term=151126) | -1.6 | 0.06054 | 6.4 | 16.0 |
| TMX1 | thioredoxin related transmembrane protein 1 | [81542](https://www.ncbi.nlm.nih.gov/gene/?term=81542) | 1.6 | 0.00000 | 2005.6 | 1237.9 |
| PPP2R5E | protein phosphatase 2 regulatory subunit B', epsilon | [5529](https://www.ncbi.nlm.nih.gov/gene/?term=5529) | -1.6 | 0.00045 | 624.9 | 1038.1 |
| LRRC24 | leucine rich repeat containing 24 | [441381](https://www.ncbi.nlm.nih.gov/gene/?term=441381) | 1.6 | 0.02235 | 26.1 | 14.3 |
| ZNF37BP | zinc finger protein 37B, pseudogene | [100129482](https://www.ncbi.nlm.nih.gov/gene/?term=100129482) | -1.6 | 0.00001 | 633.9 | 1033.0 |
| FAM45A | family with sequence similarity 45 member A | [404636](https://www.ncbi.nlm.nih.gov/gene/?term=404636) | 1.6 | 0.00130 | 1771.8 | 1060.2 |
| EXOC6B | exocyst complex component 6B | [23233](https://www.ncbi.nlm.nih.gov/gene/?term=23233) | -1.6 | 0.03967 | 747.9 | 1437.6 |
| GRB2 | growth factor receptor bound protein 2 | [2885](https://www.ncbi.nlm.nih.gov/gene/?term=2885) | 1.6 | 0.00001 | 8717.7 | 5357.0 |
| SDF2 | stromal cell derived factor 2 | [6388](https://www.ncbi.nlm.nih.gov/gene/?term=6388) | 1.6 | 0.00024 | 1151.6 | 695.9 |
| FTH1P2 | ferritin, heavy polypeptide 1 pseudogene 2 | [2497](https://www.ncbi.nlm.nih.gov/gene/?term=2497) | -1.6 | 0.04290 | 13.3 | 25.4 |
| LOC105378669 |  | [105378669](https://www.ncbi.nlm.nih.gov/gene/?term=105378669) | 1.6 | 0.00790 | 28.0 | 16.6 |
| AKAP12 | A-kinase anchoring protein 12 | [9590](https://www.ncbi.nlm.nih.gov/gene/?term=9590) | -1.6 | 0.01819 | 118.2 | 210.1 |
| VAMP3 | vesicle associated membrane protein 3 | [9341](https://www.ncbi.nlm.nih.gov/gene/?term=9341) | 1.6 | 0.00083 | 2382.7 | 1433.0 |
| RAPGEF3 | Rap guanine nucleotide exchange factor 3 | [10411](https://www.ncbi.nlm.nih.gov/gene/?term=10411) | 1.6 | 0.06827 | 8.0 | 3.2 |
| RFX1 | regulatory factor X1 | [5989](https://www.ncbi.nlm.nih.gov/gene/?term=5989) | -1.6 | 0.02197 | 55.6 | 101.2 |
| TAS2R5 | taste 2 receptor member 5 | [54429](https://www.ncbi.nlm.nih.gov/gene/?term=54429) | -1.6 | 0.06009 | 6.0 | 13.9 |
| LOC105377291 |  | [105377291](https://www.ncbi.nlm.nih.gov/gene/?term=105377291) | -1.6 | 0.01754 | 25.3 | 45.9 |
| ARID3A | AT-rich interaction domain 3A | [1820](https://www.ncbi.nlm.nih.gov/gene/?term=1820) | 1.6 | 0.00367 | 1016.9 | 601.2 |
| C1GALT1C1 | C1GALT1 specific chaperone 1 | [29071](https://www.ncbi.nlm.nih.gov/gene/?term=29071) | 1.6 | 0.00035 | 718.6 | 434.7 |
| ZNF778 | zinc finger protein 778 | [197320](https://www.ncbi.nlm.nih.gov/gene/?term=197320) | -1.6 | 0.00185 | 368.0 | 615.3 |
| GGT5 | gamma-glutamyltransferase 5 | [2687](https://www.ncbi.nlm.nih.gov/gene/?term=2687) | -1.6 | 0.06179 | 5.2 | 13.5 |
| EPB41 | erythrocyte membrane protein band 4.1 | [2035](https://www.ncbi.nlm.nih.gov/gene/?term=2035) | -1.6 | 0.04188 | 224079.8 | 435866.9 |
| RTN2 | reticulon 2 | [6253](https://www.ncbi.nlm.nih.gov/gene/?term=6253) | 1.6 | 0.00415 | 76.2 | 45.4 |
| RECQL4 | RecQ like helicase 4 | [9401](https://www.ncbi.nlm.nih.gov/gene/?term=9401) | 1.6 | 0.02601 | 50.9 | 28.2 |
| PPIF | peptidylprolyl isomerase F | [10105](https://www.ncbi.nlm.nih.gov/gene/?term=10105) | 1.6 | 0.00930 | 835.2 | 485.2 |
| PPT1 | palmitoyl-protein thioesterase 1 | [5538](https://www.ncbi.nlm.nih.gov/gene/?term=5538) | 1.6 | 0.00389 | 5478.2 | 3228.6 |
| HPCAL4 | hippocalcin like 4 | [51440](https://www.ncbi.nlm.nih.gov/gene/?term=51440) | -1.6 | 0.01696 | 36.2 | 64.7 |
| AMDHD2 | amidohydrolase domain containing 2 | [51005](https://www.ncbi.nlm.nih.gov/gene/?term=51005) | 1.6 | 0.00140 | 479.1 | 286.7 |
| CCND3 | cyclin D3 | [896](https://www.ncbi.nlm.nih.gov/gene/?term=896) | 1.6 | 0.00001 | 3272.4 | 2014.7 |
| LOC107984631 |  | [107984631](https://www.ncbi.nlm.nih.gov/gene/?term=107984631) | -1.6 | 0.06172 | 5.2 | 12.2 |
| TRNL2 |  | [4568](https://www.ncbi.nlm.nih.gov/gene/?term=4568) | -1.6 | 0.05491 | 6.3 | 12.9 |
| LOC646214 | p21 protein (Cdc42/Rac)-activated kinase 2 pseudogene | [646214](https://www.ncbi.nlm.nih.gov/gene/?term=646214) | -1.6 | 0.06036 | 7.8 | 20.3 |
| ICE1 | interactor of little elongation complex ELL subunit 1 | [23379](https://www.ncbi.nlm.nih.gov/gene/?term=23379) | -1.6 | 0.00114 | 800.8 | 1335.0 |
| DSP | desmoplakin | [1832](https://www.ncbi.nlm.nih.gov/gene/?term=1832) | -1.6 | 0.06027 | 95.1 | 221.9 |
| LOC101928762 |  | [101928762](https://www.ncbi.nlm.nih.gov/gene/?term=101928762) | 1.6 | 0.00713 | 94.5 | 55.2 |
| NUP88 | nucleoporin 88 | [4927](https://www.ncbi.nlm.nih.gov/gene/?term=4927) | -1.6 | 0.00002 | 681.2 | 1106.3 |
| HIST1H2AI | histone cluster 1, H2ai | [8329](https://www.ncbi.nlm.nih.gov/gene/?term=8329) | 1.6 | 0.01291 | 815.6 | 467.9 |
| ARHGEF12 | Rho guanine nucleotide exchange factor 12 | [23365](https://www.ncbi.nlm.nih.gov/gene/?term=23365) | -1.6 | 0.03826 | 40372.5 | 81715.2 |
| MIR181A1HG | MIR181A1 host gene | [100131234](https://www.ncbi.nlm.nih.gov/gene/?term=100131234) | -1.6 | 0.01662 | 22.4 | 41.0 |
| ADHFE1 | alcohol dehydrogenase, iron containing 1 | [137872](https://www.ncbi.nlm.nih.gov/gene/?term=137872) | -1.6 | 0.01374 | 29.2 | 51.6 |
| ITGB2-AS1 | ITGB2 antisense RNA 1 | [100505746](https://www.ncbi.nlm.nih.gov/gene/?term=100505746) | 1.6 | 0.00217 | 527.9 | 315.9 |
| AHI1 | Abelson helper integration site 1 | [54806](https://www.ncbi.nlm.nih.gov/gene/?term=54806) | -1.6 | 0.00023 | 210.0 | 344.1 |
| CLPTM1 | cleft lip and palate associated transmembrane protein 1 | [1209](https://www.ncbi.nlm.nih.gov/gene/?term=1209) | -1.6 | 0.00092 | 335.7 | 558.5 |
| ACSL3 | acyl-CoA synthetase long-chain family member 3 | [2181](https://www.ncbi.nlm.nih.gov/gene/?term=2181) | 1.6 | 0.00076 | 1980.8 | 1193.0 |
| ASPH | aspartate beta-hydroxylase | [444](https://www.ncbi.nlm.nih.gov/gene/?term=444) | 1.6 | 0.00609 | 2346.1 | 1378.0 |
| TRNR |  | [4573](https://www.ncbi.nlm.nih.gov/gene/?term=4573) | -1.6 | 0.01249 | 68.6 | 118.4 |
| FAM157C | family with sequence similarity 157 member C | [100996541](https://www.ncbi.nlm.nih.gov/gene/?term=100996541) | 1.6 | 0.03111 | 380.3 | 206.9 |
| SLC31A2 | solute carrier family 31 member 2 | [1318](https://www.ncbi.nlm.nih.gov/gene/?term=1318) | 1.6 | 0.00502 | 3084.8 | 1817.6 |
| ZNF204P | zinc finger protein 204, pseudogene | [7754](https://www.ncbi.nlm.nih.gov/gene/?term=7754) | -1.6 | 0.02227 | 45.1 | 80.9 |
| RN7SL674P | RNA, 7SL, cytoplasmic 674, pseudogene | [106479468](https://www.ncbi.nlm.nih.gov/gene/?term=106479468) | -1.6 | 0.03705 | 38.3 | 70.6 |
| DIAPH1 | diaphanous related formin 1 | [1729](https://www.ncbi.nlm.nih.gov/gene/?term=1729) | -1.6 | 0.01138 | 2059.4 | 3585.9 |
| SWSAP1 | SWIM-type zinc finger 7 associated protein 1 | [126074](https://www.ncbi.nlm.nih.gov/gene/?term=126074) | 1.6 | 0.00061 | 55.6 | 33.9 |
| TRMT1 | tRNA methyltransferase 1 | [55621](https://www.ncbi.nlm.nih.gov/gene/?term=55621) | 1.6 | 0.00144 | 434.3 | 260.1 |
| DIP2A-IT1 | DIP2A intronic transcript 1 | [100862692](https://www.ncbi.nlm.nih.gov/gene/?term=100862692) | -1.6 | 0.06227 | 4.3 | 11.6 |
| TXNDC17 | thioredoxin domain containing 17 | [84817](https://www.ncbi.nlm.nih.gov/gene/?term=84817) | 1.6 | 0.00006 | 468.1 | 287.2 |
| LOC100506844 | uncharacterized LOC100506844 | [100506844](https://www.ncbi.nlm.nih.gov/gene/?term=100506844) | 1.6 | 0.01031 | 58.3 | 34.0 |
| MIR1304 | microRNA 1304 | [100302240](https://www.ncbi.nlm.nih.gov/gene/?term=100302240) | -1.6 | 0.01398 | 11.3 | 20.6 |
| MAGIX | MAGI family member, X-linked | [79917](https://www.ncbi.nlm.nih.gov/gene/?term=79917) | 1.6 | 0.00619 | 29.3 | 17.0 |
| LOC105371224 |  | [105371224](https://www.ncbi.nlm.nih.gov/gene/?term=105371224) | -1.6 | 0.02803 | 275.0 | 502.8 |
| LOC107985033 |  | [107985033](https://www.ncbi.nlm.nih.gov/gene/?term=107985033) | -1.6 | 0.06307 | 4.6 | 10.9 |
| SMARCC2 | SWI/SNF related, matrix associated, actin dependent regulator of chromatin subfamily c member 2 | [6601](https://www.ncbi.nlm.nih.gov/gene/?term=6601) | -1.6 | 0.00077 | 707.9 | 1172.3 |
| ANKRD18A | ankyrin repeat domain 18A | [253650](https://www.ncbi.nlm.nih.gov/gene/?term=253650) | -1.6 | 0.01561 | 42.3 | 75.0 |
| MUS81 | MUS81 structure-specific endonuclease subunit | [80198](https://www.ncbi.nlm.nih.gov/gene/?term=80198) | 1.6 | 0.00004 | 444.4 | 272.6 |
| ANKAR | ankyrin and armadillo repeat containing | [150709](https://www.ncbi.nlm.nih.gov/gene/?term=150709) | -1.6 | 0.00001 | 215.9 | 348.9 |
| LOC613266 | uncharacterized LOC613266 | [613266](https://www.ncbi.nlm.nih.gov/gene/?term=613266) | -1.6 | 0.03737 | 21.8 | 40.9 |
| LOC105370571 |  | [105370571](https://www.ncbi.nlm.nih.gov/gene/?term=105370571) | 1.6 | 0.05922 | 9.6 | 4.5 |
| NFKB2 | nuclear factor of kappa light polypeptide gene enhancer in B-cells 2 | [4791](https://www.ncbi.nlm.nih.gov/gene/?term=4791) | 1.6 | 0.00040 | 1200.0 | 731.2 |
| TNFRSF8 | tumor necrosis factor receptor superfamily member 8 | [943](https://www.ncbi.nlm.nih.gov/gene/?term=943) | 1.6 | 0.00492 | 188.6 | 112.1 |
| LOC107984921 |  | [107984921](https://www.ncbi.nlm.nih.gov/gene/?term=107984921) | -1.6 | 0.01642 | 48.6 | 86.7 |
| LOC105376834 |  | [105376834](https://www.ncbi.nlm.nih.gov/gene/?term=105376834) | 1.6 | 0.04401 | 25.7 | 13.5 |
| LSP1 | lymphocyte-specific protein 1 | [4046](https://www.ncbi.nlm.nih.gov/gene/?term=4046) | 1.6 | 0.00194 | 6874.5 | 4134.3 |
| TMEM70 | transmembrane protein 70 | [54968](https://www.ncbi.nlm.nih.gov/gene/?term=54968) | 1.6 | 0.00053 | 539.6 | 328.6 |
| CBX3P2 | chromobox 3 pseudogene 2 | [645158](https://www.ncbi.nlm.nih.gov/gene/?term=645158) | 1.6 | 0.00017 | 89.8 | 55.0 |
| DCAF10 | DDB1 and CUL4 associated factor 10 | [79269](https://www.ncbi.nlm.nih.gov/gene/?term=79269) | -1.6 | 0.02525 | 2832.1 | 5089.9 |
| GCLC | glutamate-cysteine ligase catalytic subunit | [2729](https://www.ncbi.nlm.nih.gov/gene/?term=2729) | -1.6 | 0.04131 | 4501.4 | 8576.3 |
| PFKFB1 | 6-phosphofructo-2-kinase/fructose-2,6-biphosphatase 1 | [5207](https://www.ncbi.nlm.nih.gov/gene/?term=5207) | -1.6 | 0.03579 | 19.0 | 34.5 |
| CARS | cysteinyl-tRNA synthetase | [833](https://www.ncbi.nlm.nih.gov/gene/?term=833) | 1.6 | 0.00010 | 103.0 | 63.2 |
| DVL1 | dishevelled segment polarity protein 1 | [1855](https://www.ncbi.nlm.nih.gov/gene/?term=1855) | 1.6 | 0.00121 | 478.2 | 287.7 |
| LOC100130370 | uncharacterized LOC100130370 | [100130370](https://www.ncbi.nlm.nih.gov/gene/?term=100130370) | 1.6 | 0.02076 | 57.6 | 32.4 |
| SCN3A | sodium voltage-gated channel alpha subunit 3 | [6328](https://www.ncbi.nlm.nih.gov/gene/?term=6328) | -1.6 | 0.03531 | 130.4 | 243.7 |
| LOC107984546 |  | [107984546](https://www.ncbi.nlm.nih.gov/gene/?term=107984546) | -1.6 | 0.05591 | 18.1 | 38.0 |
| MIAT | myocardial infarction associated transcript (non-protein coding) | [440823](https://www.ncbi.nlm.nih.gov/gene/?term=440823) | 1.6 | 0.01856 | 717.6 | 407.9 |
| P2RX1 | purinergic receptor P2X 1 | [5023](https://www.ncbi.nlm.nih.gov/gene/?term=5023) | 1.6 | 0.00866 | 936.7 | 548.7 |
| MCMBP | minichromosome maintenance complex binding protein | [79892](https://www.ncbi.nlm.nih.gov/gene/?term=79892) | 1.6 | 0.00000 | 3671.5 | 2281.2 |
| RAB44 | RAB44, member RAS oncogene family | [401258](https://www.ncbi.nlm.nih.gov/gene/?term=401258) | -1.6 | 0.01962 | 72.4 | 128.5 |
| TRAJ40 | T cell receptor alpha joining 40 | [28715](https://www.ncbi.nlm.nih.gov/gene/?term=28715) | -1.6 | 0.06018 | 9.9 | 21.8 |
| MIR3648-1 | microRNA 3648-1 | [100500862](https://www.ncbi.nlm.nih.gov/gene/?term=100500862) | -1.6 | 0.06630 | 0.5 | 1.6 |
| SIMC1 | SUMO interacting motifs containing 1 | [375484](https://www.ncbi.nlm.nih.gov/gene/?term=375484) | -1.6 | 0.00018 | 125.2 | 205.3 |
| XKR9 | XK related 9 | [389668](https://www.ncbi.nlm.nih.gov/gene/?term=389668) | -1.6 | 0.03792 | 6.4 | 12.4 |
| ALS2CR11 | amyotrophic lateral sclerosis 2 chromosome region candidate 11 | [151254](https://www.ncbi.nlm.nih.gov/gene/?term=151254) | -1.6 | 0.06020 | 4.4 | 9.4 |
| NAA60 | N(alpha)-acetyltransferase 60, NatF catalytic subunit | [79903](https://www.ncbi.nlm.nih.gov/gene/?term=79903) | -1.6 | 0.00018 | 157.5 | 257.4 |
| LAPTM4A | lysosomal protein transmembrane 4 alpha | [9741](https://www.ncbi.nlm.nih.gov/gene/?term=9741) | 1.6 | 0.00008 | 1372.5 | 844.5 |
| TMEM63B | transmembrane protein 63B | [55362](https://www.ncbi.nlm.nih.gov/gene/?term=55362) | -1.6 | 0.04870 | 4311.9 | 8893.6 |
| PRMT5 | protein arginine methyltransferase 5 | [10419](https://www.ncbi.nlm.nih.gov/gene/?term=10419) | 1.6 | 0.00015 | 754.5 | 464.0 |
| RNF165 | ring finger protein 165 | [494470](https://www.ncbi.nlm.nih.gov/gene/?term=494470) | -1.6 | 0.06422 | 10.9 | 26.0 |
| MYO1G | myosin IG | [64005](https://www.ncbi.nlm.nih.gov/gene/?term=64005) | 1.6 | 0.00016 | 4155.8 | 2553.9 |
| KLF3 | Kruppel-like factor 3 (basic) | [51274](https://www.ncbi.nlm.nih.gov/gene/?term=51274) | -1.6 | 0.00261 | 10444.5 | 17411.3 |
| LINC01003 | long intergenic non-protein coding RNA 1003 | [100128822](https://www.ncbi.nlm.nih.gov/gene/?term=100128822) | 1.6 | 0.00233 | 187.9 | 112.8 |
| DNASE1L2 | deoxyribonuclease I-like 2 | [1775](https://www.ncbi.nlm.nih.gov/gene/?term=1775) | 1.6 | 0.01393 | 28.1 | 16.3 |
| AP1S3 | adaptor related protein complex 1 sigma 3 subunit | [130340](https://www.ncbi.nlm.nih.gov/gene/?term=130340) | -1.6 | 0.00954 | 31.8 | 55.7 |
| IFFO1 | intermediate filament family orphan 1 | [25900](https://www.ncbi.nlm.nih.gov/gene/?term=25900) | 1.6 | 0.00066 | 399.7 | 242.9 |
| CLPB | ClpB homolog, mitochondrial AAA ATPase chaperonin | [81570](https://www.ncbi.nlm.nih.gov/gene/?term=81570) | -1.6 | 0.01245 | 373.1 | 641.8 |
| HSPH1 | heat shock protein family H (Hsp110) member 1 | [10808](https://www.ncbi.nlm.nih.gov/gene/?term=10808) | 1.6 | 0.01853 | 2641.3 | 1506.1 |
| CD164 | CD164 molecule | [8763](https://www.ncbi.nlm.nih.gov/gene/?term=8763) | 1.6 | 0.00003 | 10095.6 | 6268.3 |
| KLHDC8B | kelch domain containing 8B | [200942](https://www.ncbi.nlm.nih.gov/gene/?term=200942) | 1.6 | 0.00948 | 375.7 | 220.1 |
| JAM2 | junctional adhesion molecule 2 | [58494](https://www.ncbi.nlm.nih.gov/gene/?term=58494) | -1.6 | 0.06578 | 3.5 | 8.8 |
| ARHGAP27 | Rho GTPase activating protein 27 | [201176](https://www.ncbi.nlm.nih.gov/gene/?term=201176) | 1.6 | 0.01329 | 193.0 | 112.2 |
| CORO1A | coronin 1A | [11151](https://www.ncbi.nlm.nih.gov/gene/?term=11151) | 1.6 | 0.00000 | 12171.5 | 7609.8 |
| FLJ32255 | uncharacterized LOC643977 | [643977](https://www.ncbi.nlm.nih.gov/gene/?term=643977) | 1.6 | 0.00058 | 534.3 | 325.6 |
| LOC105372392 |  | [105372392](https://www.ncbi.nlm.nih.gov/gene/?term=105372392) | -1.6 | 0.03204 | 22.5 | 42.1 |
| BCKDHA | branched chain keto acid dehydrogenase E1, alpha polypeptide | [593](https://www.ncbi.nlm.nih.gov/gene/?term=593) | 1.6 | 0.00052 | 625.5 | 381.0 |
| ZNF585B | zinc finger protein 585B | [92285](https://www.ncbi.nlm.nih.gov/gene/?term=92285) | -1.6 | 0.00014 | 225.4 | 368.4 |
| EML6 | echinoderm microtubule associated protein like 6 | [400954](https://www.ncbi.nlm.nih.gov/gene/?term=400954) | -1.6 | 0.01754 | 89.4 | 156.2 |
| EMC3-AS1 | EMC3 antisense RNA 1 | [442075](https://www.ncbi.nlm.nih.gov/gene/?term=442075) | -1.6 | 0.01014 | 23.7 | 41.3 |
| ZNF221 | zinc finger protein 221 | [7638](https://www.ncbi.nlm.nih.gov/gene/?term=7638) | -1.6 | 0.01883 | 20.8 | 37.0 |
| FCRLB | Fc receptor like B | [127943](https://www.ncbi.nlm.nih.gov/gene/?term=127943) | 1.6 | 0.01749 | 23.2 | 13.4 |
| SF1 | splicing factor 1 | [7536](https://www.ncbi.nlm.nih.gov/gene/?term=7536) | -1.6 | 0.00673 | 1012.9 | 1712.9 |
| EIF2S2P3 | eukaryotic translation initiation factor 2 subunit 2 beta pseudogene 3 | [283014](https://www.ncbi.nlm.nih.gov/gene/?term=283014) | -1.6 | 0.06532 | 4.3 | 10.3 |
| DOCK3 | dedicator of cytokinesis 3 | [1795](https://www.ncbi.nlm.nih.gov/gene/?term=1795) | -1.6 | 0.03566 | 8.2 | 15.8 |
| ANG | angiogenin | [283](https://www.ncbi.nlm.nih.gov/gene/?term=283) | 1.6 | 0.01582 | 28.6 | 16.4 |
| GPR174 | G protein-coupled receptor 174 | [84636](https://www.ncbi.nlm.nih.gov/gene/?term=84636) | -1.6 | 0.00622 | 335.3 | 566.8 |
| CFLAR | CASP8 and FADD like apoptosis regulator | [8837](https://www.ncbi.nlm.nih.gov/gene/?term=8837) | 1.6 | 0.00242 | 19425.6 | 11744.4 |
| CENPW | centromere protein W | [387103](https://www.ncbi.nlm.nih.gov/gene/?term=387103) | 1.6 | 0.01293 | 47.5 | 27.9 |
| TRPC1 | transient receptor potential cation channel subfamily C member 1 | [7220](https://www.ncbi.nlm.nih.gov/gene/?term=7220) | -1.6 | 0.02910 | 77.8 | 139.7 |
| TP53 | tumor protein p53 | [7157](https://www.ncbi.nlm.nih.gov/gene/?term=7157) | -1.6 | 0.01543 | 149.5 | 260.9 |
| ZNF737 | zinc finger protein 737 | [100129842](https://www.ncbi.nlm.nih.gov/gene/?term=100129842) | -1.6 | 0.00000 | 530.4 | 851.9 |
| MAST4 | microtubule associated serine/threonine kinase family member 4 | [375449](https://www.ncbi.nlm.nih.gov/gene/?term=375449) | -1.6 | 0.00284 | 329.9 | 548.7 |
| RP2 | retinitis pigmentosa 2 (X-linked recessive) | [6102](https://www.ncbi.nlm.nih.gov/gene/?term=6102) | 1.6 | 0.00228 | 3301.0 | 1990.8 |
| LARGE | like-glycosyltransferase | [9215](https://www.ncbi.nlm.nih.gov/gene/?term=9215) | -1.6 | 0.01335 | 83.7 | 143.4 |
| TMEM199 | transmembrane protein 199 | [147007](https://www.ncbi.nlm.nih.gov/gene/?term=147007) | 1.6 | 0.00000 | 658.7 | 413.9 |
| SGCD | sarcoglycan delta | [6444](https://www.ncbi.nlm.nih.gov/gene/?term=6444) | -1.6 | 0.06615 | 0.7 | 2.3 |
| MGRN1 | mahogunin ring finger 1 | [23295](https://www.ncbi.nlm.nih.gov/gene/?term=23295) | -1.6 | 0.00638 | 260.8 | 440.2 |
| TMEM205 | transmembrane protein 205 | [374882](https://www.ncbi.nlm.nih.gov/gene/?term=374882) | 1.6 | 0.00064 | 402.8 | 246.7 |
| AURKB | aurora kinase B | [9212](https://www.ncbi.nlm.nih.gov/gene/?term=9212) | 1.6 | 0.06044 | 26.7 | 13.2 |
| LOC107985400 |  | [107985400](https://www.ncbi.nlm.nih.gov/gene/?term=107985400) | -1.6 | 0.06206 | 6.2 | 14.0 |
| LOC107984960 |  | [107984960](https://www.ncbi.nlm.nih.gov/gene/?term=107984960) | -1.6 | 0.04941 | 31.3 | 61.7 |
| COX5B | cytochrome c oxidase subunit 5B | [1329](https://www.ncbi.nlm.nih.gov/gene/?term=1329) | 1.6 | 0.00016 | 1796.7 | 1109.2 |
| ERN1 | endoplasmic reticulum to nucleus signaling 1 | [2081](https://www.ncbi.nlm.nih.gov/gene/?term=2081) | -1.6 | 0.00835 | 580.5 | 982.2 |
| RGS19 | regulator of G-protein signaling 19 | [10287](https://www.ncbi.nlm.nih.gov/gene/?term=10287) | 1.6 | 0.00007 | 2362.1 | 1459.1 |
| EIF4HP1 | eukaryotic translation initiation factor 4H pseudogene 1 | [401316](https://www.ncbi.nlm.nih.gov/gene/?term=401316) | 1.6 | 0.01780 | 10.7 | 6.2 |
| LOC145474 | uncharacterized LOC145474 | [145474](https://www.ncbi.nlm.nih.gov/gene/?term=145474) | -1.6 | 0.05429 | 50.2 | 100.2 |
| FUNDC2 | FUN14 domain containing 2 | [65991](https://www.ncbi.nlm.nih.gov/gene/?term=65991) | -1.6 | 0.02505 | 1785.0 | 3144.7 |
| C1orf162 | chromosome 1 open reading frame 162 | [128346](https://www.ncbi.nlm.nih.gov/gene/?term=128346) | 1.6 | 0.00001 | 1487.3 | 926.3 |
| COPS2 | COP9 signalosome subunit 2 | [9318](https://www.ncbi.nlm.nih.gov/gene/?term=9318) | -1.6 | 0.01201 | 3799.9 | 6520.5 |
| MGST3 | microsomal glutathione S-transferase 3 | [4259](https://www.ncbi.nlm.nih.gov/gene/?term=4259) | 1.6 | 0.00007 | 576.0 | 356.7 |
| LOC91450 | uncharacterized LOC91450 | [91450](https://www.ncbi.nlm.nih.gov/gene/?term=91450) | 1.6 | 0.05557 | 10.2 | 5.2 |
| FAS | Fas cell surface death receptor | [355](https://www.ncbi.nlm.nih.gov/gene/?term=355) | 1.6 | 0.00884 | 2966.7 | 1753.1 |
| SYNRG | synergin, gamma | [11276](https://www.ncbi.nlm.nih.gov/gene/?term=11276) | -1.6 | 0.00057 | 48.1 | 78.1 |
| CTSC | cathepsin C | [1075](https://www.ncbi.nlm.nih.gov/gene/?term=1075) | 1.6 | 0.00008 | 5176.9 | 3212.9 |
| MYEF2 | myelin expression factor 2 | [50804](https://www.ncbi.nlm.nih.gov/gene/?term=50804) | -1.6 | 0.00643 | 85.7 | 145.5 |
| SSBP4 | single stranded DNA binding protein 4 | [170463](https://www.ncbi.nlm.nih.gov/gene/?term=170463) | 1.6 | 0.00085 | 348.0 | 213.7 |
| AMACR | alpha-methylacyl-CoA racemase | [23600](https://www.ncbi.nlm.nih.gov/gene/?term=23600) | -1.6 | 0.02235 | 7.9 | 14.9 |
| C1R | complement C1r subcomponent | [715](https://www.ncbi.nlm.nih.gov/gene/?term=715) | 1.6 | 0.01544 | 18.6 | 11.6 |
| CCR2 | C-C motif chemokine receptor 2 | [729230](https://www.ncbi.nlm.nih.gov/gene/?term=729230) | 1.6 | 0.00548 | 5498.8 | 3289.2 |
| ZNF865 | zinc finger protein 865 | [100507290](https://www.ncbi.nlm.nih.gov/gene/?term=100507290) | -1.6 | 0.04427 | 23.3 | 45.4 |
| NCOA2 | nuclear receptor coactivator 2 | [10499](https://www.ncbi.nlm.nih.gov/gene/?term=10499) | -1.6 | 0.00059 | 8635.2 | 14161.0 |
| KLHL3 | kelch like family member 3 | [26249](https://www.ncbi.nlm.nih.gov/gene/?term=26249) | -1.6 | 0.01051 | 77.9 | 133.4 |
| IL15 | interleukin 15 | [3600](https://www.ncbi.nlm.nih.gov/gene/?term=3600) | 1.6 | 0.00095 | 308.0 | 188.2 |
| AP1S1 | adaptor related protein complex 1 sigma 1 subunit | [1174](https://www.ncbi.nlm.nih.gov/gene/?term=1174) | 1.6 | 0.00012 | 200.2 | 123.2 |
| UBC | ubiquitin C | [7316](https://www.ncbi.nlm.nih.gov/gene/?term=7316) | 1.6 | 0.00055 | 34949.7 | 21547.9 |
| PTMAP5 | prothymosin, alpha pseudogene 5 | [150928](https://www.ncbi.nlm.nih.gov/gene/?term=150928) | 1.6 | 0.01800 | 12.9 | 7.3 |
| SPTSSA | serine palmitoyltransferase small subunit A | [171546](https://www.ncbi.nlm.nih.gov/gene/?term=171546) | 1.6 | 0.00040 | 348.3 | 214.6 |
| ZNF592 | zinc finger protein 592 | [9640](https://www.ncbi.nlm.nih.gov/gene/?term=9640) | -1.6 | 0.00622 | 770.3 | 1292.7 |
| LOC107985350 |  | [107985350](https://www.ncbi.nlm.nih.gov/gene/?term=107985350) | 1.6 | 0.02409 | 49.2 | 27.9 |
| APOBEC3C | apolipoprotein B mRNA editing enzyme catalytic subunit 3C | [27350](https://www.ncbi.nlm.nih.gov/gene/?term=27350) | -1.6 | 0.01068 | 240.0 | 406.9 |
| TMEM55B | transmembrane protein 55B | [90809](https://www.ncbi.nlm.nih.gov/gene/?term=90809) | 1.6 | 0.00001 | 750.9 | 469.3 |
| LOC100131859 | SAP domain containing ribonucleoprotein pseudogene | [100131859](https://www.ncbi.nlm.nih.gov/gene/?term=100131859) | -1.6 | 0.01223 | 18.3 | 31.2 |
| IKZF2 | IKAROS family zinc finger 2 | [22807](https://www.ncbi.nlm.nih.gov/gene/?term=22807) | -1.6 | 0.01279 | 1128.8 | 1933.5 |
| LOC101929506 |  | [101929506](https://www.ncbi.nlm.nih.gov/gene/?term=101929506) | -1.6 | 0.04159 | 28.8 | 53.1 |
| APBB3 | amyloid beta precursor protein binding family B member 3 | [10307](https://www.ncbi.nlm.nih.gov/gene/?term=10307) | 1.6 | 0.00072 | 264.7 | 162.2 |
| LOC105378342 |  | [105378342](https://www.ncbi.nlm.nih.gov/gene/?term=105378342) | 1.6 | 0.07183 | 5.2 | 2.4 |
| LOC107984895 |  | [107984895](https://www.ncbi.nlm.nih.gov/gene/?term=107984895) | -1.6 | 0.05716 | 6.9 | 14.9 |
| LOC105370114 |  | [105370114](https://www.ncbi.nlm.nih.gov/gene/?term=105370114) | -1.6 | 0.05603 | 58.3 | 172.6 |
| TRIM41 | tripartite motif containing 41 | [90933](https://www.ncbi.nlm.nih.gov/gene/?term=90933) | 1.6 | 0.00004 | 438.9 | 272.8 |
| RBMS2 | RNA binding motif single stranded interacting protein 2 | [5939](https://www.ncbi.nlm.nih.gov/gene/?term=5939) | -1.6 | 0.01094 | 110.2 | 188.7 |
| RBCK1 | RANBP2-type and C3HC4-type zinc finger containing 1 | [10616](https://www.ncbi.nlm.nih.gov/gene/?term=10616) | 1.6 | 0.00039 | 1431.3 | 879.8 |
| SLC52A2 | solute carrier family 52 member 2 | [79581](https://www.ncbi.nlm.nih.gov/gene/?term=79581) | 1.6 | 0.00124 | 244.3 | 148.9 |
| ZNF282 | zinc finger protein 282 | [8427](https://www.ncbi.nlm.nih.gov/gene/?term=8427) | 1.6 | 0.00253 | 246.2 | 149.4 |
| RGMB | repulsive guidance molecule family member b | [285704](https://www.ncbi.nlm.nih.gov/gene/?term=285704) | -1.6 | 0.00745 | 53.1 | 90.3 |
| CENPB | centromere protein B | [1059](https://www.ncbi.nlm.nih.gov/gene/?term=1059) | 1.6 | 0.00027 | 1147.7 | 708.0 |
| PURA | purine-rich element binding protein A | [5813](https://www.ncbi.nlm.nih.gov/gene/?term=5813) | -1.6 | 0.00005 | 417.2 | 670.3 |
| CCRL2 | C-C motif chemokine receptor like 2 | [9034](https://www.ncbi.nlm.nih.gov/gene/?term=9034) | 1.6 | 0.01486 | 544.0 | 317.2 |
| HEIH | hepatocellular carcinoma up-regulated EZH2-associated long non-coding RNA | [100859930](https://www.ncbi.nlm.nih.gov/gene/?term=100859930) | 1.6 | 0.00068 | 439.3 | 269.4 |
| HIST2H2AA4 | histone cluster 2, H2aa4 | [723790](https://www.ncbi.nlm.nih.gov/gene/?term=723790) | 1.6 | 0.02078 | 21.8 | 12.3 |
| PLXNC1 | plexin C1 | [10154](https://www.ncbi.nlm.nih.gov/gene/?term=10154) | 1.6 | 0.00689 | 20878.4 | 12480.0 |
| FBXL14 | F-box and leucine-rich repeat protein 14 | [144699](https://www.ncbi.nlm.nih.gov/gene/?term=144699) | 1.6 | 0.00005 | 283.7 | 176.3 |
| SYT17 | synaptotagmin 17 | [51760](https://www.ncbi.nlm.nih.gov/gene/?term=51760) | -1.6 | 0.02962 | 15.1 | 27.0 |
| MYO9A | myosin IXA | [4649](https://www.ncbi.nlm.nih.gov/gene/?term=4649) | -1.6 | 0.00000 | 1046.4 | 1649.6 |
| GUSBP4 | glucuronidase, beta pseudogene 4 | [375513](https://www.ncbi.nlm.nih.gov/gene/?term=375513) | -1.6 | 0.00093 | 53.2 | 88.1 |
| FBXL20 | F-box and leucine-rich repeat protein 20 | [84961](https://www.ncbi.nlm.nih.gov/gene/?term=84961) | -1.6 | 0.00013 | 899.3 | 1448.3 |
| SRGAP3 | SLIT-ROBO Rho GTPase activating protein 3 | [9901](https://www.ncbi.nlm.nih.gov/gene/?term=9901) | -1.6 | 0.02428 | 29.4 | 52.0 |
| PSMC2 | proteasome 26S subunit, ATPase 2 | [5701](https://www.ncbi.nlm.nih.gov/gene/?term=5701) | 1.6 | 0.00002 | 1344.9 | 842.3 |
| PTGES2-AS1 | PTGES2 antisense RNA 1 (head to head) | [389791](https://www.ncbi.nlm.nih.gov/gene/?term=389791) | 1.6 | 0.04284 | 8.5 | 4.8 |
| RRH | retinal pigment epithelium-derived rhodopsin homolog | [10692](https://www.ncbi.nlm.nih.gov/gene/?term=10692) | -1.6 | 0.06109 | 5.4 | 11.1 |
| PRCP | prolylcarboxypeptidase | [5547](https://www.ncbi.nlm.nih.gov/gene/?term=5547) | 1.6 | 0.00044 | 3040.1 | 1876.8 |
| ZNF137P | zinc finger protein 137, pseudogene | [7696](https://www.ncbi.nlm.nih.gov/gene/?term=7696) | -1.6 | 0.00320 | 46.7 | 78.1 |
| ASPRV1 | aspartic peptidase, retroviral-like 1 | [151516](https://www.ncbi.nlm.nih.gov/gene/?term=151516) | 1.6 | 0.04235 | 195.9 | 106.1 |
| LOC390586 | hydroxyacyl-CoA dehydrogenase pseudogene | [390586](https://www.ncbi.nlm.nih.gov/gene/?term=390586) | -1.6 | 0.05318 | 21.1 | 41.8 |
| RELT | RELT tumor necrosis factor receptor | [84957](https://www.ncbi.nlm.nih.gov/gene/?term=84957) | 1.6 | 0.00329 | 2195.9 | 1330.5 |
| ZNF469 | zinc finger protein 469 | [84627](https://www.ncbi.nlm.nih.gov/gene/?term=84627) | -1.6 | 0.06479 | 8.8 | 19.4 |
| GEMIN8P1 | gem nuclear organelle associated protein 8 pseudogene 1 | [100128431](https://www.ncbi.nlm.nih.gov/gene/?term=100128431) | 1.6 | 0.06839 | 13.9 | 5.9 |
| OPTN | optineurin | [10133](https://www.ncbi.nlm.nih.gov/gene/?term=10133) | -1.6 | 0.03912 | 18526.9 | 33119.4 |
| ZNF814 | zinc finger protein 814 | [730051](https://www.ncbi.nlm.nih.gov/gene/?term=730051) | -1.6 | 0.00028 | 175.7 | 286.3 |
| LOC107985724 |  | [107985724](https://www.ncbi.nlm.nih.gov/gene/?term=107985724) | -1.6 | 0.04500 | 7.2 | 14.4 |
| NCS1 | neuronal calcium sensor 1 | [23413](https://www.ncbi.nlm.nih.gov/gene/?term=23413) | -1.6 | 0.05193 | 7.0 | 13.5 |
| LRRC28 | leucine rich repeat containing 28 | [123355](https://www.ncbi.nlm.nih.gov/gene/?term=123355) | -1.6 | 0.00004 | 287.5 | 457.9 |
| RNF181 | ring finger protein 181 | [51255](https://www.ncbi.nlm.nih.gov/gene/?term=51255) | 1.6 | 0.00006 | 1265.3 | 790.2 |
| LPP | LIM domain containing preferred translocation partner in lipoma | [4026](https://www.ncbi.nlm.nih.gov/gene/?term=4026) | -1.6 | 0.00278 | 2181.1 | 3579.8 |
| MIRLET7D | microRNA let-7d | [406886](https://www.ncbi.nlm.nih.gov/gene/?term=406886) | -1.6 | 0.06714 | 4.4 | 10.5 |
| ORC6 | origin recognition complex, subunit 6 | [23594](https://www.ncbi.nlm.nih.gov/gene/?term=23594) | 1.6 | 0.01618 | 59.0 | 34.6 |
| LOC102725044 |  | [102725044](https://www.ncbi.nlm.nih.gov/gene/?term=102725044) | 1.6 | 0.00250 | 49.7 | 30.4 |
| LOC101928669 |  | [101928669](https://www.ncbi.nlm.nih.gov/gene/?term=101928669) | -1.6 | 0.05718 | 12.8 | 25.5 |
| NTAN1P2 | N-terminal asparagine amidase pseudogene 2 | [100420307](https://www.ncbi.nlm.nih.gov/gene/?term=100420307) | -1.6 | 0.03032 | 17.1 | 31.2 |
| SNX18 | sorting nexin 18 | [112574](https://www.ncbi.nlm.nih.gov/gene/?term=112574) | 1.6 | 0.00505 | 4964.5 | 3007.3 |
| LOC105369441 |  | [105369441](https://www.ncbi.nlm.nih.gov/gene/?term=105369441) | -1.6 | 0.05475 | 5.8 | 12.2 |
| LOC105376266 |  | [105376266](https://www.ncbi.nlm.nih.gov/gene/?term=105376266) | -1.6 | 0.02600 | 102.3 | 179.1 |
| IPW | imprinted in Prader-Willi syndrome (non-protein coding) | [3653](https://www.ncbi.nlm.nih.gov/gene/?term=3653) | -1.6 | 0.02710 | 35.6 | 64.6 |
| LINC01220 | long intergenic non-protein coding RNA 1220 | [731223](https://www.ncbi.nlm.nih.gov/gene/?term=731223) | 1.6 | 0.03684 | 28.5 | 15.6 |
| LOC105375384 |  | [105375384](https://www.ncbi.nlm.nih.gov/gene/?term=105375384) | -1.6 | 0.01459 | 57.6 | 99.9 |
| TICAM1 | toll like receptor adaptor molecule 1 | [148022](https://www.ncbi.nlm.nih.gov/gene/?term=148022) | -1.6 | 0.04422 | 13.4 | 26.1 |
| DHFR | dihydrofolate reductase | [1719](https://www.ncbi.nlm.nih.gov/gene/?term=1719) | 1.6 | 0.00174 | 411.8 | 252.6 |
| LOC107986408 |  | [107986408](https://www.ncbi.nlm.nih.gov/gene/?term=107986408) | -1.6 | 0.01921 | 48.6 | 84.9 |
| PWWP2B | PWWP domain containing 2B | [170394](https://www.ncbi.nlm.nih.gov/gene/?term=170394) | 1.6 | 0.00090 | 285.2 | 175.1 |
| LOC105378939 |  | [105378939](https://www.ncbi.nlm.nih.gov/gene/?term=105378939) | -1.6 | 0.00824 | 35.6 | 60.0 |
| RBM41 | RNA binding motif protein 41 | [55285](https://www.ncbi.nlm.nih.gov/gene/?term=55285) | -1.6 | 0.00137 | 1081.9 | 1759.6 |
| FAM20B | family with sequence similarity 20 member B | [9917](https://www.ncbi.nlm.nih.gov/gene/?term=9917) | -1.6 | 0.02835 | 4099.2 | 7256.1 |
| TNKS | tankyrase | [8658](https://www.ncbi.nlm.nih.gov/gene/?term=8658) | -1.6 | 0.00004 | 971.5 | 1551.0 |
| CHKB-AS1 | CHKB antisense RNA 1 (head to head) | [100144603](https://www.ncbi.nlm.nih.gov/gene/?term=100144603) | 1.6 | 0.00573 | 26.8 | 16.7 |
| PIWIL4 | piwi like RNA-mediated gene silencing 4 | [143689](https://www.ncbi.nlm.nih.gov/gene/?term=143689) | 1.6 | 0.00394 | 148.1 | 89.7 |
| FAM8A1 | family with sequence similarity 8 member A1 | [51439](https://www.ncbi.nlm.nih.gov/gene/?term=51439) | 1.6 | 0.00313 | 6264.1 | 3830.3 |
| HNRNPA3 | heterogeneous nuclear ribonucleoprotein A3 | [220988](https://www.ncbi.nlm.nih.gov/gene/?term=220988) | -1.6 | 0.00289 | 1502.0 | 2453.8 |
| CDK14 | cyclin-dependent kinase 14 | [5218](https://www.ncbi.nlm.nih.gov/gene/?term=5218) | -1.6 | 0.00080 | 648.1 | 1053.0 |
| TCEB1 | transcription elongation factor B subunit 1 | [6921](https://www.ncbi.nlm.nih.gov/gene/?term=6921) | 1.6 | 0.00002 | 510.2 | 320.5 |
| PPP4C | protein phosphatase 4 catalytic subunit | [5531](https://www.ncbi.nlm.nih.gov/gene/?term=5531) | 1.6 | 0.00004 | 1813.3 | 1138.0 |
| RN7SL4P | RNA, 7SL, cytoplasmic 4, pseudogene | [6030](https://www.ncbi.nlm.nih.gov/gene/?term=6030) | -1.6 | 0.04456 | 5502.3 | 10287.9 |
| TSC1 | tuberous sclerosis 1 | [7248](https://www.ncbi.nlm.nih.gov/gene/?term=7248) | -1.6 | 0.00074 | 696.2 | 1124.7 |
| WDFY1 | WD repeat and FYVE domain containing 1 | [57590](https://www.ncbi.nlm.nih.gov/gene/?term=57590) | 1.6 | 0.00037 | 2926.2 | 1819.3 |
| PLCD1 | phospholipase C delta 1 | [5333](https://www.ncbi.nlm.nih.gov/gene/?term=5333) | 1.6 | 0.00016 | 297.5 | 186.0 |
| SPAG5 | sperm associated antigen 5 | [10615](https://www.ncbi.nlm.nih.gov/gene/?term=10615) | 1.6 | 0.01373 | 255.5 | 150.5 |
| ZFYVE19 | zinc finger FYVE-type containing 19 | [84936](https://www.ncbi.nlm.nih.gov/gene/?term=84936) | 1.6 | 0.00005 | 274.5 | 171.5 |
| BCKDHB | branched chain keto acid dehydrogenase E1, beta polypeptide | [594](https://www.ncbi.nlm.nih.gov/gene/?term=594) | -1.6 | 0.00112 | 182.3 | 296.4 |
| KMT2C | lysine methyltransferase 2C | [58508](https://www.ncbi.nlm.nih.gov/gene/?term=58508) | -1.6 | 0.00064 | 6070.1 | 9796.2 |
| RTCB | RNA 2',3'-cyclic phosphate and 5'-OH ligase | [51493](https://www.ncbi.nlm.nih.gov/gene/?term=51493) | 1.6 | 0.00001 | 1159.5 | 729.2 |
| EIF4H | eukaryotic translation initiation factor 4H | [7458](https://www.ncbi.nlm.nih.gov/gene/?term=7458) | 1.6 | 0.00001 | 4087.0 | 2579.8 |
| LOC101927595 |  | [101927595](https://www.ncbi.nlm.nih.gov/gene/?term=101927595) | -1.6 | 0.05362 | 11.7 | 22.0 |
| ASGR2 | asialoglycoprotein receptor 2 | [433](https://www.ncbi.nlm.nih.gov/gene/?term=433) | 1.6 | 0.01117 | 74.0 | 44.1 |
| ANKHD1-EIF4EBP3 | ANKHD1-EIF4EBP3 readthrough | [404734](https://www.ncbi.nlm.nih.gov/gene/?term=404734) | -1.6 | 0.06690 | 3.1 | 7.7 |
| GRIP1 | glutamate receptor interacting protein 1 | [23426](https://www.ncbi.nlm.nih.gov/gene/?term=23426) | -1.6 | 0.03277 | 16.6 | 29.9 |
| RN7SL124P | RNA, 7SL, cytoplasmic 124, pseudogene | [106480955](https://www.ncbi.nlm.nih.gov/gene/?term=106480955) | 1.6 | 0.07206 | 10.1 | 4.4 |
| ANKRD13D | ankyrin repeat domain 13 family member D | [338692](https://www.ncbi.nlm.nih.gov/gene/?term=338692) | 1.6 | 0.00105 | 1370.7 | 846.6 |
| LOC101928354 |  | [101928354](https://www.ncbi.nlm.nih.gov/gene/?term=101928354) | -1.6 | 0.05048 | 22.4 | 42.0 |
| ZNF326 | zinc finger protein 326 | [284695](https://www.ncbi.nlm.nih.gov/gene/?term=284695) | -1.6 | 0.00002 | 485.6 | 771.2 |
| SIN3A | SIN3 transcription regulator family member A | [25942](https://www.ncbi.nlm.nih.gov/gene/?term=25942) | -1.6 | 0.00400 | 819.9 | 1351.7 |
| BRAT1 | BRCA1 associated ATM activator 1 | [221927](https://www.ncbi.nlm.nih.gov/gene/?term=221927) | 1.6 | 0.00077 | 698.9 | 433.2 |
| MED13L | mediator complex subunit 13 like | [23389](https://www.ncbi.nlm.nih.gov/gene/?term=23389) | -1.6 | 0.00020 | 7373.0 | 11842.5 |
| NFKBID | NFKB inhibitor delta | [84807](https://www.ncbi.nlm.nih.gov/gene/?term=84807) | 1.6 | 0.00040 | 221.0 | 137.7 |
| ZNF876P | zinc finger protein 876, pseudogene | [642280](https://www.ncbi.nlm.nih.gov/gene/?term=642280) | -1.6 | 0.03368 | 30.5 | 53.0 |
| CYBB | cytochrome b-245, beta polypeptide | [1536](https://www.ncbi.nlm.nih.gov/gene/?term=1536) | 1.6 | 0.00253 | 26546.4 | 16260.8 |
| WSB1 | WD repeat and SOCS box containing 1 | [26118](https://www.ncbi.nlm.nih.gov/gene/?term=26118) | 1.6 | 0.00476 | 4292.7 | 2592.2 |
| TNFRSF12A | tumor necrosis factor receptor superfamily member 12A | [51330](https://www.ncbi.nlm.nih.gov/gene/?term=51330) | 1.6 | 0.01708 | 93.0 | 54.6 |
| SRSF9 | serine/arginine-rich splicing factor 9 | [8683](https://www.ncbi.nlm.nih.gov/gene/?term=8683) | 1.6 | 0.00001 | 1752.1 | 1106.7 |
| PCDH1 | protocadherin 1 | [5097](https://www.ncbi.nlm.nih.gov/gene/?term=5097) | -1.6 | 0.06023 | 138.6 | 280.5 |
| ERGIC3 | ERGIC and golgi 3 | [51614](https://www.ncbi.nlm.nih.gov/gene/?term=51614) | -1.6 | 0.00003 | 355.1 | 564.2 |
| NCF4 | neutrophil cytosolic factor 4 | [4689](https://www.ncbi.nlm.nih.gov/gene/?term=4689) | 1.6 | 0.01117 | 737.8 | 440.2 |
| UNC119 | unc-119 lipid binding chaperone | [9094](https://www.ncbi.nlm.nih.gov/gene/?term=9094) | 1.6 | 0.00053 | 1693.9 | 1052.7 |
| CREB3 | cAMP responsive element binding protein 3 | [10488](https://www.ncbi.nlm.nih.gov/gene/?term=10488) | 1.6 | 0.00003 | 261.5 | 163.6 |
| ZNF503-AS2 | ZNF503 antisense RNA 2 | [100131213](https://www.ncbi.nlm.nih.gov/gene/?term=100131213) | 1.6 | 0.07330 | 5.0 | 2.2 |
| RUNX1T1 | RUNX1 translocation partner 1 | [862](https://www.ncbi.nlm.nih.gov/gene/?term=862) | -1.6 | 0.06355 | 18.8 | 40.4 |
| PTPRB | protein tyrosine phosphatase, receptor type B | [5787](https://www.ncbi.nlm.nih.gov/gene/?term=5787) | -1.6 | 0.06797 | 8.6 | 19.3 |
| ZFYVE1 | zinc finger FYVE-type containing 1 | [53349](https://www.ncbi.nlm.nih.gov/gene/?term=53349) | -1.6 | 0.00062 | 480.0 | 773.1 |
| LINC00623 | long intergenic non-protein coding RNA 623 | [728855](https://www.ncbi.nlm.nih.gov/gene/?term=728855) | 1.6 | 0.01334 | 271.3 | 160.5 |
| TICAM2 | toll like receptor adaptor molecule 2 | [353376](https://www.ncbi.nlm.nih.gov/gene/?term=353376) | 1.6 | 0.00763 | 27.9 | 16.8 |
| SLIRP | SRA stem-loop interacting RNA binding protein | [81892](https://www.ncbi.nlm.nih.gov/gene/?term=81892) | 1.6 | 0.00014 | 269.9 | 168.2 |
| PTCRA | pre T-cell antigen receptor alpha | [171558](https://www.ncbi.nlm.nih.gov/gene/?term=171558) | 1.6 | 0.02893 | 309.3 | 174.4 |
| NAGLU | N-acetyl-alpha-glucosaminidase | [4669](https://www.ncbi.nlm.nih.gov/gene/?term=4669) | 1.6 | 0.00263 | 234.6 | 143.6 |
| NDUFA9 | NADH:ubiquinone oxidoreductase subunit A9 | [4704](https://www.ncbi.nlm.nih.gov/gene/?term=4704) | 1.6 | 0.00007 | 926.8 | 580.3 |
| LSM3P1 | LSM3 homolog, U6 small nuclear RNA and mRNA degradation associated pseudogene 1 | [100303748](https://www.ncbi.nlm.nih.gov/gene/?term=100303748) | -1.6 | 0.06533 | 6.2 | 17.3 |
| CEACAM1 | carcinoembryonic antigen related cell adhesion molecule 1 | [634](https://www.ncbi.nlm.nih.gov/gene/?term=634) | 1.6 | 0.06003 | 551.3 | 278.0 |
| LOC100422382 | nocturnin pseudogene | [100422382](https://www.ncbi.nlm.nih.gov/gene/?term=100422382) | -1.6 | 0.07019 | 5.1 | 12.5 |
| OLAH | oleoyl-ACP hydrolase | [55301](https://www.ncbi.nlm.nih.gov/gene/?term=55301) | 1.6 | 0.05332 | 18.2 | 5.2 |
| ISM1 | isthmin 1, angiogenesis inhibitor | [140862](https://www.ncbi.nlm.nih.gov/gene/?term=140862) | -1.6 | 0.05693 | 6.0 | 11.6 |
| TPM3 | tropomyosin 3 | [7170](https://www.ncbi.nlm.nih.gov/gene/?term=7170) | 1.6 | 0.00001 | 12966.4 | 8220.4 |
| IQCE | IQ motif containing E | [23288](https://www.ncbi.nlm.nih.gov/gene/?term=23288) | 1.6 | 0.00097 | 528.3 | 327.8 |
| C14orf80 | chromosome 14 open reading frame 80 | [283643](https://www.ncbi.nlm.nih.gov/gene/?term=283643) | 1.6 | 0.00240 | 129.3 | 79.1 |
| HTR7P1 | 5-hydroxytryptamine receptor 7 pseudogene 1 | [93164](https://www.ncbi.nlm.nih.gov/gene/?term=93164) | 1.6 | 0.00144 | 109.7 | 67.6 |
| IMPA2 | inositol monophosphatase 2 | [3613](https://www.ncbi.nlm.nih.gov/gene/?term=3613) | 1.6 | 0.01037 | 674.7 | 403.0 |
| RAPGEFL1 | Rap guanine nucleotide exchange factor like 1 | [51195](https://www.ncbi.nlm.nih.gov/gene/?term=51195) | -1.6 | 0.04343 | 28.8 | 54.2 |
| CBFA2T2 | CBFA2/RUNX1 translocation partner 2 | [9139](https://www.ncbi.nlm.nih.gov/gene/?term=9139) | -1.6 | 0.00566 | 251.5 | 416.2 |
| POMC | proopiomelanocortin | [5443](https://www.ncbi.nlm.nih.gov/gene/?term=5443) | 1.5 | 0.01498 | 100.1 | 59.3 |
| NBPF15 | neuroblastoma breakpoint family member 15 | [284565](https://www.ncbi.nlm.nih.gov/gene/?term=284565) | -1.5 | 0.00275 | 150.4 | 246.1 |
| LOC100128905 | uncharacterized LOC100128905 | [100128905](https://www.ncbi.nlm.nih.gov/gene/?term=100128905) | 1.5 | 0.06128 | 8.2 | 4.3 |
| GNG2 | G protein subunit gamma 2 | [54331](https://www.ncbi.nlm.nih.gov/gene/?term=54331) | -1.5 | 0.00748 | 1085.6 | 1787.7 |
| DHX34 | DEAH-box helicase 34 | [9704](https://www.ncbi.nlm.nih.gov/gene/?term=9704) | 1.5 | 0.00462 | 1255.8 | 764.5 |
| RPP25 | ribonuclease P/MRP subunit p25 | [54913](https://www.ncbi.nlm.nih.gov/gene/?term=54913) | 1.5 | 0.00411 | 37.6 | 22.8 |
| NAIF1 | nuclear apoptosis inducing factor 1 | [203245](https://www.ncbi.nlm.nih.gov/gene/?term=203245) | 1.5 | 0.00000 | 260.4 | 164.3 |
| PLGRKT | plasminogen receptor with a C-terminal lysine | [55848](https://www.ncbi.nlm.nih.gov/gene/?term=55848) | 1.5 | 0.00227 | 293.3 | 181.3 |
| TINF2 | TERF1 (TRF1)-interacting nuclear factor 2 | [26277](https://www.ncbi.nlm.nih.gov/gene/?term=26277) | 1.5 | 0.00005 | 2697.4 | 1700.9 |
| RIC8A | RIC8 guanine nucleotide exchange factor A | [60626](https://www.ncbi.nlm.nih.gov/gene/?term=60626) | 1.5 | 0.00000 | 1875.6 | 1190.0 |
| NSUN3 | NOP2/Sun RNA methyltransferase family member 3 | [63899](https://www.ncbi.nlm.nih.gov/gene/?term=63899) | -1.5 | 0.03888 | 11094.3 | 19900.6 |
| BCL2 | B-cell CLL/lymphoma 2 | [596](https://www.ncbi.nlm.nih.gov/gene/?term=596) | -1.5 | 0.00052 | 1190.8 | 1914.1 |
| SORBS1 | sorbin and SH3 domain containing 1 | [10580](https://www.ncbi.nlm.nih.gov/gene/?term=10580) | -1.5 | 0.03259 | 163.5 | 288.2 |
| COMMD8 | COMM domain containing 8 | [54951](https://www.ncbi.nlm.nih.gov/gene/?term=54951) | 1.5 | 0.00011 | 645.5 | 406.4 |
| LOC105378756 |  | [105378756](https://www.ncbi.nlm.nih.gov/gene/?term=105378756) | 1.5 | 0.06088 | 10.2 | 5.3 |
| ZBTB44 | zinc finger and BTB domain containing 44 | [29068](https://www.ncbi.nlm.nih.gov/gene/?term=29068) | -1.5 | 0.00098 | 5087.3 | 8202.6 |
| MRVI1 | murine retrovirus integration site 1 homolog | [10335](https://www.ncbi.nlm.nih.gov/gene/?term=10335) | 1.5 | 0.03259 | 1465.0 | 834.1 |
| C8orf44 | chromosome 8 open reading frame 44 | [56260](https://www.ncbi.nlm.nih.gov/gene/?term=56260) | -1.5 | 0.01469 | 26.8 | 47.0 |
| MIR25 | microRNA 25 | [407014](https://www.ncbi.nlm.nih.gov/gene/?term=407014) | 1.5 | 0.06902 | 7.6 | 3.7 |
| CD40LG | CD40 ligand | [959](https://www.ncbi.nlm.nih.gov/gene/?term=959) | -1.5 | 0.00413 | 140.4 | 230.1 |
| RCBTB2 | RCC1 and BTB domain containing protein 2 | [1102](https://www.ncbi.nlm.nih.gov/gene/?term=1102) | 1.5 | 0.00133 | 2501.2 | 1551.7 |
| SLIT1 | slit guidance ligand 1 | [6585](https://www.ncbi.nlm.nih.gov/gene/?term=6585) | 1.5 | 0.03263 | 25.3 | 14.5 |
| ADA | adenosine deaminase | [100](https://www.ncbi.nlm.nih.gov/gene/?term=100) | 1.5 | 0.00619 | 508.5 | 309.1 |
| CCND2 | cyclin D2 | [894](https://www.ncbi.nlm.nih.gov/gene/?term=894) | -1.5 | 0.00821 | 1594.8 | 2641.2 |
| LINC01506 | long intergenic non-protein coding RNA 1506 | [101927015](https://www.ncbi.nlm.nih.gov/gene/?term=101927015) | 1.5 | 0.02721 | 898.3 | 516.4 |
| MIR421 | microRNA 421 | [693122](https://www.ncbi.nlm.nih.gov/gene/?term=693122) | -1.5 | 0.05847 | 14.6 | 29.0 |
| RELL2 | RELT like 2 | [285613](https://www.ncbi.nlm.nih.gov/gene/?term=285613) | 1.5 | 0.00671 | 170.6 | 103.7 |
| CSTA | cystatin A | [1475](https://www.ncbi.nlm.nih.gov/gene/?term=1475) | 1.5 | 0.00897 | 1670.4 | 1010.0 |
| LOC105373331 |  | [105373331](https://www.ncbi.nlm.nih.gov/gene/?term=105373331) | -1.5 | 0.06745 | 8.3 | 22.4 |
| C9orf114 | chromosome 9 open reading frame 114 | [51490](https://www.ncbi.nlm.nih.gov/gene/?term=51490) | 1.5 | 0.00086 | 474.9 | 294.7 |
| CACNG6 | calcium voltage-gated channel auxiliary subunit gamma 6 | [59285](https://www.ncbi.nlm.nih.gov/gene/?term=59285) | -1.5 | 0.06130 | 12.9 | 25.5 |
| SOX13 | SRY-box 13 | [9580](https://www.ncbi.nlm.nih.gov/gene/?term=9580) | -1.5 | 0.05851 | 6.7 | 14.1 |
| LOC107986069 |  | [107986069](https://www.ncbi.nlm.nih.gov/gene/?term=107986069) | -1.5 | 0.06438 | 8.3 | 17.6 |
| LOC101928487 |  | [101928487](https://www.ncbi.nlm.nih.gov/gene/?term=101928487) | 1.5 | 0.02552 | 14.2 | 8.1 |
| LAMB3 | laminin subunit beta 3 | [3914](https://www.ncbi.nlm.nih.gov/gene/?term=3914) | 1.5 | 0.04555 | 27.9 | 15.2 |
| RPS17P16 | ribosomal protein S17 pseudogene 16 | [402057](https://www.ncbi.nlm.nih.gov/gene/?term=402057) | 1.5 | 0.05305 | 18.8 | 9.2 |
| LOC107984251 |  | [107984251](https://www.ncbi.nlm.nih.gov/gene/?term=107984251) | 1.5 | 0.01699 | 311.5 | 183.8 |
| JOSD2 | Josephin domain containing 2 | [126119](https://www.ncbi.nlm.nih.gov/gene/?term=126119) | 1.5 | 0.00005 | 113.1 | 71.5 |
| MR1 | major histocompatibility complex, class I-related | [3140](https://www.ncbi.nlm.nih.gov/gene/?term=3140) | 1.5 | 0.00042 | 1920.1 | 1202.1 |
| TRIP6 | thyroid hormone receptor interactor 6 | [7205](https://www.ncbi.nlm.nih.gov/gene/?term=7205) | 1.5 | 0.00059 | 82.5 | 51.7 |
| RPSAP41 | ribosomal protein SA pseudogene 41 | [100270918](https://www.ncbi.nlm.nih.gov/gene/?term=100270918) | 1.5 | 0.03585 | 10.7 | 6.0 |
| PTGER3 | prostaglandin E receptor 3 | [5733](https://www.ncbi.nlm.nih.gov/gene/?term=5733) | -1.5 | 0.01824 | 19.2 | 32.3 |
| LOC105369199 |  | [105369199](https://www.ncbi.nlm.nih.gov/gene/?term=105369199) | 1.5 | 0.01837 | 94.7 | 56.0 |
| LOC101929638 |  | [101929638](https://www.ncbi.nlm.nih.gov/gene/?term=101929638) | -1.5 | 0.05592 | 70.3 | 134.5 |
| LOC100996842 | uncharacterized LOC100996842 | [100996842](https://www.ncbi.nlm.nih.gov/gene/?term=100996842) | 1.5 | 0.01012 | 33.1 | 19.8 |
| GCC1 | GRIP and coiled-coil domain containing 1 | [79571](https://www.ncbi.nlm.nih.gov/gene/?term=79571) | 1.5 | 0.00000 | 1590.6 | 1015.1 |
| LOC283788 | FSHD region gene 1 pseudogene | [283788](https://www.ncbi.nlm.nih.gov/gene/?term=283788) | -1.5 | 0.04629 | 444.4 | 831.1 |
| DGKQ | diacylglycerol kinase theta | [1609](https://www.ncbi.nlm.nih.gov/gene/?term=1609) | 1.5 | 0.00974 | 414.7 | 250.1 |
| CACYBP | calcyclin binding protein | [27101](https://www.ncbi.nlm.nih.gov/gene/?term=27101) | 1.5 | 0.00053 | 1310.2 | 816.2 |
| LOC728175 | uncharacterized LOC728175 | [728175](https://www.ncbi.nlm.nih.gov/gene/?term=728175) | 1.5 | 0.05713 | 22.4 | 12.0 |
| LOC107984529 |  | [107984529](https://www.ncbi.nlm.nih.gov/gene/?term=107984529) | -1.5 | 0.00746 | 261.9 | 429.7 |
| ANKRD17 | ankyrin repeat domain 17 | [26057](https://www.ncbi.nlm.nih.gov/gene/?term=26057) | -1.5 | 0.00036 | 2527.0 | 4021.7 |
| RTCA-AS1 | RTCA antisense RNA 1 | [100506007](https://www.ncbi.nlm.nih.gov/gene/?term=100506007) | -1.5 | 0.04248 | 230.9 | 416.3 |
| NDUFS2 | NADH:ubiquinone oxidoreductase core subunit S2 | [4720](https://www.ncbi.nlm.nih.gov/gene/?term=4720) | 1.5 | 0.00000 | 1647.1 | 1053.2 |
| HCFC1 | host cell factor C1 | [3054](https://www.ncbi.nlm.nih.gov/gene/?term=3054) | -1.5 | 0.02443 | 596.7 | 1028.8 |
| KLF4 | Kruppel-like factor 4 (gut) | [9314](https://www.ncbi.nlm.nih.gov/gene/?term=9314) | 1.5 | 0.00866 | 522.8 | 316.0 |
| CD1D | CD1d molecule | [912](https://www.ncbi.nlm.nih.gov/gene/?term=912) | 1.5 | 0.00451 | 1141.3 | 697.3 |
| CS | citrate synthase | [1431](https://www.ncbi.nlm.nih.gov/gene/?term=1431) | -1.5 | 0.00317 | 468.1 | 761.4 |
| UBE2T | ubiquitin conjugating enzyme E2 T | [29089](https://www.ncbi.nlm.nih.gov/gene/?term=29089) | 1.5 | 0.02855 | 69.1 | 40.1 |
| DDX41 | DEAD-box helicase 41 | [51428](https://www.ncbi.nlm.nih.gov/gene/?term=51428) | 1.5 | 0.00000 | 930.0 | 594.4 |
| B3GNT8 | UDP-GlcNAc:betaGal beta-1,3-N-acetylglucosaminyltransferase 8 | [374907](https://www.ncbi.nlm.nih.gov/gene/?term=374907) | 1.5 | 0.00770 | 466.2 | 283.4 |
| KIF2C | kinesin family member 2C | [11004](https://www.ncbi.nlm.nih.gov/gene/?term=11004) | 1.5 | 0.04726 | 128.0 | 69.9 |
| TRIM38 | tripartite motif containing 38 | [10475](https://www.ncbi.nlm.nih.gov/gene/?term=10475) | 1.5 | 0.00524 | 5562.4 | 3404.3 |
| B3GNT2 | UDP-GlcNAc:betaGal beta-1,3-N-acetylglucosaminyltransferase 2 | [10678](https://www.ncbi.nlm.nih.gov/gene/?term=10678) | 1.5 | 0.00027 | 1534.4 | 964.1 |
| BCL2L15 | BCL2 like 15 | [440603](https://www.ncbi.nlm.nih.gov/gene/?term=440603) | -1.5 | 0.04593 | 17.0 | 31.8 |
| SLC16A5 | solute carrier family 16 member 5 | [9121](https://www.ncbi.nlm.nih.gov/gene/?term=9121) | 1.5 | 0.00145 | 229.0 | 143.3 |
| RNASE2 | ribonuclease A family member 2 | [6036](https://www.ncbi.nlm.nih.gov/gene/?term=6036) | 1.5 | 0.06438 | 886.3 | 444.9 |
| LRRC4 | leucine rich repeat containing 4 | [64101](https://www.ncbi.nlm.nih.gov/gene/?term=64101) | 1.5 | 0.00937 | 888.2 | 537.4 |
| C16orf59 | chromosome 16 open reading frame 59 | [80178](https://www.ncbi.nlm.nih.gov/gene/?term=80178) | 1.5 | 0.07409 | 13.4 | 6.4 |
| LOC101926913 | uncharacterized LOC101926913 | [101926913](https://www.ncbi.nlm.nih.gov/gene/?term=101926913) | -1.5 | 0.06603 | 17.9 | 36.9 |
| ERMN | ermin | [57471](https://www.ncbi.nlm.nih.gov/gene/?term=57471) | -1.5 | 0.01749 | 79.6 | 136.3 |
| CLTA | clathrin light chain A | [1211](https://www.ncbi.nlm.nih.gov/gene/?term=1211) | 1.5 | 0.00000 | 1378.7 | 882.4 |
| RPL7AP22 | ribosomal protein L7a pseudogene 22 | [100271039](https://www.ncbi.nlm.nih.gov/gene/?term=100271039) | -1.5 | 0.07039 | 4.9 | 12.1 |
| LOC107985083 |  | [107985083](https://www.ncbi.nlm.nih.gov/gene/?term=107985083) | -1.5 | 0.05262 | 28.9 | 55.8 |
| PPP1R14BP3 | protein phosphatase 1 regulatory inhibitor subunit 14B pseudogene 3 | [100507617](https://www.ncbi.nlm.nih.gov/gene/?term=100507617) | -1.5 | 0.04845 | 25.1 | 47.5 |
| ADPRHL2 | ADP-ribosylhydrolase like 2 | [54936](https://www.ncbi.nlm.nih.gov/gene/?term=54936) | 1.5 | 0.00006 | 748.6 | 473.8 |
| RWDD3 | RWD domain containing 3 | [25950](https://www.ncbi.nlm.nih.gov/gene/?term=25950) | -1.5 | 0.00530 | 260.1 | 423.1 |
| MLX | MLX, MAX dimerization protein | [6945](https://www.ncbi.nlm.nih.gov/gene/?term=6945) | 1.5 | 0.00000 | 1236.1 | 789.5 |
| RRM2 | ribonucleotide reductase regulatory subunit M2 | [6241](https://www.ncbi.nlm.nih.gov/gene/?term=6241) | 1.5 | 0.06348 | 592.7 | 301.9 |
| C1orf216 | chromosome 1 open reading frame 216 | [127703](https://www.ncbi.nlm.nih.gov/gene/?term=127703) | 1.5 | 0.00040 | 180.6 | 113.1 |
| ANP32B | acidic nuclear phosphoprotein 32 family member B | [10541](https://www.ncbi.nlm.nih.gov/gene/?term=10541) | -1.5 | 0.03527 | 2282.0 | 4023.1 |
| PEX6 | peroxisomal biogenesis factor 6 | [5190](https://www.ncbi.nlm.nih.gov/gene/?term=5190) | 1.5 | 0.00054 | 701.1 | 440.5 |
| SLX4 | SLX4 structure-specific endonuclease subunit | [84464](https://www.ncbi.nlm.nih.gov/gene/?term=84464) | -1.5 | 0.04327 | 60.1 | 109.7 |
| LILRB4 | leukocyte immunoglobulin like receptor B4 | [11006](https://www.ncbi.nlm.nih.gov/gene/?term=11006) | 1.5 | 0.05361 | 19.9 | 10.7 |
| RIBC1 | RIB43A domain with coiled-coils 1 | [158787](https://www.ncbi.nlm.nih.gov/gene/?term=158787) | 1.5 | 0.04545 | 12.6 | 7.1 |
| LOC107984656 |  | [107984656](https://www.ncbi.nlm.nih.gov/gene/?term=107984656) | -1.5 | 0.04624 | 436.8 | 800.9 |
| LYZ | lysozyme | [4069](https://www.ncbi.nlm.nih.gov/gene/?term=4069) | 1.5 | 0.01183 | 90207.0 | 54288.4 |
| NADK | NAD kinase | [65220](https://www.ncbi.nlm.nih.gov/gene/?term=65220) | 1.5 | 0.00217 | 4688.0 | 2922.9 |
| UBN2 | ubinuclein 2 | [254048](https://www.ncbi.nlm.nih.gov/gene/?term=254048) | -1.5 | 0.00005 | 1457.6 | 2289.8 |
| EPN2 | epsin 2 | [22905](https://www.ncbi.nlm.nih.gov/gene/?term=22905) | -1.5 | 0.01123 | 94.9 | 154.7 |
| CHRNA5 | cholinergic receptor nicotinic alpha 5 subunit | [1138](https://www.ncbi.nlm.nih.gov/gene/?term=1138) | 1.5 | 0.03151 | 20.2 | 11.3 |
| COPG1 | coatomer protein complex subunit gamma 1 | [22820](https://www.ncbi.nlm.nih.gov/gene/?term=22820) | 1.5 | 0.00000 | 2564.6 | 1651.5 |
| ARHGAP1 | Rho GTPase activating protein 1 | [392](https://www.ncbi.nlm.nih.gov/gene/?term=392) | -1.5 | 0.01462 | 171.6 | 288.8 |
| GRAMD1A | GRAM domain containing 1A | [57655](https://www.ncbi.nlm.nih.gov/gene/?term=57655) | 1.5 | 0.01837 | 836.5 | 496.5 |
| RNF43 | ring finger protein 43 | [54894](https://www.ncbi.nlm.nih.gov/gene/?term=54894) | -1.5 | 0.05446 | 10.3 | 20.0 |
| CYTH2 | cytohesin 2 | [9266](https://www.ncbi.nlm.nih.gov/gene/?term=9266) | 1.5 | 0.00002 | 570.5 | 363.4 |
| ARPC2 | actin related protein 2/3 complex subunit 2 | [10109](https://www.ncbi.nlm.nih.gov/gene/?term=10109) | 1.5 | 0.00019 | 14818.5 | 9398.5 |
| ELK3 | ELK3, ETS transcription factor | [2004](https://www.ncbi.nlm.nih.gov/gene/?term=2004) | -1.5 | 0.01293 | 353.2 | 590.6 |
| ACVR1B | activin A receptor type 1B | [91](https://www.ncbi.nlm.nih.gov/gene/?term=91) | 1.5 | 0.00193 | 385.4 | 240.0 |
| KCNG2 | potassium voltage-gated channel modifier subfamily G member 2 | [26251](https://www.ncbi.nlm.nih.gov/gene/?term=26251) | 1.5 | 0.07211 | 11.2 | 5.2 |
| CTBP1 | C-terminal binding protein 1 | [1487](https://www.ncbi.nlm.nih.gov/gene/?term=1487) | 1.5 | 0.00015 | 3255.1 | 2059.8 |
| TRNG |  | [4563](https://www.ncbi.nlm.nih.gov/gene/?term=4563) | -1.5 | 0.02877 | 82.0 | 140.7 |
| ADAM17 | ADAM metallopeptidase domain 17 | [6868](https://www.ncbi.nlm.nih.gov/gene/?term=6868) | 1.5 | 0.00003 | 2099.0 | 1342.5 |
| HSF5 | heat shock transcription factor 5 | [124535](https://www.ncbi.nlm.nih.gov/gene/?term=124535) | -1.5 | 0.00979 | 85.4 | 140.0 |
| LOC254896 | uncharacterized LOC254896 | [254896](https://www.ncbi.nlm.nih.gov/gene/?term=254896) | 1.5 | 0.02408 | 277.4 | 162.5 |
| FTO | fat mass and obesity associated | [79068](https://www.ncbi.nlm.nih.gov/gene/?term=79068) | -1.5 | 0.00066 | 421.4 | 672.6 |
| BHLHB9 | basic helix-loop-helix domain containing, class B, 9 | [80823](https://www.ncbi.nlm.nih.gov/gene/?term=80823) | -1.5 | 0.00221 | 28.1 | 45.4 |
| NKTR | natural killer cell triggering receptor | [4820](https://www.ncbi.nlm.nih.gov/gene/?term=4820) | -1.5 | 0.00080 | 2568.1 | 4093.8 |
| MFSD14A | major facilitator superfamily domain containing 14A | [64645](https://www.ncbi.nlm.nih.gov/gene/?term=64645) | 1.5 | 0.00014 | 2773.1 | 1759.2 |
| BBOF1 | basal body orientation factor 1 | [80127](https://www.ncbi.nlm.nih.gov/gene/?term=80127) | -1.5 | 0.05863 | 4912.1 | 9756.2 |
| CSNK2A2 | casein kinase 2 alpha 2 | [1459](https://www.ncbi.nlm.nih.gov/gene/?term=1459) | -1.5 | 0.00599 | 137.6 | 226.2 |
| KRT8P12 | keratin 8 pseudogene 12 | [90133](https://www.ncbi.nlm.nih.gov/gene/?term=90133) | 1.5 | 0.00141 | 124.0 | 77.8 |
| LIN52 | lin-52 DREAM MuvB core complex component | [91750](https://www.ncbi.nlm.nih.gov/gene/?term=91750) | -1.5 | 0.00244 | 390.1 | 629.2 |
| ZC2HC1A | zinc finger C2HC-type containing 1A | [51101](https://www.ncbi.nlm.nih.gov/gene/?term=51101) | -1.5 | 0.02045 | 102.1 | 173.9 |
| NACAP3 |  | [101928685](https://www.ncbi.nlm.nih.gov/gene/?term=101928685) | -1.5 | 0.05305 | 10.9 | 19.7 |
| NCR3LG1 | natural killer cell cytotoxicity receptor 3 ligand 1 | [374383](https://www.ncbi.nlm.nih.gov/gene/?term=374383) | -1.5 | 0.02032 | 37.6 | 64.7 |
| PGAM1 | phosphoglycerate mutase 1 | [5223](https://www.ncbi.nlm.nih.gov/gene/?term=5223) | 1.5 | 0.00018 | 843.4 | 535.6 |
| CDC14A | cell division cycle 14A | [8556](https://www.ncbi.nlm.nih.gov/gene/?term=8556) | -1.5 | 0.00006 | 789.1 | 1244.0 |
| LOC101927156 | uncharacterized LOC101927156 | [101927156](https://www.ncbi.nlm.nih.gov/gene/?term=101927156) | -1.5 | 0.01556 | 55.2 | 93.5 |
| ALYREF | Aly/REF export factor | [10189](https://www.ncbi.nlm.nih.gov/gene/?term=10189) | 1.5 | 0.00072 | 667.8 | 419.2 |
| RPL26P6 | ribosomal protein L26 pseudogene 6 | [100131526](https://www.ncbi.nlm.nih.gov/gene/?term=100131526) | -1.5 | 0.05053 | 7.5 | 14.4 |
| ACOT8 | acyl-CoA thioesterase 8 | [10005](https://www.ncbi.nlm.nih.gov/gene/?term=10005) | 1.5 | 0.00000 | 324.4 | 207.3 |
| CD53 | CD53 molecule | [963](https://www.ncbi.nlm.nih.gov/gene/?term=963) | 1.5 | 0.00043 | 10728.4 | 6760.7 |
| SFMBT1 | Scm-like with four mbt domains 1 | [51460](https://www.ncbi.nlm.nih.gov/gene/?term=51460) | -1.5 | 0.00105 | 235.8 | 377.7 |
| ADGRA2 | adhesion G protein-coupled receptor A2 | [25960](https://www.ncbi.nlm.nih.gov/gene/?term=25960) | 1.5 | 0.03119 | 51.0 | 29.1 |
| PAWR | pro-apoptotic WT1 regulator | [5074](https://www.ncbi.nlm.nih.gov/gene/?term=5074) | -1.5 | 0.00417 | 182.1 | 293.9 |
| MIR4697HG | MIR4697 host gene | [283174](https://www.ncbi.nlm.nih.gov/gene/?term=283174) | -1.5 | 0.05145 | 21.6 | 41.1 |
| PPP1R9A | protein phosphatase 1 regulatory subunit 9A | [55607](https://www.ncbi.nlm.nih.gov/gene/?term=55607) | -1.5 | 0.03801 | 70.5 | 124.9 |
| TRAF3IP2 | TRAF3 interacting protein 2 | [10758](https://www.ncbi.nlm.nih.gov/gene/?term=10758) | -1.5 | 0.01291 | 49.2 | 83.2 |
| DAAM1 | dishevelled associated activator of morphogenesis 1 | [23002](https://www.ncbi.nlm.nih.gov/gene/?term=23002) | -1.5 | 0.00019 | 358.9 | 564.8 |
| LEPROT | leptin receptor overlapping transcript | [54741](https://www.ncbi.nlm.nih.gov/gene/?term=54741) | -1.5 | 0.00246 | 893.1 | 1435.7 |
| LOC100420981 | nucleosome assembly protein 1 like 1 pseudogene | [100420981](https://www.ncbi.nlm.nih.gov/gene/?term=100420981) | -1.5 | 0.02019 | 14.0 | 24.6 |
| DAPK3 | death-associated protein kinase 3 | [1613](https://www.ncbi.nlm.nih.gov/gene/?term=1613) | 1.5 | 0.00001 | 521.5 | 333.7 |
| MSL3 | male-specific lethal 3 homolog (Drosophila) | [10943](https://www.ncbi.nlm.nih.gov/gene/?term=10943) | 1.5 | 0.00203 | 1869.7 | 1167.2 |
| LOC107984727 |  | [107984727](https://www.ncbi.nlm.nih.gov/gene/?term=107984727) | -1.5 | 0.06720 | 6.5 | 12.6 |
| NAGPA | N-acetylglucosamine-1-phosphodiester alpha-N-acetylglucosaminidase | [51172](https://www.ncbi.nlm.nih.gov/gene/?term=51172) | 1.5 | 0.00004 | 304.5 | 193.3 |
| LOC100130520 | CD300c molecule-like | [100130520](https://www.ncbi.nlm.nih.gov/gene/?term=100130520) | 1.5 | 0.06708 | 594.6 | 300.7 |
| SETD2 | SET domain containing 2 | [29072](https://www.ncbi.nlm.nih.gov/gene/?term=29072) | -1.5 | 0.00001 | 4360.3 | 6800.1 |
| CSF1 | colony stimulating factor 1 | [1435](https://www.ncbi.nlm.nih.gov/gene/?term=1435) | -1.5 | 0.04190 | 46.7 | 83.8 |
| MIR628 | microRNA 628 | [693213](https://www.ncbi.nlm.nih.gov/gene/?term=693213) | -1.5 | 0.06339 | 7.9 | 15.4 |
| SYNGR2 | synaptogyrin 2 | [9144](https://www.ncbi.nlm.nih.gov/gene/?term=9144) | 1.5 | 0.00001 | 2096.4 | 1345.5 |
| BTG2 | BTG family member 2 | [7832](https://www.ncbi.nlm.nih.gov/gene/?term=7832) | -1.5 | 0.01085 | 628.5 | 1038.2 |
| CXXC4 | CXXC finger protein 4 | [80319](https://www.ncbi.nlm.nih.gov/gene/?term=80319) | -1.5 | 0.05064 | 7.4 | 13.1 |
| CDK2 | cyclin-dependent kinase 2 | [1017](https://www.ncbi.nlm.nih.gov/gene/?term=1017) | 1.5 | 0.00027 | 180.4 | 114.8 |
| RNF130 | ring finger protein 130 | [55819](https://www.ncbi.nlm.nih.gov/gene/?term=55819) | 1.5 | 0.00397 | 7130.0 | 4415.9 |
| CDCP1 | CUB domain containing protein 1 | [64866](https://www.ncbi.nlm.nih.gov/gene/?term=64866) | 1.5 | 0.06935 | 34.7 | 17.3 |
| SLC25A38 | solute carrier family 25 member 38 | [54977](https://www.ncbi.nlm.nih.gov/gene/?term=54977) | -1.5 | 0.02285 | 1354.7 | 2276.1 |
| BIRC2 | baculoviral IAP repeat containing 2 | [329](https://www.ncbi.nlm.nih.gov/gene/?term=329) | -1.5 | 0.03491 | 25048.3 | 43949.3 |
| LOC105376095 |  | [105376095](https://www.ncbi.nlm.nih.gov/gene/?term=105376095) | -1.5 | 0.05020 | 36.9 | 68.2 |
| KCNQ5 | potassium voltage-gated channel subfamily Q member 5 | [56479](https://www.ncbi.nlm.nih.gov/gene/?term=56479) | -1.5 | 0.02464 | 63.0 | 108.4 |
| WNT7A | Wnt family member 7A | [7476](https://www.ncbi.nlm.nih.gov/gene/?term=7476) | -1.5 | 0.04363 | 9.1 | 17.0 |
| SEPT14P18 | septin 14 pseudogene 18 | [107126284](https://www.ncbi.nlm.nih.gov/gene/?term=107126284) | 1.5 | 0.05251 | 42.8 | 23.5 |
| RFX3-AS1 | RFX3 antisense RNA 1 | [101929302](https://www.ncbi.nlm.nih.gov/gene/?term=101929302) | -1.5 | 0.03388 | 62.4 | 108.2 |
| C8orf82 | chromosome 8 open reading frame 82 | [414919](https://www.ncbi.nlm.nih.gov/gene/?term=414919) | 1.5 | 0.01209 | 131.0 | 78.8 |
| RNF144A | ring finger protein 144A | [9781](https://www.ncbi.nlm.nih.gov/gene/?term=9781) | -1.5 | 0.00681 | 545.9 | 889.0 |
| ARID2 | AT-rich interaction domain 2 | [196528](https://www.ncbi.nlm.nih.gov/gene/?term=196528) | -1.5 | 0.00004 | 1365.5 | 2137.9 |
| MFSD10 | major facilitator superfamily domain containing 10 | [10227](https://www.ncbi.nlm.nih.gov/gene/?term=10227) | 1.5 | 0.00078 | 504.8 | 316.5 |
| CFL2 | cofilin 2 | [1073](https://www.ncbi.nlm.nih.gov/gene/?term=1073) | -1.5 | 0.00075 | 88.0 | 138.6 |
| PI4K2B | phosphatidylinositol 4-kinase type 2 beta | [55300](https://www.ncbi.nlm.nih.gov/gene/?term=55300) | 1.5 | 0.00020 | 1311.2 | 833.9 |
| FAM69B | family with sequence similarity 69 member B | [138311](https://www.ncbi.nlm.nih.gov/gene/?term=138311) | 1.5 | 0.03923 | 106.0 | 59.6 |
| GAB1 | GRB2 associated binding protein 1 | [2549](https://www.ncbi.nlm.nih.gov/gene/?term=2549) | -1.5 | 0.00036 | 1418.5 | 2243.3 |
| SNORA71A | small nucleolar RNA, H/ACA box 71A | [26777](https://www.ncbi.nlm.nih.gov/gene/?term=26777) | 1.5 | 0.02531 | 37.3 | 21.9 |
| RGS3 | regulator of G-protein signaling 3 | [5998](https://www.ncbi.nlm.nih.gov/gene/?term=5998) | 1.5 | 0.00251 | 1004.1 | 626.3 |
| STIP1 | stress induced phosphoprotein 1 | [10963](https://www.ncbi.nlm.nih.gov/gene/?term=10963) | 1.5 | 0.00080 | 1636.2 | 1030.2 |
| ADGRE1 | adhesion G protein-coupled receptor E1 | [2015](https://www.ncbi.nlm.nih.gov/gene/?term=2015) | 1.5 | 0.02728 | 2527.7 | 1489.3 |
| B4GALNT3 | beta-1,4-N-acetyl-galactosaminyltransferase 3 | [283358](https://www.ncbi.nlm.nih.gov/gene/?term=283358) | 1.5 | 0.07113 | 65.9 | 32.2 |
| UBXN7 | UBX domain protein 7 | [26043](https://www.ncbi.nlm.nih.gov/gene/?term=26043) | -1.5 | 0.00000 | 1681.7 | 2614.6 |
| MAEA | macrophage erythroblast attacher | [10296](https://www.ncbi.nlm.nih.gov/gene/?term=10296) | 1.5 | 0.00019 | 1085.3 | 689.8 |
| ZER1 | zyg-11 related cell cycle regulator | [10444](https://www.ncbi.nlm.nih.gov/gene/?term=10444) | -1.5 | 0.04401 | 8693.2 | 15601.9 |
| SLC6A9 | solute carrier family 6 member 9 | [6536](https://www.ncbi.nlm.nih.gov/gene/?term=6536) | -1.5 | 0.06645 | 311.3 | 619.3 |
| IGSF6 | immunoglobulin superfamily member 6 | [10261](https://www.ncbi.nlm.nih.gov/gene/?term=10261) | 1.5 | 0.00971 | 5660.2 | 3457.4 |
| MRGBP | MRG/MORF4L binding protein | [55257](https://www.ncbi.nlm.nih.gov/gene/?term=55257) | 1.5 | 0.00009 | 371.1 | 236.8 |
| LOC105376113 |  | [105376113](https://www.ncbi.nlm.nih.gov/gene/?term=105376113) | -1.5 | 0.01898 | 18.3 | 30.0 |
| GPR160 | G protein-coupled receptor 160 | [26996](https://www.ncbi.nlm.nih.gov/gene/?term=26996) | 1.5 | 0.01052 | 921.7 | 564.6 |
| UBQLN4 | ubiquilin 4 | [56893](https://www.ncbi.nlm.nih.gov/gene/?term=56893) | -1.5 | 0.02022 | 66.4 | 113.1 |
| FAM60A | family with sequence similarity 60 member A | [58516](https://www.ncbi.nlm.nih.gov/gene/?term=58516) | 1.5 | 0.01112 | 30.3 | 17.9 |
| NPAS2 | neuronal PAS domain protein 2 | [4862](https://www.ncbi.nlm.nih.gov/gene/?term=4862) | -1.5 | 0.07330 | 5.1 | 12.7 |
| ATM | ATM serine/threonine kinase | [472](https://www.ncbi.nlm.nih.gov/gene/?term=472) | -1.5 | 0.00001 | 12590.9 | 19584.8 |
| NLRP12 | NLR family, pyrin domain containing 12 | [91662](https://www.ncbi.nlm.nih.gov/gene/?term=91662) | 1.5 | 0.01065 | 2928.0 | 1786.7 |
| HIF1A | hypoxia inducible factor 1 alpha subunit | [3091](https://www.ncbi.nlm.nih.gov/gene/?term=3091) | 1.5 | 0.00134 | 7345.7 | 4624.6 |
| SRA1 | steroid receptor RNA activator 1 | [10011](https://www.ncbi.nlm.nih.gov/gene/?term=10011) | 1.5 | 0.00007 | 421.4 | 270.3 |
| LOC107984333 |  | [107984333](https://www.ncbi.nlm.nih.gov/gene/?term=107984333) | -1.5 | 0.02967 | 71.7 | 121.9 |
| LOC107985188 |  | [107985188](https://www.ncbi.nlm.nih.gov/gene/?term=107985188) | -1.5 | 0.05344 | 13.9 | 26.5 |
| VPS72 | vacuolar protein sorting 72 homolog | [6944](https://www.ncbi.nlm.nih.gov/gene/?term=6944) | 1.5 | 0.00003 | 468.9 | 300.6 |
| TRPM2 | transient receptor potential cation channel subfamily M member 2 | [7226](https://www.ncbi.nlm.nih.gov/gene/?term=7226) | 1.5 | 0.03981 | 353.8 | 201.4 |
| FTH1P10 | ferritin, heavy polypeptide 1 pseudogene 10 | [2502](https://www.ncbi.nlm.nih.gov/gene/?term=2502) | -1.5 | 0.04929 | 7.1 | 12.7 |
| S100A4 | S100 calcium binding protein A4 | [6275](https://www.ncbi.nlm.nih.gov/gene/?term=6275) | 1.5 | 0.00137 | 8002.4 | 5040.3 |
| TAMM41 | TAM41 mitochondrial translocator assembly and maintenance homolog | [132001](https://www.ncbi.nlm.nih.gov/gene/?term=132001) | -1.5 | 0.01496 | 47.2 | 77.9 |
| HS3ST1 | heparan sulfate-glucosamine 3-sulfotransferase 1 | [9957](https://www.ncbi.nlm.nih.gov/gene/?term=9957) | -1.5 | 0.05352 | 14.6 | 27.1 |
| TGFA | transforming growth factor alpha | [7039](https://www.ncbi.nlm.nih.gov/gene/?term=7039) | 1.5 | 0.02760 | 898.9 | 527.9 |
| IGFALS | insulin like growth factor binding protein acid labile subunit | [3483](https://www.ncbi.nlm.nih.gov/gene/?term=3483) | -1.5 | 0.06221 | 4.5 | 9.5 |
| LINC00211 | long intergenic non-protein coding RNA 211 | [101929559](https://www.ncbi.nlm.nih.gov/gene/?term=101929559) | 1.5 | 0.06134 | 208.5 | 109.3 |
| LZTFL1 | leucine zipper transcription factor like 1 | [54585](https://www.ncbi.nlm.nih.gov/gene/?term=54585) | -1.5 | 0.03913 | 1054.8 | 1888.3 |
| COX8A | cytochrome c oxidase subunit 8A | [1351](https://www.ncbi.nlm.nih.gov/gene/?term=1351) | 1.5 | 0.00058 | 682.1 | 432.7 |
| CTSD | cathepsin D | [1509](https://www.ncbi.nlm.nih.gov/gene/?term=1509) | 1.5 | 0.00176 | 5332.6 | 3353.2 |
| PTPRO | protein tyrosine phosphatase, receptor type O | [5800](https://www.ncbi.nlm.nih.gov/gene/?term=5800) | 1.5 | 0.00289 | 745.9 | 465.8 |
| RAB35 | RAB35, member RAS oncogene family | [11021](https://www.ncbi.nlm.nih.gov/gene/?term=11021) | 1.5 | 0.00001 | 910.7 | 586.7 |
| F8 | coagulation factor VIII | [2157](https://www.ncbi.nlm.nih.gov/gene/?term=2157) | 1.5 | 0.00596 | 159.0 | 98.2 |
| CFP | complement factor properdin | [5199](https://www.ncbi.nlm.nih.gov/gene/?term=5199) | 1.5 | 0.00416 | 4480.5 | 2787.1 |
| ZNF708 | zinc finger protein 708 | [7562](https://www.ncbi.nlm.nih.gov/gene/?term=7562) | -1.5 | 0.00015 | 618.3 | 969.9 |
| VPS13D | vacuolar protein sorting 13 homolog D | [55187](https://www.ncbi.nlm.nih.gov/gene/?term=55187) | -1.5 | 0.01256 | 1761.5 | 2887.9 |
| KPTN | kaptin (actin binding protein) | [11133](https://www.ncbi.nlm.nih.gov/gene/?term=11133) | 1.5 | 0.00344 | 127.9 | 79.6 |
| NT5C | 5', 3'-nucleotidase, cytosolic | [30833](https://www.ncbi.nlm.nih.gov/gene/?term=30833) | 1.5 | 0.00020 | 449.0 | 285.4 |
| SELT | selenoprotein T | [51714](https://www.ncbi.nlm.nih.gov/gene/?term=51714) | 1.5 | 0.00040 | 3358.6 | 2137.5 |
| ZFYVE27 | zinc finger FYVE-type containing 27 | [118813](https://www.ncbi.nlm.nih.gov/gene/?term=118813) | 1.5 | 0.00003 | 951.5 | 611.1 |
| LOC105377102 | uncharacterized LOC105377102 | [105377102](https://www.ncbi.nlm.nih.gov/gene/?term=105377102) | -1.5 | 0.04633 | 12.4 | 22.7 |
| LOC105373091 |  | [105373091](https://www.ncbi.nlm.nih.gov/gene/?term=105373091) | 1.5 | 0.06247 | 14.6 | 7.5 |
| C5orf47 | chromosome 5 open reading frame 47 | [133491](https://www.ncbi.nlm.nih.gov/gene/?term=133491) | -1.5 | 0.07019 | 3.8 | 9.2 |
| FCRL1 | Fc receptor like 1 | [115350](https://www.ncbi.nlm.nih.gov/gene/?term=115350) | -1.5 | 0.01979 | 1367.7 | 2282.2 |
| LOC105369161 |  | [105369161](https://www.ncbi.nlm.nih.gov/gene/?term=105369161) | -1.5 | 0.06135 | 152.8 | 295.7 |
| TRAJ16 | T cell receptor alpha joining 16 | [28739](https://www.ncbi.nlm.nih.gov/gene/?term=28739) | -1.5 | 0.07489 | 6.9 | 16.9 |
| KIAA1024 | KIAA1024 | [23251](https://www.ncbi.nlm.nih.gov/gene/?term=23251) | -1.5 | 0.06718 | 14.2 | 28.1 |
| LRRC70 | leucine rich repeat containing 70 | [100130733](https://www.ncbi.nlm.nih.gov/gene/?term=100130733) | 1.5 | 0.03405 | 192.2 | 111.7 |
| LOC105377921 |  | [105377921](https://www.ncbi.nlm.nih.gov/gene/?term=105377921) | 1.5 | 0.06458 | 6.8 | 2.3 |
| LOC105377070 |  | [105377070](https://www.ncbi.nlm.nih.gov/gene/?term=105377070) | -1.5 | 0.06446 | 20.9 | 55.4 |
| ANPEP | alanyl aminopeptidase, membrane | [290](https://www.ncbi.nlm.nih.gov/gene/?term=290) | -1.5 | 0.02893 | 806.9 | 1375.4 |
| TVP23CP2 | TVP23C pseudogene 2 | [261735](https://www.ncbi.nlm.nih.gov/gene/?term=261735) | -1.5 | 0.03981 | 1.6 | 8.2 |
| MAFF | v-maf avian musculoaponeurotic fibrosarcoma oncogene homolog F | [23764](https://www.ncbi.nlm.nih.gov/gene/?term=23764) | 1.5 | 0.02328 | 90.3 | 53.3 |
| ENPP3 | ectonucleotide pyrophosphatase/phosphodiesterase 3 | [5169](https://www.ncbi.nlm.nih.gov/gene/?term=5169) | -1.5 | 0.03220 | 34.3 | 59.1 |
| YY2 | YY2 transcription factor | [404281](https://www.ncbi.nlm.nih.gov/gene/?term=404281) | -1.5 | 0.03952 | 13.0 | 23.1 |
| LINC00899 | long intergenic non-protein coding RNA 899 | [100271722](https://www.ncbi.nlm.nih.gov/gene/?term=100271722) | 1.5 | 0.06247 | 30.5 | 16.1 |
| CNDP2 | CNDP dipeptidase 2 (metallopeptidase M20 family) | [55748](https://www.ncbi.nlm.nih.gov/gene/?term=55748) | 1.5 | 0.00128 | 1598.9 | 1009.2 |
[truncated: 256,769 more chars]
